# Opioids and immune checkpoint inhibitors differentially regulate a common immune network in triple-negative breast cancer

**DOI:** 10.3389/fonc.2023.1267532

**Published:** 2023-09-14

**Authors:** Joseph R. Scarpa, Giacomo Montagna, George Plitas, Amitabh Gulati, Gregory W. Fischer, Joshua S. Mincer

**Affiliations:** ^1^ Department of Anesthesiology, Weill Cornell Medicine, New York, NY, United States; ^2^ Breast Service, Department of Surgery, Memorial Sloan Kettering Cancer Center, New York, NY, United States; ^3^ Department of Anesthesiology and Critical Care Medicine, Memorial Sloan Kettering Cancer Center, New York, NY, United States

**Keywords:** opioids, ketamine, immune checkpoint inhibition, anti-PD-L1, tumor-infiltrating lymphocytes, triple-negative breast cancer

## Abstract

**Background:**

Opioids are the primary analgesics for cancer pain. Recent clinical evidence suggests opioids may counteract the effect of immune checkpoint inhibition (ICI) immunotherapy, but the mechanism for this interaction is unknown. The following experiments study how opioids and immunotherapy modulate a common RNA expression pathway in triple negative breast cancer (TNBC), a cancer subtype in which immunotherapy is increasingly used. This study identifies a mechanism by which opioids may decrease ICI efficacy, and compares ketamine, a non-opioid analgesic with emerging use in cancer pain, for potential ICI interaction.

**Methods:**

Tumor RNA expression and clinicopathologic data from a large cohort with TNBC (N=286) was used to identify RNA expression signatures of disease. Various drug-induced RNA expression profiles were extracted from multimodal RNA expression datasets and analyzed to estimate the RNA expression effects of ICI, opioids, and ketamine on TNBC.

**Results:**

We identified a RNA expression network in CD8^+^ T-cells that was relevant to TNBC pathogenesis and prognosis. Both opioids and anti-PD-L1 ICI regulated RNA expression in this network, suggesting a nexus for opioid-ICI interaction. Morphine and anti-PD-L1 therapy regulated RNA expression in opposing directions. By contrast, there was little overlap between the effect of ketamine and anti-PD-L1 therapy on RNA expression.

**Conclusions:**

Opioids and ICI may target a common immune network in TNBC and regulate gene expression in opposing fashion. No available evidence supports a similar interaction between ketamine and ICI.

## Introduction

Despite their poor side-effect profile and potential for abuse and addiction, opioids remain the primary treatment for cancer pain (1). Retrospective studies show that opioids may worsen cancer progression, an effect that varies based upon cancer type/subtype (2–4) and individual tumor genomics (5–9). Opioid-induced immunomodulation is one possible explanation for the effect of opioids on cancer. Consequently, a related but distinct question concerning opioid analgesia for cancer pain has recently been raised: can opioid use impact efficacy of immunotherapy?

Recent retrospective studies show that patients receiving immune checkpoint inhibition (ICI) have worse outcomes if they are also using opioids (10–14). While the mechanism underlying these associations is unknown, factors could range from specific effects, relying on direct crosstalk between ICI and opioid signaling (15), to more general consequences of opioid-induced immunosuppression and consequent dampening of ICI efficacy (16–18).

This study focuses on triple-negative breast cancer (TNBC), the most immunogenic breast cancer subtype with notably poor prognosis. Randomized controlled trials have demonstrated a substantial benefit from the addition of ICI to chemotherapy in TNBC (19, 20), so potential opioid-ICI interactions could be important to determine optimal analgesic strategies. Complex interactions of thousands of molecules are involved in TNBC pathogenesis and treatment response (21, 22), and systems biology methods are critical to identify how these molecules interact in networks to drive disease (23, 24). Therefore, this study identifies groups of genes that share RNA expression patterns in TNBC – “coexpression networks” – and analyzes how opioids and immunotherapy regulate them.

We hypothesize that opioids and ICI regulate the RNA expression of a common group of pathogenic genes in opposite directions. We also compare the transcriptional effect of ICI with ketamine, an emerging alternative to opioid analgesia for cancer pain (25) with potential to attenuate immunosuppression (26), to determine if ketamine and opioids compete similarly with ICI-induced RNA expression. This study explores possible mechanisms underlying ICI-opioid interactions in TNBC to corroborate the clinical epidemiological literature of ICI-opioid interactions and generate hypotheses for further experimental studies.

## Methods

### Using RNA expression data to identify TNBC gene coexpression networks

**Figure 1:**
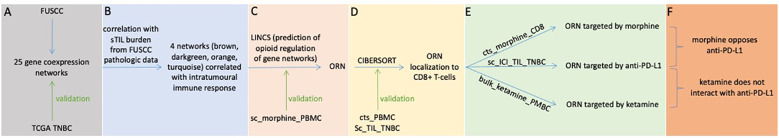
Schematic outline of the study design and a description of the RNA expression datasets used for **(A)** coexpression network analysis, **(B)** sTIL correlation, **(C)** opioid regulation, **(D)** cell-type specificity, and **(E, F)** ICI-drug interactions. CIBERSORT, Cell-type Identification by Estimating Relative Subsets of Ribonucleic Acid Transcripts; FUSCC, Fudan University Shanghai Cancer Center; LINCS, Library of Integrated Network-Based Cell Signatures; ORN, opioid-regulated stromal tumor-infiltrating lymphocytes network; PBMC, Peripheral blood mononuclear cells (lymphocytes (T-cells, B-cells, NK cells), monocytes, dendritic cells); TCGA, The Cancer Genome Atlas; TNBC, triple-negative breast cancer. Datasets are labeled as in **Table 1**.

This study integrates RNA expression data from several cohorts, all publicly accessible, detailed in **Table 1**, and fully described in **Supplementary Methods**. The entire workflow is described in **Figures 1A-F**.

**Table 1:** RNA expression datasets analyzed in this study.

Dataset ID (in this study)	Biological source	Description	Use in this study	Reference
FUSCC	TNBC tumor	Bulk RNASeq from 286 female Chinese patients with primary TNBC	Identification of gene networks and estimation of their cell-type specificity (via CIBERSORT)	(27)
TCGA TNBC	TNBC tumor	Bulk RNASeq from 162 female TCGA patients with TNBC	Validation of gene networks	(21)
LINCS	cancer cell lines	Library of RNASeq of cancer cell lines before and after drug exposure (more than 8000 compounds listed)	Drug-induced gene expression signatures for selected opioid agonists and antagonists	(28)
cts_PBMC	PBMCs	Tissue-specific RNASeq from 79 human-derived physiologically normal tissues	Validation of cell-type specificity of gene networks	(29)
sc_TIL_TNBC	TNBC intratumoral immune cells	Single-cell RNASeq of intratumoral immune cells in TNBC	Validation of cell-type specificity of gene networks	(30)
sc_morphine_PBMC	PBMCs	Single-cell RNASeq of PBMCs from 7 opioid-dependent individuals and 7 controls as well as control cells dosed with morphine	Morphine gene response signature in PBMCs	(31)
cts_morphine_CD8	CD8^+^ T-cells	Cell-type specific RNASeq of CD8^+^ T-cells from 5 donors, before and after dosing of the cells with morpine	Morphine gene response signature in CD8^+^ T-cells	(32)
sc_ICI_TIL_TNBC	TNBC intratumoral immune cells	Single-cell RNASeq of intratumoral immune cells from 22 TNBC patients who received anti-PD-L1 therapy	Anti-PD-L1 gene response signature in TNBC intratumoral immune cells	(22)
bulk_ketamine_PBMC	PBMCs	Bulk RNASeq of whole blood (PBMCs) from 26 patients with treatment-resistant depression, before and after ketamine treatment, and 21 controls	Ketamine gene response signature in PBMCs	(33)

FUSCC, Fudan University Shanghai Cancer Center; LINCS, Library of Integrated Network-Based Cell Signatures; PBMC, Peripheral blood mononuclear cells (lymphocytes (T-cells, B-cells, NK cells), monocytes, dendritic cells); RNASeq, Ribonucleic acid sequencing; TCGA, The Cancer Genome Atlas; TNBC, triple-negative breast cancer.

Note that there are three different types of RNASeq represented here: bulk, cell-type specific (cts), and single-cell (sc). Bulk RNASeq sequences RNA extracted from a large number of cells (mixed together), hence representing average gene expression across thousands of tumor cells. Cell-type specific RNASeq is bulk RNASeq, but of a specific cell-type that has been isolated from the rest. Single-cell RNASeq involves sequencing of RNA extracted from individual cells.

To study RNA expression in TNBC, we primarily examined a cohort of 465 female TNBC patients from The Fudan University Shanghai Cancer Center (“FUSCC”) (27). Clinical, histopathologic, and RNA expression data was downloaded from The National Omics Data Encyclopedia (NODE). These data included quantification of stromal tumor-infiltrating lymphocyte (sTIL) burden. A full description of the cohort is included in the **Supplementary Methods**.

Molecules, like RNA, typically do not act in isolation, but instead interact with hundreds or thousands of other molecules. Genes that share similar patterns of RNA expression form coexpression networks, where each gene can be viewed as a network node. Nodes are connected if they share a similar pattern of expression, and together they form functional units that mediate phenotypes and disease (23). For the FUSCC cohort, weighted gene coexpression analysis was used to identify these coexpression networks (34), named arbitrarily by colors. The most connected nodes in each network (“hubs”) were identified since hub genes are critical to network function and likely mediators of disease (23, 35). A second multi-ethnic dataset of TNBC patients from The Cancer Genome Atlas (21) (“TCGA TNBC”) was used as an external validation cohort to confirm that these networks were relevant to TNBC. A technical description of weighted coexpression network analysis and the methods for external validation are included in the **Supplementary Methods**.

### Determining the function and cell-type specificity of gene networks

Each network was characterized by its functional pathways and relationships to pathogenic mechanisms. This was done by comparing genes in a network to curated libraries of genes linked to specific biological pathways, cellular processes, and pathological states (**Supplementary Methods**). Network RNA expression was correlated with sTIL burden to determine which networks were likely associated with intratumoral immune response. A network-sTIL association was considered significant if Benjamini-Hochberg corrected p-values < 0.1.

Specific cell-types have unique and characteristic RNA expression signatures. By examining the RNA expression of a specific tumor sample, the proportion of each cell-type in that sample can be estimated, enabling computational estimation of the cell-type specificity of each network (via CIBERSORT) (36) (**Supplementary Methods**). A complementary approach to localizing a network to a specific cell-type is to use higher resolution RNA expression data, taken from a specific cell-type or from individual cells (**Supplementary Methods**). Therefore, we validated our computational estimation of cell-type specificity by also directly comparing networks to cell-type specific (“cts_PBMC”) and single-cell (“sc_TIL_TNBC”) RNA expression from peripheral and TNBC intratumoral immune cells, respectively (29, 30). Fisher’s exact test was used to estimate if genes shared by a given network and immune cell signature overlapped at a frequency greater than chance. A network was considered cell-type specific if p < 0.05 and odds ratio > 1.

### Identifying gene networks regulated by opioids

The Library of Integrated Network-Based Cell Signatures (“LINCS”) (28) database details effects of over 8000 compounds on expression of all genes in various cancer cell lines. Specific opioid receptor agonists and antagonists found in this database are leu-enkephalin, nalbuphine, naltrexone, and naloxone. Mapping data from LINCS, we calculated the extent to which each of these drugs alters RNA expression of genes in each network. Based on this analysis, an opioid susceptibility metric (OSM) was calculated for each network (**Supplementary Methods**). The OSM does not estimate whether a specific opioid alters RNA expression of a network. Instead, it estimates the common downstream effect on RNA expression shared by various upstream opioid agonists and antagonists. It then calculates if a network is robustly modulated by various upstream opioid-dependent pathways. This analysis enabled determination of the subset of networks predicted to be regulated by opioids and ketamine.

We also tested if human RNA expression of specific networks was altered by morphine and ketamine *in vivo*. No publicly available data is available to study how oxycodone, fentanyl, or hydromorphone alter RNA expression of immune cells. We compared our networks to genes whose expression is altered by morphine in peripheral immune cells (31) (“sc_morphine_PBMC”) in general and CD8^+^ T-cells specifically (32) (“cts_morphine_CD8”). We determined that a network was likely modulated by morphine if a significant number of genes in a network had their RNA expression altered when exposed to morphine (Fisher’s exact test p-value < 0.05, odds ratio > 1). We performed a similar experiment to identify networks likely altered by ketamine (“bulk_ketamine_PBMC”) (33).

### ICI regulation of gene networks and intersection with opioids

To determine the effect of ICI on tumor RNA expression, we investigated RNA expression from individual tumor cells taken from TNBC patients exposed to anti-PD-L1 therapy (paclitaxel and atezolizumab) (22) (“sc_ICI_TIL_TNBC”). We compared genes in each network with genes whose RNA expression was altered by ICI using Fisher’s exact test (p < 0.05, odds ratio > 1). To determine if ICI and morphine had similar effects on immune cell RNA expression, we used the Kolmogorov-Smirnov test to test if ICI and opioid resulted in a similar distribution of RNA expression. Rejecting the null hypothesis suggests that ICI and opioid result in different changes in RNA expression.

## Results

### Genome-wide RNA expression revealed TNBC coexpression networks

**Figure 2:**
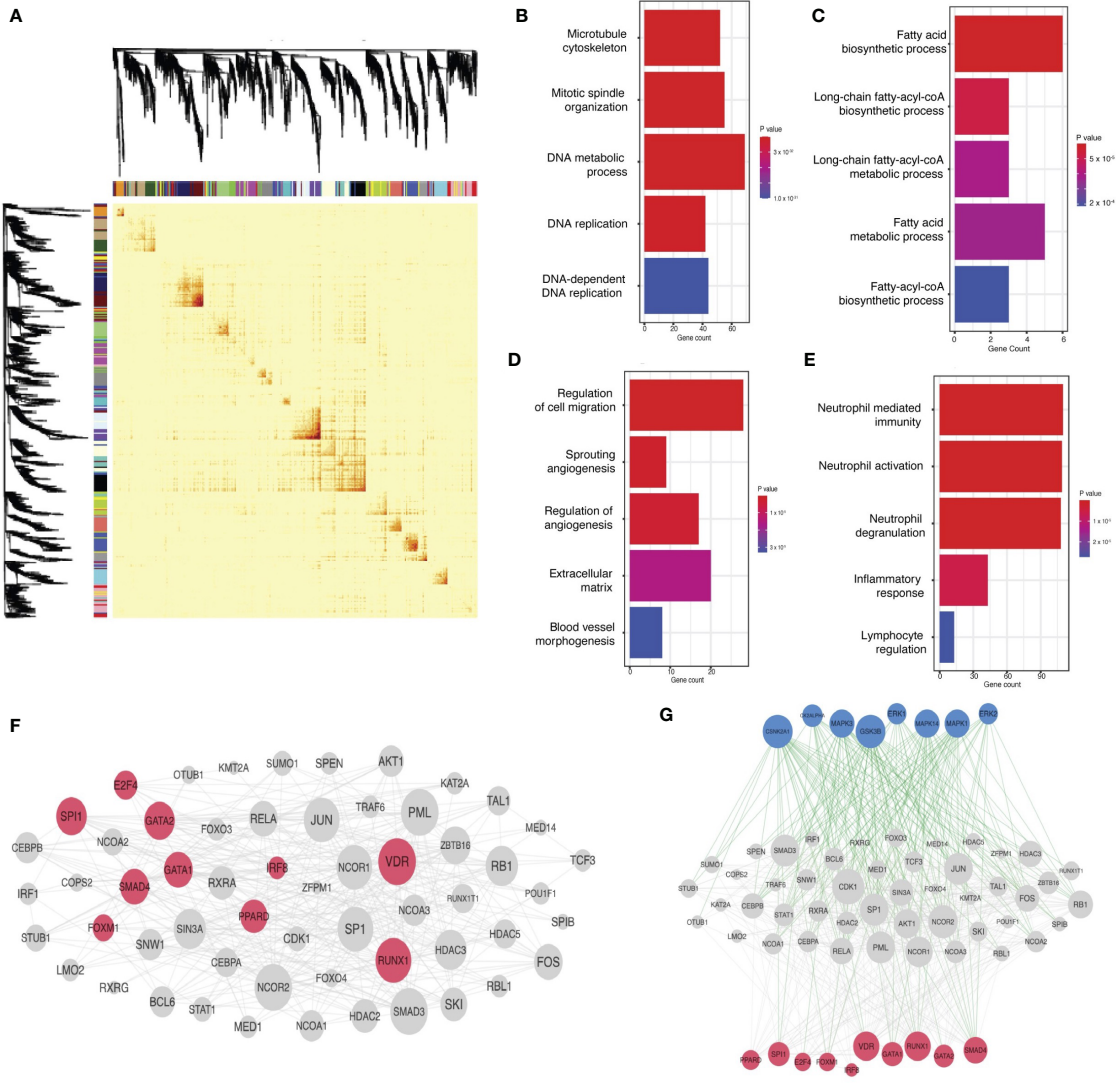
TNBC gene coexpression networks: derivation in the FUSCC cohort, functional characterization, and linkage to oncogenic mechanisms. **(A)** Weighted gene coexpression analysis separates genes with expression correlated across patients in the FUSCC cohort into distinct networks, as illustrated in the topological overlap matrix. Though not labeled, genes line the x and y axes, and any point in the matrix records the correlation between the corresponding genes in the pair (red reflects greater correlation). Each gene is also labeled by the color of the network to which it belongs (as derived in the coexpression analysis). Dendrograms and network colors are depicted on both axes, illustrating that the coexpression analysis in fact results in a clustering of genes into networks. **(B-E)** Functional characterization of networks, illustrating functionally-relevant gene sets that overlap significantly with each network for green, darkgreen, black, and brown networks, respectively: for each plot, the x-axis notes gene count within each network for respective ontology(functional) categories (labeled on the y-axis). More red signifies lower p-values. All gene pathways pictured have p < 0.05. **(F)** Linking gene networks to oncogenic mechanisms: protein-protein interaction network for transcription factors targeting network hub genes. Transcription factors predicted to directly bind hub genes are in pink and their interaction partners are in gray. **(G)** Kinase regulation of the transcription factor protein-protein interaction network: kinases are in blue, transcription factors are in pink, and their intermediate protein interaction partners are in grey. Green edges represent kinase-substrate phosphorylation interactions. Grey edges are physical protein-protein interactions.

A first step for identifying how opioids and immunotherapy may interact mechanistically in TNBC is to identify a robust molecular signature of disease (**Figure 1A**). Disease states are driven by changes in RNA expression (27, 37). Molecules, like RNA, typically do not act in isolation, but instead interact with hundreds or thousands of other molecules (23). Groups of genes sharing similar RNA expression patterns form coexpression networks that mediate disease (23, 24, 35). Therefore, we analyzed genome-wide RNA expression data from a large human cohort (**Table 1**, “FUSCC”) and calculated coexpression networks associated with TNBC (N_samples_=286, N_genes_=14,540, **Supplementary Methods**). We identified 25 coexpression networks, each named by arbitrarily assigned color (**Figure 2A**). In each network, a gene is a represented by a node. Nodes (genes) are connected if their RNA expression is strongly correlated, allowing us to identify the organization and structure of RNA expression for 14,540 genes. As demonstrated in previous studies, most nodes connect with only a few partners, while some nodes are highly-connected (23, 38). These highly-connected nodes – “hub genes” – are critical to the network as a whole and likely mediators of disease, so we also identified these hub genes for each network for downstream analyses. These networks were externally validated in an independent TNBC cohort (**Table 1**, “TCGA TNBC”) to confirm that they were robust and reproducible (**Supplementary Methods**). These analyses confirmed that 18 of the 25 networks were externally replicable, and these robust and reproducible networks were the focus of our investigation (**Figure 1A**, **Supplementary Figure 1**). The genes coexpressed in each network are listed in **Supplementary Table 1**.

### TNBC coexpression networks were associated with oncogenic mechanisms

Next, we hypothesized that these networks were associated with known cellular functions or biological pathways (**Figures 2B-E**). We identified networks associated with neutrophil-mediated immunity (“brown” network, p=1.5x10^-15^, Odds Ratio=2.99), DNA replication machinery (“green” network, p=2.5x10^-20^, Odds Ratio=39) and fatty acid biosynthesis and metabolism (“darkgreen” network, p=1.3x10^-4^, Odds Ratio = 25.8). Some networks were targets for general oncogenic transcription factors and pathways, like cell migration (p=3.9x10^-4^, Odds Ratio = 3.4), angiogenesis (p=2.6x10^-3^, Odds Ratio=4.1), and epithelial to mesenchymal transition (p=1.7x^-3^, Odds Ratio= 3.4), evidence that some networks were functionally oncogenic.

We next tested if these networks were regulated by mechanisms more specific to TNBC. This analysis revealed that network hubs – the likely mediators of network function and disease – were regulated by known oncogenic transcription factors in TNBC (**Figure 2F**), including IRF8 and SPI1, both correlated with survival and tumor immunogenicity in TNBC (39). RUNX1, another transcription factor targeting hub nodes, correlates with poor prognosis in TNBC (40). Further analysis showed that proteins known to interact with hubs are preferentially phosphorylated by MAPK and ERK pathways (**Figure 2G**), independently associated with metastatic mechanisms and prognosis in TNBC (41). These analyses supported the hypothesis that the networks calculated here were relevant to pathogenesis and prognosis in TNBC.

### Four coexpression networks were correlated with intratumoral immune response

**Figure 3:**
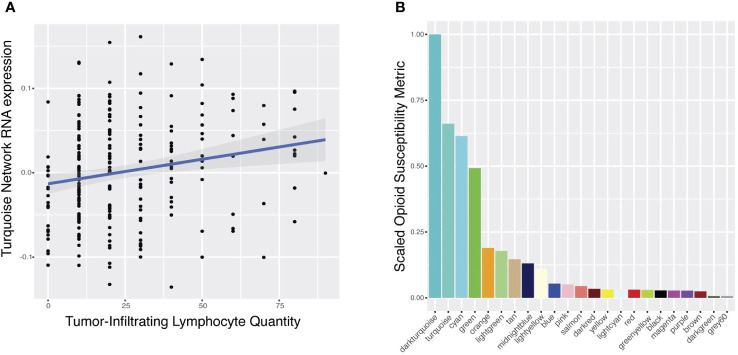
Predicted modulation of gene networks by opioid agonists and antagonists. **(A)** Correlation of RNA expression of the turquoise network (y-axis) with the quantity of stromal tumor-infiltrating lymphocytes (sTILs) measured by histopathologic analysis (x-axis). Each point represents a patient in the FUSCC cohort. **(B)** Scaled opioid susceptibility metrics (OSM) (y-axis) for each gene network (x-axis) are presented. The OSM estimates how susceptible the RNA expression of each network is in response to the common downstream pathway of various opioid receptor modulators. Color of each bar corresponds to network name, labeled along the x-axis. Only those networks (N=23) with sufficient drug-induced RNA expression data for enrichment calculations are plotted.

TNBC patients with more stromal intratumoral lymphocytes (sTILs) in the tumor microenvironment (TME) have a better prognosis, and ICI allows T-cells to destroy cancer cells. Therefore, we reasoned that specific networks correlated with sTIL burden would be strong candidates for a molecular signature where opioids and immunotherapy converge (**Figure 1B**). Four coexpression networks – brown, darkgreen, orange, and turquoise – were significantly correlated with sTIL levels across the FUSCC cohort (Benjamini-Hochberg p- value < 0.1), so we prioritized these networks in subsequent analyses. (**Figure 3A**, **Supplementary Figure 2**).

### Opioids regulated a network associated with intratumoral immune response

Next, we hypothesized that opioids would directly target TNBC coexpression networks associated with intratumoral lymphocyte burden (**Figure 1C**). We used publicly available opioid-induced RNA expression signatures from various opioids in various cancer cell lines to calculate an opioid susceptibility metric (OSM) for each network (**Supplementary Methods**). This data-driven approach estimates if RNA expression of a network is robustly altered by the downstream effects shared by various opioids, enabling selection of networks with high OSM for further analyses. The turquoise network was the sTILs network most susceptible to opioids, with the second highest OSM of all networks (**Figure 3B**).

We confirmed that turquoise is opioid-dependent in humans by studying *in vivo* RNA expression effects of morphine in human peripheral blood mononuclear cell (PBMCs) (31) (**Table 1**, “sc_morphine_PBMC”). Though peripheral and intratumoral immune cells are different, they both share similar expression patterns for opioid receptors, with high basal expression of OGFR and low expression of mu-opioid receptor (8, 42). Morphine strongly modulated the RNA expression of genes in the turquoise network (p=1.8x10^-22^, Odds Ratio=17.5), providing further evidence that turquoise was an opioid-regulated network (ORN) (**Figure 1C**). No publicly available human RNA expression data exist for other commonly prescribed opioids, like oxycodone, fentanyl, or hydromorphone, precluding similar testing.

### The ORN was localized to CD8^+^ T-cells in TNBC

**Figure 4:**
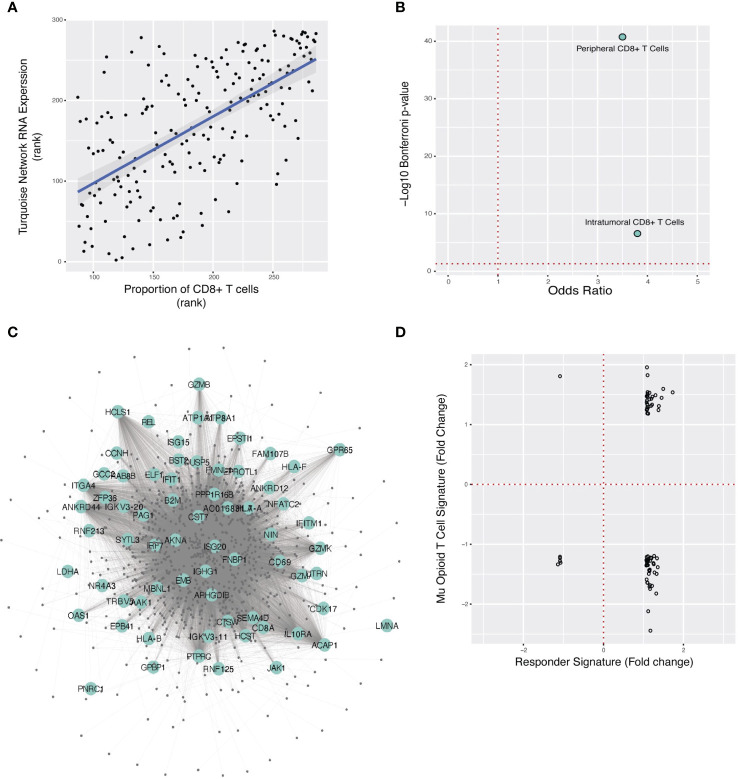
Localization of the ORN to CD8+ T-cells, overlap of ORN with TNBC anti-PD-L1 therapy response gene signature, and predicted opioid downregulation of anti-PD-L1 response. **(A)** Spearman correlation plot between turquoise network expression (“ORN”) and CIBERSORT estimate of CD8^+^ T-cell proportion for each FUSCC patient sample. **(B)**. Fisher’s exact test odds ratio and -log10 Bonferroni p-value estimating ORN significant overlap with the anti-PD-L1 gene signature in peripheral and TNBC intratumoral T-cells. **(C)** A subset of the ORN is pictured here, with large labeled nodes denoting genes whose RNA expression is modulated in immunotherapy responders in TNBC. **(D)** For the 72 genes that overlap between the morphine and anti-PD-L1 response signatures, the change in RNA expression for each gene in response to morphine (y-axis) and immunotherapy (x-axis) is respectively illustrated. Each point is one of the overlapping genes.

Since opioids targeted a network associated with tumor-infiltrating lymphocyte burden, we reasoned that opioids primarily affected a specific lymphocyte type (**Figure 1D**). Computational estimation of cell-type specificity of each network (**Supplementary Methods**) revealed that the ORN was primarily a CD8^+^ T-cell coexpression network (rho=0.66, Benjamini-Hochberg p-value = 4.4x10^-37^) (**Figure 4A**). We confirmed this by comparing the ORN with an independent CD8^+^ T-cell specific RNA expression signature (29) (**Table 1**, “cts_PBMC”, p=1.8x10^-41^, Odds Ratio=3.5) (**Figure 4B**).

To directly validate that the ORN is specific to T-cell RNA expression from the TNBC microenvironment, we studied single-cell RNA expression data collected from TNBC tumors (30) (**Table 1**, “sc_TIL_TNBC”) (**Figure 4B**). Genes coexpressed in the ORN were primarily genes specific to intratumoral CD8^+^ effector T-cells in TNBC, further corroborating that the ORN was specific to CD8^+^ in TNBC tumors (p=2.8x10^-7^, Odds Ratio=3.8).

### Gene response to anti-PD-L1 therapy in TNBC targeted the ORN

T-cell regulation of the TME influences tumor progression and mediates the therapeutic activity of ICI (43, 44). Localization of the ORN to CD8+ T-cells in the TME suggested a possible nexus for opioid-ICI interaction (**Figure 1E**) if ICI targeted the ORN as well. We tested this hypothesis by studying intratumoral RNA expression data from TNBC patients receiving paclitaxel and atezolizumab (22) (**Table 1**, “sc_ICI_TIL_TNBC”). We identified the intratumoral RNA expression signature that predicted ICI response and compared it to the ORN, demonstrating overlap between the anti-PD-L1 gene response signature and the ORN (p = 1.8x10^-16^, Odds Ratio=2.2) (**Figure 4C**). This suggested that the ORN was critical to immunotherapy response.

### Opioids opposed gene expression response to anti-PD-L1 therapy in the ORN

To study if opioids and anti-PD-L1 therapy had similar or opposing effects on intratumoral RNA expression, we first studied whether opioid and immunotherapy RNA expression signatures intersected directly at all. This analysis showed that anti-PD-L1 response signature strongly overlapped with morphine-induced differential expression in human CD8^+^ T-cells (32) (**Table 1**, “cts_morphine_CD8”). (p=7.9x10^-3^, Odds Ratio=1.4).

Next, we examined how morphine and immunotherapy changed RNA expression of the 72 genes common to their gene response signatures (**Figure 1F**). The two pharmacological interventions typically altered RNA expression of genes in opposing directions (**Figure 4D**). For example, morphine downregulated 54% of genes upregulated in the anti-PD-L1 signature. Further analysis showed that morphine and anti-PD-L1 resulted in different distributions of expression fold change for the 72 overlapping genes, suggesting they affect RNA expression differently (D = 0.56944, Kolmogorov-Smirnov two-sided p-value=1.45 x 10^-10^, **Supplementary Figure 3**). Taken together, these two analyses supported the conclusion that these two interventions have different and opposing effects on the same core genes.

### Ketamine did not oppose the anti-PD-L1 gene response signature

We tested whether ketamine had a similar effect (**Figure 1F**). When comparing the anti-PD-L1 signature to the ketamine response signature in PBMCs (33) (**Table 1**, “bulk_ketamine_PBMC”), we found that only three genes overlapped – PER1, TSC22D3, and DUSP1 – far fewer than the 72 genes that overlapped between the opioid and anti-PD-L1 signatures. All three genes were modulated in the same direction for both therapies. This suggested that ketamine and anti-PD-L1 did not modulate the expression of common genes, and those genes that were commonly modulated shared similar directionality.

## Discussion

The mechanistic role of opioids in cancer is complex. Single-cell RNA expression data shows that opioid receptors vary in expression by cell-type in the TME, with some opioid-related genes more highly expressed in TME T-cells than in tumor (8). OGFR, the non-canonical opioid receptor, has strong basal levels of expression in both TNBC TME T-cells (8) and peripheral T-cells (42). Notably, the mu-opioid receptor has minimal RNA expression in the TME but has been shown to be inducible in peripheral lymphocytes by various stimuli, including chronic opioids (45). Opioid receptors in the TME may enable direct opioid action but do not constitute a detailed mechanism to explain opioid effects on cancer or opioid-ICI interaction. Consequently, this study leverages large genome-wide RNA expression datasets to elucidate a more detailed molecular hypothesis: opioids and ICI target a common gene coexpression network localized to intratumoral CD8^+^ effector T-cells but regulate its RNA expression in opposite directions.

Localization of opioid-ICI interaction to CD8^+^ T-cells is consistent with recent research highlighting the role of CD8^+^ TILs specifically in mediating the response to immunotherapy (44) and is consistent with analyses of gene expression in TNBC (8) and colon adenocarcinoma (6). Interestingly, opioids were associated with anti-tumor effects in these cancer types, suggesting the possibility that opioids can have anti-tumor effects on the tumor itself while at the same time countering the efficacy of ICI. Ketamine, an emerging analgesic in general and in cancer specifically (25), may be worth considering for patients in whom opioids would negatively affect immunotherapy since it does not alter RNA expression of genes necessary for clinical response to ICI. Ketamine is a very promiscuous drug, targeting NMDA receptors among many others, and competing effects across various receptors may account for its overall net neutral effect on ICI targets of RNA expression. These insights may have significant clinical implications, suggesting that a precision medicine approach may be needed to identify appropriate analgesic options for patients based on their tumor subtype or immunotherapy regimens. This may become increasingly important since both perioperative and intraoperative immunotherapy show promise (46, 47), extending the relevance of opioid-ICI interactions beyond the chronic cancer pain patient to all early-stage cancer patients undergoing tumor resection.

These analyses should be interpreted cautiously since we do not directly examine the effect of both opioids (or ketamine) and ICI simultaneously on human tumor *in vivo*, and instead rely on inferences made from integrating multiple experimental datasets. Another limitation of our study was the paucity of public data testing the *in vivo* molecular effects of various synthetic (fentanyl, oxycodone, or hydromorphone) and endogenous opioids on human RNA expression in immune cells or tumor. This limited our ability to differentiate between opioid types and understand how endogenous opioid-ICI interactions may influence outcomes, while at the same time highlighting the need for a systematic understanding of how different opioids affect RNA expression in immunologic and tumor tissue. Lastly, our proof-of-principle analysis focuses solely on TNBC and should not be interpreted in the context of other cancers, however, we note that the role of effector CD8^+^ T cells in mediating outcomes has been well documented across numerous cancer types, suggesting that opioid-ICI interactions in other cancer types is worth further study.

Rather than justifying any immediate change in clinical practice, our study was designed to generate the first plausible, specific data-driven hypotheses to explain opioid-ICI interaction. These findings provide strong justification for future experiments focused on studying the direct effects of opioids and ICI in human TNBC *in vivo* in a randomized control trial or an organoid-based system. Our analyses also suggest that further investigation of ketamine as an alternative to opioids in ICI patients is warranted.

## Data availability statement

Publicly available datasets were analyzed in this study. All data employed in this study is publicly available at the respective references cited.

## Ethics statement

Ethical approval was not required for the study involving humans in accordance with the local legislation and institutional requirements. Written informed consent to participate in this study was not required from the participants or the participants’ legal guardians/next of kin in accordance with the national legislation and the institutional requirements.

## Author contributions

JS: Conceptualization, Data curation, Formal Analysis, Investigation, Methodology, Software, Validation, Visualization, Writing – original draft, Writing – review & editing. GM: Conceptualization, Investigation, Methodology, Validation, Writing – review & editing. GP: Methodology, Writing – review & editing. AG: Writing – review & editing. GF: Project administration, Resources, Supervision, Writing – review & editing. JM: Conceptualization, Formal Analysis, Investigation, Methodology, Project administration, Resources, Supervision, Validation, Visualization, Writing – original draft, Writing – review & editing.
