# Supplementary material for: Opioids and immune checkpoint inhibitors differentially regulate a common immune network in triple-negative breast cancer
Source: Front Oncol. 2023 Sep 14;13:1267532. doi: 10.3389/fonc.2023.1267532 (PMC10539607; doi:10.3389/fonc.2023.1267532)
Supplement: Supplementary file 1 [file DataSheet_1.docx]

Opioids and immune checkpoint inhibitors differentially regulate a common immune network in triple-negative breast cancer

Joseph R. Scarpa^1^, Giacomo Montagna^2^, George Plitas^2^, Amitabh Gulati^1,3^, Gregory W. Fischer^1,3^, Joshua S. Mincer^1,3*^

^1^Department of Anesthesiology, Weill Cornell Medicine, New York, NY, USA

^2^Breast Service, Department of Surgery, Memorial Sloan Kettering Cancer Center, New York, NY, USA

^3^Department of Anesthesiology and Critical Care Medicine, Memorial Sloan Kettering Cancer Center, New York, NY, USA

**SUPPLEMENTARY INFORMATION**

**SUPPLEMENTARY METHODS**

**Types of RNA expression data**

The studies in this manuscript measured RNA expression using ribonucleic acid sequencing (RNASeq). This experimental method can be conducted at different degrees of resolution to capture different components of cellular biology. RNAseq can be performed on bulk tissue, on specific cell types, or on individual single cells. Bulk RNASeq sequences RNA extracted from a large number of cells (mixed together), hence representing average gene expression across thousands of cells. Cell-type specific RNASeq is bulk RNASeq, but of a specific cell type that has been isolated from the rest. Single-cell RNASeq involves sequencing of RNA extracted from individual cells. The type of RNA expression experiment is described for each dataset in Table 1.

**Cohort characteristics and biospecimen collection**

Clinical and biospecimen data was previously collected and made publicly available by investigators from the Fudan University Shanghai Cancer Center (FUSCC) cohort(1). Briefly, this cohort includes female patients diagnosed with unilateral invasive ductal carcinoma with an ER-, PR- and HER2- phenotype (confirmed by immunochemical analysis and in situ hybridization), with no evidence of distant metastasis at diagnosis. Patients with breast carcinoma in situ or with inflammatory breast cancer were excluded. Tumor tissue was macrodissected, so that tumor cells made up at least 50% of the final sample. Tumor infilitrating lymphocytes (TILs) were evaluated on hematoxylin and eosin–stained sections. Stromal TILs and intratumoral TILs were quantified separately.

**Gene expression data in triple-negative breast cancer cohorts**

Previously published RNA-sequencing gene expression data from the Fudan University Shanghai Cancer Center (FUSCC) cohort(1) was downloaded from The National Omics Data Encyclopedia (NODE). Tumor samples were macrodissected. Stromal tumor infiltrating lymphocyte burden was calculated by histopathologic assessment. Covariates were evaluated by fitting a linear model for each gene and comparing the distribution of model p-values against uniform distribution and were finally adjusted for by fitting the expression data with a robust linear model and taking intercept + residuals as the adjusted expression values. RNA-sequencing gene expression data for triple-negative cases were also retrieved from The Cancer Gene Atlas (TCGA), and covariate correction was performed using the same methodology.

**Gene coexpression network calculation and validation**

Weighted gene coexpression analysis was used to identify gene expression networks(2). Pearson correlation was calculated between each gene pair and an adjacency was derived by raising correlation matrix to a power, β, that approximates scale-free topology. The adjacency matrix was quadratically transformed into a topological overlap matrix (TOM) to estimate nearest-neighbor associations(3). Hierarchical clustering defined gene network were assigned arbitrary labels (names or colors)(4). For each network, connectivity was calculated for each gene. Hub genes were identified as the top 10% most connected genes within each network. Two tests were used to assess the external validity of these networks in the TCGA TNBC cohort – a network membership test and network connectivity test. To test connectivity, a conservation metric was calculated for each network. For each network, the difference between the Pearson gene-gene correlation in the FUSCC and TCGA cohorts was calculated. The conservation metric was derived by calculating the mean of the absolute of this difference in correlation matrices. A conservation metric of zero reflects perfect correspondence between the two cohorts. A false discovery rate was calculated for each conservation metric by permuting genes in the TCGA cohort 10,000 times, re-calculating the conservation metric for each permutation, and comparing it to the true value. To test network membership conservation, coexpression networks were independently calculated in the TCGA TNBC cohort using the same procedure as described. Fisher’s exact test was used to estimate network member overlap between FUSCC and TCGA networks. A FUSCC network was only considered validated if it met two criteria – it was overrepresented for at least one TCGA network and its connectivity conservation metric was FDR < 0.01.

**Functional and phenotypic characterization of coexpression networks**

To functionally characterize coexpressoin networks, various gene signatures were analyzed using Enrichr(5), including gene ontology molecular function and biological process categories. Bonferroni corrected p values and odds ratios are reported. Hub genes were further analyzed in order to link networks more directly to oncogenic mechanisms. First, transcription factor enrichment for hub genes was performed and the protein-interaction network of these transcription factors were calculated using the Genes2Networks algorithm(6). Kinase enrichment analysis was then performed to predict kinase regulators of this expanded transcription factor protein-interaction network and eXpression2Kinases was used to display the interactions(7).

To identify networks relevant to sTIL burden, the eigengene was calculated for each network and correlated with sTIL counts, correcting for tumor grade. Network-sTILs correlation was considered significant if Benjamini-Hochberg p-value < 0.1.

**Estimating cell-type specificity for gene coexpression networks**

CIBERSORT was used to estimate various cell-type proportions for each sample in the FUSCC cohort(8). Spearman correlation between network eigengene and cell-type proportion was performed to identify network-relevance for various cell-types (Benjamini-Hochberg p < 0.05), and the primary cell-type identity of a network was determined by ranking cell-types by Spearman correlation coefficient (ρ). Validation of cell-type specificity was performed by estimating network enrichment by Fisher’s exact test for two RNA expression signatures specific to T-cells. The first was derived from human tissue-specific gene expression profiles in protein-encoding genes(9) and the second was from single-cell RNA-seq collected from breast tumor microenvironment(10) .

**Estimating opioid susceptibility of each network**

To estimate opioid effects on gene networks, PharmacoGx was used(11). First, median gene expression was calculated for each gene in the FUSCC cohort and converted to Z-scores. Next, drug-induced gene expression signatures were procured from the Library of Integrated Network-Based Cell Signatures (LINCS)(12,13) for the following opioid agonists and antagonists: leu-enkephalin, nalbuphine, naltrexone, and naloxone. For each network, gene set enrichment analysis then was used to estimate similarity between gene expression in TNBC and after opioid exposure(14). P-values were estimated after 1000 permutations. A drug-network pair was considered significant if Benjamini-Hochberg p-value < 0.05. Based on this analysis, an opioid susceptibility metric (OSM) was calculated for each network by summing its connectivity scores for leu-enkephalin, nalbuphine, naltrexone, and naloxone. Connectivity scores associated with a p < 0.05 were weighted fully, while connectivity scores with p > 0.05 were first penalized (connectivity score*0.1) before being added to the OSM.

**REFERENCES**

1. Jiang Y-Z, Ma D, Suo C, Shi J, Xue M, Hu X, Xiao Y, Yu K-D, Liu Y-R, Yu Y, et al. Genomic and Transcriptomic Landscape of Triple-Negative Breast Cancers: Subtypes and Treatment Strategies. *Cancer Cell* (2019) 35:428-440.e5. doi: 10.1016/j.ccell.2019.02.001

2. Zhang B, Horvath S. A general framework for weighted gene co-expression network analysis. *Stat Appl Genet Mol* (2005) 4:Article17. doi: 10.2202/1544-6115.1128

3. Ravasz E, Somera AL, Mongru DA, Oltvai ZN, Barabási AL. Hierarchical organization of modularity in metabolic networks. *Science* (2002) 297:1551 1555. doi: 10.1126/science.1073374

4. Langfelder P, Zhang B, Horvath S. Defining clusters from a hierarchical cluster tree: the Dynamic Tree Cut package for R. *Bioinformatics* (2007) 24:719–720. doi: 10.1093/bioinformatics/btm563

5. Chen EY, Tan CM, Kou Y, Duan Q, Wang Z, Meirelles GV, Clark NR, Ma’ayan A. Enrichr: interactive and collaborative HTML5 gene list enrichment analysis tool. *Bmc Bioinformatics* (2013) 14:128. doi: 10.1186/1471-2105-14-128

6. Berger SI, Posner JM, Ma’ayan A. Genes2Networks: connecting lists of gene symbols using mammalian protein interactions databases. *Bmc Bioinformatics* (2007) 8:372. doi: 10.1186/1471-2105-8-372

7. Clarke DJB, Kuleshov MV, Schilder BM, Torre D, Duffy ME, Keenan AB, Lachmann A, Feldmann AS, Gundersen GW, Silverstein MC, et al. eXpression2Kinases (X2K) Web: linking expression signatures to upstream cell signaling networks. *Nucleic Acids Res* (2018) 46:gky458-. doi: 10.1093/nar/gky458

8. Newman AM, Liu CL, Green MR, Gentles AJ, Feng W, Xu Y, Hoang CD, Diehn M, Alizadeh AA. Robust enumeration of cell subsets from tissue expression profiles. *Nat Methods* (2015) 12:453–457. doi: 10.1038/nmeth.3337

9. Su AI, Wiltshire T, Batalov S, Lapp H, Ching KA, Block D, Zhang J, Soden R, Hayakawa M, Kreiman G, et al. A gene atlas of the mouse and human protein-encoding transcriptomes. *Proc National Acad Sci* (2004) 101:6062–6067. doi: 10.1073/pnas.0400782101

10. Azizi E, Carr AJ, Plitas G, Cornish AE, Konopacki C, Prabhakaran S, Nainys J, Wu K, Kiseliovas V, Setty M, et al. Single-Cell Map of Diverse Immune Phenotypes in the Breast Tumor Microenvironment. *Cell* (2018) 174:1293-1308.e36. doi: 10.1016/j.cell.2018.05.060

11. Smirnov P, Safikhani Z, El-Hachem N, Wang D, She A, Olsen C, Freeman M, Selby H, Gendoo DMA, Grossmann P, et al. PharmacoGx: an R package for analysis of large pharmacogenomic datasets. *Bioinform Oxf Engl* (2015) 32:1244–6. doi: 10.1093/bioinformatics/btv723

12. Keenan AB, Jenkins SL, Jagodnik KM, Koplev S, He E, Torre D, Wang Z, Dohlman AB, Silverstein MC, Lachmann A, et al. The Library of Integrated Network-Based Cellular Signatures NIH Program: System-Level Cataloging of Human Cells Response to Perturbations. *Cell Syst* (2018) 6:13–24. doi: 10.1016/j.cels.2017.11.001

13. Subramanian A, Narayan R, Corsello SM, Peck DD, Natoli TE, Lu X, Gould J, Davis JF, Tubelli AA, Asiedu JK, et al. A Next Generation Connectivity Map: L1000 Platform and the First 1,000,000 Profiles. *Cell* (2017) 171:1437-1452.e17. doi: 10.1016/j.cell.2017.10.049

14. Väremo L, Nielsen J, Nookaew I. Enriching the gene set analysis of genome-wide data by incorporating directionality of gene expression and combining statistical hypotheses and methods. *Nucleic Acids Res* (2013) 41:4378–4391. doi: 10.1093/nar/gkt111

**SUPPLEMENTARY FIGURES**

**Supplementary Figure 1. Testing the robustness and reproducibility of coexpression networks in an independent cohort.**

**
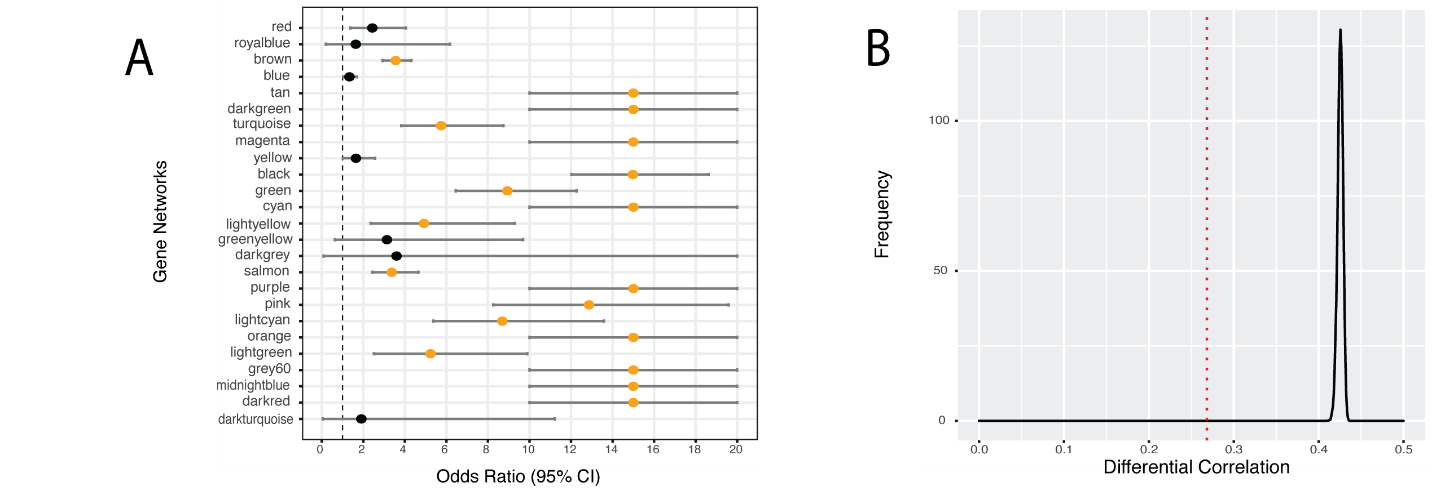
**

(A) Validation of gene networks in TCGA: Fisher’s exact test odds ratio and 95% confidence interval (CI) are pictured along the x-axis for each network on the y-axis, corresponding to network membership conservation in the TCGA TNBC cohort. Yellow nodes denote p < 0.05. All enrichment scores with odds ratios > 15 were automatically set to 15 with 95% CI 10-20 for purposes of visualization. (B) X axis reflects differential correlation between “black” network genes in the FUSCC and TCGA TNBC cohorts. A score of zero signifies perfect agreement of gene-gene correlation between the two cohorts. Red vertical line is the true differential correlation. The black histogram reflects the distribution of differential correlation for 10,000 permutations. This plot signifies that the “black” network correlation is conserved between the two cohorts with an FDR < 0.0001.

**Supplementary Figure 2. Correlating various coexpression networks with sTIL burden.**


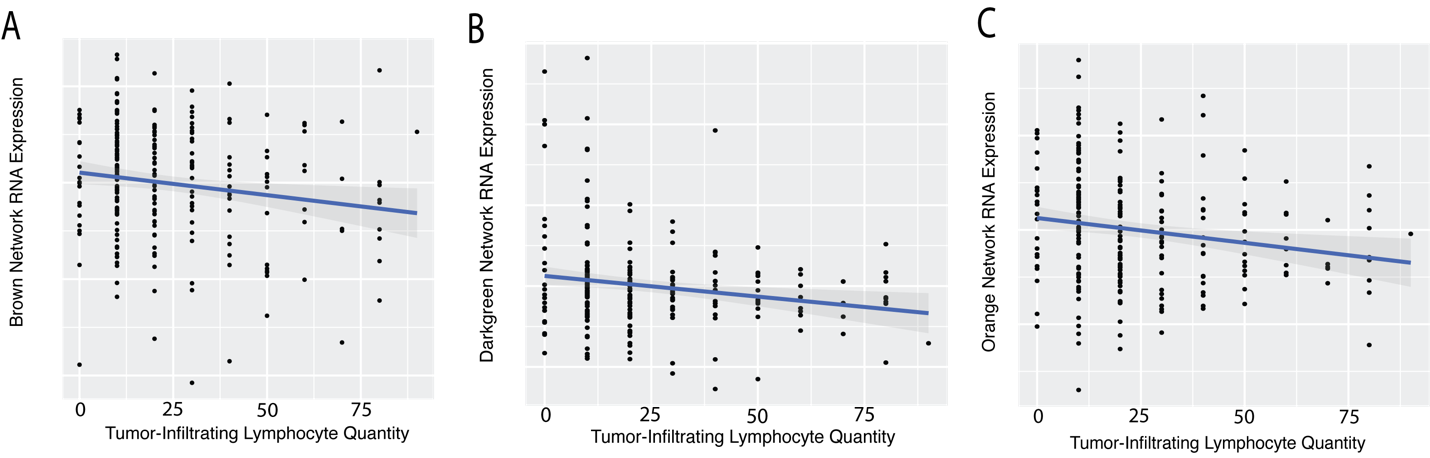


**(**A-C) Correlation of coexpression networks with sTIL counts: correlation between network expression (y-axis) and sTIL counts (x-axis) for brown, darkgreen, and orange networks, respectively.

**Supplementary Figure 3. Differential effects of morphine and anti-PD-L1 therapy on gene expression.**

**
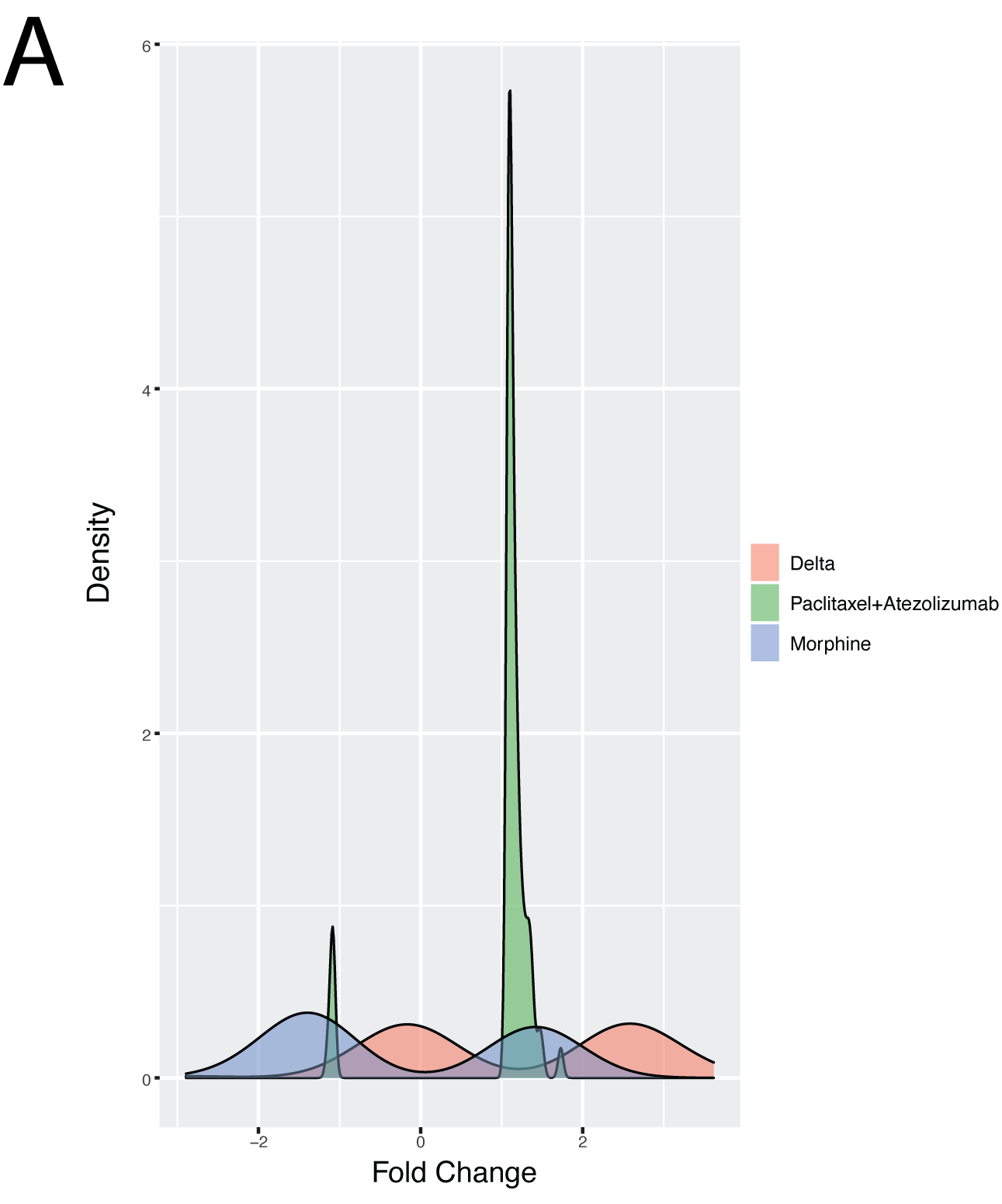
**

(A) Histogram of the change in RNA expression for the 72 overlapping genes, pictured separately for morphine (blue) and anti-PD-L1 therapy (green). A third (red) distribution – “delta”, or the calculated difference between the morphine and immunotherapy fold change – is included.

**SUPPLEMENTARY TABLE 1. Coexpression network membership of each gene.**

| GENE | NETWORK |
| --- | --- |
| 5_8S_rRNA | red |
| 5S_rRNA | royalblue |
| 7SK | brown |
| A1BG | blue |
| A26C1B | tan |
| A2LD1 | brown |
| A2M | blue |
| A2ML1 | blue |
| A4GALT | blue |
| AACS | tan |
| AADACL1 | brown |
| AADAT | darkgreen |
| AAK1 | turquoise |
| AAMP | blue |
| AARS | turquoise |
| AARS2 | magenta |
| AARSD1 | yellow |
| AASS | black |
| AB019439.3 | turquoise |
| ABAT | blue |
| ABCA1 | brown |
| ABCA10 | black |
| ABCA12 | tan |
| ABCA13 | turquoise |
| ABCA17P | brown |
| ABCA2 | yellow |
| ABCA3 | brown |
| ABCA4 | brown |
| ABCA5 | blue |
| ABCA6 | black |
| ABCA7 | turquoise |
| ABCA8 | black |
| ABCA9 | black |
| ABCB1 | turquoise |
| ABCB10 | green |
| ABCB6 | brown |
| ABCB8 | yellow |
| ABCB9 | turquoise |
| ABCC1 | yellow |
| ABCC10 | yellow |
| ABCC11 | tan |
| ABCC2 | tan |
| ABCC3 | brown |
| ABCC4 | turquoise |
| ABCC5 | blue |
| ABCC9 | black |
| ABCD1 | brown |
| ABCD3 | red |
| ABCD4 | black |
| ABCE1 | green |
| ABCF1 | magenta |
| ABCF2 | blue |
| ABCF3 | yellow |
| ABCG1 | turquoise |
| ABHD1 | yellow |
| ABHD10 | blue |
| ABHD11 | turquoise |
| ABHD12 | blue |
| ABHD13 | turquoise |
| ABHD14A | tan |
| ABHD14B | blue |
| ABHD15 | turquoise |
| ABHD2 | blue |
| ABHD3 | brown |
| ABHD4 | blue |
| ABHD5 | turquoise |
| ABHD6 | brown |
| ABHD8 | blue |
| ABI2 | cyan |
| ABI3 | turquoise |
| ABI3BP | turquoise |
| ABL1 | blue |
| ABL2 | blue |
| ABLIM1 | blue |
| ABLIM2 | blue |
| ABLIM3 | blue |
| ABR | turquoise |
| ABTB1 | turquoise |
| ABTB2 | brown |
| AC000036.5 | lightyellow |
| AC000078.5 | yellow |
| AC000089.3 | blue |
| AC000120.7 | blue |
| AC000123.4 | turquoise |
| AC002055.4 | turquoise |
| AC002115.6 | yellow |
| AC002117.1 | blue |
| AC002303.1 | turquoise |
| AC002310.1 | brown |
| AC002365.4 | yellow |
| AC002464.1 | greenyellow |
| AC002467.7 | yellow |
| AC002472.9 | blue |
| AC002553.2 | turquoise |
| AC003043.2 | green |
| AC003080.4 | brown |
| AC003104.1 | turquoise |
| AC003665.1 | blue |
| AC003963.1 | turquoise |
| AC003989.4 | blue |
| AC004016.2 | turquoise |
| AC004041.2 | darkgrey |
| AC004057.1 | salmon |
| AC004086.1 | salmon |
| AC004128.1 | yellow |
| AC004142.3 | yellow |
| AC004221.1 | yellow |
| AC004223.1 | yellow |
| AC004258.1 | blue |
| AC004381.6 | blue |
| AC004383.4 | black |
| AC004386.1 | blue |
| AC004410.1 | yellow |
| AC004448.1 | red |
| AC004448.3 | salmon |
| AC004453.8 | blue |
| AC004460.1 | green |
| AC004471.9 | blue |
| AC004538.3 | purple |
| AC004611.1 | turquoise |
| AC004696.1 | pink |
| AC004771.1 | yellow |
| AC004803.1 | cyan |
| AC004840.9 | turquoise |
| AC004841.1 | brown |
| AC004866.1 | red |
| AC004878.2 | yellow |
| AC004884.1 | yellow |
| AC004895.2 | greenyellow |
| AC004906.3 | turquoise |
| AC004917.1 | blue |
| AC004932.1 | yellow |
| AC004945.2 | blue |
| AC004951.6 | yellow |
| AC004967.11 | turquoise |
| AC004967.8 | blue |
| AC004968.2 | brown |
| AC004980.10 | yellow |
| AC004980.7 | yellow |
| AC004980.9 | yellow |
| AC004985.13 | turquoise |
| AC004988.1 | lightcyan |
| AC005007.3 | blue |
| AC005013.5 | turquoise |
| AC005013.6 | tan |
| AC005020.1 | turquoise |
| AC005035.1 | blue |
| AC005037.3 | turquoise |
| AC005037.4 | turquoise |
| AC005037.5 | turquoise |
| AC005042.2 | brown |
| AC005042.4 | brown |
| AC005062.2 | turquoise |
| AC005076.1 | salmon |
| AC005083.1 | turquoise |
| AC005086.1 | royalblue |
| AC005104.3 | turquoise |
| AC005105.2 | blue |
| AC005152.2 | brown |
| AC005154.5 | lightyellow |
| AC005154.6 | turquoise |
| AC005154.7 | turquoise |
| AC005189.1 | turquoise |
| AC005197.2 | turquoise |
| AC005236.1 | greenyellow |
| AC005239.1 | blue |
| AC005258.1 | turquoise |
| AC005301.5 | blue |
| AC005324.1 | blue |
| AC005332.1 | turquoise |
| AC005332.2 | turquoise |
| AC005484.1 | darkgrey |
| AC005498.2 | pink |
| AC005517.1 | turquoise |
| AC005517.3 | turquoise |
| AC005534.6 | brown |
| AC005534.9 | yellow |
| AC005540.3 | turquoise |
| AC005592.2 | turquoise |
| AC005594.1 | darkgrey |
| AC005609.12 | orange |
| AC005618.2 | orange |
| AC005670.1 | turquoise |
| AC005674.1 | turquoise |
| AC005682.5 | blue |
| AC005696.1 | blue |
| AC005696.2 | greenyellow |
| AC005726.6 | yellow |
| AC005754.1 | orange |
| AC005840.2 | cyan |
| AC005841.1 | turquoise |
| AC005895.3 | blue |
| AC005946.1 | brown |
| AC006027.2 | darkgrey |
| AC006028.10 | yellow |
| AC006039.5 | pink |
| AC006057.3 | brown |
| AC006132.1 | greenyellow |
| AC006160.1 | turquoise |
| AC006276.1 | blue |
| AC006357.1 | blue |
| AC006367.1 | blue |
| AC006368.1 | red |
| AC006427.1 | blue |
| AC006460.2 | blue |
| AC006461.2 | blue |
| AC006465.3 | turquoise |
| AC006480.2 | brown |
| AC006504.1 | blue |
| AC006504.2 | turquoise |
| AC006504.3 | green |
| AC006547.8 | yellow |
| AC006946.12 | turquoise |
| AC006953.1 | purple |
| AC006978.1 | turquoise |
| AC006978.6 | turquoise |
| AC006994.1 | black |
| AC007000.10 | yellow |
| AC007009.1 | brown |
| AC007014.1 | blue |
| AC007038.7 | turquoise |
| AC007064.24 | turquoise |
| AC007098.1 | turquoise |
| AC007099.1 | blue |
| AC007130.1 | brown |
| AC007246.3 | blue |
| AC007254.3 | brown |
| AC007255.7 | blue |
| AC007272.3 | turquoise |
| AC007276.4 | yellow |
| AC007279.2 | pink |
| AC007283.5 | turquoise |
| AC007319.1 | black |
| AC007362.1 | brown |
| AC007383.3 | blue |
| AC007383.4 | red |
| AC007383.5 | brown |
| AC007384.1 | yellow |
| AC007384.3 | greenyellow |
| AC007390.5 | red |
| AC007401.2 | blue |
| AC007431.1 | yellow |
| AC007436.2 | turquoise |
| AC007450.3 | darkgreen |
| AC007551.2 | turquoise |
| AC007563.5 | lightyellow |
| AC007566.10 | black |
| AC007599.1 | red |
| AC007620.3 | blue |
| AC007639.1 | turquoise |
| AC007690.1 | turquoise |
| AC007743.1 | darkgrey |
| AC007773.1 | brown |
| AC007790.3 | yellow |
| AC007875.2 | turquoise |
| AC007875.3 | blue |
| AC007878.1 | blue |
| AC007879.5 | turquoise |
| AC007899.3 | turquoise |
| AC007919.19 | brown |
| AC007963.1 | black |
| AC008013.2 | cyan |
| AC008038.1 | salmon |
| AC008062.1 | blue |
| AC008068.1 | pink |
| AC008073.5 | blue |
| AC008074.1 | brown |
| AC008083.1 | turquoise |
| AC008268.3 | red |
| AC008280.1 | turquoise |
| AC008280.2 | blue |
| AC008280.4 | blue |
| AC008280.5 | brown |
| AC008392.1 | turquoise |
| AC008403.1 | yellow |
| AC008427.1 | blue |
| AC008440.5 | lightyellow |
| AC008537.3 | yellow |
| AC008541.1 | red |
| AC008567.1 | pink |
| AC008733.3 | pink |
| AC008734.1 | lightgreen |
| AC008734.2 | lightgreen |
| AC008735.1 | blue |
| AC008740.1 | turquoise |
| AC008740.2 | turquoise |
| AC008746.11 | yellow |
| AC008746.5 | turquoise |
| AC008763.1 | royalblue |
| AC008865.2 | turquoise |
| AC008865.3 | blue |
| AC008870.1 | pink |
| AC008895.1 | yellow |
| AC008937.2 | turquoise |
| AC008969.1 | pink |
| AC008993.1 | yellow |
| AC009041.3 | yellow |
| AC009053.2 | yellow |
| AC009060.3 | yellow |
| AC009065.1 | blue |
| AC009065.4 | yellow |
| AC009086.1 | turquoise |
| AC009086.2 | blue |
| AC009086.4 | blue |
| AC009116.1 | pink |
| AC009118.3 | brown |
| AC009120.3 | turquoise |
| AC009133.2 | greenyellow |
| AC009237.1 | brown |
| AC009237.11 | brown |
| AC009237.8 | brown |
| AC009299.2 | turquoise |
| AC009299.3 | turquoise |
| AC009318.1 | turquoise |
| AC009403.2 | turquoise |
| AC009404.2 | blue |
| AC009452.1 | greenyellow |
| AC009475.2 | blue |
| AC009501.4 | brown |
| AC009501.5 | brown |
| AC009505.1 | turquoise |
| AC009505.2 | magenta |
| AC009506.1 | blue |
| AC009520.1 | turquoise |
| AC009533.2 | cyan |
| AC009570.1 | blue |
| AC009753.1 | greenyellow |
| AC009908.1 | brown |
| AC009948.3 | blue |
| AC009948.5 | turquoise |
| AC009950.2 | turquoise |
| AC009951.2 | brown |
| AC009961.3 | turquoise |
| AC009967.5 | red |
| AC010149.1 | turquoise |
| AC010150.1 | turquoise |
| AC010168.1 | cyan |
| AC010226.4 | turquoise |
| AC010240.1 | yellow |
| AC010319.2 | turquoise |
| AC010326.2 | pink |
| AC010326.5 | yellow |
| AC010329.1 | pink |
| AC010333.1 | black |
| AC010442.1 | turquoise |
| AC010491.1 | turquoise |
| AC010522.1 | pink |
| AC010522.2 | pink |
| AC010524.1 | turquoise |
| AC010531.3 | turquoise |
| AC010536.1 | yellow |
| AC010614.1 | pink |
| AC010615.2 | pink |
| AC010619.1 | salmon |
| AC010632.1 | greenyellow |
| AC010655.1 | turquoise |
| AC010679.2 | turquoise |
| AC010733.1 | turquoise |
| AC010733.5 | turquoise |
| AC010761.3 | blue |
| AC010886.1 | black |
| AC011236.8 | yellow |
| AC011247.2 | greenyellow |
| AC011288.2 | blue |
| AC011322.1 | blue |
| AC011394.1 | brown |
| AC011406.2 | turquoise |
| AC011442.1 | brown |
| AC011450.1 | yellow |
| AC011460.1 | darkgreen |
| AC011468.1 | blue |
| AC011476.1 | magenta |
| AC011477.1 | pink |
| AC011479.1 | turquoise |
| AC011484.1 | blue |
| AC011484.2 | brown |
| AC011498.2 | blue |
| AC011526.1 | black |
| AC011816.4 | turquoise |
| AC011899.9 | brown |
| AC011933.2 | turquoise |
| AC011994.3 | greenyellow |
| AC012044.2 | yellow |
| AC012066.1 | brown |
| AC012085.2 | turquoise |
| AC012087.2 | blue |
| AC012100.1 | brown |
| AC012146.7 | salmon |
| AC012170.2 | pink |
| AC012305.1 | magenta |
| AC012309.3 | pink |
| AC012318.1 | turquoise |
| AC012358.4 | yellow |
| AC012358.7 | pink |
| AC012360.6 | blue |
| AC012363.12 | turquoise |
| AC012363.5 | red |
| AC012379.1 | blue |
| AC012379.2 | turquoise |
| AC012409.1 | blue |
| AC012487.2 | blue |
| AC012513.3 | yellow |
| AC012513.4 | yellow |
| AC012652.1 | blue |
| AC013267.2 | brown |
| AC013268.5 | brown |
| AC013272.3 | brown |
| AC013283.1 | blue |
| AC013414.2 | pink |
| AC013418.3 | blue |
| AC013461.1 | blue |
| AC013476.1 | yellow |
| AC013553.1 | blue |
| AC015550.1 | turquoise |
| AC015712.1 | blue |
| AC015712.2 | brown |
| AC015720.2 | blue |
| AC015802.2 | turquoise |
| AC015802.3 | grey60 |
| AC015813.2 | turquoise |
| AC015845.2 | red |
| AC015849.1 | turquoise |
| AC015936.1 | green |
| AC015971.2 | red |
| AC015976.3 | blue |
| AC015982.2 | black |
| AC015987.1 | greenyellow |
| AC016525.3 | turquoise |
| AC016590.1 | pink |
| AC016596.2 | darkgrey |
| AC016601.1 | turquoise |
| AC016683.5 | yellow |
| AC016683.6 | blue |
| AC016725.4 | yellow |
| AC016734.1 | brown |
| AC016735.2 | brown |
| AC016736.2 | blue |
| AC016738.1 | black |
| AC016745.3 | brown |
| AC016747.3 | blue |
| AC016831.7 | turquoise |
| AC016894.1 | turquoise |
| AC016906.1 | turquoise |
| AC016995.3 | cyan |
| AC017002.1 | brown |
| AC017002.2 | brown |
| AC017002.3 | blue |
| AC017048.3 | blue |
| AC017071.1 | turquoise |
| AC017074.2 | red |
| AC017076.1 | red |
| AC017076.4 | lightcyan |
| AC017099.3 | blue |
| AC017101.10 | lightcyan |
| AC017104.2 | blue |
| AC017116.11 | turquoise |
| AC017116.8 | turquoise |
| AC018500.3 | blue |
| AC018557.1 | turquoise |
| AC018607.1 | red |
| AC018628.3 | turquoise |
| AC018633.4 | blue |
| AC018638.2 | yellow |
| AC018642.1 | brown |
| AC018648.6 | yellow |
| AC018720.10 | yellow |
| AC018737.1 | turquoise |
| AC018755.1 | turquoise |
| AC018766.1 | pink |
| AC018809.6 | brown |
| AC018816.3 | blue |
| AC018816.4 | turquoise |
| AC018865.8 | tan |
| AC018890.6 | blue |
| AC018892.1 | red |
| AC018926.2 | brown |
| AC019014.1 | blue |
| AC019097.7 | yellow |
| AC019100.5 | tan |
| AC019171.4 | turquoise |
| AC019171.5 | turquoise |
| AC019181.3 | blue |
| AC019186.1 | blue |
| AC019206.3 | brown |
| AC020659.1 | turquoise |
| AC020907.6 | yellow |
| AC020915.1 | pink |
| AC020915.2 | pink |
| AC020915.4 | darkgreen |
| AC020922.1 | brown |
| AC020926.1 | darkgreen |
| AC020928.1 | pink |
| AC020928.2 | pink |
| AC021049.1 | cyan |
| AC021054.1 | cyan |
| AC021593.1 | turquoise |
| AC021593.2 | turquoise |
| AC021937.1 | yellow |
| AC022001.1 | yellow |
| AC022007.1 | brown |
| AC022007.5 | brown |
| AC022080.1 | brown |
| AC022087.1 | pink |
| AC022087.3 | turquoise |
| AC022098.2 | brown |
| AC022098.3 | brown |
| AC022150.1 | blue |
| AC022165.1 | brown |
| AC022173.2 | blue |
| AC022311.1 | yellow |
| AC022405.2 | turquoise |
| AC022415.4 | blue |
| AC022498.1 | yellow |
| AC022498.2 | blue |
| AC022596.1 | blue |
| AC022692.2 | blue |
| AC022826.1 | red |
| AC023024.2 | green |
| AC023055.2 | lightyellow |
| AC023105.2 | blue |
| AC023162.1 | royalblue |
| AC023509.1 | yellow |
| AC023590.1 | turquoise |
| AC023632.1 | brown |
| AC023818.1 | blue |
| AC023818.3 | blue |
| AC023818.4 | black |
| AC023830.4 | yellow |
| AC024075.1 | blue |
| AC024270.1 | blue |
| AC024560.1 | darkgrey |
| AC024560.3 | yellow |
| AC024575.2 | blue |
| AC024896.1 | cyan |
| AC024902.1 | cyan |
| AC024937.1 | yellow |
| AC024940.2 | cyan |
| AC024952.1 | yellow |
| AC025171.1 | blue |
| AC025260.1 | red |
| AC025260.4 | blue |
| AC025272.1 | yellow |
| AC025279.1 | turquoise |
| AC025562.1 | darkgrey |
| AC025857.1 | brown |
| AC025917.1 | turquoise |
| AC025918.2 | yellow |
| AC026150.5 | black |
| AC026191.1 | greenyellow |
| AC026202.3 | turquoise |
| AC026271.1 | brown |
| AC026271.4 | magenta |
| AC026333.1 | blue |
| AC026444.1 | yellow |
| AC026468.1 | yellow |
| AC027097.3 | blue |
| AC027125.2 | yellow |
| AC027237.2 | blue |
| AC027277.1 | turquoise |
| AC027607.1 | blue |
| AC027612.6 | blue |
| AC027644.1 | blue |
| AC027644.2 | pink |
| AC027708.1 | turquoise |
| AC027763.1 | black |
| AC027763.2 | turquoise |
| AC034193.5 | yellow |
| AC034220.3 | brown |
| AC037459.4 | purple |
| AC037487.1 | brown |
| AC040934.1 | blue |
| AC040975.1 | blue |
| AC040980.1 | grey60 |
| AC044839.2 | red |
| AC046143.5 | turquoise |
| AC046176.3 | brown |
| AC046185.1 | turquoise |
| AC055740.1 | royalblue |
| AC055764.1 | blue |
| AC055839.1 | turquoise |
| AC055876.1 | black |
| AC058791.1 | turquoise |
| AC058791.2 | turquoise |
| AC060780.1 | yellow |
| AC062017.1 | turquoise |
| AC062037.3 | turquoise |
| AC063943.1 | brown |
| AC063965.3 | black |
| AC064836.1 | red |
| AC064852.5 | turquoise |
| AC066692.4 | blue |
| AC067945.1 | blue |
| AC068152.1 | blue |
| AC068288.1 | black |
| AC068302.1 | yellow |
| AC068302.3 | brown |
| AC068400.1 | yellow |
| AC068446.3 | blue |
| AC068491.1 | lightcyan |
| AC068491.2 | lightcyan |
| AC068491.4 | lightcyan |
| AC068533.1 | brown |
| AC068542.1 | red |
| AC068580.3 | yellow |
| AC068580.6 | brown |
| AC068587.1 | blue |
| AC068594.1 | red |
| AC068888.1 | yellow |
| AC069209.1 | brown |
| AC069213.1 | brown |
| AC069213.4 | turquoise |
| AC069214.1 | yellow |
| AC069234.1 | yellow |
| AC069257.9 | turquoise |
| AC069259.1 | brown |
| AC069271.1 | turquoise |
| AC069277.2 | yellow |
| AC069281.1 | turquoise |
| AC069282.6 | blue |
| AC069287.3 | yellow |
| AC069360.2 | turquoise |
| AC069513.3 | yellow |
| AC069513.4 | brown |
| AC073043.2 | blue |
| AC073046.25 | yellow |
| AC073046.4 | brown |
| AC073052.1 | turquoise |
| AC073058.1 | blue |
| AC073130.3 | yellow |
| AC073210.1 | yellow |
| AC073254.1 | greenyellow |
| AC073283.4 | brown |
| AC073283.7 | yellow |
| AC073342.1 | blue |
| AC073342.12 | brown |
| AC073342.2 | greenyellow |
| AC073343.11 | yellow |
| AC073346.2 | turquoise |
| AC073410.1 | blue |
| AC073422.1 | greenyellow |
| AC073502.1 | yellow |
| AC073573.2 | blue |
| AC073621.1 | turquoise |
| AC073834.1 | red |
| AC073869.1 | greenyellow |
| AC073871.2 | turquoise |
| AC073995.2 | red |
| AC074092.1 | turquoise |
| AC074093.1 | black |
| AC074138.1 | pink |
| AC074138.3 | pink |
| AC074286.1 | turquoise |
| AC074289.1 | turquoise |
| AC074338.2 | greenyellow |
| AC074367.1 | brown |
| AC078784.1 | darkgrey |
| AC078794.2 | greenyellow |
| AC078799.3 | turquoise |
| AC078802.1 | turquoise |
| AC078843.2 | turquoise |
| AC078883.3 | turquoise |
| AC078883.4 | turquoise |
| AC078953.2 | blue |
| AC078983.1 | turquoise |
| AC079096.1 | turquoise |
| AC079305.10 | greenyellow |
| AC079305.9 | brown |
| AC079316.1 | turquoise |
| AC079588.1 | brown |
| AC079601.3 | blue |
| AC079628.1 | blue |
| AC079807.2 | yellow |
| AC079807.3 | turquoise |
| AC079922.4 | turquoise |
| AC079944.1 | brown |
| AC079949.1 | red |
| AC080112.3 | blue |
| AC083799.4 | blue |
| AC083868.1 | turquoise |
| AC083871.2 | turquoise |
| AC083875.2 | blue |
| AC083883.1 | yellow |
| AC083884.7 | red |
| AC083906.1 | yellow |
| AC083949.1 | turquoise |
| AC083982.1 | yellow |
| AC084018.1 | yellow |
| AC084031.1 | turquoise |
| AC084082.3 | royalblue |
| AC084083.1 | midnightblue |
| AC084083.2 | midnightblue |
| AC084125.1 | darkred |
| AC084251.1 | turquoise |
| AC087071.1 | brown |
| AC087071.2 | yellow |
| AC087257.1 | lightcyan |
| AC087284.1 | yellow |
| AC087284.2 | yellow |
| AC087289.2 | grey60 |
| AC087590.3 | turquoise |
| AC087650.3 | salmon |
| AC087651.1 | greenyellow |
| AC087742.1 | greenyellow |
| AC087749.3 | black |
| AC087854.1 | purple |
| AC087859.1 | darkgrey |
| AC087885.1 | greenyellow |
| AC089999.2 | blue |
| AC090018.3 | turquoise |
| AC090186.1 | midnightblue |
| AC090360.1 | red |
| AC090421.1 | brown |
| AC090517.2 | blue |
| AC090691.1 | turquoise |
| AC090699.1 | grey60 |
| AC090774.1 | blue |
| AC090804.1 | turquoise |
| AC090937.2 | blue |
| AC090945.1 | brown |
| AC091047.2 | red |
| AC091062.2 | blue |
| AC091103.1 | turquoise |
| AC091132.2 | turquoise |
| AC091132.4 | turquoise |
| AC091167.3 | brown |
| AC091320.1 | blue |
| AC091565.3 | blue |
| AC091565.4 | blue |
| AC091588.1 | blue |
| AC091729.9 | turquoise |
| AC091736.1 | brown |
| AC091736.5 | greenyellow |
| AC091805.1 | cyan |
| AC091814.3 | yellow |
| AC091849.1 | blue |
| AC092024.2 | blue |
| AC092037.1 | blue |
| AC092123.1 | blue |
| AC092139.1 | blue |
| AC092139.2 | turquoise |
| AC092143.2 | yellow |
| AC092145.1 | brown |
| AC092168.2 | turquoise |
| AC092171.2 | brown |
| AC092171.4 | turquoise |
| AC092198.1 | yellow |
| AC092214.10 | turquoise |
| AC092279.1 | red |
| AC092279.4 | pink |
| AC092295.1 | pink |
| AC092295.3 | brown |
| AC092295.7 | pink |
| AC092296.1 | magenta |
| AC092296.3 | pink |
| AC092329.2 | turquoise |
| AC092365.1 | black |
| AC092375.4 | turquoise |
| AC092375.5 | turquoise |
| AC092415.1 | darkgrey |
| AC092431.1 | blue |
| AC092490.1 | cyan |
| AC092490.2 | cyan |
| AC092535.3 | blue |
| AC092536.1 | yellow |
| AC092594.1 | yellow |
| AC092597.3 | brown |
| AC092610.2 | yellow |
| AC092620.2 | blue |
| AC092653.5 | turquoise |
| AC092669.4 | blue |
| AC092798.2 | turquoise |
| AC092801.1 | blue |
| AC092835.2 | magenta |
| AC092865.1 | turquoise |
| AC092964.2 | blue |
| AC093038.1 | pink |
| AC093110.3 | blue |
| AC093162.5 | yellow |
| AC093264.4 | red |
| AC093323.3 | brown |
| AC093375.1 | brown |
| AC093376.2 | yellow |
| AC093391.2 | yellow |
| AC093415.2 | brown |
| AC093420.1 | brown |
| AC093484.6 | yellow |
| AC093520.2 | greenyellow |
| AC093525.1 | yellow |
| AC093620.5 | turquoise |
| AC093627.10 | blue |
| AC093627.6 | brown |
| AC093642.3 | blue |
| AC093642.5 | yellow |
| AC093673.5 | yellow |
| AC093724.2 | blue |
| AC093726.6 | turquoise |
| AC093734.11 | blue |
| AC093734.13 | turquoise |
| AC093818.1 | turquoise |
| AC093827.1 | turquoise |
| AC093838.4 | yellow |
| AC093850.2 | lightcyan |
| AC093927.1 | red |
| AC094019.4 | blue |
| AC094107.1 | blue |
| AC096579.14 | turquoise |
| AC096579.7 | turquoise |
| AC096637.2 | greenyellow |
| AC096772.6 | blue |
| AC096921.2 | turquoise |
| AC096947.1 | blue |
| AC097359.2 | turquoise |
| AC097381.1 | blue |
| AC097461.4 | yellow |
| AC097500.2 | pink |
| AC097532.2 | red |
| AC097636.1 | greenyellow |
| AC097724.3 | brown |
| AC098479.1 | brown |
| AC098691.1 | red |
| AC098818.1 | brown |
| AC098820.3 | brown |
| AC098824.5 | darkgreen |
| AC099057.8 | green |
| AC099508.1 | yellow |
| AC099524.1 | yellow |
| AC099535.3 | turquoise |
| AC099548.1 | yellow |
| AC099557.1 | turquoise |
| AC099778.1 | yellow |
| AC099850.1 | grey60 |
| AC100748.2 | brown |
| AC100778.1 | salmon |
| AC100784.1 | blue |
| AC100786.1 | salmon |
| AC100788.1 | lightyellow |
| AC100791.2 | yellow |
| AC103560.1 | blue |
| AC103681.1 | brown |
| AC103686.3 | midnightblue |
| AC103705.1 | darkred |
| AC103706.1 | turquoise |
| AC103810.3 | grey60 |
| AC103810.4 | grey60 |
| AC103965.1 | magenta |
| AC104066.1 | blue |
| AC104115.1 | turquoise |
| AC104184.1 | turquoise |
| AC104186.1 | turquoise |
| AC104307.1 | yellow |
| AC104452.2 | turquoise |
| AC104597.1 | turquoise |
| AC104651.2 | brown |
| AC104651.3 | brown |
| AC104655.2 | brown |
| AC104699.1 | turquoise |
| AC104772.1 | yellow |
| AC104942.3 | red |
| AC105049.1 | brown |
| AC105049.2 | darkred |
| AC105210.1 | darkred |
| AC105219.1 | darkred |
| AC105287.1 | red |
| AC105287.2 | brown |
| AC105427.1 | blue |
| AC106017.4 | turquoise |
| AC106722.1 | blue |
| AC106782.1 | yellow |
| AC106782.2 | turquoise |
| AC106782.4 | turquoise |
| AC106782.5 | red |
| AC106782.7 | turquoise |
| AC106788.3 | turquoise |
| AC106827.2 | yellow |
| AC106873.4 | yellow |
| AC107016.2 | turquoise |
| AC107021.1 | lightcyan |
| AC107033.1 | royalblue |
| AC107081.5 | green |
| AC107204.1 | darkgrey |
| AC107241.1 | turquoise |
| AC107377.1 | grey60 |
| AC107883.1 | blue |
| AC107977.10 | turquoise |
| AC107977.2 | turquoise |
| AC107977.4 | turquoise |
| AC107983.1 | blue |
| AC107983.3 | blue |
| AC108062.1 | turquoise |
| AC108078.1 | red |
| AC108134.3 | pink |
| AC108134.8 | brown |
| AC108477.4 | yellow |
| AC108488.3 | blue |
| AC108488.4 | blue |
| AC108667.1 | brown |
| AC108670.1 | brown |
| AC108860.1 | brown |
| AC109826.2 | turquoise |
| AC110491.1 | blue |
| AC110615.2 | red |
| AC110792.1 | blue |
| AC110926.1 | turquoise |
| AC111170.2 | yellow |
| AC111170.3 | yellow |
| AC112211.2 | turquoise |
| AC112229.1 | turquoise |
| AC112497.2 | greenyellow |
| AC112504.1 | yellow |
| AC112512.1 | yellow |
| AC112641.1 | purple |
| AC112777.1 | red |
| AC113189.5 | greenyellow |
| AC113420.1 | darkgreen |
| AC114546.1 | red |
| AC114730.11 | yellow |
| AC114760.1 | turquoise |
| AC114772.2 | blue |
| AC114810.5 | yellow |
| AC115110.1 | greenyellow |
| AC115115.3 | turquoise |
| AC116340.1 | red |
| AC116366.6 | turquoise |
| AC116552.1 | turquoise |
| AC116904.1 | red |
| AC116914.1 | green |
| AC117378.1 | yellow |
| AC117834.1 | blue |
| AC118344.5 | yellow |
| AC119673.2 | green |
| AC120194.1 | greenyellow |
| AC121251.1 | turquoise |
| AC121253.1 | red |
| AC122108.1 | turquoise |
| AC122179.1 | brown |
| AC122688.1 | turquoise |
| AC123788.1 | yellow |
| AC123789.1 | blue |
| AC124283.1 | grey60 |
| AC124312.1 | blue |
| AC124319.1 | turquoise |
| AC124319.2 | turquoise |
| AC124781.1 | brown |
| AC124781.2 | yellow |
| AC124798.1 | blue |
| AC124914.3 | blue |
| AC124944.1 | brown |
| AC124944.2 | yellow |
| AC126118.1 | midnightblue |
| AC126118.2 | turquoise |
| AC126389.2 | red |
| AC126544.1 | yellow |
| AC126755.1 | yellow |
| AC127024.1 | turquoise |
| AC127496.1 | yellow |
| AC127496.3 | grey60 |
| AC127904.2 | yellow |
| AC128648.1 | blue |
| AC130304.1 | greenyellow |
| AC130343.2 | brown |
| AC130352.1 | turquoise |
| AC131025.8 | black |
| AC131097.3 | blue |
| AC131182.1 | blue |
| AC131263.3 | yellow |
| AC131571.1 | yellow |
| AC131649.1 | turquoise |
| AC131971.1 | turquoise |
| AC132217.4 | black |
| AC132812.1 | grey60 |
| AC132872.1 | yellow |
| AC132872.2 | yellow |
| AC133111.1 | yellow |
| AC133528.2 | blue |
| AC133552.1 | blue |
| AC133555.2 | darkgrey |
| AC133555.6 | yellow |
| AC133555.7 | greenyellow |
| AC133644.1 | turquoise |
| AC133919.4 | yellow |
| AC133919.6 | turquoise |
| AC133961.1 | brown |
| AC134669.1 | red |
| AC134684.1 | greenyellow |
| AC135050.1 | turquoise |
| AC135457.1 | turquoise |
| AC135724.1 | blue |
| AC135983.5 | blue |
| AC136007.2 | blue |
| AC136443.1 | yellow |
| AC137055.1 | turquoise |
| AC137055.2 | yellow |
| AC137834.1 | purple |
| AC137932.2 | yellow |
| AC138028.2 | yellow |
| AC138035.4 | yellow |
| AC138128.1 | blue |
| AC138230.1 | greenyellow |
| AC138409.1 | yellow |
| AC138645.1 | turquoise |
| AC138749.1 | blue |
| AC138783.12 | yellow |
| AC138894.4 | brown |
| AC138932.1 | yellow |
| AC138951.1 | yellow |
| AC138951.2 | yellow |
| AC138969.1 | yellow |
| AC138969.2 | yellow |
| AC139099.1 | brown |
| AC139272.1 | blue |
| AC139453.1 | brown |
| AC139495.1 | blue |
| AC139666.1 | magenta |
| AC139769.1 | brown |
| AC139769.3 | brown |
| AC140076.1 | turquoise |
| AC140134.1 | blue |
| AC140481.1 | brown |
| AC141586.5 | yellow |
| AC142528.1 | blue |
| AC144441.1 | turquoise |
| AC144449.1 | turquoise |
| AC144450.2 | turquoise |
| AC144521.1 | turquoise |
| AC144530.1 | turquoise |
| AC144568.3 | yellow |
| AC145098.3 | blue |
| AC145123.1 | darkred |
| AC145146.1 | blue |
| AC145207.1 | turquoise |
| AC145285.3 | turquoise |
| AC145676.2 | salmon |
| AC146944.4 | blue |
| AC146949.1 | blue |
| AC149644.1 | black |
| AC159540.1 | red |
| AC159540.14 | red |
| AC159540.2 | red |
| AC174470.1 | grey60 |
| AC215219.1 | yellow |
| AC217773.3 | turquoise |
| AC226119.2 | yellow |
| AC233309.1 | cyan |
| ACAA1 | tan |
| ACAA2 | brown |
| ACACA | turquoise |
| ACACB | black |
| ACAD11 | blue |
| ACAD8 | tan |
| ACAD9 | brown |
| ACADL | tan |
| ACADM | tan |
| ACADS | turquoise |
| ACADSB | blue |
| ACADVL | lightyellow |
| ACAN | turquoise |
| ACAP1 | turquoise |
| ACAP2 | turquoise |
| ACAP3 | yellow |
| ACAT1 | yellow |
| ACAT2 | green |
| ACBD3 | green |
| ACBD4 | turquoise |
| ACBD5 | green |
| ACBD7 | brown |
| ACCS | black |
| ACD | blue |
| ACE | brown |
| ACE2 | tan |
| ACER2 | blue |
| ACER3 | blue |
| ACLY | yellow |
| ACN9 | blue |
| ACO1 | blue |
| ACO2 | yellow |
| ACOT1 | yellow |
| ACOT11 | brown |
| ACOT13 | magenta |
| ACOT2 | blue |
| ACOT4 | tan |
| ACOT7 | green |
| ACOT9 | brown |
| ACOX1 | grey60 |
| ACOX2 | blue |
| ACOX3 | yellow |
| ACP1 | brown |
| ACP2 | brown |
| ACP5 | brown |
| ACP6 | brown |
| ACPL2 | yellow |
| ACPP | blue |
| ACSBG1 | darkgreen |
| ACSF2 | blue |
| ACSF3 | turquoise |
| ACSL1 | tan |
| ACSL3 | turquoise |
| ACSL4 | brown |
| ACSL5 | turquoise |
| ACSL6 | turquoise |
| ACSM1 | darkgreen |
| ACSM3 | darkgreen |
| ACSS1 | turquoise |
| ACSS2 | brown |
| ACSS3 | darkgreen |
| ACTA1 | blue |
| ACTA2 | black |
| ACTB | lightcyan |
| ACTG1 | grey60 |
| ACTG1P10 | pink |
| ACTG2 | blue |
| ACTGP3 | turquoise |
| ACTL6A | blue |
| ACTN1 | purple |
| ACTN4 | turquoise |
| ACTR3B | brown |
| ACTR3C | blue |
| ACTR6 | blue |
| ACVR1 | lightcyan |
| ACVR1B | yellow |
| ACVR1C | black |
| ACVR2A | blue |
| ACVR2B | magenta |
| ACVRL1 | black |
| ACY1 | turquoise |
| ACY3 | turquoise |
| ACYP1 | yellow |
| ACYP2 | brown |
| AD000090.2 | purple |
| AD000671.1 | yellow |
| AD000671.3 | yellow |
| ADA | turquoise |
| ADAM1 | turquoise |
| ADAM12 | purple |
| ADAM15 | turquoise |
| ADAM19 | turquoise |
| ADAM22 | turquoise |
| ADAM28 | turquoise |
| ADAM32 | turquoise |
| ADAM33 | black |
| ADAM8 | brown |
| ADAM9 | purple |
| ADAMDEC1 | turquoise |
| ADAMTS1 | black |
| ADAMTS10 | turquoise |
| ADAMTS12 | purple |
| ADAMTS13 | yellow |
| ADAMTS14 | purple |
| ADAMTS16 | lightcyan |
| ADAMTS17 | blue |
| ADAMTS2 | purple |
| ADAMTS3 | blue |
| ADAMTS4 | brown |
| ADAMTS5 | blue |
| ADAMTS6 | blue |
| ADAMTS7 | lightcyan |
| ADAMTS9 | black |
| ADAMTSL1 | black |
| ADAMTSL2 | lightcyan |
| ADAMTSL3 | blue |
| ADAMTSL4 | blue |
| ADAP1 | brown |
| ADAP2 | brown |
| ADAR | turquoise |
| ADARB1 | blue |
| ADAT1 | yellow |
| ADAT2 | turquoise |
| ADAT3 | yellow |
| ADC | turquoise |
| ADCK2 | blue |
| ADCK4 | yellow |
| ADCK5 | darkred |
| ADCY2 | turquoise |
| ADCY3 | yellow |
| ADCY4 | black |
| ADCY6 | turquoise |
| ADCY7 | turquoise |
| ADCY9 | lightyellow |
| ADD1 | blue |
| ADD2 | cyan |
| ADD3 | turquoise |
| ADH1B | black |
| ADH5 | red |
| ADHFE1 | blue |
| ADI1 | turquoise |
| ADIPOR2 | cyan |
| ADK | blue |
| ADM | lightcyan |
| ADM2 | blue |
| ADNP | greenyellow |
| ADORA1 | blue |
| ADORA2A | turquoise |
| ADORA2B | turquoise |
| ADORA3 | brown |
| ADPRH | brown |
| ADPRHL1 | turquoise |
| ADPRHL2 | green |
| ADRA2A | black |
| ADRB2 | turquoise |
| ADRBK1 | turquoise |
| ADRBK2 | turquoise |
| ADRM1 | blue |
| ADSL | blue |
| ADSS | green |
| ADSSL1 | turquoise |
| AE000661.26 | turquoise |
| AEBP1 | purple |
| AEBP2 | cyan |
| AEN | blue |
| AES | brown |
| AF011889.2 | pink |
| AF011889.4 | pink |
| AF011889.5 | turquoise |
| AF111168.3 | greenyellow |
| AF111169.2 | turquoise |
| AF127577.10 | blue |
| AF127577.11 | blue |
| AF127936.7 | blue |
| AF129408.15 | yellow |
| AF131215.1 | turquoise |
| AF131215.2 | turquoise |
| AF131215.3 | turquoise |
| AF146191.4 | blue |
| AF178030.2 | blue |
| AF205589.2 | darkred |
| AF207550.1 | turquoise |
| AF224669.3 | brown |
| AF235103.2 | darkred |
| AFAP1 | yellow |
| AFAP1L1 | black |
| AFAP1L2 | blue |
| AFF1 | turquoise |
| AFF3 | black |
| AFG3L1 | yellow |
| AFG3L2 | blue |
| AFMID | tan |
| AGA | blue |
| AGAP1 | magenta |
| AGAP10 | yellow |
| AGAP11 | black |
| AGAP2 | turquoise |
| AGAP3 | yellow |
| AGAP6 | yellow |
| AGAP8 | yellow |
| AGBL3 | black |
| AGBL5 | magenta |
| AGER | magenta |
| AGFG1 | red |
| AGFG2 | turquoise |
| AGK | yellow |
| AGL | red |
| AGPAT2 | blue |
| AGPAT3 | yellow |
| AGPAT4 | blue |
| AGPAT5 | blue |
| AGPAT6 | lightgreen |
| AGPAT9 | blue |
| AGPHD1 | turquoise |
| AGPS | turquoise |
| AGRN | yellow |
| AGTPBP1 | turquoise |
| AGTRAP | blue |
| AGXT2L2 | turquoise |
| AHCTF1 | green |
| AHCY | blue |
| AHCYL2 | yellow |
| AHDC1 | yellow |
| AHI1 | pink |
| AHNAK | blue |
| AHNAK2 | purple |
| AHR | brown |
| AHRR | turquoise |
| AHSA1 | green |
| AHSA2 | blue |
| AIF1 | brown |
| AIF1L | brown |
| AIFM1 | blue |
| AIFM2 | turquoise |
| AIFM3 | brown |
| AIG1 | blue |
| AIM1 | blue |
| AIM1L | blue |
| AIM2 | turquoise |
| AIMP2 | blue |
| AJ003147.3 | turquoise |
| AK1 | blue |
| AK2 | blue |
| AK3 | turquoise |
| AK5 | blue |
| AKAP1 | grey60 |
| AKAP10 | turquoise |
| AKAP11 | blue |
| AKAP12 | black |
| AKAP13 | black |
| AKAP2 | turquoise |
| AKAP5 | turquoise |
| AKAP7 | turquoise |
| AKAP9 | yellow |
| AKD1 | pink |
| AKIRIN1 | greenyellow |
| AKIRIN2 | turquoise |
| AKNA | turquoise |
| AKR1A1 | blue |
| AKR1B1 | brown |
| AKR1B15 | darkgreen |
| AKR1C1 | blue |
| AKR1C2 | tan |
| AKR1C3 | blue |
| AKR1E2 | turquoise |
| AKR7A2 | yellow |
| AKR7A3 | tan |
| AKT1 | turquoise |
| AKT1S1 | yellow |
| AKT2 | yellow |
| AKT3 | turquoise |
| AKTIP | brown |
| AL009179.1 | yellow |
| AL021579.2 | greenyellow |
| AL021807.1 | blue |
| AL023553.1 | yellow |
| AL031009.1 | yellow |
| AL031297.1 | turquoise |
| AL031587.1 | brown |
| AL031666.1 | blue |
| AL031670.1 | greenyellow |
| AL031714.1 | yellow |
| AL031777.1 | blue |
| AL031781.1 | blue |
| AL033523.1 | darkgreen |
| AL033532.1 | yellow |
| AL034548.1 | turquoise |
| AL034548.2 | yellow |
| AL035400.1 | turquoise |
| AL035456.1 | blue |
| AL035661.1 | brown |
| AL049651.1 | black |
| AL049776.2 | turquoise |
| AL049829.2 | blue |
| AL050318.1 | magenta |
| AL050329.1 | pink |
| AL079295.1 | turquoise |
| AL096678.1 | blue |
| AL109761.5 | purple |
| AL109823.1 | royalblue |
| AL109917.1 | turquoise |
| AL109936.1 | blue |
| AL110502.1 | yellow |
| AL117209.2 | yellow |
| AL117328.1 | red |
| AL117340.2 | black |
| AL117340.3 | turquoise |
| AL117352.1 | yellow |
| AL121574.1 | yellow |
| AL121893.1 | brown |
| AL121896.1 | darkgrey |
| AL121928.1 | yellow |
| AL121929.1 | lightyellow |
| AL121963.1 | lightcyan |
| AL132709.5 | black |
| AL132768.1 | yellow |
| AL133216.3 | turquoise |
| AL133245.1 | pink |
| AL133330.1 | turquoise |
| AL133458.1 | turquoise |
| AL133509.1 | greenyellow |
| AL135752.1 | turquoise |
| AL135927.2 | lightgreen |
| AL135999.1 | turquoise |
| AL136115.1 | turquoise |
| AL136129.1 | purple |
| AL136180.1 | darkgrey |
| AL136219.2 | darkgrey |
| AL136293.2 | greenyellow |
| AL136303.2 | royalblue |
| AL136304.1 | greenyellow |
| AL136304.2 | magenta |
| AL136324.1 | turquoise |
| AL136419.1 | yellow |
| AL136527.1 | blue |
| AL136981.3 | black |
| AL137003.1 | blue |
| AL137059.3 | turquoise |
| AL137120.1 | turquoise |
| AL137247.2 | turquoise |
| AL138781.1 | blue |
| AL138831.1 | pink |
| AL138955.2 | brown |
| AL139023.1 | turquoise |
| AL139035.1 | turquoise |
| AL139041.1 | blue |
| AL139044.2 | magenta |
| AL139089.1 | black |
| AL139092.1 | pink |
| AL139099.2 | brown |
| AL139244.1 | green |
| AL139300.2 | black |
| AL139807.1 | blue |
| AL157392.1 | yellow |
| AL157687.1 | turquoise |
| AL157687.2 | turquoise |
| AL157877.1 | blue |
| AL158014.1 | greenyellow |
| AL158139.1 | yellow |
| AL158139.2 | turquoise |
| AL158167.1 | yellow |
| AL158801.1 | turquoise |
| AL158824.3 | blue |
| AL158824.4 | blue |
| AL158827.1 | turquoise |
| AL159974.2 | turquoise |
| AL160060.1 | yellow |
| AL160271.2 | turquoise |
| AL160281.1 | blue |
| AL161626.1 | red |
| AL161932.2 | greenyellow |
| AL162151.1 | red |
| AL162742.2 | blue |
| AL163011.2 | red |
| AL353600.1 | blue |
| AL353716.1 | magenta |
| AL353768.1 | blue |
| AL353805.1 | darkgrey |
| AL354696.5 | red |
| AL354696.6 | red |
| AL354751.3 | turquoise |
| AL355488.1 | pink |
| AL355574.1 | blue |
| AL355679.1 | greenyellow |
| AL355796.1 | red |
| AL355861.1 | turquoise |
| AL355922.2 | brown |
| AL356017.1 | brown |
| AL356017.3 | yellow |
| AL356057.1 | red |
| AL356107.2 | blue |
| AL356356.3 | blue |
| AL356356.4 | yellow |
| AL356356.5 | yellow |
| AL356390.1 | brown |
| AL356423.1 | turquoise |
| AL356475.1 | blue |
| AL356512.1 | turquoise |
| AL356580.1 | turquoise |
| AL356793.1 | tan |
| AL356970.1 | darkgrey |
| AL358073.1 | turquoise |
| AL358334.1 | blue |
| AL358512.1 | yellow |
| AL358781.1 | yellow |
| AL358813.4 | turquoise |
| AL358933.2 | blue |
| AL359736.1 | yellow |
| AL359752.1 | yellow |
| AL360169.1 | pink |
| AL360169.2 | turquoise |
| AL360269.1 | yellow |
| AL365277.2 | lightcyan |
| AL365502.1 | yellow |
| AL390242.1 | turquoise |
| AL390994.1 | yellow |
| AL391001.2 | brown |
| AL391152.1 | red |
| AL391417.2 | greenyellow |
| AL391421.1 | blue |
| AL442003.2 | yellow |
| AL442003.3 | yellow |
| AL442125.2 | magenta |
| AL442663.2 | greenyellow |
| AL445163.1 | greenyellow |
| AL445197.2 | blue |
| AL445486.1 | yellow |
| AL449305.2 | brown |
| AL450226.1 | yellow |
| AL450342.2 | blue |
| AL450992.2 | turquoise |
| AL512288.1 | turquoise |
| AL512356.1 | blue |
| AL512631.1 | royalblue |
| AL512658.2 | yellow |
| AL513007.1 | red |
| AL513122.1 | blue |
| AL513423.1 | darkgrey |
| AL513497.1 | salmon |
| AL583834.1 | royalblue |
| AL583842.3 | blue |
| AL583842.4 | blue |
| AL583842.5 | blue |
| AL589182.4 | yellow |
| AL589743.1 | yellow |
| AL589743.2 | yellow |
| AL589743.3 | yellow |
| AL589863.1 | brown |
| AL590666.2 | blue |
| AL590762.11 | yellow |
| AL590785.1 | yellow |
| AL590822.1 | blue |
| AL591479.1 | purple |
| AL591845.1 | yellow |
| AL591866.1 | yellow |
| AL592103.1 | purple |
| AL592284.1 | turquoise |
| AL592284.2 | yellow |
| AL592284.3 | greenyellow |
| AL592295.2 | blue |
| AL592307.2 | red |
| AL592492.1 | blue |
| AL593851.1 | salmon |
| AL596114.1 | greenyellow |
| AL603910.1 | greenyellow |
| AL606830.1 | red |
| AL627107.2 | pink |
| AL627309.2 | yellow |
| AL627402.1 | greenyellow |
| AL645811.1 | greenyellow |
| AL645941.1 | turquoise |
| AL646019.1 | turquoise |
| AL662797.1 | turquoise |
| AL663074.1 | yellow |
| AL669831.1 | yellow |
| AL669918.1 | green |
| AL671309.1 | black |
| AL671972.2 | blue |
| AL672187.1 | blue |
| AL713867.1 | turquoise |
| AL731556.1 | brown |
| AL731563.2 | lightcyan |
| AL731576.1 | lightcyan |
| AL773572.7 | turquoise |
| AL773603.1 | blue |
| AL844908.5 | turquoise |
| AL928768.3 | turquoise |
| AL929101.1 | turquoise |
| ALAD | yellow |
| ALAS1 | turquoise |
| ALCAM | tan |
| ALDH16A1 | turquoise |
| ALDH18A1 | blue |
| ALDH1A1 | turquoise |
| ALDH1A3 | blue |
| ALDH1B1 | lightcyan |
| ALDH1L1 | blue |
| ALDH1L2 | lightcyan |
| ALDH2 | turquoise |
| ALDH3A2 | brown |
| ALDH3B1 | brown |
| ALDH3B2 | tan |
| ALDH4A1 | turquoise |
| ALDH5A1 | magenta |
| ALDH6A1 | blue |
| ALDH7A1 | brown |
| ALDH9A1 | purple |
| ALDOA | turquoise |
| ALDOC | blue |
| ALG1 | blue |
| ALG10 | blue |
| ALG10B | blue |
| ALG12 | yellow |
| ALG13 | turquoise |
| ALG14 | red |
| ALG3 | blue |
| ALG5 | brown |
| ALG6 | red |
| ALG8 | turquoise |
| ALKBH2 | salmon |
| ALKBH3 | red |
| ALKBH4 | blue |
| ALKBH6 | yellow |
| ALKBH7 | turquoise |
| ALMS1 | pink |
| ALOX15B | tan |
| ALOX5 | brown |
| ALOX5AP | brown |
| ALPK1 | turquoise |
| ALPK2 | brown |
| ALPK3 | purple |
| ALPL | black |
| ALS2 | pink |
| ALS2CL | blue |
| ALS2CR4 | pink |
| ALS2CR8 | blue |
| ALX3 | yellow |
| AMACR | tan |
| AMD1 | red |
| AMDHD2 | brown |
| AMFR | blue |
| AMH | yellow |
| AMICA1 | turquoise |
| AMIGO1 | pink |
| AMIGO2 | blue |
| AMMECR1 | red |
| AMN1 | turquoise |
| AMOT | red |
| AMOTL1 | blue |
| AMOTL2 | blue |
| AMPD2 | brown |
| AMPD3 | brown |
| AMT | lightyellow |
| AMY2B | turquoise |
| AMZ2 | grey60 |
| ANAPC11 | grey60 |
| ANAPC13 | red |
| ANAPC2 | turquoise |
| ANG | blue |
| ANGEL1 | turquoise |
| ANGPT2 | blue |
| ANGPTL1 | black |
| ANGPTL2 | purple |
| ANGPTL3 | brown |
| ANGPTL4 | blue |
| ANGPTL6 | turquoise |
| ANK1 | turquoise |
| ANK2 | black |
| ANK3 | red |
| ANKAR | turquoise |
| ANKDD1A | turquoise |
| ANKH | purple |
| ANKIB1 | yellow |
| ANKLE2 | yellow |
| ANKMY1 | yellow |
| ANKMY2 | blue |
| ANKRA2 | turquoise |
| ANKRD10 | blue |
| ANKRD11 | yellow |
| ANKRD12 | turquoise |
| ANKRD13B | magenta |
| ANKRD13D | yellow |
| ANKRD16 | blue |
| ANKRD17 | red |
| ANKRD18A | brown |
| ANKRD22 | turquoise |
| ANKRD23 | yellow |
| ANKRD26 | yellow |
| ANKRD27 | brown |
| ANKRD28 | blue |
| ANKRD29 | black |
| ANKRD32 | turquoise |
| ANKRD33B | turquoise |
| ANKRD35 | blue |
| ANKRD36B | red |
| ANKRD36BL1 | lightgreen |
| ANKRD37 | turquoise |
| ANKRD39 | blue |
| ANKRD40 | grey60 |
| ANKRD42 | blue |
| ANKRD44 | turquoise |
| ANKRD46 | midnightblue |
| ANKRD5 | blue |
| ANKRD50 | blue |
| ANKRD52 | yellow |
| ANKRD54 | turquoise |
| ANKRD56 | turquoise |
| ANKRD57 | turquoise |
| ANKRD58 | turquoise |
| ANKRD6 | blue |
| ANKRD9 | turquoise |
| ANKS1A | magenta |
| ANKS1B | brown |
| ANKS6 | yellow |
| ANKZF1 | yellow |
| ANLN | green |
| ANO1 | blue |
| ANO10 | turquoise |
| ANO6 | blue |
| ANO8 | yellow |
| ANO9 | turquoise |
| ANP32A | lightgreen |
| ANP32B | yellow |
| ANP32E | blue |
| ANPEP | brown |
| ANTXR1 | purple |
| ANTXR2 | brown |
| ANUBL1 | turquoise |
| ANXA1 | blue |
| ANXA11 | blue |
| ANXA2 | turquoise |
| ANXA3 | turquoise |
| ANXA4 | turquoise |
| ANXA5 | blue |
| ANXA6 | turquoise |
| ANXA8L2 | brown |
| AOAH | turquoise |
| AOC3 | black |
| AOX1 | black |
| AP000269.1 | blue |
| AP000271.1 | blue |
| AP000295.7 | turquoise |
| AP000297.1 | turquoise |
| AP000304.2 | turquoise |
| AP000330.8 | blue |
| AP000337.1 | pink |
| AP000338.2 | yellow |
| AP000339.4 | turquoise |
| AP000347.1 | blue |
| AP000347.2 | yellow |
| AP000347.4 | yellow |
| AP000350.4 | turquoise |
| AP000350.6 | yellow |
| AP000356.6 | tan |
| AP000471.1 | yellow |
| AP000487.1 | yellow |
| AP000525.1 | yellow |
| AP000525.10 | yellow |
| AP000525.8 | yellow |
| AP000555.2 | turquoise |
| AP000560.1 | brown |
| AP000580.1 | turquoise |
| AP000654.2 | purple |
| AP000688.29 | tan |
| AP000688.8 | blue |
| AP000689.8 | tan |
| AP000692.9 | turquoise |
| AP000695.4 | lightcyan |
| AP000695.6 | purple |
| AP000699.2 | turquoise |
| AP000767.2 | black |
| AP000873.1 | yellow |
| AP000874.1 | cyan |
| AP000926.1 | blue |
| AP000936.1 | turquoise |
| AP000936.3 | turquoise |
| AP000944.1 | lightyellow |
| AP000998.1 | blue |
| AP001007.3 | brown |
| AP001011.3 | turquoise |
| AP001046.5 | turquoise |
| AP001053.11 | magenta |
| AP001055.6 | brown |
| AP001056.1 | turquoise |
| AP001062.7 | yellow |
| AP001065.2 | brown |
| AP001107.3 | greenyellow |
| AP001157.2 | brown |
| AP001178.2 | yellow |
| AP001258.1 | turquoise |
| AP001266.1 | yellow |
| AP001267.2 | turquoise |
| AP001318.4 | red |
| AP001347.1 | red |
| AP001372.2 | brown |
| AP001432.14 | turquoise |
| AP001441.1 | turquoise |
| AP001442.2 | turquoise |
| AP001468.1 | brown |
| AP001469.9 | pink |
| AP001496.1 | greenyellow |
| AP001610.5 | turquoise |
| AP001619.2 | turquoise |
| AP001767.1 | brown |
| AP001885.1 | greenyellow |
| AP001992.1 | turquoise |
| AP002381.2 | turquoise |
| AP002478.2 | turquoise |
| AP002478.4 | yellow |
| AP002490.2 | yellow |
| AP002495.1 | pink |
| AP002748.1 | yellow |
| AP002841.2 | blue |
| AP002884.2 | blue |
| AP003035.1 | red |
| AP003065.3 | turquoise |
| AP003068.4 | red |
| AP003108.1 | turquoise |
| AP003357.1 | midnightblue |
| AP003385.1 | yellow |
| AP003476.1 | turquoise |
| AP003781.2 | turquoise |
| AP004289.1 | yellow |
| AP005717.1 | royalblue |
| AP006216.11 | red |
| AP006216.12 | turquoise |
| AP006222.2 | brown |
| AP006284.3 | turquoise |
| AP006294.1 | blue |
| AP006621.1 | yellow |
| AP1AR | turquoise |
| AP1B1 | yellow |
| AP1G2 | turquoise |
| AP1M2 | turquoise |
| AP1S1 | blue |
| AP1S2 | turquoise |
| AP1S3 | blue |
| AP2A2 | brown |
| AP2B1 | yellow |
| AP2M1 | turquoise |
| AP2S1 | turquoise |
| AP3D1 | yellow |
| AP3M2 | green |
| AP3S2 | blue |
| AP4B1 | turquoise |
| AP4M1 | brown |
| AP4S1 | turquoise |
| APBA1 | brown |
| APBA2 | yellow |
| APBA3 | turquoise |
| APBB1 | turquoise |
| APBB1IP | turquoise |
| APBB2 | blue |
| APBB3 | turquoise |
| APC | turquoise |
| APCDD1 | blue |
| APCDD1L | yellow |
| APEX1 | green |
| APEX2 | blue |
| APH1A | lightgreen |
| APH1B | black |
| API5 | red |
| APIP | red |
| APITD1 | blue |
| APLF | pink |
| APLN | lightcyan |
| APLNR | turquoise |
| APLP2 | turquoise |
| APOA1BP | lightgreen |
| APOBEC2 | turquoise |
| APOBEC3A | brown |
| APOBEC3B | blue |
| APOBEC3C | turquoise |
| APOBEC3D | turquoise |
| APOBEC3F | turquoise |
| APOBEC3G | turquoise |
| APOC1 | brown |
| APOC2 | brown |
| APOD | darkgreen |
| APOE | brown |
| APOL1 | turquoise |
| APOL2 | turquoise |
| APOL3 | turquoise |
| APOL4 | turquoise |
| APOL6 | turquoise |
| APOLD1 | black |
| APOM | magenta |
| APOO | blue |
| APOOL | red |
| APP | turquoise |
| APPBP2 | grey60 |
| APPL1 | pink |
| APPL2 | blue |
| APRT | blue |
| APTX | brown |
| AQP1 | black |
| AQP3 | tan |
| AQP5 | brown |
| AQP7P3 | black |
| AQP9 | brown |
| AR | tan |
| ARAF | yellow |
| ARAP1 | turquoise |
| ARAP2 | turquoise |
| ARAP3 | black |
| ARCN1 | red |
| ARF1 | green |
| ARF4 | purple |
| ARF5 | turquoise |
| ARF6 | green |
| ARFGAP1 | yellow |
| ARFGAP3 | blue |
| ARFGEF1 | midnightblue |
| ARFGEF2 | midnightblue |
| ARFIP2 | tan |
| ARGLU1 | turquoise |
| ARHGAP1 | lightyellow |
| ARHGAP10 | blue |
| ARHGAP11A | green |
| ARHGAP11B | green |
| ARHGAP12 | brown |
| ARHGAP15 | turquoise |
| ARHGAP17 | yellow |
| ARHGAP18 | brown |
| ARHGAP19 | turquoise |
| ARHGAP21 | turquoise |
| ARHGAP22 | brown |
| ARHGAP23 | lightyellow |
| ARHGAP24 | turquoise |
| ARHGAP25 | turquoise |
| ARHGAP26 | turquoise |
| ARHGAP28 | blue |
| ARHGAP29 | blue |
| ARHGAP30 | turquoise |
| ARHGAP31 | black |
| ARHGAP32 | turquoise |
| ARHGAP33 | yellow |
| ARHGAP39 | darkred |
| ARHGAP4 | turquoise |
| ARHGAP42 | blue |
| ARHGAP44 | yellow |
| ARHGAP5 | brown |
| ARHGAP6 | black |
| ARHGAP8 | turquoise |
| ARHGAP9 | turquoise |
| ARHGDIA | yellow |
| ARHGDIB | turquoise |
| ARHGEF1 | turquoise |
| ARHGEF10 | yellow |
| ARHGEF10L | yellow |
| ARHGEF11 | yellow |
| ARHGEF12 | red |
| ARHGEF15 | black |
| ARHGEF16 | turquoise |
| ARHGEF17 | lightyellow |
| ARHGEF19 | brown |
| ARHGEF2 | magenta |
| ARHGEF25 | blue |
| ARHGEF26 | brown |
| ARHGEF3 | turquoise |
| ARHGEF35 | brown |
| ARHGEF37 | brown |
| ARHGEF38 | tan |
| ARHGEF4 | yellow |
| ARHGEF40 | blue |
| ARHGEF5 | brown |
| ARHGEF6 | turquoise |
| ARHGEF7 | blue |
| ARHGEF9 | brown |
| ARID1A | yellow |
| ARID1B | pink |
| ARID2 | yellow |
| ARID3A | turquoise |
| ARID3B | turquoise |
| ARID4A | turquoise |
| ARID4B | red |
| ARID5A | turquoise |
| ARID5B | turquoise |
| ARL11 | turquoise |
| ARL13B | blue |
| ARL15 | black |
| ARL16 | grey60 |
| ARL17A | turquoise |
| ARL17B | turquoise |
| ARL2 | turquoise |
| ARL3 | blue |
| ARL4A | blue |
| ARL4C | brown |
| ARL5B | green |
| ARL6 | turquoise |
| ARL6IP1 | green |
| ARL6IP5 | brown |
| ARL6IP6 | green |
| ARL8A | magenta |
| ARL8B | blue |
| ARL9 | blue |
| ARMC1 | midnightblue |
| ARMC10 | brown |
| ARMC2 | blue |
| ARMC6 | blue |
| ARMC8 | blue |
| ARMC9 | blue |
| ARMCX1 | blue |
| ARMCX2 | blue |
| ARMCX3 | blue |
| ARMCX4 | blue |
| ARMCX5 | blue |
| ARMCX6 | turquoise |
| ARNTL | turquoise |
| ARNTL2 | cyan |
| ARPC1A | brown |
| ARPC1B | brown |
| ARPC4 | brown |
| ARPC5 | brown |
| ARPC5L | blue |
| ARRB1 | brown |
| ARRB2 | brown |
| ARRDC1 | tan |
| ARRDC2 | turquoise |
| ARRDC3 | blue |
| ARRDC4 | blue |
| ARSA | turquoise |
| ARSB | brown |
| ARSD | brown |
| ARSE | turquoise |
| ARSG | turquoise |
| ARSI | purple |
| ARSJ | blue |
| ART3 | blue |
| ARTN | turquoise |
| ARV1 | green |
| ARVCF | brown |
| AS3MT | brown |
| ASAH1 | brown |
| ASAP1 | blue |
| ASAP2 | greenyellow |
| ASAP3 | turquoise |
| ASB1 | blue |
| ASB13 | brown |
| ASB2 | turquoise |
| ASB9 | cyan |
| ASB9P1 | yellow |
| ASCC1 | yellow |
| ASCC2 | yellow |
| ASCC3 | red |
| ASCL2 | brown |
| ASF1A | red |
| ASF1B | green |
| ASH2L | yellow |
| ASL | turquoise |
| ASMTL | turquoise |
| ASNA1 | blue |
| ASNS | blue |
| ASPH | blue |
| ASPHD1 | blue |
| ASPM | green |
| ASPN | purple |
| ASPSCR1 | grey60 |
| ASRGL1 | blue |
| ASS1 | blue |
| ASTN2 | yellow |
| ATAD1 | red |
| ATAD2 | green |
| ATAD3A | blue |
| ATAD3B | blue |
| ATAD3C | yellow |
| ATAD5 | green |
| ATBF1 | lightyellow |
| ATF3 | blue |
| ATF5 | yellow |
| ATF7 | yellow |
| ATF7IP | turquoise |
| ATF7IP2 | turquoise |
| ATG10 | brown |
| ATG12P2 | turquoise |
| ATG16L1 | yellow |
| ATG16L2 | turquoise |
| ATG4C | turquoise |
| ATG4D | blue |
| ATG5 | red |
| ATG7 | brown |
| ATG9B | turquoise |
| ATHL1 | turquoise |
| ATIC | yellow |
| ATL1 | brown |
| ATL2 | brown |
| ATL3 | blue |
| ATM | turquoise |
| ATN1 | yellow |
| ATOX1 | brown |
| ATP10A | blue |
| ATP10B | blue |
| ATP10D | turquoise |
| ATP11A | yellow |
| ATP11B | yellow |
| ATP11C | red |
| ATP13A1 | yellow |
| ATP13A2 | yellow |
| ATP13A3 | green |
| ATP13A4 | tan |
| ATP13A5 | brown |
| ATP1A1 | turquoise |
| ATP1A2 | black |
| ATP1B1 | brown |
| ATP1B3 | turquoise |
| ATP2A2 | yellow |
| ATP2A3 | turquoise |
| ATP2B1 | blue |
| ATP2B4 | blue |
| ATP2C1 | turquoise |
| ATP2C2 | turquoise |
| ATP5A1 | red |
| ATP5C1 | blue |
| ATP5D | yellow |
| ATP5E | yellow |
| ATP5F1 | blue |
| ATP5G1 | blue |
| ATP5G3 | blue |
| ATP5H | grey60 |
| ATP5I | yellow |
| ATP5J | blue |
| ATP5J2 | blue |
| ATP5O | blue |
| ATP6AP1 | blue |
| ATP6AP1L | blue |
| ATP6V0A1 | blue |
| ATP6V0A4 | brown |
| ATP6V0B | blue |
| ATP6V0C | greenyellow |
| ATP6V0D1 | tan |
| ATP6V0E1 | brown |
| ATP6V0E2 | brown |
| ATP6V1B1 | brown |
| ATP6V1B2 | brown |
| ATP6V1C1 | midnightblue |
| ATP6V1C2 | turquoise |
| ATP6V1E2 | red |
| ATP6V1F | blue |
| ATP6V1G1 | turquoise |
| ATP6V1G1P4 | blue |
| ATP6V1H | midnightblue |
| ATP7A | blue |
| ATP7B | turquoise |
| ATP8A1 | turquoise |
| ATP8B1 | turquoise |
| ATP8B2 | turquoise |
| ATP8B4 | brown |
| ATP9A | brown |
| ATP9B | turquoise |
| ATPAF1 | yellow |
| ATPAF2 | turquoise |
| ATPBD4 | red |
| ATPIF1 | yellow |
| ATRNL1 | brown |
| ATXN1 | black |
| ATXN10 | yellow |
| ATXN2 | yellow |
| ATXN2L | yellow |
| ATXN7 | turquoise |
| ATXN7L1 | turquoise |
| ATXN7L2 | magenta |
| ATXN7L3B | red |
| AUH | pink |
| AURKA | green |
| AURKAIP1 | blue |
| AURKAPS1 | yellow |
| AURKB | green |
| AUTS2 | yellow |
| AVIL | blue |
| AVL9 | blue |
| AVPI1 | pink |
| AXIN1 | blue |
| AXIN2 | black |
| AXL | brown |
| AZGP1 | brown |
| AZI1 | grey60 |
| AZIN1 | midnightblue |
| B2M | turquoise |
| B3GALNT1 | turquoise |
| B3GALNT2 | green |
| B3GALT4 | turquoise |
| B3GALT6 | green |
| B3GALTL | blue |
| B3GNT1 | red |
| B3GNT3 | brown |
| B3GNT5 | green |
| B3GNT7 | blue |
| B3GNT8 | blue |
| B3GNT9 | lightcyan |
| B3GNTL1 | grey60 |
| B4GALNT1 | turquoise |
| B4GALNT3 | cyan |
| B4GALNT4 | blue |
| B4GALT1 | yellow |
| B4GALT2 | green |
| B4GALT3 | lightgreen |
| B4GALT4 | turquoise |
| B4GALT5 | yellow |
| B4GALT7 | blue |
| B9D1 | blue |
| B9D2 | turquoise |
| BACE1 | lightyellow |
| BACE2 | turquoise |
| BACH1 | brown |
| BACH2 | turquoise |
| BAD | turquoise |
| BAG1 | turquoise |
| BAG2 | turquoise |
| BAG3 | greenyellow |
| BAG4 | turquoise |
| BAG5 | midnightblue |
| BAHCC1 | yellow |
| BAHD1 | yellow |
| BAI1 | blue |
| BAIAP2 | grey60 |
| BAIAP2L1 | turquoise |
| BAIAP2L2 | turquoise |
| BAIAP3 | blue |
| BAK1 | turquoise |
| BAMBI | brown |
| BANF1 | blue |
| BANK1 | turquoise |
| BARD1 | green |
| BARX2 | brown |
| BASP1 | brown |
| BAT1 | magenta |
| BAT2 | magenta |
| BAT2D1 | red |
| BAT2L | yellow |
| BAT3 | magenta |
| BAT4 | magenta |
| BATF | turquoise |
| BATF2 | turquoise |
| BATF3 | turquoise |
| BAZ1A | red |
| BAZ2A | yellow |
| BAZ2B | blue |
| BBC3 | red |
| BBOX1 | brown |
| BBS10 | blue |
| BBS2 | blue |
| BBS7 | blue |
| BBS9 | blue |
| BBX | turquoise |
| BCAM | blue |
| BCAP29 | blue |
| BCAP31 | blue |
| BCAR1 | turquoise |
| BCAR3 | blue |
| BCAS2 | red |
| BCAS3 | turquoise |
| BCAS4 | turquoise |
| BCAT1 | brown |
| BCAT2 | turquoise |
| BCKDHA | blue |
| BCKDHB | turquoise |
| BCL11A | yellow |
| BCL11B | turquoise |
| BCL2 | turquoise |
| BCL2A1 | brown |
| BCL2L1 | yellow |
| BCL2L11 | turquoise |
| BCL2L12 | green |
| BCL2L13 | midnightblue |
| BCL2L14 | cyan |
| BCL2L15 | blue |
| BCL2L2 | turquoise |
| BCL3 | yellow |
| BCL6 | blue |
| BCL6B | black |
| BCL7A | magenta |
| BCL7B | green |
| BCL7C | turquoise |
| BCL9 | yellow |
| BCL9L | yellow |
| BCO2 | blue |
| BCOR | yellow |
| BCORL1 | yellow |
| BCR | yellow |
| BCS1L | blue |
| BCYRN1 | red |
| BDH1 | brown |
| BDH2 | blue |
| BDP1 | blue |
| BEND3 | green |
| BEND6 | blue |
| BEND7 | yellow |
| BEST1 | brown |
| BET1 | blue |
| BET1L | turquoise |
| BEX2 | brown |
| BEX4 | blue |
| BEX5 | turquoise |
| BGN | purple |
| BHLHA15 | turquoise |
| BHLHB9 | brown |
| BHLHE40 | lightcyan |
| BHLHE41 | cyan |
| BHMT2 | black |
| BICC1 | blue |
| BICD1 | yellow |
| BID | darkturquoise |
| BIK | brown |
| BIN1 | turquoise |
| BIN2 | turquoise |
| BIN3 | blue |
| BIRC2 | pink |
| BIRC3 | turquoise |
| BIRC5 | green |
| BIVM | yellow |
| BLCAP | yellow |
| BLM | green |
| BLMH | turquoise |
| BLNK | turquoise |
| BLOC1S1 | brown |
| BLOC1S2 | brown |
| BLOC1S3 | yellow |
| BLVRA | brown |
| BLVRB | tan |
| BMF | turquoise |
| BMI1 | brown |
| BMP1 | purple |
| BMP2K | brown |
| BMP2KL | blue |
| BMP4 | blue |
| BMP6 | turquoise |
| BMP7 | blue |
| BMP8A | lightcyan |
| BMP8B | brown |
| BMPR1A | red |
| BMPR1B | turquoise |
| BMS1P1 | yellow |
| BNC2 | purple |
| BNIP3 | turquoise |
| BNIP3L | lightcyan |
| BNIPL | brown |
| BOC | blue |
| BOD1L | turquoise |
| BOK | turquoise |
| BOLA1 | blue |
| BOLA2B | darkgrey |
| BOLA3 | blue |
| BOP1 | darkred |
| BPGM | green |
| BPHL | magenta |
| BPI | blue |
| BPNT1 | blue |
| BPTF | grey60 |
| BRAF | yellow |
| BRCA1 | brown |
| BRCA2 | green |
| BRCC3 | blue |
| BRD1 | yellow |
| BRD3 | yellow |
| BRD4 | yellow |
| BRD7P3 | turquoise |
| BRD8 | yellow |
| BRD9 | yellow |
| BRE | blue |
| BRF1 | yellow |
| BRF2 | blue |
| BRI3 | brown |
| BRI3BP | blue |
| BRIP1 | green |
| BRIX1 | brown |
| BRMS1 | blue |
| BRP44 | turquoise |
| BRP44L | yellow |
| BRPF1 | yellow |
| BRPF3 | magenta |
| BRSK1 | yellow |
| BRWD1 | pink |
| BRWD3 | turquoise |
| BSCL2 | turquoise |
| BSDC1 | yellow |
| BSG | turquoise |
| BSPRY | brown |
| BST1 | brown |
| BST2 | turquoise |
| BTBD11 | blue |
| BTBD19 | purple |
| BTBD2 | yellow |
| BTBD3 | turquoise |
| BTBD6 | turquoise |
| BTBD8 | pink |
| BTBD9 | blue |
| BTC | brown |
| BTD | blue |
| BTF3 | salmon |
| BTF3L4 | green |
| BTG1 | turquoise |
| BTG2 | turquoise |
| BTG3 | green |
| BTK | turquoise |
| BTN2A1 | magenta |
| BTN2A2 | turquoise |
| BTN3A1 | turquoise |
| BTN3A2 | turquoise |
| BTN3A3 | turquoise |
| BTNL9 | black |
| BUB1 | green |
| BUB1B | green |
| BUB3 | blue |
| BUD31 | turquoise |
| BX004987.4 | turquoise |
| BX004987.5 | yellow |
| BX248398.1 | turquoise |
| BX276089.1 | pink |
| BX284650.1 | turquoise |
| BX284650.2 | turquoise |
| BX284650.3 | yellow |
| BX284650.4 | turquoise |
| BX322557.10 | turquoise |
| BX470102.3 | brown |
| BX571672.1 | brown |
| BX571672.2 | yellow |
| BX571672.4 | yellow |
| BX571672.5 | blue |
| BX664615.1 | blue |
| BYSL | magenta |
| BZRAP1 | yellow |
| BZW1 | red |
| BZW2 | turquoise |
| C10orf10 | turquoise |
| C10orf108 | yellow |
| C10orf11 | brown |
| C10orf113 | turquoise |
| C10orf114 | turquoise |
| C10orf116 | black |
| C10orf125 | turquoise |
| C10orf128 | turquoise |
| C10orf131 | black |
| C10orf18 | green |
| C10orf2 | blue |
| C10orf26 | black |
| C10orf31 | turquoise |
| C10orf32 | blue |
| C10orf35 | lightgreen |
| C10orf4 | blue |
| C10orf47 | turquoise |
| C10orf54 | turquoise |
| C10orf55 | lightcyan |
| C10orf57 | greenyellow |
| C10orf72 | blue |
| C10orf75 | turquoise |
| C10orf81 | brown |
| C10orf90 | brown |
| C10orf95 | blue |
| C11orf1 | red |
| C11orf10 | yellow |
| C11orf17 | brown |
| C11orf2 | salmon |
| C11orf24 | yellow |
| C11orf30 | yellow |
| C11orf31 | blue |
| C11orf35 | brown |
| C11orf41 | cyan |
| C11orf46 | red |
| C11orf48 | blue |
| C11orf49 | brown |
| C11orf51 | turquoise |
| C11orf52 | brown |
| C11orf54 | pink |
| C11orf58 | red |
| C11orf61 | blue |
| C11orf63 | blue |
| C11orf67 | brown |
| C11orf71 | blue |
| C11orf73 | yellow |
| C11orf74 | red |
| C11orf75 | yellow |
| C11orf80 | magenta |
| C11orf82 | green |
| C11orf83 | turquoise |
| C11orf84 | blue |
| C11orf89 | turquoise |
| C11orf95 | brown |
| C11orf96 | blue |
| C12orf11 | cyan |
| C12orf23 | purple |
| C12orf24 | salmon |
| C12orf26 | turquoise |
| C12orf29 | turquoise |
| C12orf32 | cyan |
| C12orf34 | turquoise |
| C12orf35 | turquoise |
| C12orf4 | cyan |
| C12orf44 | turquoise |
| C12orf45 | blue |
| C12orf48 | green |
| C12orf49 | red |
| C12orf5 | brown |
| C12orf51 | yellow |
| C12orf52 | turquoise |
| C12orf57 | salmon |
| C12orf61 | turquoise |
| C12orf62 | greenyellow |
| C12orf66 | turquoise |
| C12orf75 | brown |
| C13orf15 | turquoise |
| C13orf18 | turquoise |
| C13orf23 | yellow |
| C13orf27 | green |
| C13orf31 | brown |
| C13orf33 | black |
| C13orf34 | green |
| C13orf37 | blue |
| C13orf38 | brown |
| C14orf1 | yellow |
| C14orf104 | blue |
| C14orf106 | turquoise |
| C14orf109 | yellow |
| C14orf126 | red |
| C14orf129 | greenyellow |
| C14orf138 | red |
| C14orf139 | turquoise |
| C14orf142 | blue |
| C14orf145 | turquoise |
| C14orf147 | turquoise |
| C14orf149 | turquoise |
| C14orf156 | yellow |
| C14orf159 | turquoise |
| C14orf167 | brown |
| C14orf179 | yellow |
| C14orf181 | turquoise |
| C14orf182 | turquoise |
| C14orf2 | yellow |
| C14orf37 | blue |
| C14orf4 | yellow |
| C14orf43 | blue |
| C14orf45 | blue |
| C14orf49 | turquoise |
| C14orf79 | brown |
| C14orf80 | blue |
| C15orf23 | green |
| C15orf29 | red |
| C15orf38 | blue |
| C15orf39 | yellow |
| C15orf40 | yellow |
| C15orf42 | green |
| C15orf48 | brown |
| C15orf52 | magenta |
| C15orf57 | turquoise |
| C15orf58 | yellow |
| C15orf61 | turquoise |
| C15orf62 | yellow |
| C16orf28 | turquoise |
| C16orf3 | brown |
| C16orf35 | yellow |
| C16orf45 | blue |
| C16orf48 | brown |
| C16orf5 | turquoise |
| C16orf53 | blue |
| C16orf54 | turquoise |
| C16orf55 | magenta |
| C16orf57 | yellow |
| C16orf58 | brown |
| C16orf59 | green |
| C16orf61 | blue |
| C16orf62 | blue |
| C16orf63 | red |
| C16orf67 | turquoise |
| C16orf7 | salmon |
| C16orf75 | green |
| C16orf79 | yellow |
| C16orf80 | turquoise |
| C16orf87 | green |
| C16orf88 | brown |
| C16orf91 | blue |
| C16orf93 | brown |
| C17orf101 | grey60 |
| C17orf103 | lightyellow |
| C17orf106 | grey60 |
| C17orf107 | brown |
| C17orf108 | blue |
| C17orf28 | yellow |
| C17orf37 | blue |
| C17orf44 | turquoise |
| C17orf48 | turquoise |
| C17orf49 | turquoise |
| C17orf51 | blue |
| C17orf53 | green |
| C17orf56 | turquoise |
| C17orf57 | blue |
| C17orf58 | grey60 |
| C17orf61 | greenyellow |
| C17orf62 | turquoise |
| C17orf63 | yellow |
| C17orf65 | yellow |
| C17orf68 | turquoise |
| C17orf70 | grey60 |
| C17orf75 | green |
| C17orf76 | salmon |
| C17orf79 | darkturquoise |
| C17orf84 | blue |
| C17orf86 | grey60 |
| C17orf87 | turquoise |
| C17orf89 | grey60 |
| C17orf90 | grey60 |
| C17orf91 | black |
| C17orf95 | grey60 |
| C17orf96 | blue |
| C17orf97 | turquoise |
| C18orf1 | pink |
| C18orf10 | red |
| C18orf19 | green |
| C18orf21 | brown |
| C18orf22 | brown |
| C18orf25 | red |
| C18orf32 | red |
| C18orf45 | brown |
| C18orf54 | red |
| C18orf55 | red |
| C18orf56 | green |
| C19orf10 | blue |
| C19orf12 | blue |
| C19orf18 | darkgreen |
| C19orf2 | blue |
| C19orf20 | yellow |
| C19orf21 | turquoise |
| C19orf22 | yellow |
| C19orf23 | turquoise |
| C19orf24 | yellow |
| C19orf28 | blue |
| C19orf29OS | green |
| C19orf33 | brown |
| C19orf36 | turquoise |
| C19orf38 | brown |
| C19orf40 | green |
| C19orf43 | blue |
| C19orf46 | brown |
| C19orf47 | brown |
| C19orf48 | blue |
| C19orf52 | blue |
| C19orf53 | blue |
| C19orf54 | yellow |
| C19orf55 | yellow |
| C19orf56 | turquoise |
| C19orf57 | brown |
| C19orf60 | greenyellow |
| C19orf63 | turquoise |
| C19orf66 | turquoise |
| C19orf70 | yellow |
| C19orf71 | blue |
| C19orf73 | brown |
| C1GALT1 | turquoise |
| C1GALT1C1 | blue |
| C1orf103 | red |
| C1orf104 | lightgreen |
| C1orf106 | blue |
| C1orf107 | green |
| C1orf109 | green |
| C1orf112 | green |
| C1orf113 | yellow |
| C1orf115 | blue |
| C1orf116 | brown |
| C1orf122 | turquoise |
| C1orf126 | yellow |
| C1orf132 | turquoise |
| C1orf133 | turquoise |
| C1orf135 | green |
| C1orf138 | lightyellow |
| C1orf144 | yellow |
| C1orf145 | yellow |
| C1orf147 | turquoise |
| C1orf151 | yellow |
| C1orf159 | brown |
| C1orf161 | darkgrey |
| C1orf162 | brown |
| C1orf163 | green |
| C1orf170 | greenyellow |
| C1orf172 | brown |
| C1orf174 | midnightblue |
| C1orf183 | blue |
| C1orf186 | turquoise |
| C1orf190 | blue |
| C1orf192 | brown |
| C1orf195 | brown |
| C1orf198 | turquoise |
| C1orf201 | brown |
| C1orf203 | pink |
| C1orf204 | turquoise |
| C1orf21 | turquoise |
| C1orf213 | blue |
| C1orf216 | blue |
| C1orf228 | turquoise |
| C1orf25 | red |
| C1orf31 | green |
| C1orf35 | green |
| C1orf38 | brown |
| C1orf43 | lightgreen |
| C1orf50 | green |
| C1orf51 | brown |
| C1orf52 | pink |
| C1orf53 | turquoise |
| C1orf54 | brown |
| C1orf57 | green |
| C1orf59 | blue |
| C1orf61 | blue |
| C1orf63 | turquoise |
| C1orf66 | lightgreen |
| C1orf69 | yellow |
| C1orf74 | turquoise |
| C1orf84 | yellow |
| C1orf85 | lightgreen |
| C1orf86 | yellow |
| C1orf89 | blue |
| C1orf9 | green |
| C1orf93 | blue |
| C1orf96 | green |
| C1orf97 | green |
| C1orf98 | tan |
| C1QA | turquoise |
| C1QB | brown |
| C1QBP | blue |
| C1QC | brown |
| C1QL3 | turquoise |
| C1QTNF1 | blue |
| C1QTNF3 | lightcyan |
| C1QTNF5 | purple |
| C1QTNF6 | purple |
| C1QTNF7 | blue |
| C1R | turquoise |
| C1RL | turquoise |
| C1S | turquoise |
| C2 | brown |
| C20orf100 | turquoise |
| C20orf103 | blue |
| C20orf108 | brown |
| C20orf11 | brown |
| C20orf111 | brown |
| C20orf112 | yellow |
| C20orf117 | yellow |
| C20orf118 | turquoise |
| C20orf12 | brown |
| C20orf134 | yellow |
| C20orf135 | turquoise |
| C20orf151 | brown |
| C20orf165 | yellow |
| C20orf177 | yellow |
| C20orf194 | black |
| C20orf196 | turquoise |
| C20orf199 | salmon |
| C20orf20 | blue |
| C20orf24 | blue |
| C20orf27 | blue |
| C20orf3 | turquoise |
| C20orf46 | blue |
| C20orf54 | brown |
| C20orf7 | brown |
| C20orf82 | blue |
| C20orf94 | yellow |
| C20orf95 | darkturquoise |
| C20orf96 | yellow |
| C21orf125 | turquoise |
| C21orf2 | turquoise |
| C21orf33 | blue |
| C21orf34 | blue |
| C21orf45 | green |
| C21orf49 | blue |
| C21orf51 | yellow |
| C21orf56 | blue |
| C21orf57 | yellow |
| C21orf58 | green |
| C21orf59 | brown |
| C21orf63 | turquoise |
| C21orf67 | turquoise |
| C21orf7 | black |
| C21orf70 | blue |
| C21orf82 | pink |
| C21orf84 | turquoise |
| C21orf91 | blue |
| C22orf13 | tan |
| C22orf23 | yellow |
| C22orf25 | brown |
| C22orf26 | yellow |
| C22orf27 | yellow |
| C22orf29 | brown |
| C22orf30 | yellow |
| C22orf32 | turquoise |
| C22orf34 | turquoise |
| C22orf36 | turquoise |
| C22orf39 | yellow |
| C22orf40 | darkred |
| C22orf46 | blue |
| C22orf9 | brown |
| C2CD2 | brown |
| C2CD2L | yellow |
| C2CD3 | yellow |
| C2CD4D | blue |
| C2orf15 | red |
| C2orf18 | brown |
| C2orf27A | blue |
| C2orf29 | red |
| C2orf3 | red |
| C2orf34 | blue |
| C2orf40 | blue |
| C2orf43 | turquoise |
| C2orf47 | blue |
| C2orf54 | tan |
| C2orf55 | blue |
| C2orf58 | turquoise |
| C2orf63 | blue |
| C2orf67 | pink |
| C2orf68 | yellow |
| C2orf7 | turquoise |
| C2orf72 | turquoise |
| C2orf74 | turquoise |
| C2orf76 | turquoise |
| C2orf79 | blue |
| C2orf82 | blue |
| C2orf84 | blue |
| C2orf88 | turquoise |
| C2orf89 | turquoise |
| C3 | turquoise |
| C3AR1 | brown |
| C3orf10 | turquoise |
| C3orf14 | turquoise |
| C3orf15 | blue |
| C3orf18 | blue |
| C3orf21 | blue |
| C3orf23 | green |
| C3orf26 | blue |
| C3orf31 | yellow |
| C3orf32 | blue |
| C3orf33 | turquoise |
| C3orf35 | yellow |
| C3orf37 | green |
| C3orf39 | turquoise |
| C3orf42 | turquoise |
| C3orf52 | brown |
| C3orf54 | black |
| C3orf55 | turquoise |
| C3orf58 | darkgreen |
| C3orf59 | brown |
| C3orf62 | blue |
| C3orf64 | blue |
| C3orf67 | turquoise |
| C3orf70 | blue |
| C3orf78 | yellow |
| C4A | turquoise |
| C4B | turquoise |
| C4orf10 | turquoise |
| C4orf18 | blue |
| C4orf19 | blue |
| C4orf21 | green |
| C4orf23 | yellow |
| C4orf27 | blue |
| C4ORF3 | turquoise |
| C4orf31 | blue |
| C4orf32 | black |
| C4orf33 | brown |
| C4orf34 | blue |
| C4orf36 | blue |
| C4orf42 | yellow |
| C4orf44 | yellow |
| C4orf46 | green |
| C4orf48 | yellow |
| C4orf7 | cyan |
| C4orf8 | yellow |
| C5 | blue |
| C5AR1 | brown |
| C5orf13 | blue |
| C5orf22 | turquoise |
| C5orf23 | turquoise |
| C5orf25 | yellow |
| C5orf27 | turquoise |
| C5orf28 | turquoise |
| C5orf30 | brown |
| C5orf32 | blue |
| C5orf33 | turquoise |
| C5orf34 | green |
| C5orf35 | turquoise |
| C5orf36 | blue |
| C5orf37 | turquoise |
| C5orf38 | turquoise |
| C5orf39 | turquoise |
| C5orf4 | blue |
| C5orf41 | turquoise |
| C5orf42 | red |
| C5orf45 | blue |
| C5orf46 | turquoise |
| C5orf51 | yellow |
| C5orf53 | blue |
| C5orf54 | brown |
| C5orf56 | turquoise |
| C5orf58 | turquoise |
| C5orf62 | blue |
| C6orf1 | magenta |
| C6orf105 | turquoise |
| C6orf106 | magenta |
| C6orf108 | darkturquoise |
| C6orf114 | turquoise |
| C6orf115 | blue |
| C6orf120 | turquoise |
| C6orf123 | yellow |
| C6orf124 | brown |
| C6orf125 | blue |
| C6orf127 | brown |
| C6orf129 | blue |
| C6orf130 | magenta |
| C6orf132 | brown |
| C6orf134 | magenta |
| C6orf136 | magenta |
| C6orf141 | brown |
| C6orf142 | turquoise |
| C6orf145 | blue |
| C6orf150 | turquoise |
| C6orf153 | magenta |
| C6orf155 | cyan |
| C6orf162 | red |
| C6orf164 | black |
| C6orf167 | red |
| C6orf168 | magenta |
| C6orf170 | pink |
| C6orf174 | black |
| C6orf182 | red |
| C6orf192 | turquoise |
| C6orf203 | red |
| C6orf204 | turquoise |
| C6orf211 | blue |
| C6orf223 | darkgreen |
| C6orf225 | blue |
| C6orf226 | yellow |
| C6orf25 | yellow |
| C6orf26 | magenta |
| C6orf27 | turquoise |
| C6orf35 | pink |
| C6orf48 | magenta |
| C6orf52 | blue |
| C6orf57 | brown |
| C6orf62 | magenta |
| C6orf64 | magenta |
| C6orf70 | blue |
| C6orf72 | blue |
| C6orf81 | darkgreen |
| C6orf89 | magenta |
| C7 | black |
| C7orf10 | lightcyan |
| C7orf11 | blue |
| C7orf13 | brown |
| C7orf23 | turquoise |
| C7orf25 | turquoise |
| C7orf26 | yellow |
| C7orf27 | blue |
| C7orf28A | blue |
| C7orf28B | yellow |
| C7orf29 | turquoise |
| C7orf30 | turquoise |
| C7orf36 | brown |
| C7orf40 | blue |
| C7orf41 | black |
| C7orf43 | turquoise |
| C7orf44 | turquoise |
| C7orf46 | blue |
| C7orf47 | blue |
| C7orf50 | turquoise |
| C7orf53 | brown |
| C7orf55 | darkturquoise |
| C7orf58 | turquoise |
| C7orf59 | salmon |
| C7orf60 | blue |
| C7orf63 | blue |
| C7orf64 | yellow |
| C7orf68 | turquoise |
| C7orf70 | turquoise |
| C8G | cyan |
| C8orf33 | darkred |
| C8orf38 | midnightblue |
| C8orf39 | midnightblue |
| C8orf4 | blue |
| C8orf40 | brown |
| C8orf42 | blue |
| C8orf44 | yellow |
| C8orf45 | midnightblue |
| C8orf46 | blue |
| C8orf51 | darkred |
| C8orf55 | darkred |
| C8orf58 | lightyellow |
| C8orf59 | midnightblue |
| C8orf73 | turquoise |
| C8orf76 | midnightblue |
| C8orf79 | blue |
| C8orf82 | darkred |
| C8orf83 | blue |
| C8orf84 | blue |
| C8orf85 | brown |
| C9orf100 | green |
| C9orf102 | turquoise |
| C9orf103 | turquoise |
| C9orf114 | blue |
| C9orf116 | turquoise |
| C9orf119 | turquoise |
| C9orf123 | lightyellow |
| C9orf126 | yellow |
| C9orf130 | turquoise |
| C9orf131 | turquoise |
| C9orf133 | yellow |
| C9orf140 | blue |
| C9orf142 | blue |
| C9orf150 | brown |
| C9orf152 | tan |
| C9orf156 | turquoise |
| C9orf16 | brown |
| C9orf163 | blue |
| C9orf167 | brown |
| C9orf172 | yellow |
| C9orf21 | brown |
| C9orf23 | blue |
| C9orf25 | yellow |
| C9orf3 | blue |
| C9orf30 | green |
| C9orf31 | lightyellow |
| C9orf37 | turquoise |
| C9orf40 | green |
| C9orf41 | red |
| C9orf45 | yellow |
| C9orf46 | yellow |
| C9orf47 | blue |
| C9orf5 | red |
| C9orf6 | greenyellow |
| C9orf61 | blue |
| C9orf64 | blue |
| C9orf68 | blue |
| C9orf69 | blue |
| C9orf7 | brown |
| C9orf72 | brown |
| C9orf75 | blue |
| C9orf80 | red |
| C9orf82 | brown |
| C9orf85 | darkgrey |
| C9orf86 | yellow |
| C9orf89 | turquoise |
| C9orf9 | brown |
| C9orf91 | yellow |
| C9orf93 | brown |
| C9orf95 | turquoise |
| C9orf96 | yellow |
| CA1 | brown |
| CA11 | turquoise |
| CA13 | brown |
| CA2 | brown |
| CA3 | brown |
| CA5B | turquoise |
| CA5BP | magenta |
| CA8 | red |
| CA9 | turquoise |
| CAB39L | brown |
| CABC1 | purple |
| CABIN1 | yellow |
| CABLES1 | brown |
| CABLES2 | greenyellow |
| CABYR | brown |
| CACHD1 | blue |
| CACNA1C | blue |
| CACNA1D | yellow |
| CACNA2D1 | black |
| CACNA2D3 | blue |
| CACNB1 | yellow |
| CACNB2 | pink |
| CACNB3 | magenta |
| CACNG4 | turquoise |
| CACYBP | green |
| CAD | magenta |
| CADM1 | blue |
| CADM4 | magenta |
| CADPS | purple |
| CADPS2 | blue |
| CALB2 | turquoise |
| CALCOCO1 | black |
| CALCOCO2 | turquoise |
| CALCRL | black |
| CALD1 | blue |
| CALHM2 | black |
| CALM1 | blue |
| CALM2 | red |
| CALM3 | brown |
| CALML3 | blue |
| CALML4 | turquoise |
| CALML5 | brown |
| CALML6 | yellow |
| CALR | blue |
| CALU | turquoise |
| CAMK1 | turquoise |
| CAMK1D | brown |
| CAMK2D | turquoise |
| CAMK2G | yellow |
| CAMK2N1 | lightcyan |
| CAMK2N2 | turquoise |
| CAMK4 | turquoise |
| CAMKK1 | brown |
| CAMLG | blue |
| CAMSAP1 | greenyellow |
| CAMSAP1L1 | turquoise |
| CAMTA1 | turquoise |
| CAMTA2 | yellow |
| CAND1 | green |
| CAND2 | yellow |
| CANT1 | grey60 |
| CANX | turquoise |
| CAP2 | turquoise |
| CAPG | blue |
| CAPN1 | turquoise |
| CAPN10 | turquoise |
| CAPN12 | greenyellow |
| CAPN13 | tan |
| CAPN2 | purple |
| CAPN3 | turquoise |
| CAPN6 | blue |
| CAPN8 | blue |
| CAPNS1 | turquoise |
| CAPRIN1 | red |
| CAPRIN2 | cyan |
| CAPS | brown |
| CAPS2 | blue |
| CAPZB | brown |
| CARD10 | blue |
| CARD11 | turquoise |
| CARD14 | brown |
| CARD16 | turquoise |
| CARD6 | black |
| CARD8 | turquoise |
| CARD9 | brown |
| CARHSP1 | brown |
| CARKD | brown |
| CARM1 | blue |
| CARS2 | blue |
| CASC2 | blue |
| CASC3 | yellow |
| CASC4 | lightcyan |
| CASC5 | green |
| CASD1 | red |
| CASK | red |
| CASKIN2 | yellow |
| CASP1 | turquoise |
| CASP10 | turquoise |
| CASP2 | yellow |
| CASP3 | turquoise |
| CASP4 | turquoise |
| CASP6 | brown |
| CASP7 | brown |
| CASP8 | turquoise |
| CASP8AP2 | red |
| CASS4 | turquoise |
| CAST | blue |
| CASZ1 | yellow |
| CAT | brown |
| CATSPER2 | turquoise |
| CATSPER2P1 | turquoise |
| CATSPERG | pink |
| CAV1 | black |
| CAV2 | blue |
| CBFA2T2 | yellow |
| CBL | red |
| CBLB | turquoise |
| CBLC | brown |
| CBLL1 | yellow |
| CBR1 | blue |
| CBR3 | blue |
| CBR4 | blue |
| CBS | blue |
| CBWD1 | brown |
| CBWD3 | midnightblue |
| CBWD5 | brown |
| CBWD6 | brown |
| CBWD7 | blue |
| CBX1 | green |
| CBX2 | green |
| CBX3 | turquoise |
| CBX4 | blue |
| CBX5 | yellow |
| CBX5P1 | turquoise |
| CBX6 | turquoise |
| CBX7 | turquoise |
| CBX8 | blue |
| CBY1 | turquoise |
| CC2D1A | yellow |
| CC2D1B | yellow |
| CC2D2A | blue |
| CC2D2B | turquoise |
| CCBL1 | darkgreen |
| CCBL2 | pink |
| CCBP2 | tan |
| CCDC101 | yellow |
| CCDC102A | yellow |
| CCDC102B | black |
| CCDC103 | turquoise |
| CCDC104 | blue |
| CCDC106 | yellow |
| CCDC107 | turquoise |
| CCDC109A | blue |
| CCDC109B | turquoise |
| CCDC110 | brown |
| CCDC111 | blue |
| CCDC112 | red |
| CCDC114 | brown |
| CCDC115 | blue |
| CCDC117 | yellow |
| CCDC120 | yellow |
| CCDC122 | pink |
| CCDC123 | yellow |
| CCDC124 | turquoise |
| CCDC125 | blue |
| CCDC126 | blue |
| CCDC127 | turquoise |
| CCDC132 | pink |
| CCDC134 | blue |
| CCDC136 | turquoise |
| CCDC137 | grey60 |
| CCDC138 | brown |
| CCDC14 | brown |
| CCDC141 | turquoise |
| CCDC142 | turquoise |
| CCDC144A | blue |
| CCDC144C | blue |
| CCDC146 | turquoise |
| CCDC148 | brown |
| CCDC149 | yellow |
| CCDC15 | red |
| CCDC150 | green |
| CCDC154 | turquoise |
| CCDC159 | blue |
| CCDC160 | brown |
| CCDC17 | turquoise |
| CCDC18 | green |
| CCDC21 | green |
| CCDC22 | yellow |
| CCDC23 | green |
| CCDC24 | brown |
| CCDC25 | blue |
| CCDC28A | turquoise |
| CCDC28B | turquoise |
| CCDC3 | black |
| CCDC30 | yellow |
| CCDC34 | green |
| CCDC36 | black |
| CCDC39 | turquoise |
| CCDC40 | yellow |
| CCDC41 | red |
| CCDC45 | grey60 |
| CCDC46 | black |
| CCDC47 | grey60 |
| CCDC48 | blue |
| CCDC50 | blue |
| CCDC51 | blue |
| CCDC52 | brown |
| CCDC53 | brown |
| CCDC55 | lightyellow |
| CCDC56 | yellow |
| CCDC57 | yellow |
| CCDC58 | turquoise |
| CCDC6 | yellow |
| CCDC61 | yellow |
| CCDC64 | turquoise |
| CCDC64B | brown |
| CCDC66 | turquoise |
| CCDC69 | turquoise |
| CCDC7 | blue |
| CCDC72 | yellow |
| CCDC74A | blue |
| CCDC74B | blue |
| CCDC76 | pink |
| CCDC77 | cyan |
| CCDC78 | blue |
| CCDC8 | blue |
| CCDC80 | blue |
| CCDC82 | yellow |
| CCDC84 | turquoise |
| CCDC85B | blue |
| CCDC85C | brown |
| CCDC86 | blue |
| CCDC88A | turquoise |
| CCDC88B | turquoise |
| CCDC88C | turquoise |
| CCDC9 | yellow |
| CCDC90A | green |
| CCDC90B | pink |
| CCDC91 | cyan |
| CCDC92 | yellow |
| CCDC94 | turquoise |
| CCDC97 | yellow |
| CCDC99 | green |
| CCHCR1 | magenta |
| CCL13 | turquoise |
| CCL14 | black |
| CCL17 | turquoise |
| CCL18 | brown |
| CCL19 | turquoise |
| CCL2 | brown |
| CCL20 | brown |
| CCL21 | turquoise |
| CCL28 | blue |
| CCL3 | brown |
| CCL3L1 | turquoise |
| CCL4 | turquoise |
| CCL4L2 | turquoise |
| CCL5 | turquoise |
| CCL8 | turquoise |
| CCM2 | turquoise |
| CCNA2 | green |
| CCNB1 | green |
| CCNB1IP1 | brown |
| CCNB2 | green |
| CCNC | red |
| CCND1 | brown |
| CCND2 | turquoise |
| CCND3 | turquoise |
| CCNDBP1 | turquoise |
| CCNE1 | green |
| CCNE2 | green |
| CCNF | green |
| CCNG1 | blue |
| CCNG2 | yellow |
| CCNH | turquoise |
| CCNI | yellow |
| CCNJ | red |
| CCNK | yellow |
| CCNL1 | turquoise |
| CCNL2 | blue |
| CCNY | turquoise |
| CCNYL1 | brown |
| CCR1 | brown |
| CCR2 | turquoise |
| CCR5 | turquoise |
| CCRK | brown |
| CCRL1 | blue |
| CCRL2 | brown |
| CCRN4L | blue |
| CCS | brown |
| CCT2 | blue |
| CCT3 | lightgreen |
| CCT4 | green |
| CCT5 | turquoise |
| CCT6A | brown |
| CCT6P3 | pink |
| CCT7 | blue |
| CCT8 | blue |
| CCT8P1 | darkgreen |
| CD101 | turquoise |
| CD109 | purple |
| CD14 | brown |
| CD151 | turquoise |
| CD160 | turquoise |
| CD163 | brown |
| CD163L1 | blue |
| CD164 | red |
| CD164L2 | brown |
| CD180 | turquoise |
| CD1C | turquoise |
| CD1E | turquoise |
| CD2 | turquoise |
| CD200 | turquoise |
| CD200R1 | turquoise |
| CD207 | yellow |
| CD226 | turquoise |
| CD247 | turquoise |
| CD248 | blue |
| CD24L4 | brown |
| CD27 | turquoise |
| CD274 | turquoise |
| CD276 | yellow |
| CD28 | turquoise |
| CD2AP | magenta |
| CD300A | brown |
| CD300C | brown |
| CD300LF | turquoise |
| CD302 | black |
| CD320 | blue |
| CD33 | brown |
| CD34 | black |
| CD36 | black |
| CD37 | turquoise |
| CD38 | turquoise |
| CD3D | turquoise |
| CD3E | turquoise |
| CD3EAP | yellow |
| CD4 | turquoise |
| CD40 | turquoise |
| CD44 | brown |
| CD46 | brown |
| CD47 | turquoise |
| CD48 | turquoise |
| CD5 | turquoise |
| CD52 | turquoise |
| CD53 | turquoise |
| CD55 | turquoise |
| CD58 | brown |
| CD59 | red |
| CD63 | brown |
| CD68 | brown |
| CD69 | turquoise |
| CD7 | turquoise |
| CD70 | turquoise |
| CD72 | turquoise |
| CD74 | turquoise |
| CD79A | turquoise |
| CD79B | turquoise |
| CD80 | turquoise |
| CD81 | brown |
| CD82 | blue |
| CD83 | turquoise |
| CD84 | turquoise |
| CD86 | turquoise |
| CD8A | turquoise |
| CD8B | turquoise |
| CD9 | turquoise |
| CD93 | black |
| CD96 | turquoise |
| CD97 | turquoise |
| CD99 | lightcyan |
| CD99L2 | blue |
| CDADC1 | blue |
| CDC123 | blue |
| CDC14A | turquoise |
| CDC14B | blue |
| CDC16 | yellow |
| CDC20 | green |
| CDC25A | green |
| CDC25B | yellow |
| CDC25C | green |
| CDC2L1 | blue |
| CDC2L6 | red |
| CDC34 | blue |
| CDC37 | blue |
| CDC37L1 | brown |
| CDC42BPA | turquoise |
| CDC42BPG | brown |
| CDC42EP1 | turquoise |
| CDC42EP2 | blue |
| CDC42EP3 | lightyellow |
| CDC42EP4 | cyan |
| CDC42EP5 | blue |
| CDC42P1 | turquoise |
| CDC42SE1 | lightgreen |
| CDC42SE2 | turquoise |
| CDC45 | green |
| CDC5L | magenta |
| CDC6 | green |
| CDC7 | green |
| CDCA2 | green |
| CDCA3 | cyan |
| CDCA4 | green |
| CDCA5 | green |
| CDCA7 | green |
| CDCA7L | brown |
| CDCA8 | green |
| CDCP1 | turquoise |
| CDH1 | brown |
| CDH11 | purple |
| CDH13 | blue |
| CDH2 | purple |
| CDH23 | black |
| CDH24 | yellow |
| CDH26 | turquoise |
| CDH3 | brown |
| CDH5 | black |
| CDH6 | black |
| CDHR3 | turquoise |
| CDK1 | green |
| CDK10 | yellow |
| CDK11A | turquoise |
| CDK14 | turquoise |
| CDK16 | green |
| CDK17 | turquoise |
| CDK2 | green |
| CDK2AP1 | turquoise |
| CDK2AP2 | blue |
| CDK4 | blue |
| CDK5 | blue |
| CDK5RAP1 | darkgreen |
| CDK5RAP2 | yellow |
| CDK5RAP3 | turquoise |
| CDK6 | yellow |
| CDK7 | blue |
| CDK8 | green |
| CDKAL1 | magenta |
| CDKL5 | yellow |
| CDKN1A | lightcyan |
| CDKN1B | cyan |
| CDKN1C | blue |
| CDKN2A | blue |
| CDKN2AIPNL | red |
| CDKN2B | purple |
| CDKN2BAS | blue |
| CDKN2C | turquoise |
| CDKN2D | blue |
| CDKN3 | darkturquoise |
| CDON | blue |
| CDR1 | lightyellow |
| CDR2L | grey60 |
| CDRT1 | blue |
| CDRT4 | yellow |
| CDS1 | turquoise |
| CDT1 | green |
| CDYL | magenta |
| CDYL2 | turquoise |
| CEACAM1 | yellow |
| CEACAM19 | yellow |
| CEACAM21 | turquoise |
| CEACAM6 | blue |
| CEBPA | brown |
| CEBPB | blue |
| CEBPD | brown |
| CEBPG | brown |
| CECR1 | turquoise |
| CECR2 | tan |
| CECR4 | brown |
| CECR5 | blue |
| CECR7 | brown |
| CEL | brown |
| CELF2 | turquoise |
| CELF6 | black |
| CELSR1 | turquoise |
| CELSR2 | magenta |
| CEMP1 | yellow |
| CENPA | green |
| CENPB | blue |
| CENPBD1 | brown |
| CENPC1 | turquoise |
| CENPE | green |
| CENPF | green |
| CENPH | green |
| CENPI | green |
| CENPJ | green |
| CENPK | green |
| CENPL | green |
| CENPM | green |
| CENPN | blue |
| CENPO | green |
| CENPP | green |
| CENPQ | green |
| CENPV | magenta |
| CENPW | green |
| CEP110 | turquoise |
| CEP120 | turquoise |
| CEP135 | turquoise |
| CEP170 | brown |
| CEP192 | green |
| CEP250 | yellow |
| CEP55 | green |
| CEP57 | pink |
| CEP68 | black |
| CEP70 | brown |
| CEP72 | green |
| CEP76 | blue |
| CEP78 | red |
| CEP97 | pink |
| CEPT1 | turquoise |
| CERCAM | purple |
| CERK | turquoise |
| CERKL | turquoise |
| CES2 | yellow |
| CES3 | tan |
| CETN2 | turquoise |
| CETN3 | turquoise |
| CFB | turquoise |
| CFD | black |
| CFDP1 | turquoise |
| CFH | brown |
| CFI | black |
| CFL1 | green |
| CFL2 | blue |
| CFLAR | turquoise |
| CFLP1 | cyan |
| CFLP3 | blue |
| CFP | turquoise |
| CGN | brown |
| CGNL1 | yellow |
| CGREF1 | turquoise |
| CHAC1 | blue |
| CHAC2 | green |
| CHADL | brown |
| CHAF1A | green |
| CHAF1B | green |
| CHCHD1 | blue |
| CHCHD10 | blue |
| CHCHD2 | brown |
| CHCHD3 | blue |
| CHCHD4 | blue |
| CHCHD5 | blue |
| CHCHD6 | blue |
| CHCHD7 | yellow |
| CHCHD8 | blue |
| CHD1 | turquoise |
| CHD1L | turquoise |
| CHD3 | yellow |
| CHD6 | yellow |
| CHD7 | midnightblue |
| CHD9 | blue |
| CHDH | brown |
| CHEK1 | green |
| CHEK2 | green |
| CHERP | yellow |
| CHFR | brown |
| CHI3L1 | turquoise |
| CHI3L2 | blue |
| CHIC1 | turquoise |
| CHID1 | turquoise |
| CHKA | brown |
| CHKB | turquoise |
| CHL1 | black |
| CHM | blue |
| CHML | green |
| CHMP1A | turquoise |
| CHMP2A | blue |
| CHMP2B | turquoise |
| CHMP4A | turquoise |
| CHMP4B | yellow |
| CHMP4C | midnightblue |
| CHMP5 | greenyellow |
| CHMP6 | yellow |
| CHMP7 | turquoise |
| CHN1 | blue |
| CHN2 | brown |
| CHODL | blue |
| CHORDC1 | green |
| CHPF | turquoise |
| CHPF2 | yellow |
| CHPT1 | brown |
| CHRAC1 | darkred |
| CHRD | black |
| CHRDL1 | black |
| CHRM1 | brown |
| CHRM3 | brown |
| CHRNA10 | turquoise |
| CHRNA5 | green |
| CHRNB1 | blue |
| CHST1 | tan |
| CHST10 | yellow |
| CHST11 | brown |
| CHST12 | turquoise |
| CHST13 | brown |
| CHST15 | brown |
| CHST2 | turquoise |
| CHST3 | turquoise |
| CHST4 | turquoise |
| CHST6 | turquoise |
| CHST7 | black |
| CHST9 | brown |
| CHSY1 | blue |
| CHSY3 | blue |
| CHTF18 | green |
| CIAPIN1 | turquoise |
| CIB1 | tan |
| CIB2 | lightgreen |
| CIC | yellow |
| CIDECP | turquoise |
| CIITA | turquoise |
| CILP | turquoise |
| CILP2 | purple |
| CINP | blue |
| CIR1 | turquoise |
| CIRBP | black |
| CIRH1A | greenyellow |
| CISD1 | green |
| CISD3 | blue |
| CISH | turquoise |
| CIT | green |
| CITED2 | blue |
| CITED4 | brown |
| CIZ1 | yellow |
| CKAP2 | green |
| CKAP2L | green |
| CKAP4 | yellow |
| CKAP5 | green |
| CKB | turquoise |
| CKLF | yellow |
| CKMT1A | blue |
| CKMT1B | blue |
| CKMT2 | blue |
| CKS1B | lightgreen |
| CKS2 | darkturquoise |
| CLASP2 | blue |
| CLCA2 | tan |
| CLCF1 | yellow |
| CLCN2 | brown |
| CLCN3 | red |
| CLCN4 | cyan |
| CLCN5 | midnightblue |
| CLCN6 | blue |
| CLCN7 | yellow |
| CLDN1 | turquoise |
| CLDN11 | black |
| CLDN12 | turquoise |
| CLDN14 | darkgreen |
| CLDN15 | turquoise |
| CLDN23 | blue |
| CLDN3 | blue |
| CLDN4 | brown |
| CLDN5 | black |
| CLDN7 | turquoise |
| CLDN8 | tan |
| CLDND1 | turquoise |
| CLEC10A | turquoise |
| CLEC11A | purple |
| CLEC12B | turquoise |
| CLEC14A | black |
| CLEC16A | yellow |
| CLEC2B | brown |
| CLEC2D | turquoise |
| CLEC3B | black |
| CLEC4A | brown |
| CLEC5A | brown |
| CLEC7A | brown |
| CLIC2 | turquoise |
| CLIC3 | tan |
| CLIC4 | purple |
| CLINT1 | brown |
| CLIP2 | lightyellow |
| CLIP3 | lightyellow |
| CLIP4 | turquoise |
| CLK1 | turquoise |
| CLK2 | lightgreen |
| CLK4 | turquoise |
| CLMN | turquoise |
| CLN3 | turquoise |
| CLN5 | turquoise |
| CLN6 | green |
| CLN8 | brown |
| CLNS1A | blue |
| CLPB | blue |
| CLPP | blue |
| CLPTM1 | yellow |
| CLPTM1L | blue |
| CLSPN | green |
| CLSTN1 | yellow |
| CLSTN3 | cyan |
| CLTA | blue |
| CLTB | turquoise |
| CLTC | grey60 |
| CLTCL1 | lightcyan |
| CLU | turquoise |
| CLUAP1 | yellow |
| CLYBL | turquoise |
| CMAH | turquoise |
| CMAS | cyan |
| CMBL | turquoise |
| CMC1 | greenyellow |
| CMKLR1 | turquoise |
| CMPK1 | turquoise |
| CMPK2 | turquoise |
| CMTM1 | yellow |
| CMTM3 | blue |
| CMTM4 | turquoise |
| CMTM6 | brown |
| CMTM7 | turquoise |
| CMTM8 | brown |
| CMYA5 | blue |
| CNDP2 | brown |
| CNFN | brown |
| CNGA1 | brown |
| CNIH | red |
| CNIH2 | brown |
| CNIH3 | turquoise |
| CNIH4 | green |
| CNKSR1 | magenta |
| CNKSR3 | brown |
| CNN1 | black |
| CNN2 | blue |
| CNN3 | lightcyan |
| CNNM2 | yellow |
| CNNM3 | yellow |
| CNNM4 | magenta |
| CNO | brown |
| CNOT10 | yellow |
| CNOT3 | yellow |
| CNOT6 | yellow |
| CNOT6L | turquoise |
| CNOT7 | blue |
| CNP | turquoise |
| CNPY2 | blue |
| CNPY3 | magenta |
| CNPY4 | blue |
| CNRIP1 | black |
| CNTD2 | turquoise |
| CNTLN | turquoise |
| CNTN1 | darkturquoise |
| CNTN4 | blue |
| CNTNAP1 | black |
| CNTNAP3 | blue |
| CNTNAP3B | blue |
| COASY | yellow |
| COBL | brown |
| COBLL1 | blue |
| COG1 | yellow |
| COG3 | blue |
| COG4 | blue |
| COG6 | blue |
| COG7 | yellow |
| COG8 | brown |
| COL10A1 | lightcyan |
| COL11A1 | lightcyan |
| COL12A1 | purple |
| COL13A1 | purple |
| COL14A1 | black |
| COL15A1 | blue |
| COL16A1 | blue |
| COL17A1 | black |
| COL18A1 | lightyellow |
| COL1A1 | purple |
| COL1A2 | purple |
| COL21A1 | blue |
| COL22A1 | cyan |
| COL27A1 | magenta |
| COL28A1 | brown |
| COL3A1 | purple |
| COL4A1 | blue |
| COL4A2 | lightyellow |
| COL4A3 | turquoise |
| COL4A3BP | turquoise |
| COL4A4 | turquoise |
| COL4A5 | blue |
| COL5A1 | purple |
| COL5A2 | purple |
| COL5A3 | purple |
| COL6A1 | blue |
| COL6A2 | purple |
| COL6A3 | purple |
| COL7A1 | purple |
| COL8A1 | purple |
| COL8A2 | lightcyan |
| COL9A2 | brown |
| COL9A3 | brown |
| COLEC11 | blue |
| COLEC12 | blue |
| COLQ | blue |
| COMMD10 | turquoise |
| COMMD2 | green |
| COMMD3 | blue |
| COMMD4 | turquoise |
| COMMD5 | darkred |
| COMMD6 | salmon |
| COMMD7 | turquoise |
| COMMD8 | greenyellow |
| COMMD9 | red |
| COMP | purple |
| COMT | turquoise |
| COMTD1 | blue |
| COPA | lightgreen |
| COPB1 | red |
| COPB2 | turquoise |
| COPE | blue |
| COPG | turquoise |
| COPG2 | brown |
| COPS3 | green |
| COPS5 | midnightblue |
| COPS6 | yellow |
| COPS7A | cyan |
| COPZ2 | purple |
| COQ10B | blue |
| COQ2 | brown |
| COQ3 | green |
| COQ4 | yellow |
| COQ6 | tan |
| COQ9 | turquoise |
| CORIN | lightcyan |
| CORO1A | turquoise |
| CORO1B | blue |
| CORO1C | brown |
| CORO2A | brown |
| CORO6 | blue |
| CORO7 | turquoise |
| COTL1 | brown |
| COX11 | grey60 |
| COX17 | tan |
| COX18 | yellow |
| COX4NB | blue |
| COX5A | green |
| COX6A1 | blue |
| COX6B1 | turquoise |
| COX6C | blue |
| COX7A1 | blue |
| COX7A2 | yellow |
| COX7A2L | turquoise |
| COX7B | blue |
| COX7C | greenyellow |
| COX8A | blue |
| CP | brown |
| CPA4 | blue |
| CPAMD8 | magenta |
| CPD | blue |
| CPE | black |
| CPEB2 | blue |
| CPEB4 | turquoise |
| CPHL1 | tan |
| CPLX1 | blue |
| CPM | brown |
| CPNE1 | yellow |
| CPNE2 | yellow |
| CPNE3 | midnightblue |
| CPNE7 | blue |
| CPNE8 | blue |
| CPOX | yellow |
| CPPED1 | blue |
| CPSF1 | darkred |
| CPSF1P1 | blue |
| CPSF3 | green |
| CPSF3L | yellow |
| CPSF4 | yellow |
| CPSF6 | red |
| CPT1A | blue |
| CPT1B | yellow |
| CPT1C | black |
| CPT2 | tan |
| CPVL | brown |
| CPXM1 | blue |
| CPXM2 | blue |
| CPZ | blue |
| CR381653.1 | yellow |
| CR381653.2 | yellow |
| CR381670.1 | yellow |
| CR392039.3 | blue |
| CRABP1 | brown |
| CRABP2 | brown |
| CRADD | darkgreen |
| CRAMP1L | yellow |
| CRAT | tan |
| CRB3 | turquoise |
| CRCP | brown |
| CREB3 | turquoise |
| CREB3L1 | lightyellow |
| CREB3L2 | yellow |
| CREB3L4 | brown |
| CREB5 | blue |
| CREBBP | yellow |
| CREBL1 | magenta |
| CREBL2 | turquoise |
| CREBZF | yellow |
| CREG1 | brown |
| CRELD1 | yellow |
| CRELD2 | turquoise |
| CREM | brown |
| CRIM1 | blue |
| CRIP1 | turquoise |
| CRIP2 | turquoise |
| CRIPAK | yellow |
| CRIPT | pink |
| CRISPLD1 | brown |
| CRISPLD2 | blue |
| CRKRS | turquoise |
| CRLF3 | turquoise |
| CRLS1 | blue |
| CRMP1 | lightyellow |
| CRNDE | turquoise |
| CRNKL1 | yellow |
| CROCC | lightyellow |
| CROCCP2 | turquoise |
| CROT | tan |
| CRTAP | black |
| CRTC1 | lightyellow |
| CRTC2 | yellow |
| CRTC3 | yellow |
| CRY1 | red |
| CRY2 | black |
| CRYAB | turquoise |
| CRYBB1 | brown |
| CRYBB2P1 | yellow |
| CRYBG3 | blue |
| CRYL1 | blue |
| CRYZ | red |
| CRYZL1 | blue |
| CS | yellow |
| CSAD | lightyellow |
| CSDA | cyan |
| CSDC2 | blue |
| CSDE1 | red |
| CSE1L | green |
| CSF1 | brown |
| CSF1R | brown |
| CSF2RA | turquoise |
| CSF2RB | turquoise |
| CSF3R | brown |
| CSGALNACT1 | blue |
| CSGALNACT2 | brown |
| CSK | turquoise |
| CSMD2 | lightcyan |
| CSNK1D | yellow |
| CSNK1E | yellow |
| CSNK1G2 | yellow |
| CSNK1G3 | turquoise |
| CSNK2A1 | green |
| CSNK2A2 | yellow |
| CSNK2B | magenta |
| CSPG4 | blue |
| CSPG4P5 | yellow |
| CSPG5 | turquoise |
| CSPP1 | midnightblue |
| CSRNP1 | blue |
| CSRNP3 | blue |
| CSRP1 | blue |
| CSRP2 | brown |
| CST1 | purple |
| CST3 | turquoise |
| CST6 | blue |
| CST7 | turquoise |
| CSTA | brown |
| CSTB | turquoise |
| CSTF2 | green |
| CSTF2T | turquoise |
| CSTF3 | red |
| CT45A6 | darkgrey |
| CTA-211A9.5 | turquoise |
| CTA-217C2.1 | yellow |
| CTA-221G9.10 | yellow |
| CTA-221G9.11 | yellow |
| CTA-253N17.1 | yellow |
| CTA-256D12.12 | turquoise |
| CTA-292E10.6 | brown |
| CTA-313A17.4 | yellow |
| CTA-351J1.1 | turquoise |
| CTA-373H7.7 | turquoise |
| CTA-384D8.20 | turquoise |
| CTA-398F10.2 | brown |
| CTA-407F11.6 | turquoise |
| CTA-55I10.1 | brown |
| CTA-963H5.5 | blue |
| CTAGE5 | tan |
| CTB-109A12.1 | turquoise |
| CTB-113P19.1 | blue |
| CTB-118N6.3 | turquoise |
| CTB-131B5.4 | blue |
| CTB-13H5.1 | midnightblue |
| CTB-157D17.1 | blue |
| CTB-161A2.2 | yellow |
| CTB-161A2.3 | brown |
| CTB-161A2.4 | yellow |
| CTB-174D11.1 | black |
| CTB-36O1.7 | yellow |
| CTB-43P18.1 | turquoise |
| CTB-46B19.2 | blue |
| CTB-46E9.1 | turquoise |
| CTB-75G16.1 | turquoise |
| CTB-79E8.2 | blue |
| CTB-89H12.1 | greenyellow |
| CTB-89H12.4 | red |
| CTBP2 | turquoise |
| CTBS | brown |
| CTC-205M6.2 | turquoise |
| CTC-209H22.2 | turquoise |
| CTC-210G5.1 | tan |
| CTC-228N24.3 | blue |
| CTC-231O11.1 | turquoise |
| CTC-250P20.1 | brown |
| CTC-250P20.2 | brown |
| CTC-281B15.1 | blue |
| CTC-287O8.1 | turquoise |
| CTC-293A9.2 | salmon |
| CTC-327F10.4 | turquoise |
| CTC-338M12.3 | blue |
| CTC-338M12.4 | brown |
| CTC-348L14.1 | purple |
| CTC-358I24.1 | turquoise |
| CTC-359D24.3 | pink |
| CTC-366B18.2 | blue |
| CTC-379B2.1 | yellow |
| CTC-398G3.1 | yellow |
| CTC-426B10.1 | blue |
| CTC-428G20.3 | turquoise |
| CTC-428I3.1 | blue |
| CTC-436K13.3 | darkgrey |
| CTC-436P18.1 | yellow |
| CTC-448F2.1 | pink |
| CTC-454M9.1 | turquoise |
| CTC-463N11.3 | tan |
| CTC-499J9.1 | yellow |
| CTC-506B8.1 | blue |
| CTC-513N18.1 | blue |
| CTC-563A5.2 | yellow |
| CTC-563A5.3 | blue |
| CTC-564N23.2 | yellow |
| CTC-564N23.3 | turquoise |
| CTD-2001E22.2 | lightcyan |
| CTD-2006C1.2 | turquoise |
| CTD-2015H6.3 | turquoise |
| CTD-2031P19.4 | salmon |
| CTD-2034L19.1 | yellow |
| CTD-2037K23.2 | turquoise |
| CTD-2046J7.1 | brown |
| CTD-2048F20.1 | turquoise |
| CTD-2061E19.5 | turquoise |
| CTD-2062O1.3 | brown |
| CTD-2117L12.1 | darkgrey |
| CTD-2124B20.1 | salmon |
| CTD-2193P3.2 | blue |
| CTD-2195M18.1 | darkgreen |
| CTD-2197O4.1 | blue |
| CTD-2201E18.3 | yellow |
| CTD-2224J9.2 | yellow |
| CTD-2228K2.5 | brown |
| CTD-2230D16.1 | black |
| CTD-2231H16.1 | magenta |
| CTD-2235C13.1 | brown |
| CTD-2260A17.1 | turquoise |
| CTD-2275D10.1 | turquoise |
| CTD-2283N19.1 | blue |
| CTD-2301A4.3 | turquoise |
| CTD-2313F11.1 | darkgrey |
| CTD-2314I6.1 | blue |
| CTD-2330K9.2 | blue |
| CTD-2331H7.1 | black |
| CTD-2336O2.1 | blue |
| CTD-2339M3.1 | turquoise |
| CTD-2340D6.2 | midnightblue |
| CTD-2353F22.1 | turquoise |
| CTD-2375G15.1 | blue |
| CTD-2522F11.1 | blue |
| CTD-2526A2.1 | yellow |
| CTD-2530N21.4 | brown |
| CTD-2544N14.3 | turquoise |
| CTD-2547E10.2 | turquoise |
| CTD-2562G15.1 | greenyellow |
| CTD-2600H12.1 | darkgreen |
| CTD-2653M23.1 | turquoise |
| CTD-2666L21.1 | brown |
| CTD-2666L21.2 | turquoise |
| CTD-3083F21.1 | turquoise |
| CTD-3083F21.5 | turquoise |
| CTD-3107M8.4 | blue |
| CTD-3184A7.4 | turquoise |
| CTD-3211M3.1 | yellow |
| CTD-3233P19.1 | pink |
| CTD-3236F5.1 | turquoise |
| CTDP1 | yellow |
| CTDSP2 | blue |
| CTDSPL | turquoise |
| CTF1 | blue |
| CTGF | blue |
| CTH | blue |
| CTHRC1 | purple |
| CTLA4 | turquoise |
| CTNNAL1 | blue |
| CTNNB1 | turquoise |
| CTNNBIP1 | tan |
| CTNNBL1 | turquoise |
| CTNND1 | red |
| CTPS | green |
| CTPS2 | red |
| CTRL | turquoise |
| CTSA | brown |
| CTSB | brown |
| CTSC | turquoise |
| CTSD | brown |
| CTSF | blue |
| CTSH | turquoise |
| CTSK | purple |
| CTSL1 | brown |
| CTSL2 | turquoise |
| CTSO | blue |
| CTSS | turquoise |
| CTSW | turquoise |
| CTSZ | brown |
| CTTN | turquoise |
| CTTNBP2NL | yellow |
| CTU1 | blue |
| CTU2 | blue |
| CTXN1 | magenta |
| CUEDC1 | yellow |
| CUL2 | green |
| CUL4A | brown |
| CUL4B | red |
| CUL7 | magenta |
| CUL9 | magenta |
| CUTA | magenta |
| CUTC | turquoise |
| CUX1 | lightyellow |
| CUX2 | darkgreen |
| CWC27 | yellow |
| CWF19L2 | turquoise |
| CX3CL1 | yellow |
| CX3CR1 | blue |
| CXADR | brown |
| CXCL1 | brown |
| CXCL10 | turquoise |
| CXCL11 | turquoise |
| CXCL12 | black |
| CXCL13 | turquoise |
| CXCL14 | black |
| CXCL16 | brown |
| CXCL17 | tan |
| CXCL2 | brown |
| CXCL3 | brown |
| CXCL5 | brown |
| CXCL9 | turquoise |
| CXCR4 | turquoise |
| CXCR6 | turquoise |
| CXCR7 | blue |
| CXorf15 | red |
| CXorf21 | turquoise |
| CXorf22 | blue |
| CXorf23 | blue |
| CXorf24 | red |
| CXorf26 | blue |
| CXorf36 | black |
| CXorf38 | turquoise |
| CXorf40A | blue |
| CXorf40B | blue |
| CXorf56 | blue |
| CXXC4 | brown |
| CXXC5 | brown |
| CYB561 | grey60 |
| CYB561D2 | brown |
| CYB5A | tan |
| CYB5D1 | yellow |
| CYB5D2 | blue |
| CYB5R1 | turquoise |
| CYB5R2 | turquoise |
| CYB5R3 | yellow |
| CYB5R4 | brown |
| CYB5RL | yellow |
| CYBA | turquoise |
| CYBASC3 | turquoise |
| CYBB | turquoise |
| CYBRD1 | black |
| CYC1 | darkred |
| CYCS | blue |
| CYFIP2 | turquoise |
| CYGB | black |
| CYHR1 | darkred |
| CYLD | turquoise |
| CYP1B1 | turquoise |
| CYP26B1 | yellow |
| CYP27A1 | cyan |
| CYP27B1 | turquoise |
| CYP27C1 | turquoise |
| CYP2D6 | yellow |
| CYP2D7P1 | yellow |
| CYP2D8P1 | yellow |
| CYP2E1 | blue |
| CYP2J2 | brown |
| CYP2R1 | tan |
| CYP2U1 | black |
| CYP39A1 | brown |
| CYP3A5 | turquoise |
| CYP4V2 | turquoise |
| CYP51A1 | turquoise |
| CYP7B1 | turquoise |
| CYR61 | blue |
| CYS1 | purple |
| CYSLTR1 | turquoise |
| CYTH1 | turquoise |
| CYTH2 | yellow |
| CYTH3 | blue |
| CYTH4 | turquoise |
| CYTIP | turquoise |
| CYTL1 | black |
| CYTSA | yellow |
| CYTSB | blue |
| CYYR1 | blue |
| D2HGDH | yellow |
| DAAM1 | red |
| DAAM2 | black |
| DAB2 | black |
| DAB2IP | yellow |
| DACT1 | purple |
| DACT3 | blue |
| DAD1 | yellow |
| DAG1 | turquoise |
| DAGLA | yellow |
| DAGLB | yellow |
| DAK | brown |
| DAP | turquoise |
| DAP3 | lightgreen |
| DAPK1 | brown |
| DAPK2 | yellow |
| DAPK3 | turquoise |
| DAPP1 | turquoise |
| DAQB-335A13.8 | turquoise |
| DARC | turquoise |
| DARS2 | green |
| DAXX | magenta |
| DAZAP1 | green |
| DBF4 | green |
| DBF4B | magenta |
| DBI | tan |
| DBN1 | magenta |
| DBNDD1 | turquoise |
| DBNDD2 | blue |
| DBP | turquoise |
| DCAF10 | brown |
| DCAF12 | green |
| DCAF13 | midnightblue |
| DCAF16 | pink |
| DCAF4 | brown |
| DCAF5 | blue |
| DCAF6 | turquoise |
| DCAF7 | grey60 |
| DCAKD | yellow |
| DCBLD1 | purple |
| DCBLD2 | blue |
| DCHS1 | blue |
| DCHS2 | cyan |
| DCI | brown |
| DCK | turquoise |
| DCLK1 | black |
| DCLK2 | blue |
| DCLRE1C | yellow |
| DCN | purple |
| DCP1B | cyan |
| DCP2 | turquoise |
| DCPS | blue |
| DCST2 | brown |
| DCTN1 | yellow |
| DCTN3 | yellow |
| DCTN5 | red |
| DCTN6 | turquoise |
| DCTPP1 | turquoise |
| DCUN1D2 | green |
| DCUN1D3 | black |
| DCUN1D4 | turquoise |
| DCUN1D5 | blue |
| DCXR | blue |
| DDA1 | blue |
| DDAH1 | blue |
| DDAH2 | magenta |
| DDB2 | turquoise |
| DDHD1 | turquoise |
| DDHD2 | blue |
| DDIT3 | turquoise |
| DDIT4 | blue |
| DDR1 | brown |
| DDR2 | blue |
| DDRGK1 | blue |
| DDT | blue |
| DDX10 | red |
| DDX11 | cyan |
| DDX12 | cyan |
| DDX21 | green |
| DDX26B | turquoise |
| DDX27 | green |
| DDX31 | brown |
| DDX39 | green |
| DDX41 | turquoise |
| DDX42 | grey60 |
| DDX43 | turquoise |
| DDX47 | cyan |
| DDX49 | blue |
| DDX5 | turquoise |
| DDX51 | brown |
| DDX52 | pink |
| DDX55 | green |
| DDX58 | turquoise |
| DDX60 | turquoise |
| DDX60L | turquoise |
| DEAF1 | brown |
| DECR1 | midnightblue |
| DECR2 | brown |
| DEDD | lightgreen |
| DEDD2 | turquoise |
| DEF6 | turquoise |
| DEF8 | turquoise |
| DEFB1 | yellow |
| DEGS1 | green |
| DEGS2 | brown |
| DEK | green |
| DEM1 | green |
| DENND1A | turquoise |
| DENND1B | turquoise |
| DENND1C | turquoise |
| DENND2A | blue |
| DENND2C | blue |
| DENND2D | turquoise |
| DENND3 | turquoise |
| DENND4A | turquoise |
| DENND4B | yellow |
| DENND4C | blue |
| DENND5A | brown |
| DENND5B | turquoise |
| DENR | green |
| DEPDC1 | green |
| DEPDC1B | green |
| DEPDC5 | turquoise |
| DEPDC6 | blue |
| DEPDC7 | purple |
| DERA | cyan |
| DERL1 | midnightblue |
| DERL3 | turquoise |
| DES | blue |
| DET1 | blue |
| DFFA | blue |
| DFFB | turquoise |
| DFNA5 | blue |
| DFNB31 | yellow |
| DFNB59 | blue |
| DGAT1 | darkred |
| DGAT2 | blue |
| DGCR5 | turquoise |
| DGCR6 | turquoise |
| DGCR6L | turquoise |
| DGCR8 | yellow |
| DGKA | turquoise |
| DGKD | blue |
| DGKE | turquoise |
| DGKG | turquoise |
| DGKH | pink |
| DGKI | purple |
| DGKQ | turquoise |
| DGKZ | blue |
| DGUOK | blue |
| DHCR24 | tan |
| DHCR7 | turquoise |
| DHDH | turquoise |
| DHFR | green |
| DHODH | brown |
| DHRS1 | brown |
| DHRS11 | brown |
| DHRS12 | blue |
| DHRS13 | blue |
| DHRS2 | tan |
| DHRS3 | blue |
| DHRS4 | tan |
| DHRS4L2 | tan |
| DHRS7 | blue |
| DHRS7B | blue |
| DHRSX | turquoise |
| DHTKD1 | blue |
| DHX16 | magenta |
| DHX32 | turquoise |
| DHX33 | lightgreen |
| DHX34 | yellow |
| DHX36 | blue |
| DHX40 | grey60 |
| DHX57 | pink |
| DHX58 | turquoise |
| DIAPH1 | turquoise |
| DIAPH2 | brown |
| DIAPH3 | green |
| DIO2 | lightcyan |
| DIP2A | pink |
| DIP2B | yellow |
| DIP2C | yellow |
| DIRAS1 | brown |
| DIRC2 | red |
| DIS3L2 | yellow |
| DISC1 | turquoise |
| DISP1 | black |
| DIXDC1 | blue |
| DKC1 | blue |
| DKK1 | blue |
| DKK3 | blue |
| DLAT | red |
| DLC1 | blue |
| DLEU1 | darkgreen |
| DLEU2 | turquoise |
| DLG1 | green |
| DLG3 | brown |
| DLG4 | lightyellow |
| DLG5 | yellow |
| DLGAP1 | cyan |
| DLGAP4 | yellow |
| DLGAP5 | green |
| DLL1 | blue |
| DLL4 | blue |
| DMAP1 | yellow |
| DMC1 | blue |
| DMD | blue |
| DMGDH | blue |
| DMKN | turquoise |
| DMPK | yellow |
| DMRTA1 | turquoise |
| DMWD | yellow |
| DMXL1 | turquoise |
| DMXL2 | brown |
| DNA2 | green |
| DNAH1 | turquoise |
| DNAH11 | turquoise |
| DNAH14 | green |
| DNAH17 | yellow |
| DNAJA1 | green |
| DNAJA2 | midnightblue |
| DNAJA3 | blue |
| DNAJA4 | brown |
| DNAJB1 | turquoise |
| DNAJB11 | green |
| DNAJB2 | brown |
| DNAJB4 | blue |
| DNAJB5 | blue |
| DNAJB6 | turquoise |
| DNAJB9 | turquoise |
| DNAJC1 | yellow |
| DNAJC10 | pink |
| DNAJC11 | yellow |
| DNAJC15 | brown |
| DNAJC16 | red |
| DNAJC18 | black |
| DNAJC19 | brown |
| DNAJC2 | green |
| DNAJC21 | brown |
| DNAJC22 | tan |
| DNAJC25 | greenyellow |
| DNAJC3 | brown |
| DNAJC30 | brown |
| DNAJC4 | yellow |
| DNAJC6 | blue |
| DNAJC9 | green |
| DNAL4 | turquoise |
| DNASE1 | yellow |
| DNASE1L1 | brown |
| DNASE1L2 | brown |
| DNASE2 | blue |
| DNHD1 | turquoise |
| DNLZ | blue |
| DNM1 | blue |
| DNM1L | cyan |
| DNM2 | darkgreen |
| DNM3 | blue |
| DNMBP | blue |
| DNMT1 | green |
| DNMT3A | magenta |
| DNMT3B | turquoise |
| DNTTIP1 | yellow |
| DOC2A | salmon |
| DOC2B | turquoise |
| DOCK1 | turquoise |
| DOCK10 | turquoise |
| DOCK11 | turquoise |
| DOCK2 | turquoise |
| DOCK3 | cyan |
| DOCK4 | black |
| DOCK5 | turquoise |
| DOCK6 | yellow |
| DOCK7 | turquoise |
| DOCK8 | turquoise |
| DOCK9 | blue |
| DOHH | blue |
| DOK1 | turquoise |
| DOK2 | turquoise |
| DOK3 | turquoise |
| DOK4 | blue |
| DOK5 | blue |
| DOK7 | blue |
| DOLPP1 | blue |
| DOM3Z | magenta |
| DONSON | green |
| DOPEY1 | turquoise |
| DOPEY2 | yellow |
| DOT1L | yellow |
| DPAGT1 | red |
| DPCD | turquoise |
| DPEP1 | turquoise |
| DPEP2 | turquoise |
| DPF2 | yellow |
| DPH2 | green |
| DPM1 | blue |
| DPM2 | green |
| DPM3 | blue |
| DPP4 | black |
| DPP7 | turquoise |
| DPP9 | brown |
| DPT | blue |
| DPY19L1 | blue |
| DPY19L2P2 | turquoise |
| DPY19L3 | pink |
| DPY19L4 | midnightblue |
| DPY30 | turquoise |
| DPYD | brown |
| DPYSL2 | blue |
| DPYSL3 | blue |
| DR1 | red |
| DRAM1 | brown |
| DRAM2 | brown |
| DRAP1 | blue |
| DRG1 | blue |
| DSC2 | brown |
| DSC3 | brown |
| DSCC1 | midnightblue |
| DSE | brown |
| DSEL | black |
| DSG1 | brown |
| DSG2 | turquoise |
| DSG3 | brown |
| DSN1 | green |
| DSP | magenta |
| DST | blue |
| DSTN | turquoise |
| DTD1 | brown |
| DTL | green |
| DTNA | turquoise |
| DTNB | magenta |
| DTNBP1 | yellow |
| DTWD1 | blue |
| DTWD2 | blue |
| DTX2 | yellow |
| DTX3 | blue |
| DTX3L | turquoise |
| DTX4 | yellow |
| DTYMK | green |
| DULLARD | yellow |
| DUOX1 | brown |
| DUOXA1 | turquoise |
| DUS1L | grey60 |
| DUS3L | blue |
| DUSP1 | blue |
| DUSP10 | brown |
| DUSP12 | lightgreen |
| DUSP14 | turquoise |
| DUSP16 | cyan |
| DUSP18 | lightyellow |
| DUSP2 | turquoise |
| DUSP22 | turquoise |
| DUSP23 | blue |
| DUSP4 | turquoise |
| DUSP5 | turquoise |
| DUSP5P | turquoise |
| DUSP6 | blue |
| DUSP7 | yellow |
| DUSP8 | yellow |
| DUT | blue |
| DVL1 | blue |
| DVL2 | yellow |
| DVL3 | greenyellow |
| DYM | red |
| DYNC1I1 | blue |
| DYNC1LI2 | turquoise |
| DYNC2H1 | blue |
| DYNC2LI1 | pink |
| DYNLL2 | grey60 |
| DYNLRB1 | greenyellow |
| DYNLT1 | blue |
| DYNLT3 | blue |
| DYRK1B | yellow |
| DYRK2 | turquoise |
| DYRK3 | blue |
| DYRK4 | cyan |
| DYSF | lightyellow |
| DYX1C1 | turquoise |
| DZIP1 | blue |
| DZIP1L | blue |
| DZIP3 | blue |
| E2F1 | green |
| E2F2 | green |
| E2F3 | green |
| E2F5 | midnightblue |
| E2F6 | red |
| EAF2 | turquoise |
| EAPP | red |
| EARS2 | brown |
| EBAG9 | midnightblue |
| EBF1 | black |
| EBF4 | blue |
| EBI3 | turquoise |
| EBNA1BP2 | green |
| EBP | turquoise |
| EBPL | turquoise |
| ECE1 | lightyellow |
| ECE2 | black |
| ECH1 | blue |
| ECHDC1 | turquoise |
| ECHDC2 | darkgreen |
| ECHDC3 | blue |
| ECHS1 | blue |
| ECM1 | purple |
| ECM2 | blue |
| ECSCR | black |
| ECSIT | blue |
| ECT2 | green |
| EDA2R | black |
| EDARADD | blue |
| EDEM1 | turquoise |
| EDIL3 | lightcyan |
| EDN1 | blue |
| EDN2 | brown |
| EDNRA | blue |
| EDNRB | black |
| EEA1 | blue |
| EEF1A1 | salmon |
| EEF1A1P25 | midnightblue |
| EEF1A1P32 | turquoise |
| EEF1A1P6 | yellow |
| EEF1AL1 | turquoise |
| EEF1AL3 | salmon |
| EEF1B2 | salmon |
| EEF1D | darkred |
| EEF1DP2 | turquoise |
| EEF1DP3 | blue |
| EEF1DP5 | blue |
| EEF1E1 | blue |
| EEF2 | salmon |
| EEF2K | yellow |
| EEFSEC | yellow |
| EEPD1 | blue |
| EFCAB10 | blue |
| EFCAB2 | blue |
| EFCAB4A | brown |
| EFCAB4B | turquoise |
| EFCAB7 | pink |
| EFEMP1 | black |
| EFEMP2 | blue |
| EFHA2 | blue |
| EFHC1 | blue |
| EFHD1 | brown |
| EFHD2 | brown |
| EFNA1 | turquoise |
| EFNA3 | turquoise |
| EFNA4 | brown |
| EFNA5 | yellow |
| EFNB1 | blue |
| EFNB2 | blue |
| EFR3A | midnightblue |
| EFS | magenta |
| EFTUD1 | green |
| EGF | brown |
| EGFL7 | black |
| EGFL8 | blue |
| EGFLAM | black |
| EGFR | brown |
| EGLN1 | turquoise |
| EGLN2 | turquoise |
| EGLN3 | turquoise |
| EGR1 | blue |
| EGR2 | turquoise |
| EGR3 | blue |
| EHBP1 | brown |
| EHBP1L1 | turquoise |
| EHD1 | yellow |
| EHD2 | purple |
| EHD3 | black |
| EHF | brown |
| EHHADH | yellow |
| EHMT1 | yellow |
| EHMT2 | magenta |
| EI24 | red |
| EID2 | turquoise |
| EIF1 | blue |
| EIF1AX | green |
| EIF1B | blue |
| EIF2A | green |
| EIF2AK1 | turquoise |
| EIF2AK2 | turquoise |
| EIF2B4 | red |
| EIF2B5 | green |
| EIF2C1 | yellow |
| EIF2C2 | darkred |
| EIF2C3 | pink |
| EIF2C4 | pink |
| EIF2S1 | blue |
| EIF2S3 | red |
| EIF3B | blue |
| EIF3E | midnightblue |
| EIF3F | salmon |
| EIF3G | blue |
| EIF3H | midnightblue |
| EIF3I | turquoise |
| EIF3K | blue |
| EIF3L | salmon |
| EIF3M | red |
| EIF3S8 | greenyellow |
| EIF4A2 | royalblue |
| EIF4A3 | green |
| EIF4B | salmon |
| EIF4E3 | turquoise |
| EIF4EBP1 | turquoise |
| EIF4EBP2 | yellow |
| EIF4EBP3 | salmon |
| EIF4ENIF1 | yellow |
| EIF4G1 | green |
| EIF4G2 | red |
| EIF4G3 | lightyellow |
| EIF5 | blue |
| EIF5A | blue |
| EIF5A2 | purple |
| EIF6 | turquoise |
| ELF1 | turquoise |
| ELF3 | brown |
| ELF4 | brown |
| ELF5 | blue |
| ELK1 | yellow |
| ELK3 | blue |
| ELK4 | turquoise |
| ELL2 | brown |
| ELL3 | blue |
| ELMO1 | turquoise |
| ELMO3 | brown |
| ELMOD2 | blue |
| ELN | blue |
| ELOF1 | blue |
| ELOVL1 | turquoise |
| ELOVL4 | turquoise |
| ELOVL5 | darkgreen |
| ELOVL6 | green |
| ELOVL7 | darkgreen |
| ELP2 | red |
| ELP3 | turquoise |
| ELP4 | red |
| ELTD1 | black |
| EMB | turquoise |
| EMCN | black |
| EMD | greenyellow |
| EME1 | green |
| EME2 | yellow |
| EMG1 | cyan |
| EMID1 | turquoise |
| EMILIN1 | purple |
| EMILIN2 | brown |
| EMILIN3 | turquoise |
| EML1 | blue |
| EML2 | blue |
| EML3 | turquoise |
| EML5 | tan |
| EML6 | turquoise |
| EMP1 | blue |
| EMP2 | turquoise |
| EMP3 | brown |
| EMR2 | brown |
| EN1 | blue |
| ENAH | turquoise |
| ENC1 | blue |
| ENDOD1 | turquoise |
| ENDOG | blue |
| ENG | black |
| ENGASE | turquoise |
| ENHO | brown |
| ENO1 | turquoise |
| ENO2 | purple |
| ENO3 | turquoise |
| ENOPH1 | red |
| ENOSF1 | yellow |
| ENPP1 | lightcyan |
| ENPP2 | turquoise |
| ENPP3 | tan |
| ENPP4 | blue |
| ENPP5 | brown |
| ENSA | lightgreen |
| ENTPD1 | turquoise |
| ENTPD2 | brown |
| ENTPD4 | turquoise |
| ENTPD5 | tan |
| ENTPD6 | brown |
| ENTPD7 | lightcyan |
| ENY2 | midnightblue |
| EP300 | yellow |
| EP400 | yellow |
| EP400NL | yellow |
| EPAS1 | black |
| EPB41 | turquoise |
| EPB41L1 | turquoise |
| EPB41L2 | blue |
| EPB41L3 | brown |
| EPB41L4A | blue |
| EPB41L4B | turquoise |
| EPB41L5 | brown |
| EPB49 | yellow |
| EPC1 | turquoise |
| EPC2 | turquoise |
| EPCAM | brown |
| EPDR1 | blue |
| EPHA1 | brown |
| EPHA2 | blue |
| EPHA3 | black |
| EPHA4 | turquoise |
| EPHB1 | blue |
| EPHB2 | brown |
| EPHB3 | brown |
| EPHB4 | brown |
| EPHB6 | yellow |
| EPHX1 | greenyellow |
| EPHX2 | blue |
| EPHX4 | brown |
| EPM2A | pink |
| EPM2AIP1 | turquoise |
| EPN1 | yellow |
| EPN2 | yellow |
| EPN3 | brown |
| EPOR | blue |
| EPRS | green |
| EPS15 | turquoise |
| EPS15L1 | yellow |
| EPS8 | blue |
| EPS8L1 | brown |
| EPS8L2 | brown |
| EPSTI1 | turquoise |
| EPT1 | green |
| ERAL1 | green |
| ERAP1 | turquoise |
| ERAP2 | turquoise |
| ERBB2 | brown |
| ERBB3 | brown |
| ERC1 | cyan |
| ERCC2 | yellow |
| ERCC5 | turquoise |
| ERCC6 | pink |
| ERF | magenta |
| ERG | black |
| ERGIC1 | blue |
| ERGIC2 | cyan |
| ERGIC3 | brown |
| ERH | yellow |
| ERI1 | blue |
| ERI2 | red |
| ERI3 | green |
| ERICH1 | turquoise |
| ERLIN2 | turquoise |
| ERMAP | blue |
| ERMN | turquoise |
| ERMP1 | turquoise |
| ERN1 | turquoise |
| ERO1L | turquoise |
| ERO1LB | turquoise |
| ERP27 | brown |
| ERP29 | yellow |
| ERRFI1 | turquoise |
| ERV3 | brown |
| ESAM | black |
| ESCO2 | green |
| ESD | blue |
| ESM1 | turquoise |
| ESPL1 | green |
| ESPN | yellow |
| ESR1 | turquoise |
| ESRP1 | darkred |
| ESRP2 | brown |
| ESRRA | blue |
| ESRRG | brown |
| ESYT2 | turquoise |
| ESYT3 | magenta |
| ETAA1 | red |
| ETFA | tan |
| ETFB | brown |
| ETFDH | blue |
| ETHE1 | yellow |
| ETNK1 | cyan |
| ETNK2 | brown |
| ETS1 | turquoise |
| ETS2 | blue |
| ETV1 | blue |
| ETV3 | magenta |
| ETV5 | blue |
| ETV6 | cyan |
| ETV7 | turquoise |
| EVC | blue |
| EVC2 | lightyellow |
| EVI2A | brown |
| EVI2B | turquoise |
| EVI5L | yellow |
| EVL | turquoise |
| EVPL | grey60 |
| EXD3 | yellow |
| EXO1 | green |
| EXOC1 | blue |
| EXOC2 | turquoise |
| EXOC3L | brown |
| EXOC3L2 | lightyellow |
| EXOC6 | turquoise |
| EXOC6B | yellow |
| EXOC7 | yellow |
| EXOG | turquoise |
| EXOSC10 | red |
| EXOSC2 | green |
| EXOSC3 | blue |
| EXOSC4 | darkred |
| EXOSC5 | blue |
| EXOSC6 | green |
| EXOSC8 | pink |
| EXOSC9 | red |
| EXPH5 | brown |
| EXT1 | midnightblue |
| EXT2 | red |
| EXTL1 | turquoise |
| EXTL2 | pink |
| EXTL3 | turquoise |
| EYA2 | brown |
| EYS | darkgreen |
| EZH1 | turquoise |
| EZH2 | green |
| F11R | lightgreen |
| F12 | turquoise |
| F13A1 | black |
| F2R | black |
| F2RL1 | turquoise |
| F2RL2 | black |
| F3 | blue |
| F8 | blue |
| F8A1 | greenyellow |
| FAAH | brown |
| FAAH2 | turquoise |
| FABP3 | brown |
| FABP4 | black |
| FABP5 | brown |
| FABP7 | blue |
| FADD | blue |
| FADS1 | turquoise |
| FADS2 | brown |
| FADS3 | blue |
| FAF1 | green |
| FAF2 | yellow |
| FAH | tan |
| FAHD1 | turquoise |
| FAHD2A | brown |
| FAHD2B | brown |
| FAIM | turquoise |
| FAIM3 | turquoise |
| FAM100A | yellow |
| FAM100B | yellow |
| FAM101A | blue |
| FAM101B | blue |
| FAM102A | yellow |
| FAM102B | pink |
| FAM103A1 | turquoise |
| FAM104B | brown |
| FAM105A | turquoise |
| FAM105B | turquoise |
| FAM106B | blue |
| FAM107A | blue |
| FAM107B | turquoise |
| FAM108B1 | pink |
| FAM108C1 | brown |
| FAM109A | turquoise |
| FAM109B | purple |
| FAM110A | blue |
| FAM110B | blue |
| FAM110C | orange |
| FAM111A | turquoise |
| FAM111B | green |
| FAM113A | turquoise |
| FAM113B | turquoise |
| FAM114A1 | lightcyan |
| FAM114A2 | turquoise |
| FAM115A | brown |
| FAM115C | turquoise |
| FAM116A | turquoise |
| FAM116B | yellow |
| FAM117A | turquoise |
| FAM117B | yellow |
| FAM118A | blue |
| FAM119B | orange |
| FAM120B | pink |
| FAM120C | magenta |
| FAM122A | turquoise |
| FAM122B | yellow |
| FAM122C | yellow |
| FAM123B | magenta |
| FAM125A | blue |
| FAM125B | turquoise |
| FAM126A | blue |
| FAM126B | turquoise |
| FAM127A | turquoise |
| FAM127B | greenyellow |
| FAM127C | greenyellow |
| FAM128A | blue |
| FAM128B | blue |
| FAM129A | turquoise |
| FAM129B | turquoise |
| FAM131A | brown |
| FAM132A | yellow |
| FAM133B | turquoise |
| FAM134B | blue |
| FAM135A | red |
| FAM136A | green |
| FAM13A | blue |
| FAM13AOS | black |
| FAM13B | turquoise |
| FAM13C | black |
| FAM149A | turquoise |
| FAM150B | brown |
| FAM156A | yellow |
| FAM157B | tan |
| FAM158A | darkturquoise |
| FAM159A | turquoise |
| FAM160A1 | brown |
| FAM160B1 | turquoise |
| FAM160B2 | lightyellow |
| FAM161A | red |
| FAM162A | turquoise |
| FAM164A | blue |
| FAM166A | red |
| FAM166B | yellow |
| FAM167B | blue |
| FAM171A1 | magenta |
| FAM171B | brown |
| FAM172A | blue |
| FAM173A | yellow |
| FAM173B | turquoise |
| FAM174A | turquoise |
| FAM174B | turquoise |
| FAM175A | blue |
| FAM176A | purple |
| FAM176B | blue |
| FAM177A1 | turquoise |
| FAM178A | turquoise |
| FAM180A | blue |
| FAM183A | brown |
| FAM184A | red |
| FAM185A | purple |
| FAM188A | blue |
| FAM188B | brown |
| FAM189B | lightgreen |
| FAM18B | blue |
| FAM18B2 | turquoise |
| FAM190 | turquoise |
| FAM190A | black |
| FAM192A | brown |
| FAM193B | blue |
| FAM194B | blue |
| FAM195A | blue |
| FAM195B | grey60 |
| FAM196B | turquoise |
| FAM198A | brown |
| FAM19A5 | purple |
| FAM200B | turquoise |
| FAM20A | turquoise |
| FAM20B | green |
| FAM20C | salmon |
| FAM21A | turquoise |
| FAM21C | turquoise |
| FAM22D | yellow |
| FAM24B | turquoise |
| FAM26F | turquoise |
| FAM35A | red |
| FAM36A | brown |
| FAM38A | yellow |
| FAM3A | brown |
| FAM3B | brown |
| FAM3C | yellow |
| FAM3D | brown |
| FAM40B | turquoise |
| FAM41C | yellow |
| FAM43A | black |
| FAM46A | blue |
| FAM46B | brown |
| FAM46C | turquoise |
| FAM47E | blue |
| FAM48A | blue |
| FAM49A | turquoise |
| FAM49B | brown |
| FAM50A | blue |
| FAM50B | brown |
| FAM53A | yellow |
| FAM53B | turquoise |
| FAM54A | green |
| FAM55C | turquoise |
| FAM57A | turquoise |
| FAM58A | blue |
| FAM59A | brown |
| FAM59B | yellow |
| FAM60A | cyan |
| FAM63A | brown |
| FAM63B | lightcyan |
| FAM64A | green |
| FAM65A | lightyellow |
| FAM65B | turquoise |
| FAM65C | turquoise |
| FAM66A | blue |
| FAM69A | turquoise |
| FAM69B | brown |
| FAM70A | turquoise |
| FAM70B | black |
| FAM71F2 | yellow |
| FAM72A | brown |
| FAM72B | brown |
| FAM73A | pink |
| FAM73B | turquoise |
| FAM76B | pink |
| FAM78A | turquoise |
| FAM78B | brown |
| FAM81A | turquoise |
| FAM82A1 | black |
| FAM82A2 | brown |
| FAM82B | midnightblue |
| FAM83B | brown |
| FAM83D | green |
| FAM83F | blue |
| FAM83G | blue |
| FAM83H | darkred |
| FAM84A | brown |
| FAM84B | brown |
| FAM86A | blue |
| FAM86B1 | yellow |
| FAM86C | yellow |
| FAM86D | yellow |
| FAM89A | brown |
| FAM89B | blue |
| FAM8A1 | magenta |
| FAM90A1 | cyan |
| FAM91A1 | midnightblue |
| FAM92A1 | red |
| FAM95B1 | turquoise |
| FAM96A | brown |
| FAM96B | blue |
| FAM98A | turquoise |
| FAM98C | brown |
| FANCA | green |
| FANCB | green |
| FANCC | yellow |
| FANCD2 | green |
| FANCE | magenta |
| FANCF | red |
| FANCG | green |
| FANCI | green |
| FANCL | red |
| FANK1 | blue |
| FAP | purple |
| FAR1 | red |
| FAR2 | brown |
| FARP1 | turquoise |
| FARP2 | yellow |
| FARS2 | pink |
| FARSA | blue |
| FARSB | green |
| FAS | turquoise |
| FASLG | turquoise |
| FASN | tan |
| FASTK | blue |
| FASTKD1 | turquoise |
| FASTKD3 | green |
| FAT1 | turquoise |
| FAT2 | blue |
| FAT4 | black |
| FBF1 | yellow |
| FBL | salmon |
| FBLIM1 | turquoise |
| FBLN1 | blue |
| FBLN2 | blue |
| FBLN5 | blue |
| FBLN7 | black |
| FBN1 | purple |
| FBN2 | turquoise |
| FBP1 | brown |
| FBRS | yellow |
| FBRSL1 | yellow |
| FBXL13 | cyan |
| FBXL14 | cyan |
| FBXL16 | brown |
| FBXL17 | turquoise |
| FBXL18 | yellow |
| FBXL19 | blue |
| FBXL2 | turquoise |
| FBXL20 | turquoise |
| FBXL3 | blue |
| FBXL4 | red |
| FBXL6 | darkred |
| FBXL8 | turquoise |
| FBXO10 | yellow |
| FBXO16 | brown |
| FBXO18 | yellow |
| FBXO2 | purple |
| FBXO22 | green |
| FBXO25 | black |
| FBXO27 | brown |
| FBXO3 | red |
| FBXO30 | pink |
| FBXO32 | blue |
| FBXO38 | turquoise |
| FBXO4 | turquoise |
| FBXO41 | yellow |
| FBXO44 | turquoise |
| FBXO45 | green |
| FBXO46 | yellow |
| FBXO5 | green |
| FBXO6 | turquoise |
| FBXO9 | magenta |
| FBXW5 | turquoise |
| FBXW7 | turquoise |
| FBXW9 | brown |
| FCER1A | black |
| FCER1G | brown |
| FCGBP | blue |
| FCGR1A | brown |
| FCGR1B | brown |
| FCGR2A | brown |
| FCGR2B | brown |
| FCGR2C | brown |
| FCGR3A | brown |
| FCGRT | brown |
| FCHO1 | turquoise |
| FCHO2 | black |
| FCHSD1 | turquoise |
| FCHSD2 | turquoise |
| FCN1 | turquoise |
| FCRL5 | turquoise |
| FCRLB | brown |
| FDFT1 | brown |
| FDPS | lightgreen |
| FDPSL2A | blue |
| FDX1L | turquoise |
| FDXR | grey60 |
| FECH | red |
| FEM1B | blue |
| FEM1C | blue |
| FEN1 | green |
| FER | black |
| FER1L4 | turquoise |
| FERMT1 | cyan |
| FERMT3 | turquoise |
| FERP1 | yellow |
| FES | brown |
| FEZ1 | blue |
| FEZ2 | blue |
| FGD1 | magenta |
| FGD2 | turquoise |
| FGD3 | turquoise |
| FGD4 | blue |
| FGD5 | blue |
| FGD6 | blue |
| FGF1 | blue |
| FGF11 | turquoise |
| FGF12 | tan |
| FGF13 | turquoise |
| FGF2 | blue |
| FGF7 | black |
| FGFR1 | blue |
| FGFR1OP | pink |
| FGFR1OP2 | turquoise |
| FGFR2 | brown |
| FGFR4 | blue |
| FGFRL1 | turquoise |
| FGGY | darkgreen |
| FGL2 | turquoise |
| FGR | brown |
| FH | green |
| FHAD1 | brown |
| FHDC1 | brown |
| FHIT | black |
| FHL1 | black |
| FHL2 | blue |
| FHL3 | lightyellow |
| FHOD1 | tan |
| FHOD3 | blue |
| FIBIN | purple |
| FIG4 | turquoise |
| FIGN | yellow |
| FIGNL1 | green |
| FILIP1L | blue |
| FIS1 | blue |
| FITM2 | darkgreen |
| FIZ1 | yellow |
| FJX1 | green |
| FKBP10 | lightcyan |
| FKBP11 | turquoise |
| FKBP14 | lightcyan |
| FKBP1A | brown |
| FKBP1B | blue |
| FKBP2 | blue |
| FKBP3 | blue |
| FKBP4 | cyan |
| FKBP5 | darkgreen |
| FKBP7 | blue |
| FKBP9 | lightcyan |
| FKBPL | magenta |
| FKTN | red |
| FLAD1 | lightgreen |
| FLCN | lightyellow |
| FLI1 | turquoise |
| FLII | yellow |
| FLNA | blue |
| FLNB | turquoise |
| FLNC | blue |
| FLOT1 | magenta |
| FLOT2 | turquoise |
| FLRT1 | yellow |
| FLRT2 | black |
| FLRT3 | turquoise |
| FLT1 | blue |
| FLT3LG | turquoise |
| FLT4 | black |
| FLVCR1 | green |
| FLVCR2 | brown |
| FLYWCH1 | blue |
| FLYWCH2 | turquoise |
| FMN1 | brown |
| FMNL1 | turquoise |
| FMNL2 | brown |
| FMNL3 | turquoise |
| FMO1 | black |
| FMO2 | blue |
| FMO3 | blue |
| FMO4 | blue |
| FMO5 | darkgreen |
| FMO6P | darkgreen |
| FMOD | black |
| FMR1 | pink |
| FN1 | purple |
| FN3K | magenta |
| FN3KRP | grey60 |
| FNBP1 | turquoise |
| FNBP1L | brown |
| FNDC1 | purple |
| FNDC3A | turquoise |
| FNDC3B | brown |
| FNDC4 | blue |
| FNDC5 | blue |
| FNIP2 | blue |
| FNTA | blue |
| FNTB | blue |
| FOLH1 | brown |
| FOLR1 | brown |
| FOLR2 | black |
| FOS | blue |
| FOSB | blue |
| FOSL1 | turquoise |
| FOSL2 | lightcyan |
| FOXA1 | tan |
| FOXC1 | brown |
| FOXH1 | darkred |
| FOXI1 | brown |
| FOXJ2 | yellow |
| FOXJ3 | yellow |
| FOXK1 | yellow |
| FOXK2 | grey60 |
| FOXL1 | turquoise |
| FOXM1 | cyan |
| FOXN2 | turquoise |
| FOXN3 | turquoise |
| FOXO1 | turquoise |
| FOXO3 | pink |
| FOXO3B | magenta |
| FOXO4 | yellow |
| FOXO6 | magenta |
| FOXP1 | blue |
| FOXP2 | blue |
| FOXP4 | greenyellow |
| FOXQ1 | brown |
| FOXRED1 | red |
| FOXRED2 | blue |
| FOXS1 | blue |
| FPGS | brown |
| FPR1 | brown |
| FPR3 | brown |
| FRAS1 | yellow |
| FRAT1 | turquoise |
| FRAT2 | blue |
| FREM1 | black |
| FRG1B | blue |
| FRK | brown |
| FRMD3 | blue |
| FRMD4A | yellow |
| FRMD4B | black |
| FRMD5 | green |
| FRMD6 | blue |
| FRMD8 | green |
| FRRS1 | blue |
| FRS2 | red |
| FRS3 | blue |
| FRY | blue |
| FRYL | turquoise |
| FRZB | blue |
| FSCN1 | green |
| FSD1L | yellow |
| FSIP2 | yellow |
| FST | blue |
| FSTL1 | blue |
| FSTL3 | blue |
| FSTL4 | brown |
| FTCD | darkgreen |
| FTH1 | lightcyan |
| FTHL1 | yellow |
| FTHL23 | blue |
| FTHL24 | blue |
| FTHL3 | yellow |
| FTHL4 | lightyellow |
| FTL | brown |
| FTO | blue |
| FTSJ1 | blue |
| FTSJ2 | green |
| FTSJ3 | grey60 |
| FTSJD1 | turquoise |
| FTSJD2 | magenta |
| FUCA1 | brown |
| FUCA2 | brown |
| FUK | brown |
| FUNDC1 | tan |
| FURIN | yellow |
| FUS | yellow |
| FUT1 | brown |
| FUT10 | yellow |
| FUT11 | blue |
| FUT2 | brown |
| FUT3 | tan |
| FUT4 | turquoise |
| FUT6 | brown |
| FUT8 | turquoise |
| FUZ | brown |
| FXN | yellow |
| FXR1 | green |
| FXR2 | lightgreen |
| FXYD1 | black |
| FXYD3 | brown |
| FXYD5 | blue |
| FXYD6 | blue |
| FYB | turquoise |
| FYCO1 | lightyellow |
| FYN | turquoise |
| FYTTD1 | green |
| FZD1 | turquoise |
| FZD2 | brown |
| FZD3 | brown |
| FZD4 | black |
| FZD5 | yellow |
| FZD6 | brown |
| FZD7 | brown |
| FZD8 | turquoise |
| FZD9 | turquoise |
| G0S2 | brown |
| G2E3 | red |
| G3BP2 | red |
| G6PC3 | blue |
| G6PD | tan |
| GAA | brown |
| GAB1 | turquoise |
| GAB2 | yellow |
| GAB3 | turquoise |
| GABARAPL1 | cyan |
| GABARAPL2 | blue |
| GABBR1 | yellow |
| GABPB2 | lightgreen |
| GABRE | tan |
| GABRP | purple |
| GAD1 | turquoise |
| GADD45A | blue |
| GADD45B | blue |
| GADD45G | blue |
| GADD45GIP1 | blue |
| GAK | turquoise |
| GAL | blue |
| GAL3ST4 | brown |
| GALC | brown |
| GALE | tan |
| GALK1 | grey60 |
| GALM | turquoise |
| GALNS | yellow |
| GALNT1 | purple |
| GALNT10 | blue |
| GALNT11 | lightgreen |
| GALNT12 | turquoise |
| GALNT13 | turquoise |
| GALNT14 | blue |
| GALNT2 | brown |
| GALNT3 | brown |
| GALNT5 | purple |
| GALNT6 | tan |
| GALNT7 | blue |
| GALNTL2 | blue |
| GALNTL4 | turquoise |
| GALT | turquoise |
| GAMT | turquoise |
| GAN | turquoise |
| GANC | turquoise |
| GAPDH | cyan |
| GAPDHL6 | turquoise |
| GAPDHL7 | blue |
| GAPDHP39 | turquoise |
| GAPDHP42 | turquoise |
| GAPDHP49 | darkgreen |
| GAPT | turquoise |
| GAR1 | blue |
| GARNL3 | yellow |
| GARS | green |
| GART | green |
| GAS1 | blue |
| GAS2L1 | turquoise |
| GAS2L3 | green |
| GAS5 | salmon |
| GAS6 | blue |
| GAS7 | black |
| GAS8 | brown |
| GATA3 | yellow |
| GATA6 | blue |
| GATAD1 | blue |
| GATAD2A | greenyellow |
| GATAD2B | yellow |
| GATC | blue |
| GATM | blue |
| GATS | yellow |
| GATSL3 | blue |
| GBA | lightgreen |
| GBA2 | yellow |
| GBAP | magenta |
| GBAS | brown |
| GBE1 | blue |
| GBGT1 | turquoise |
| GBP1 | turquoise |
| GBP2 | turquoise |
| GBP3 | turquoise |
| GBP4 | turquoise |
| GBP5 | turquoise |
| GCA | turquoise |
| GCAT | turquoise |
| GCC1 | blue |
| GCC2 | turquoise |
| GCDH | blue |
| GCFC1 | pink |
| GCH1 | turquoise |
| GCHFR | brown |
| GCLC | blue |
| GCLM | red |
| GCN1L1 | yellow |
| GCNT1 | blue |
| GCNT1P3 | lightcyan |
| GCNT2 | magenta |
| GCSH | turquoise |
| GDAP1 | turquoise |
| GDF15 | turquoise |
| GDI1 | yellow |
| GDI2 | blue |
| GDPD3 | blue |
| GDPD5 | yellow |
| GEM | black |
| GEMIN4 | blue |
| GEMIN6 | green |
| GEMIN8 | brown |
| GEN1 | red |
| GET4 | turquoise |
| GFER | blue |
| GFI1 | turquoise |
| GFM1 | green |
| GFOD1 | yellow |
| GFOD2 | brown |
| GFPT1 | red |
| GFPT2 | blue |
| GFRA1 | blue |
| GGA1 | yellow |
| GGA2 | turquoise |
| GGA3 | yellow |
| GGCT | turquoise |
| GGCX | turquoise |
| GGH | green |
| GGN | brown |
| GGPS1 | green |
| GGT1 | tan |
| GGT5 | black |
| GGT6 | brown |
| GGT7 | blue |
| GGTA1 | black |
| GHDC | turquoise |
| GHITM | midnightblue |
| GHR | blue |
| GHRL | turquoise |
| GHRLOS | turquoise |
| GIGYF1 | yellow |
| GIMAP1 | turquoise |
| GIMAP2 | turquoise |
| GIMAP4 | turquoise |
| GIMAP5 | turquoise |
| GIMAP6 | turquoise |
| GIMAP7 | turquoise |
| GIMAP8 | turquoise |
| GIN1 | turquoise |
| GINS1 | green |
| GINS2 | green |
| GINS3 | green |
| GINS4 | green |
| GIPC1 | turquoise |
| GIT1 | magenta |
| GIT2 | turquoise |
| GJA1 | blue |
| GJA4 | black |
| GJA5 | blue |
| GJB2 | turquoise |
| GJB3 | blue |
| GJB5 | blue |
| GJC1 | blue |
| GJC2 | yellow |
| GJD3 | yellow |
| GK | brown |
| GK5 | brown |
| GKAP1 | pink |
| GLA | brown |
| GLB1 | brown |
| GLB1L | blue |
| GLB1L2 | brown |
| GLCCI1 | turquoise |
| GLCE | turquoise |
| GLG1 | blue |
| GLI2 | blue |
| GLI3 | lightyellow |
| GLI4 | darkred |
| GLIPR1 | brown |
| GLIPR1L2 | blue |
| GLIPR2 | brown |
| GLIS2 | purple |
| GLIS3 | purple |
| GLMN | pink |
| GLO1 | turquoise |
| GLOD4 | green |
| GLRB | turquoise |
| GLRX | brown |
| GLRX2 | blue |
| GLRX3 | blue |
| GLRX5 | blue |
| GLS | blue |
| GLS2 | pink |
| GLT25D1 | blue |
| GLT8D2 | purple |
| GLT8D4 | blue |
| GLTP | turquoise |
| GLTPD1 | blue |
| GLTSCR1 | yellow |
| GLTSCR2 | salmon |
| GLUD1 | red |
| GLUL | brown |
| GLYATL1 | tan |
| GLYATL2 | turquoise |
| GLYCTK | turquoise |
| GM2A | brown |
| GMCL1 | red |
| GMDS | brown |
| GMFB | greenyellow |
| GMFG | turquoise |
| GMIP | turquoise |
| GMNN | green |
| GMPPB | turquoise |
| GMPR | turquoise |
| GMPS | green |
| GNA11 | turquoise |
| GNA12 | yellow |
| GNA13 | brown |
| GNA15 | brown |
| GNAI1 | blue |
| GNAI2 | brown |
| GNAI3 | red |
| GNAL | blue |
| GNAQ | blue |
| GNAS | brown |
| GNAZ | brown |
| GNB1 | yellow |
| GNB1L | blue |
| GNB2 | yellow |
| GNB2L1 | salmon |
| GNB3 | cyan |
| GNB4 | green |
| GNB5 | blue |
| GNE | purple |
| GNG10 | brown |
| GNG11 | black |
| GNG12 | blue |
| GNG2 | turquoise |
| GNG5 | brown |
| GNG7 | turquoise |
| GNL1 | magenta |
| GNL2 | green |
| GNL3L | magenta |
| GNLY | turquoise |
| GNMT | tan |
| GNPAT | green |
| GNPDA2 | blue |
| GNPNAT1 | turquoise |
| GNRH1 | blue |
| GNRHR | brown |
| GNRHR2 | darkgrey |
| GNS | brown |
| GOLGA1 | blue |
| GOLGA2 | yellow |
| GOLGA2B | yellow |
| GOLGA4 | turquoise |
| GOLGA6L3 | magenta |
| GOLGA6L4 | magenta |
| GOLGA7 | turquoise |
| GOLGA7B | blue |
| GOLGA8A | blue |
| GOLGA8B | turquoise |
| GOLGA9P | black |
| GOLIM4 | lightcyan |
| GOLM1 | purple |
| GOLPH3 | turquoise |
| GOLPH3L | lightgreen |
| GOLT1A | brown |
| GOLT1B | cyan |
| GOPC | pink |
| GORAB | red |
| GOT1 | turquoise |
| GOT2 | turquoise |
| GPAA1 | darkred |
| GPAM | black |
| GPAT2 | blue |
| GPATCH2 | red |
| GPATCH4 | lightgreen |
| GPATCH8 | yellow |
| GPBP1 | turquoise |
| GPC1 | purple |
| GPC2 | magenta |
| GPC4 | blue |
| GPC6 | purple |
| GPCPD1 | brown |
| GPD1 | black |
| GPD1L | tan |
| GPD2 | red |
| GPER | yellow |
| GPHN | blue |
| GPI | turquoise |
| GPLD1 | magenta |
| GPM6B | brown |
| GPN1 | blue |
| GPNMB | brown |
| GPR1 | purple |
| GPR110 | brown |
| GPR116 | black |
| GPR124 | blue |
| GPR125 | brown |
| GPR126 | blue |
| GPR132 | turquoise |
| GPR137 | yellow |
| GPR137B | brown |
| GPR141 | brown |
| GPR143 | blue |
| GPR146 | yellow |
| GPR153 | blue |
| GPR155 | turquoise |
| GPR157 | brown |
| GPR160 | blue |
| GPR161 | brown |
| GPR162 | blue |
| GPR172A | darkred |
| GPR172B | blue |
| GPR173 | yellow |
| GPR176 | blue |
| GPR177 | blue |
| GPR18 | turquoise |
| GPR180 | blue |
| GPR183 | turquoise |
| GPR21 | turquoise |
| GPR34 | brown |
| GPR35 | turquoise |
| GPR37 | turquoise |
| GPR39 | turquoise |
| GPR52 | turquoise |
| GPR56 | turquoise |
| GPR63 | cyan |
| GPR64 | blue |
| GPR65 | turquoise |
| GPR68 | brown |
| GPR79 | turquoise |
| GPR81 | blue |
| GPR82 | turquoise |
| GPR84 | brown |
| GPR89A | blue |
| GPR89C | blue |
| GPR98 | blue |
| GPRASP1 | black |
| GPRASP2 | blue |
| GPRC5A | turquoise |
| GPRC5B | brown |
| GPRC5C | blue |
| GPRC5D | cyan |
| GPRIN1 | black |
| GPRIN2 | magenta |
| GPRIN3 | turquoise |
| GPS1 | grey60 |
| GPS2 | turquoise |
| GPSM1 | yellow |
| GPSM2 | green |
| GPSM3 | turquoise |
| GPT | blue |
| GPT2 | turquoise |
| GPX1 | brown |
| GPX3 | black |
| GPX4 | turquoise |
| GPX7 | turquoise |
| GPX8 | purple |
| GRAMD1A | yellow |
| GRAMD1C | blue |
| GRAMD2 | turquoise |
| GRAMD3 | blue |
| GRAMD4 | greenyellow |
| GRAP | turquoise |
| GRAP2 | turquoise |
| GRASP | black |
| GRB10 | turquoise |
| GRB14 | brown |
| GRB2 | brown |
| GRB7 | brown |
| GREB1 | darkturquoise |
| GREM1 | blue |
| GRHL1 | brown |
| GRHL2 | brown |
| GRHL3 | brown |
| GRHPR | brown |
| GRIA3 | blue |
| GRID1 | black |
| GRINA | darkred |
| GRINL1A | blue |
| GRIP1 | brown |
| GRIPAP1 | yellow |
| GRK4 | blue |
| GRK5 | turquoise |
| GRK6 | turquoise |
| GRLF1 | yellow |
| GRN | brown |
| GRPEL1 | blue |
| GRSF1 | red |
| GRTP1 | brown |
| GS1-115G20.1 | blue |
| GS1-124K5.11 | yellow |
| GS1-124K5.2 | blue |
| GS1-124K5.3 | yellow |
| GS1-124K5.4 | turquoise |
| GS1-251I9.4 | midnightblue |
| GS1-526D21.5 | purple |
| GSC | blue |
| GSDMB | turquoise |
| GSDMC | blue |
| GSDMD | turquoise |
| GSK3A | yellow |
| GSK3B | yellow |
| GSN | blue |
| GSPT1 | turquoise |
| GSR | tan |
| GSS | turquoise |
| GSTA4 | magenta |
| GSTCD | red |
| GSTK1 | turquoise |
| GSTM2 | blue |
| GSTM3 | turquoise |
| GSTM4 | blue |
| GSTO1 | brown |
| GSTO2 | brown |
| GSTP1 | blue |
| GSTZ1 | turquoise |
| GTDC1 | turquoise |
| GTF2A2 | blue |
| GTF2E1 | yellow |
| GTF2E2 | green |
| GTF2F2 | red |
| GTF2H2 | blue |
| GTF2H2B | blue |
| GTF2H2C | blue |
| GTF2H3 | green |
| GTF2H4 | magenta |
| GTF2H5 | yellow |
| GTF2I | brown |
| GTF2IRD1 | turquoise |
| GTF2IRD2 | blue |
| GTF2IRD2B | blue |
| GTF3A | blue |
| GTF3C2 | magenta |
| GTF3C4 | brown |
| GTF3C5 | blue |
| GTF3C6 | green |
| GTPBP1 | turquoise |
| GTPBP10 | brown |
| GTPBP2 | magenta |
| GTPBP3 | blue |
| GTPBP4 | green |
| GTPBP5 | blue |
| GTPBP6 | blue |
| GTSE1 | green |
| GUCA1B | turquoise |
| GUCY1A2 | blue |
| GUCY1A3 | blue |
| GUCY1B3 | blue |
| GUF1 | red |
| GUK1 | blue |
| GULP1 | blue |
| GUSB | darkgreen |
| GUSBL1 | blue |
| GUSBL2 | magenta |
| GUSBP1 | yellow |
| GYG1 | blue |
| GYG2 | blue |
| GYLTL1B | blue |
| GYPC | turquoise |
| GYS1 | yellow |
| GZF1 | blue |
| GZMA | turquoise |
| GZMB | turquoise |
| GZMH | turquoise |
| GZMK | turquoise |
| H19 | black |
| H1F0 | brown |
| H1FX | turquoise |
| H2AFJ | turquoise |
| H2AFV | blue |
| H2AFX | green |
| H2AFY2 | magenta |
| H2AFZ | darkturquoise |
| H2AFZP3 | turquoise |
| H3F3A | green |
| H3F3B | grey60 |
| H6PD | blue |
| HAAO | darkgreen |
| HABP4 | lightyellow |
| HACE1 | red |
| HACL1 | blue |
| HADH | yellow |
| HAGHL | blue |
| HAMP | brown |
| HAPLN3 | blue |
| HAS2 | blue |
| HAS2AS | brown |
| HAS3 | blue |
| HAUS1 | red |
| HAUS4 | brown |
| HAUS5 | turquoise |
| HAUS6 | green |
| HAUS7 | green |
| HAUS8 | green |
| HAVCR2 | brown |
| HAX1 | lightgreen |
| HBA1 | black |
| HBA2 | black |
| HBB | black |
| HBEGF | brown |
| HBS1L | red |
| HBXIP | blue |
| HBXIPL | brown |
| HCCS | blue |
| HCFC1 | yellow |
| HCFC1R1 | lightcyan |
| HCG11 | pink |
| HCG18 | magenta |
| HCG25 | magenta |
| HCG27 | turquoise |
| HCG4P7 | turquoise |
| HCK | brown |
| HCLS1 | turquoise |
| HCN3 | magenta |
| HCP5 | turquoise |
| HCST | turquoise |
| HDAC1 | blue |
| HDAC10 | turquoise |
| HDAC11 | brown |
| HDAC2 | red |
| HDAC4 | turquoise |
| HDAC5 | yellow |
| HDAC6 | yellow |
| HDAC7 | blue |
| HDAC9 | turquoise |
| HDDC2 | red |
| HDDC3 | blue |
| HDGF | lightgreen |
| HDHD1A | turquoise |
| HDHD2 | red |
| HDHD3 | brown |
| HDLBP | turquoise |
| HDX | blue |
| HEATR1 | green |
| HEATR2 | turquoise |
| HEATR5A | red |
| HEATR6 | grey60 |
| HEATR7A | darkred |
| HEBP1 | cyan |
| HEBP2 | turquoise |
| HECA | turquoise |
| HECTD1 | red |
| HECTD2 | blue |
| HECTD3 | yellow |
| HECW1 | lightcyan |
| HECW2 | blue |
| HEG1 | purple |
| HELB | turquoise |
| HELLS | green |
| HELQ | turquoise |
| HEMK1 | blue |
| HEPH | blue |
| HERC2P3 | blue |
| HERC2P9 | blue |
| HERC3 | darkgreen |
| HERC4 | turquoise |
| HERC5 | turquoise |
| HERC6 | turquoise |
| HERPUD1 | turquoise |
| HERPUD2 | turquoise |
| HES1 | turquoise |
| HES2 | purple |
| HES4 | brown |
| HES6 | blue |
| HEXA | brown |
| HEXB | brown |
| HEXDC | turquoise |
| HEXIM1 | blue |
| HEXIM2 | yellow |
| HEY1 | blue |
| HEY2 | turquoise |
| HEYL | blue |
| HFE | blue |
| HGD | tan |
| HGF | black |
| HGS | grey60 |
| HGSNAT | yellow |
| HHAT | blue |
| HHEX | turquoise |
| HHIPL1 | blue |
| HHLA3 | greenyellow |
| HIATL1 | red |
| HIATL2 | yellow |
| HIBADH | turquoise |
| HIBCH | pink |
| HIC1 | blue |
| HIF1A | lightcyan |
| HIGD1A | turquoise |
| HIGD1B | black |
| HIGD2A | brown |
| HINT1 | blue |
| HINT2 | blue |
| HINT3 | red |
| HIP1 | blue |
| HIP1R | magenta |
| HIPK2 | yellow |
| HIPK3 | red |
| HIST1H1A | green |
| HIST1H1B | darkturquoise |
| HIST1H1C | darkturquoise |
| HIST1H1D | darkturquoise |
| HIST1H1E | darkturquoise |
| HIST1H2AB | darkturquoise |
| HIST1H2AC | darkturquoise |
| HIST1H2AD | blue |
| HIST1H2AE | darkturquoise |
| HIST1H2AH | darkturquoise |
| HIST1H2AI | darkturquoise |
| HIST1H2AJ | darkturquoise |
| HIST1H2AK | darkturquoise |
| HIST1H2AL | darkturquoise |
| HIST1H2AM | darkturquoise |
| HIST1H2BB | darkturquoise |
| HIST1H2BC | darkturquoise |
| HIST1H2BD | darkturquoise |
| HIST1H2BE | salmon |
| HIST1H2BF | green |
| HIST1H2BG | darkturquoise |
| HIST1H2BH | darkturquoise |
| HIST1H2BI | darkturquoise |
| HIST1H2BJ | darkturquoise |
| HIST1H2BK | darkturquoise |
| HIST1H2BL | darkturquoise |
| HIST1H2BM | darkturquoise |
| HIST1H2BN | green |
| HIST1H2BO | darkturquoise |
| HIST1H3A | darkturquoise |
| HIST1H3B | green |
| HIST1H3C | darkturquoise |
| HIST1H3D | darkturquoise |
| HIST1H3E | blue |
| HIST1H3F | darkturquoise |
| HIST1H3G | darkturquoise |
| HIST1H3H | darkturquoise |
| HIST1H3I | darkturquoise |
| HIST1H3J | darkturquoise |
| HIST1H4A | darkturquoise |
| HIST1H4B | darkturquoise |
| HIST1H4C | darkturquoise |
| HIST1H4D | salmon |
| HIST1H4E | magenta |
| HIST1H4F | yellow |
| HIST1H4H | darkturquoise |
| HIST1H4I | turquoise |
| HIST1H4J | salmon |
| HIST1H4L | yellow |
| HIST2H2AA3 | darkgrey |
| HIST2H2AA4 | blue |
| HIST2H2AB | darkturquoise |
| HIST2H2AC | darkturquoise |
| HIST2H2BB | blue |
| HIST2H2BE | green |
| HIST2H2BF | darkturquoise |
| HIST2H3C | royalblue |
| HIST2H3D | blue |
| HIST2H3PS2 | brown |
| HIST2H4B | royalblue |
| HIST3H2A | darkturquoise |
| HIST3H2BB | darkturquoise |
| HIST4H4 | cyan |
| HIVEP1 | yellow |
| HIVEP2 | blue |
| HIVEP3 | yellow |
| HJURP | green |
| HK1 | yellow |
| HK2 | turquoise |
| HK3 | brown |
| HKR1 | pink |
| HLA-A | turquoise |
| HLA-B | turquoise |
| HLA-C | turquoise |
| HLA-DMA | turquoise |
| HLA-DMB | turquoise |
| HLA-DOA | turquoise |
| HLA-DOB | turquoise |
| HLA-DPA1 | turquoise |
| HLA-DPB1 | turquoise |
| HLA-DQA1 | turquoise |
| HLA-DQA2 | turquoise |
| HLA-DQB1 | turquoise |
| HLA-DQB2 | turquoise |
| HLA-DRA | turquoise |
| HLA-DRB1 | turquoise |
| HLA-DRB5 | turquoise |
| HLA-DRB6 | turquoise |
| HLA-E | turquoise |
| HLA-F | turquoise |
| HLA-H | turquoise |
| HLA-K | turquoise |
| HLCS | brown |
| HLTF | brown |
| HLX | black |
| HM13 | yellow |
| HMBOX1 | blue |
| HMBS | blue |
| HMCN1 | blue |
| HMCN2 | turquoise |
| HMG20B | brown |
| HMGA1 | green |
| HMGA1L2 | turquoise |
| HMGB1L14 | turquoise |
| HMGB2 | green |
| HMGB3 | turquoise |
| HMGCL | blue |
| HMGCR | tan |
| HMGCS1 | turquoise |
| HMGN1 | blue |
| HMGN1L2 | yellow |
| HMGN2 | brown |
| HMGN3 | red |
| HMGN4 | blue |
| HMGXB4 | darkred |
| HMHA1 | turquoise |
| HMMR | green |
| HMOX1 | brown |
| HMOX2 | tan |
| HN1 | grey60 |
| HN1L | turquoise |
| HNMT | brown |
| HNRNPA2B1 | yellow |
| HNRNPA3 | yellow |
| HNRNPAB | blue |
| HNRNPD | yellow |
| HNRNPF | green |
| HNRNPH1 | turquoise |
| HNRNPH3 | yellow |
| HNRNPUL1 | yellow |
| HNRNPUL2 | greenyellow |
| HNRPLL | pink |
| HOMER1 | turquoise |
| HOMER2 | brown |
| HOMER3 | turquoise |
| HOMEZ | yellow |
| HOOK1 | brown |
| HOOK2 | brown |
| HOOK3 | blue |
| HOPX | blue |
| HORMAD1 | blue |
| HOTAIR | brown |
| HOTAIRM1 | blue |
| HOXA3 | blue |
| HOXA5 | blue |
| HOXA7 | blue |
| HOXA9 | blue |
| HOXB2 | blue |
| HOXB3 | blue |
| HOXB4 | black |
| HOXB6 | blue |
| HOXB7 | blue |
| HOXC10 | brown |
| HOXC4 | lightyellow |
| HOXC6 | lightyellow |
| HOXC9 | yellow |
| HOXD3 | black |
| HOXD8 | black |
| HOXD9 | black |
| HPCAL1 | brown |
| HPDL | blue |
| HPGD | darkgreen |
| HPN | brown |
| HPRT1 | green |
| HPS4 | blue |
| HPS5 | turquoise |
| HPSE | brown |
| HR | brown |
| HRAS | turquoise |
| HRASLS | blue |
| HRCT1 | brown |
| HRH1 | blue |
| HRSP12 | blue |
| HS1BP3 | yellow |
| HS2ST1 | blue |
| HS3ST1 | brown |
| HS3ST2 | brown |
| HS3ST3A1 | brown |
| HS3ST3B1 | turquoise |
| HS6ST1 | brown |
| hsa-mir-663 | red |
| hsa-mir-663b | red |
| HSBP1 | turquoise |
| HSBP1L1 | turquoise |
| HSCB | blue |
| HSD11B1L | turquoise |
| HSD11B2 | brown |
| HSD17B1 | yellow |
| HSD17B10 | blue |
| HSD17B11 | brown |
| HSD17B12 | blue |
| HSD17B14 | brown |
| HSD17B2 | brown |
| HSD17B4 | turquoise |
| HSD17B6 | lightcyan |
| HSD17B7 | brown |
| HSD17B8 | turquoise |
| HSD3B7 | brown |
| HSDL1 | turquoise |
| HSDL2 | blue |
| HSF1 | darkred |
| HSF2 | red |
| HSF2BP | brown |
| HSF4 | brown |
| HSH2D | turquoise |
| HSP90AA1 | turquoise |
| HSP90AB1 | magenta |
| HSP90B1 | brown |
| HSPA12A | blue |
| HSPA12B | black |
| HSPA13 | turquoise |
| HSPA14 | blue |
| HSPA1A | turquoise |
| HSPA1B | turquoise |
| HSPA2 | turquoise |
| HSPA4L | brown |
| HSPA5 | yellow |
| HSPA6 | brown |
| HSPA7 | brown |
| HSPB1 | purple |
| HSPB11 | turquoise |
| HSPB2 | blue |
| HSPB6 | black |
| HSPB7 | blue |
| HSPB8 | blue |
| HSPBAP1 | turquoise |
| HSPBP1 | green |
| HSPD1 | blue |
| HSPD1P11 | blue |
| HSPE1 | blue |
| HSPG2 | blue |
| HSPH1 | turquoise |
| HTATIP2 | blue |
| HTATSF1 | green |
| HTRA1 | purple |
| HTRA3 | purple |
| HTT | turquoise |
| HUWE1 | magenta |
| HVCN1 | turquoise |
| HYAL1 | turquoise |
| HYAL2 | brown |
| HYAL3 | turquoise |
| HYDIN | blue |
| HYI | yellow |
| HYLS1 | green |
| HYOU1 | blue |
| HYPK | turquoise |
| IAH1 | green |
| IARS | green |
| IARS2 | green |
| ICA1 | turquoise |
| ICA1L | blue |
| ICAM1 | brown |
| ICAM2 | turquoise |
| ICAM3 | turquoise |
| ICK | magenta |
| ICMT | turquoise |
| ICOSLG | yellow |
| ICT1 | grey60 |
| ID1 | blue |
| ID2 | brown |
| ID3 | blue |
| ID4 | brown |
| IDH1 | tan |
| IDH2 | blue |
| IDH3A | brown |
| IDH3B | blue |
| IDI1 | darkgreen |
| IDI2 | blue |
| IDO1 | turquoise |
| IDS | black |
| IDUA | turquoise |
| IER2 | brown |
| IER3 | turquoise |
| IER3IP1 | red |
| IER5 | turquoise |
| IER5L | turquoise |
| IFFO1 | turquoise |
| IFFO2 | yellow |
| IFI16 | turquoise |
| IFI27 | turquoise |
| IFI27L1 | yellow |
| IFI27L2 | brown |
| IFI30 | brown |
| IFI35 | turquoise |
| IFI44 | turquoise |
| IFI44L | turquoise |
| IFI6 | turquoise |
| IFIH1 | turquoise |
| IFIT1 | turquoise |
| IFIT2 | turquoise |
| IFIT3 | turquoise |
| IFIT5 | turquoise |
| IFITM1 | turquoise |
| IFITM2 | brown |
| IFITM3 | brown |
| IFNAR1 | brown |
| IFNAR2 | turquoise |
| IFNGR1 | brown |
| IFNGR2 | brown |
| IFRD1 | turquoise |
| IFRD2 | blue |
| IFT122 | yellow |
| IFT140 | yellow |
| IFT172 | red |
| IFT46 | blue |
| IFT52 | brown |
| IFT57 | turquoise |
| IFT74 | black |
| IFT80 | blue |
| IFT81 | pink |
| IFT88 | blue |
| IGBP1 | blue |
| IGF1 | black |
| IGF1R | yellow |
| IGF2 | black |
| IGF2BP2 | brown |
| IGF2BP3 | brown |
| IGF2R | brown |
| IGFBP2 | blue |
| IGFBP3 | lightcyan |
| IGFBP4 | blue |
| IGFBP5 | lightyellow |
| IGFBP6 | black |
| IGFBP7 | blue |
| IGHA1 | turquoise |
| IGHA2 | turquoise |
| IGHG1 | turquoise |
| IGHG2 | turquoise |
| IGHG3 | turquoise |
| IGHG4 | turquoise |
| IGHM | turquoise |
| IGHMBP2 | yellow |
| IGHV1-18 | turquoise |
| IGHV1-24 | turquoise |
| IGHV1-3 | turquoise |
| IGHV1-46 | turquoise |
| IGHV1-69 | turquoise |
| IGHV1-8 | turquoise |
| IGHV2-5 | turquoise |
| IGHV2-70 | turquoise |
| IGHV3-11 | turquoise |
| IGHV3-13 | turquoise |
| IGHV3-15 | turquoise |
| IGHV3-20 | turquoise |
| IGHV3-21 | turquoise |
| IGHV3-23 | turquoise |
| IGHV3-30 | turquoise |
| IGHV3-33 | turquoise |
| IGHV3-43 | turquoise |
| IGHV3-48 | turquoise |
| IGHV3-49 | turquoise |
| IGHV3-53 | turquoise |
| IGHV3-66 | turquoise |
| IGHV3-7 | turquoise |
| IGHV3-72 | turquoise |
| IGHV3-73 | turquoise |
| IGHV3-74 | turquoise |
| IGHV3-9 | turquoise |
| IGHV4-31 | turquoise |
| IGHV4-34 | turquoise |
| IGHV4-39 | turquoise |
| IGHV4-4 | turquoise |
| IGHV4-59 | turquoise |
| IGHV4-61 | turquoise |
| IGHV5-51 | turquoise |
| IGHV6-1 | turquoise |
| IGJ | turquoise |
| IGKC | turquoise |
| IGKV1-12 | turquoise |
| IGKV1-16 | turquoise |
| IGKV1-17 | turquoise |
| IGKV1-27 | turquoise |
| IGKV1-5 | turquoise |
| IGKV1-6 | turquoise |
| IGKV1-9 | turquoise |
| IGKV1D-12 | turquoise |
| IGKV1D-13 | turquoise |
| IGKV2-24 | turquoise |
| IGKV2-30 | turquoise |
| IGKV3-11 | turquoise |
| IGKV3-15 | turquoise |
| IGKV3-20 | turquoise |
| IGKV4-1 | turquoise |
| IGKV6-21 | turquoise |
| IGLC1 | turquoise |
| IGLC2 | turquoise |
| IGLC3 | turquoise |
| IGLC7 | turquoise |
| IGLV1-40 | turquoise |
| IGLV1-44 | turquoise |
| IGLV1-47 | turquoise |
| IGLV1-51 | turquoise |
| IGLV10-54 | turquoise |
| IGLV2-11 | turquoise |
| IGLV2-14 | turquoise |
| IGLV2-18 | turquoise |
| IGLV2-23 | turquoise |
| IGLV2-8 | turquoise |
| IGLV3-1 | turquoise |
| IGLV3-10 | turquoise |
| IGLV3-19 | turquoise |
| IGLV3-21 | turquoise |
| IGLV3-25 | turquoise |
| IGLV3-27 | turquoise |
| IGLV4-69 | turquoise |
| IGLV5-45 | turquoise |
| IGLV6-57 | turquoise |
| IGLV7-43 | turquoise |
| IGLV7-46 | turquoise |
| IGLV8-61 | turquoise |
| IGLV9-49 | turquoise |
| IGSF10 | blue |
| IGSF22 | tan |
| IGSF3 | brown |
| IGSF6 | turquoise |
| IGSF8 | brown |
| IGSF9 | lightgreen |
| IKBIP | brown |
| IKBKB | yellow |
| IKBKE | turquoise |
| IKBKG | turquoise |
| IKZF1 | turquoise |
| IKZF2 | turquoise |
| IKZF3 | turquoise |
| IKZF5 | blue |
| IL10 | brown |
| IL10RA | turquoise |
| IL10RB | brown |
| IL11RA | black |
| IL12RB1 | turquoise |
| IL12RB2 | blue |
| IL13RA1 | lightcyan |
| IL15 | turquoise |
| IL15RA | turquoise |
| IL16 | turquoise |
| IL17D | turquoise |
| IL17RB | brown |
| IL17RC | brown |
| IL17RD | magenta |
| IL17RE | brown |
| IL18 | brown |
| IL18BP | turquoise |
| IL18R1 | turquoise |
| IL1B | brown |
| IL1R1 | black |
| IL1R2 | turquoise |
| IL1RAP | lightcyan |
| IL1RL2 | brown |
| IL1RN | brown |
| IL20RA | brown |
| IL20RB | green |
| IL21R | turquoise |
| IL23A | turquoise |
| IL27RA | turquoise |
| IL28RA | turquoise |
| IL2RA | turquoise |
| IL2RB | turquoise |
| IL2RG | turquoise |
| IL32 | turquoise |
| IL33 | black |
| IL34 | blue |
| IL3RA | turquoise |
| IL4I1 | brown |
| IL4R | turquoise |
| IL6 | blue |
| IL6R | turquoise |
| IL6ST | black |
| IL7 | turquoise |
| IL7R | turquoise |
| IL8 | turquoise |
| ILDR1 | brown |
| ILF2 | lightgreen |
| ILF3 | greenyellow |
| ILVBL | turquoise |
| IMMP1L | brown |
| IMMP2L | brown |
| IMP3 | blue |
| IMP4 | blue |
| IMPA1 | turquoise |
| IMPA2 | blue |
| IMPACT | red |
| IMPAD1 | midnightblue |
| IMPDH1 | blue |
| IMPDH2 | salmon |
| INADL | brown |
| INCENP | green |
| INE1 | yellow |
| INF2 | yellow |
| ING1 | blue |
| ING2 | greenyellow |
| ING3 | turquoise |
| ING4 | cyan |
| ING5 | yellow |
| INHBA | lightcyan |
| INHBB | blue |
| INMT | black |
| INO80B | brown |
| INO80C | blue |
| INO80D | yellow |
| INPP1 | black |
| INPP4A | turquoise |
| INPP4B | black |
| INPP5A | brown |
| INPP5B | turquoise |
| INPP5D | turquoise |
| INPP5E | yellow |
| INPP5F | turquoise |
| INPP5K | yellow |
| INPPL1 | yellow |
| INSIG1 | turquoise |
| INSIG2 | purple |
| INSR | brown |
| INTS1 | yellow |
| INTS10 | blue |
| INTS2 | grey60 |
| INTS3 | yellow |
| INTS4 | yellow |
| INTS7 | green |
| INTS8 | midnightblue |
| INTS9 | turquoise |
| INTU | blue |
| INVS | turquoise |
| IP6K2 | yellow |
| IPCEF1 | turquoise |
| IPMK | red |
| IPO13 | green |
| IPO4 | blue |
| IPO5 | turquoise |
| IPO7 | red |
| IPO8 | cyan |
| IPO9 | magenta |
| IPP | brown |
| IPPK | turquoise |
| IQCB1 | turquoise |
| IQCE | turquoise |
| IQCG | blue |
| IQCK | brown |
| IQGAP1 | blue |
| IQGAP2 | turquoise |
| IQGAP3 | green |
| IQSEC1 | turquoise |
| IQSEC2 | yellow |
| IRAK1 | blue |
| IRAK1BP1 | brown |
| IRAK2 | brown |
| IRAK3 | brown |
| IRAK4 | turquoise |
| IRF1 | turquoise |
| IRF2 | turquoise |
| IRF2BP1 | blue |
| IRF2BP2 | magenta |
| IRF3 | turquoise |
| IRF4 | turquoise |
| IRF5 | turquoise |
| IRF6 | brown |
| IRF7 | turquoise |
| IRF8 | turquoise |
| IRF9 | turquoise |
| IRGQ | yellow |
| IRS1 | blue |
| IRS2 | blue |
| IRX1 | brown |
| IRX2 | brown |
| IRX3 | turquoise |
| IRX5 | brown |
| ISG15 | turquoise |
| ISG20 | turquoise |
| ISG20L2 | lightgreen |
| ISLR | blue |
| ISOC1 | blue |
| ISOC2 | blue |
| ISPD | blue |
| ISYNA1 | brown |
| ITCH | royalblue |
| ITFG1 | blue |
| ITFG2 | cyan |
| ITFG3 | yellow |
| ITGA1 | blue |
| ITGA10 | blue |
| ITGA11 | purple |
| ITGA2 | turquoise |
| ITGA3 | blue |
| ITGA4 | turquoise |
| ITGA5 | purple |
| ITGA6 | blue |
| ITGA7 | black |
| ITGA9 | blue |
| ITGAE | turquoise |
| ITGAL | turquoise |
| ITGAM | brown |
| ITGAV | lightcyan |
| ITGAX | brown |
| ITGB1 | lightcyan |
| ITGB1BP1 | blue |
| ITGB1BP2 | yellow |
| ITGB2 | turquoise |
| ITGB3 | blue |
| ITGB3BP | yellow |
| ITGB4 | grey60 |
| ITGB5 | purple |
| ITGB6 | turquoise |
| ITGB7 | turquoise |
| ITGB8 | brown |
| ITGBL1 | blue |
| ITIH4 | turquoise |
| ITIH5 | black |
| ITK | turquoise |
| ITM2A | turquoise |
| ITM2B | black |
| ITM2C | turquoise |
| ITPA | blue |
| ITPK1 | brown |
| ITPKB | turquoise |
| ITPKC | blue |
| ITPR1 | turquoise |
| ITPR2 | turquoise |
| ITPR3 | magenta |
| ITPRIP | lightyellow |
| ITPRIPL1 | turquoise |
| ITPRIPL2 | lightcyan |
| ITSN1 | black |
| ITSN2 | turquoise |
| IVD | blue |
| IVNS1ABP | red |
| J01415.12 | red |
| J01415.5 | greenyellow |
| JAG1 | blue |
| JAG2 | yellow |
| JAGN1 | turquoise |
| JAK1 | turquoise |
| JAK2 | turquoise |
| JAK3 | turquoise |
| JAM2 | black |
| JAM3 | blue |
| JARID2 | magenta |
| JAZF1 | black |
| JDP2 | blue |
| JHDM1D | yellow |
| JMJD1C | blue |
| JMJD4 | green |
| JMJD6 | grey60 |
| JMJD7 | blue |
| JMJD7-PLA2G4B | turquoise |
| JMJD8 | turquoise |
| JMY | turquoise |
| JOSD1 | blue |
| JOSD2 | yellow |
| JPH1 | blue |
| JPH2 | blue |
| JPH3 | blue |
| JRK | darkred |
| JRKL | turquoise |
| JSRP1 | turquoise |
| JTB | lightgreen |
| JUB | turquoise |
| JUN | blue |
| JUNB | brown |
| JUND | yellow |
| JUP | brown |
| KAL1 | lightcyan |
| KALRN | magenta |
| KANK1 | brown |
| KANK2 | blue |
| KANK3 | black |
| KANK4 | brown |
| KAT2A | yellow |
| KAT2B | turquoise |
| KATNA1 | pink |
| KATNAL1 | blue |
| KATNAL2 | red |
| KAZALD1 | turquoise |
| KB-1183D5.12 | yellow |
| KB-1205A7.1 | midnightblue |
| KB-1208A12.3 | blue |
| KB-1254G8.1 | midnightblue |
| KB-1323B2.6 | turquoise |
| KB-1507C5.2 | brown |
| KB-1562D12.1 | brown |
| KB-1562D12.2 | brown |
| KB-1562D12.3 | brown |
| KB-1608C10.2 | midnightblue |
| KB-1907C4.2 | midnightblue |
| KB-318B8.7 | yellow |
| KB-431C1.4 | turquoise |
| KBTBD6 | red |
| KBTBD8 | turquoise |
| KCMF1 | green |
| KCNA3 | turquoise |
| KCNAB1 | black |
| KCNAB2 | turquoise |
| KCNC3 | yellow |
| KCNC4 | yellow |
| KCND2 | purple |
| KCND3 | blue |
| KCNE3 | brown |
| KCNE4 | blue |
| KCNG1 | turquoise |
| KCNG2 | brown |
| KCNH2 | blue |
| KCNIP2 | black |
| KCNIP3 | yellow |
| KCNJ14 | yellow |
| KCNJ15 | purple |
| KCNJ2 | brown |
| KCNJ5 | brown |
| KCNJ8 | black |
| KCNK1 | turquoise |
| KCNK12 | brown |
| KCNK13 | brown |
| KCNK15 | blue |
| KCNK5 | brown |
| KCNK6 | brown |
| KCNMA1 | brown |
| KCNMB1 | blue |
| KCNMB3 | yellow |
| KCNMB4 | blue |
| KCNN4 | brown |
| KCNQ1 | blue |
| KCNQ3 | turquoise |
| KCNQ4 | magenta |
| KCNQ5 | blue |
| KCNRG | turquoise |
| KCNS3 | turquoise |
| KCNV2 | purple |
| KCTD1 | brown |
| KCTD10 | blue |
| KCTD11 | lightcyan |
| KCTD12 | brown |
| KCTD13 | yellow |
| KCTD14 | purple |
| KCTD15 | purple |
| KCTD17 | brown |
| KCTD21 | yellow |
| KCTD3 | turquoise |
| KCTD5 | blue |
| KCTD6 | blue |
| KCTD9 | turquoise |
| KDELC1 | lightcyan |
| KDELC2 | blue |
| KDELR1 | yellow |
| KDELR2 | turquoise |
| KDELR3 | turquoise |
| KDM1A | green |
| KDM1B | green |
| KDM2A | yellow |
| KDM3A | yellow |
| KDM4A | yellow |
| KDM4B | yellow |
| KDM4C | turquoise |
| KDM5A | cyan |
| KDM5B | magenta |
| KDM5C | yellow |
| KDM6A | turquoise |
| KDM6B | yellow |
| KDR | black |
| KDSR | red |
| KEAP1 | blue |
| KHDC1 | green |
| KHDRBS3 | turquoise |
| KHK | blue |
| KHSRP | greenyellow |
| KIAA0020 | blue |
| KIAA0040 | turquoise |
| KIAA0090 | red |
| KIAA0101 | green |
| KIAA0114 | blue |
| KIAA0125 | turquoise |
| KIAA0146 | yellow |
| KIAA0182 | yellow |
| KIAA0195 | yellow |
| KIAA0196 | midnightblue |
| KIAA0226 | turquoise |
| KIAA0232 | blue |
| KIAA0240 | magenta |
| KIAA0247 | blue |
| KIAA0284 | brown |
| KIAA0319L | turquoise |
| KIAA0355 | pink |
| KIAA0415 | brown |
| KIAA0427 | turquoise |
| KIAA0467 | yellow |
| KIAA0495 | yellow |
| KIAA0513 | turquoise |
| KIAA0528 | cyan |
| KIAA0556 | yellow |
| KIAA0562 | yellow |
| KIAA0564 | pink |
| KIAA0649 | brown |
| KIAA0664 | blue |
| KIAA0776 | red |
| KIAA0802 | blue |
| KIAA0895 | turquoise |
| KIAA0895L | yellow |
| KIAA0907 | lightgreen |
| KIAA0913 | lightyellow |
| KIAA0922 | turquoise |
| KIAA1147 | brown |
| KIAA1161 | turquoise |
| KIAA1191 | yellow |
| KIAA1199 | lightcyan |
| KIAA1217 | yellow |
| KIAA1244 | brown |
| KIAA1274 | turquoise |
| KIAA1324 | brown |
| KIAA1324L | blue |
| KIAA1328 | blue |
| KIAA1370 | turquoise |
| KIAA1383 | red |
| KIAA1407 | blue |
| KIAA1429 | midnightblue |
| KIAA1430 | red |
| KIAA1432 | turquoise |
| KIAA1462 | purple |
| KIAA1467 | turquoise |
| KIAA1468 | red |
| KIAA1522 | brown |
| KIAA1524 | green |
| KIAA1529 | blue |
| KIAA1530 | turquoise |
| KIAA1539 | lightcyan |
| KIAA1543 | brown |
| KIAA1549 | brown |
| KIAA1586 | turquoise |
| KIAA1598 | yellow |
| KIAA1602 | lightyellow |
| KIAA1609 | turquoise |
| KIAA1614 | blue |
| KIAA1644 | lightcyan |
| KIAA1671 | yellow |
| KIAA1683 | lightyellow |
| KIAA1712 | red |
| KIAA1715 | turquoise |
| KIAA175 | blue |
| KIAA1797 | brown |
| KIAA1826 | blue |
| KIAA1841 | red |
| KIAA1919 | blue |
| KIAA1949 | turquoise |
| KIAA1958 | yellow |
| KIAA1967 | yellow |
| KIAA1984 | yellow |
| KIAA2013 | blue |
| KIAA2018 | yellow |
| KIAA2026 | yellow |
| KIDINS220 | pink |
| KIF11 | green |
| KIF12 | brown |
| KIF13A | blue |
| KIF13B | blue |
| KIF14 | green |
| KIF15 | green |
| KIF16B | blue |
| KIF17 | turquoise |
| KIF18A | green |
| KIF18B | green |
| KIF1A | brown |
| KIF1B | red |
| KIF1C | turquoise |
| KIF20A | green |
| KIF20B | green |
| KIF21A | turquoise |
| KIF21B | turquoise |
| KIF22 | green |
| KIF23 | green |
| KIF24 | green |
| KIF26B | purple |
| KIF27 | pink |
| KIF2A | turquoise |
| KIF2C | green |
| KIF3A | yellow |
| KIF3C | magenta |
| KIF4A | green |
| KIF5B | turquoise |
| KIF7 | blue |
| KIF9 | brown |
| KIFAP3 | pink |
| KIFC1 | green |
| KIFC2 | darkred |
| KIFC3 | lightcyan |
| KIN | yellow |
| KIRREL | blue |
| KIT | blue |
| KITLG | blue |
| KLC2 | yellow |
| KLC3 | brown |
| KLC4 | magenta |
| KLF10 | blue |
| KLF11 | pink |
| KLF12 | turquoise |
| KLF13 | turquoise |
| KLF15 | black |
| KLF16 | yellow |
| KLF2 | turquoise |
| KLF3 | blue |
| KLF4 | blue |
| KLF5 | brown |
| KLF6 | blue |
| KLF7 | blue |
| KLF8 | blue |
| KLF9 | black |
| KLHDC10 | brown |
| KLHDC2 | red |
| KLHDC3 | magenta |
| KLHDC4 | yellow |
| KLHDC5 | cyan |
| KLHDC7B | turquoise |
| KLHDC8B | brown |
| KLHDC9 | brown |
| KLHL13 | brown |
| KLHL15 | red |
| KLHL17 | blue |
| KLHL18 | green |
| KLHL2 | blue |
| KLHL21 | brown |
| KLHL22 | yellow |
| KLHL23 | magenta |
| KLHL24 | brown |
| KLHL25 | magenta |
| KLHL26 | yellow |
| KLHL29 | lightyellow |
| KLHL3 | black |
| KLHL31 | turquoise |
| KLHL35 | turquoise |
| KLHL5 | turquoise |
| KLHL6 | turquoise |
| KLHL7 | turquoise |
| KLHL8 | darkgreen |
| KLHL9 | blue |
| KLK5 | brown |
| KLK6 | turquoise |
| KLK7 | brown |
| KLRAQ1 | turquoise |
| KLRB1 | turquoise |
| KLRG1 | turquoise |
| KLRG2 | green |
| KLRK1 | turquoise |
| KMO | brown |
| KNTC1 | green |
| KPNA2 | green |
| KPNA4 | green |
| KPNA5 | turquoise |
| KPTN | brown |
| KRAS | cyan |
| KRBA1 | yellow |
| KRBA2 | blue |
| KRBOX1 | brown |
| KRCC1 | red |
| KREMEN1 | yellow |
| KREMEN2 | blue |
| KRI1 | yellow |
| KRIT1 | yellow |
| KRR1 | red |
| KRT10 | turquoise |
| KRT14 | yellow |
| KRT15 | brown |
| KRT16 | turquoise |
| KRT17 | brown |
| KRT18P12 | brown |
| KRT18P15 | brown |
| KRT18P5 | yellow |
| KRT19 | turquoise |
| KRT23 | brown |
| KRT5 | yellow |
| KRT6A | turquoise |
| KRT6B | brown |
| KRT7 | turquoise |
| KRT8 | brown |
| KRT80 | turquoise |
| KRT81 | brown |
| KRT86 | brown |
| KRT8P12 | brown |
| KRTAP5-2 | brown |
| KRTCAP2 | lightgreen |
| KRTCAP3 | brown |
| KSR1 | turquoise |
| KTELC1 | turquoise |
| KTN1 | red |
| KYNU | tan |
| L1CAM | brown |
| L29074.5 | royalblue |
| L2HGDH | brown |
| L3MBTL | blue |
| L3MBTL3 | turquoise |
| L3MBTL4 | blue |
| LA16c-395F10.1 | yellow |
| LA16c-398G5.2 | blue |
| LA16c-60H5.5 | yellow |
| LA16c-60H5.7 | yellow |
| LACE1 | red |
| LACTB | brown |
| LACTB2 | midnightblue |
| LAD1 | lightgreen |
| LAG3 | turquoise |
| LAGE3 | blue |
| LAIR1 | turquoise |
| LAMA1 | black |
| LAMA2 | black |
| LAMA3 | blue |
| LAMA4 | blue |
| LAMA5 | yellow |
| LAMB1 | black |
| LAMB2 | blue |
| LAMB3 | blue |
| LAMC1 | blue |
| LAMC2 | brown |
| LAMP1 | turquoise |
| LAMP2 | turquoise |
| LAMP3 | turquoise |
| LANCL1 | pink |
| LANCL2 | brown |
| LAP3 | turquoise |
| LAPTM4A | turquoise |
| LAPTM4B | blue |
| LAPTM5 | turquoise |
| LARGE | blue |
| LARP1 | yellow |
| LARP4B | green |
| LARP6 | blue |
| LASP1 | blue |
| LASS2 | lightgreen |
| LASS4 | tan |
| LASS6 | purple |
| LAT | turquoise |
| LAT2 | turquoise |
| LATS2 | blue |
| LAX1 | turquoise |
| LAYN | black |
| LBH | turquoise |
| LBP | tan |
| LBR | red |
| LBX2 | turquoise |
| LCA5 | blue |
| LCAT | lightyellow |
| LCK | turquoise |
| LCLAT1 | brown |
| LCMT1 | red |
| LCN12 | yellow |
| LCN2 | brown |
| LCOR | turquoise |
| LCORL | turquoise |
| LCP1 | turquoise |
| LCP2 | turquoise |
| LDB1 | yellow |
| LDB2 | black |
| LDHA | turquoise |
| LDHAL2 | turquoise |
| LDHB | cyan |
| LDHD | darkgreen |
| LDLR | brown |
| LDLRAD3 | red |
| LDLRAP1 | turquoise |
| LDOC1 | magenta |
| LDOC1L | yellow |
| LEAP2 | turquoise |
| LEF1 | turquoise |
| LENEP | magenta |
| LENG8 | yellow |
| LENG9 | yellow |
| LEPR | black |
| LEPRE1 | blue |
| LEPREL1 | blue |
| LEPREL2 | lightcyan |
| LEPREL4 | lightcyan |
| LEPROT | blue |
| LEPROTL1 | turquoise |
| LETM1 | yellow |
| LFNG | turquoise |
| LGALS1 | lightcyan |
| LGALS2 | turquoise |
| LGALS3 | brown |
| LGALS3BP | brown |
| LGALS8 | blue |
| LGALS9 | turquoise |
| LGI2 | brown |
| LGI4 | black |
| LGMN | brown |
| LGR4 | red |
| LGR6 | blue |
| LGTN | brown |
| LHFP | black |
| LHFPL2 | brown |
| LHFPL5 | brown |
| LHPP | yellow |
| LIF | blue |
| LIFR | blue |
| LIG1 | green |
| LIG3 | magenta |
| LIG4 | turquoise |
| LILRA2 | brown |
| LILRA4 | turquoise |
| LILRA5 | brown |
| LILRA6 | brown |
| LILRB1 | turquoise |
| LILRB2 | brown |
| LILRB3 | brown |
| LILRB4 | turquoise |
| LILRB5 | brown |
| LIMA1 | blue |
| LIMCH1 | turquoise |
| LIMD1 | turquoise |
| LIMD2 | turquoise |
| LIME1 | turquoise |
| LIMK1 | brown |
| LIMK2 | brown |
| LIMS2 | black |
| LIN37 | brown |
| LIN7A | blue |
| LIN7B | brown |
| LIN7C | red |
| LIN9 | green |
| LINS1 | turquoise |
| LIPA | brown |
| LIPE | black |
| LIPG | turquoise |
| LIPH | brown |
| LIPT1 | turquoise |
| LIPT2 | brown |
| LITAF | brown |
| LIX1L | black |
| LL0XNC01-46H11.1 | turquoise |
| LL0YNC03-3F3.1 | turquoise |
| LLGL1 | yellow |
| LLGL2 | grey60 |
| LMAN1 | red |
| LMAN2 | tan |
| LMBR1 | brown |
| LMBRD1 | blue |
| LMBRD2 | red |
| LMCD1 | blue |
| LMF1 | yellow |
| LMF2 | brown |
| LMLN | yellow |
| LMNA | turquoise |
| LMNB1 | green |
| LMNB2 | green |
| LMO2 | turquoise |
| LMO3 | cyan |
| LMO4 | blue |
| LMO7 | purple |
| LMOD1 | black |
| LMTK2 | yellow |
| LMTK3 | blue |
| LMX1B | yellow |
| LNP1 | turquoise |
| LNPEP | turquoise |
| LNX1 | brown |
| LNX2 | brown |
| LOC134466 | blue |
| LOC147976 | brown |
| LOH12CR1 | cyan |
| LOH3CR2A | black |
| LONP1 | blue |
| LONP2 | tan |
| LONRF1 | blue |
| LOX | purple |
| LOXL1 | blue |
| LOXL2 | lightcyan |
| LOXL3 | brown |
| LOXL4 | blue |
| LPAR1 | black |
| LPAR2 | blue |
| LPAR3 | brown |
| LPAR5 | turquoise |
| LPAR6 | brown |
| LPCAT1 | yellow |
| LPCAT2 | blue |
| LPCAT3 | brown |
| LPCAT4 | turquoise |
| LPGAT1 | green |
| LPHN1 | turquoise |
| LPHN2 | blue |
| LPHN3 | blue |
| LPIN1 | yellow |
| LPIN2 | turquoise |
| LPIN3 | yellow |
| LPL | blue |
| LPP | yellow |
| LPXN | turquoise |
| LRAT | brown |
| LRBA | turquoise |
| LRCH1 | turquoise |
| LRCH3 | yellow |
| LRCH4 | turquoise |
| LRDD | yellow |
| LRFN1 | magenta |
| LRFN3 | yellow |
| LRFN4 | green |
| LRG1 | tan |
| LRIG1 | yellow |
| LRIG2 | pink |
| LRIG3 | orange |
| LRMP | turquoise |
| LRP1 | lightyellow |
| LRP10 | blue |
| LRP11 | turquoise |
| LRP12 | midnightblue |
| LRP3 | brown |
| LRP5 | magenta |
| LRP5L | yellow |
| LRP6 | cyan |
| LRP8 | green |
| LRPAP1 | brown |
| LRPPRC | green |
| LRRC1 | brown |
| LRRC10B | brown |
| LRRC14 | darkred |
| LRRC15 | purple |
| LRRC16A | magenta |
| LRRC17 | blue |
| LRRC20 | green |
| LRRC23 | cyan |
| LRRC24 | darkred |
| LRRC25 | brown |
| LRRC26 | tan |
| LRRC27 | yellow |
| LRRC28 | blue |
| LRRC29 | blue |
| LRRC3 | blue |
| LRRC32 | blue |
| LRRC33 | turquoise |
| LRRC34 | red |
| LRRC37A | turquoise |
| LRRC37A2 | turquoise |
| LRRC37A3 | blue |
| LRRC37A4 | turquoise |
| LRRC4 | yellow |
| LRRC41 | green |
| LRRC42 | green |
| LRRC45 | grey60 |
| LRRC47 | blue |
| LRRC51 | brown |
| LRRC56 | tan |
| LRRC58 | midnightblue |
| LRRC59 | grey60 |
| LRRC6 | blue |
| LRRC61 | blue |
| LRRC68 | brown |
| LRRC69 | midnightblue |
| LRRC70 | black |
| LRRC8A | blue |
| LRRC8B | green |
| LRRC8C | turquoise |
| LRRC8D | red |
| LRRC8E | turquoise |
| LRRCC1 | green |
| LRRFIP1 | blue |
| LRRFIP2 | green |
| LRRK1 | turquoise |
| LRRK2 | turquoise |
| LRRN1 | cyan |
| LRRN2 | turquoise |
| LRRN3 | black |
| LRRN4CL | black |
| LRSAM1 | yellow |
| LRWD1 | blue |
| LSG1 | green |
| LSM1 | turquoise |
| LSM10 | turquoise |
| LSM12 | royalblue |
| LSM14A | pink |
| LSM14B | brown |
| LSM2 | magenta |
| LSM3 | blue |
| LSM4 | green |
| LSM5 | turquoise |
| LSM6 | turquoise |
| LSM7 | blue |
| LSMD1 | yellow |
| LSP1 | turquoise |
| LSR | brown |
| LSS | brown |
| LST1 | brown |
| LTA4H | brown |
| LTB | turquoise |
| LTB4R | turquoise |
| LTB4R2 | turquoise |
| LTBP1 | blue |
| LTBP2 | blue |
| LTBP3 | lightyellow |
| LTBP4 | blue |
| LTBR | cyan |
| LTC4S | blue |
| LTF | turquoise |
| LTV1 | pink |
| LUC7L | blue |
| LUC7L2 | yellow |
| LUC7L3 | turquoise |
| LUM | purple |
| LXN | brown |
| LY6D | blue |
| LY6E | darkred |
| LY6G5B | magenta |
| LY6G5C | blue |
| LY6K | turquoise |
| LY75 | turquoise |
| LY86 | brown |
| LY96 | brown |
| LYAR | green |
| LYG1 | yellow |
| LYL1 | turquoise |
| LYN | brown |
| LYNX1 | darkred |
| LYPD1 | purple |
| LYPD3 | turquoise |
| LYPLA1 | midnightblue |
| LYPLA2 | blue |
| LYPLAL1 | red |
| LYRM1 | blue |
| LYRM2 | red |
| LYRM4 | pink |
| LYRM5 | cyan |
| LYRM7 | blue |
| LYSMD1 | lightgreen |
| LYSMD2 | turquoise |
| LYSMD4 | blue |
| LYST | turquoise |
| LYVE1 | black |
| LYZ | turquoise |
| LZIC | red |
| LZTFL1 | blue |
| LZTR1 | yellow |
| LZTS1 | blue |
| LZTS2 | magenta |
| M6PR | cyan |
| MACC1 | turquoise |
| MACF1 | blue |
| MACROD1 | brown |
| MACROD2 | brown |
| MAD1L1 | yellow |
| MAD2L1 | green |
| MAD2L1BP | magenta |
| MAD2L2 | blue |
| MADD | turquoise |
| MAF | black |
| MAF1 | darkred |
| MAFB | brown |
| MAFF | turquoise |
| MAFG | grey60 |
| MAFK | turquoise |
| MAGED1 | turquoise |
| MAGED2 | blue |
| MAGEF1 | brown |
| MAGEH1 | blue |
| MAGI1 | brown |
| MAGI2 | blue |
| MAGI3 | magenta |
| MAGIX | brown |
| MAGOH | green |
| MAGOHB | cyan |
| MAGT1 | tan |
| MAK16 | green |
| MAL2 | brown |
| MALAT1 | blue |
| MALL | blue |
| MALT1 | turquoise |
| MAMDC2 | blue |
| MAMDC4 | yellow |
| MAML1 | yellow |
| MAML2 | yellow |
| MAML3 | blue |
| MAMLD1 | yellow |
| MAN1A1 | turquoise |
| MAN1B1 | darkred |
| MAN1C1 | turquoise |
| MAN2A1 | turquoise |
| MAN2A2 | yellow |
| MAN2B1 | turquoise |
| MAN2B2 | brown |
| MAN2C1 | turquoise |
| MANBA | brown |
| MANBAL | brown |
| MANEA | turquoise |
| MANEAL | blue |
| MANF | brown |
| MANSC1 | brown |
| MAOA | blue |
| MAOB | turquoise |
| MAP1A | blue |
| MAP1B | blue |
| MAP1LC3A | brown |
| MAP1LC3B | blue |
| MAP1S | yellow |
| MAP2 | yellow |
| MAP2K3 | yellow |
| MAP2K5 | lightyellow |
| MAP2K6 | turquoise |
| MAP2K7 | yellow |
| MAP3K1 | turquoise |
| MAP3K10 | yellow |
| MAP3K12 | black |
| MAP3K13 | brown |
| MAP3K14 | turquoise |
| MAP3K3 | turquoise |
| MAP3K4 | pink |
| MAP3K5 | turquoise |
| MAP3K6 | yellow |
| MAP3K8 | brown |
| MAP3K9 | brown |
| MAP4 | lightyellow |
| MAP4K1 | turquoise |
| MAP4K2 | yellow |
| MAP4K3 | red |
| MAP4K4 | lightyellow |
| MAP4K5 | red |
| MAP6D1 | brown |
| MAP7 | brown |
| MAP7D1 | lightcyan |
| MAP7D2 | brown |
| MAP7D3 | blue |
| MAP9 | blue |
| MAPK10 | blue |
| MAPK11 | brown |
| MAPK12 | brown |
| MAPK13 | magenta |
| MAPK15 | darkred |
| MAPK3 | yellow |
| MAPK6 | turquoise |
| MAPK7 | yellow |
| MAPK8 | pink |
| MAPK8IP1 | yellow |
| MAPK8IP3 | yellow |
| MAPK9 | turquoise |
| MAPKAPK2 | yellow |
| MAPKAPK3 | brown |
| MAPKBP1 | lightyellow |
| MAPKSP1 | yellow |
| MAPRE2 | blue |
| MAPRE3 | yellow |
| 1-Mar | turquoise |
| 2-Mar | brown |
| 3-Mar | blue |
| 5-Mar | red |
| 6-Mar | brown |
| 8-Mar | turquoise |
| 9-Mar | turquoise |
| MARCKS | brown |
| MARCKSL1 | magenta |
| MARCO | brown |
| MARK1 | yellow |
| MARK2 | greenyellow |
| MARK4 | yellow |
| MARS2 | blue |
| MARVELD1 | lightcyan |
| MARVELD2 | brown |
| MARVELD3 | brown |
| MAST2 | yellow |
| MAST3 | turquoise |
| MAST4 | blue |
| MASTL | green |
| MAT2A | yellow |
| MAT2B | turquoise |
| MATK | turquoise |
| MATN2 | black |
| MATN3 | lightcyan |
| MAVS | yellow |
| MAX | turquoise |
| MAZ | blue |
| MB | turquoise |
| MBD3 | blue |
| MBD4 | red |
| MBD5 | blue |
| MBD6 | yellow |
| MBIP | blue |
| MBLAC1 | blue |
| MBNL1 | turquoise |
| MBNL2 | yellow |
| MBNL3 | turquoise |
| MBOAT2 | turquoise |
| MBOAT7 | yellow |
| MBP | turquoise |
| MBTD1 | grey60 |
| MCAM | blue |
| MCART1 | brown |
| MCART6 | turquoise |
| MCAT | turquoise |
| MCC | turquoise |
| MCCC1 | brown |
| MCCC2 | darkgreen |
| MCEE | blue |
| MCF2L | yellow |
| MCF2L2 | green |
| MCL1 | turquoise |
| MCM10 | green |
| MCM2 | green |
| MCM3 | green |
| MCM3AP | pink |
| MCM3APAS | pink |
| MCM4 | green |
| MCM5 | green |
| MCM6 | green |
| MCM7 | blue |
| MCM8 | green |
| MCM9 | pink |
| MCOLN1 | brown |
| MCOLN2 | turquoise |
| MCOLN3 | yellow |
| MCPH1 | turquoise |
| MCTP1 | turquoise |
| MCTP2 | turquoise |
| MCTS1 | blue |
| MDC1 | magenta |
| MDFI | brown |
| MDFIC | turquoise |
| MDGA1 | brown |
| MDH1 | red |
| MDH2 | brown |
| MDK | turquoise |
| MDM1 | turquoise |
| MDM2 | turquoise |
| MDM4 | turquoise |
| MDN1 | red |
| MDP1 | turquoise |
| ME1 | tan |
| ME2 | brown |
| ME3 | blue |
| MEA1 | magenta |
| MEAF6 | yellow |
| MECOM | blue |
| MECP2 | turquoise |
| MECR | brown |
| MED1 | turquoise |
| MED10 | blue |
| MED12 | yellow |
| MED13 | grey60 |
| MED13L | blue |
| MED14 | yellow |
| MED15 | yellow |
| MED17 | pink |
| MED18 | yellow |
| MED19 | brown |
| MED20 | magenta |
| MED21 | cyan |
| MED22 | yellow |
| MED23 | pink |
| MED24 | yellow |
| MED25 | yellow |
| MED27 | midnightblue |
| MED28 | turquoise |
| MED29 | yellow |
| MED30 | midnightblue |
| MED6 | turquoise |
| MED8 | green |
| MEF2A | blue |
| MEF2B | turquoise |
| MEF2C | turquoise |
| MEF2D | yellow |
| MEG3 | blue |
| MEGF10 | turquoise |
| MEGF11 | blue |
| MEGF6 | blue |
| MEGF8 | yellow |
| MEGF9 | blue |
| MEI1 | turquoise |
| MEIS1 | blue |
| MEIS2 | blue |
| MEIS3 | blue |
| MEIS3P1 | blue |
| MELK | green |
| MEMO1P1 | tan |
| MEN1 | yellow |
| MEOX2 | black |
| MEPCE | blue |
| MERTK | brown |
| MESDC1 | brown |
| MESP1 | turquoise |
| MESP2 | turquoise |
| MEST | blue |
| MET | turquoise |
| METAP1 | red |
| METRN | turquoise |
| METRNL | brown |
| METT5D1 | red |
| METTL1 | blue |
| METTL10 | salmon |
| METTL12 | salmon |
| METTL13 | lightgreen |
| METTL2A | grey60 |
| METTL2B | blue |
| METTL5 | brown |
| METTL6 | blue |
| METTL7A | turquoise |
| METTL8 | pink |
| MEX3A | lightgreen |
| MEX3B | magenta |
| MEX3C | red |
| MEX3D | turquoise |
| MFAP2 | lightcyan |
| MFAP3L | turquoise |
| MFAP4 | black |
| MFAP5 | blue |
| MFGE8 | brown |
| MFHAS1 | turquoise |
| MFI2 | brown |
| MFN1 | brown |
| MFN2 | tan |
| MFNG | turquoise |
| MFSD1 | brown |
| MFSD11 | grey60 |
| MFSD2A | yellow |
| MFSD3 | darkred |
| MFSD4 | purple |
| MFSD6 | yellow |
| MFSD6L | brown |
| MFSD7 | brown |
| MFSD8 | turquoise |
| MFSD9 | blue |
| MGAT1 | brown |
| MGAT3 | turquoise |
| MGAT4A | turquoise |
| MGAT4B | turquoise |
| MGAT5 | turquoise |
| MGLL | blue |
| MGMT | turquoise |
| MGP | blue |
| MGRN1 | yellow |
| MGST1 | turquoise |
| MGST2 | brown |
| MGST3 | turquoise |
| MIA | brown |
| MIA3 | yellow |
| MIAT | turquoise |
| MIB1 | red |
| MIB2 | yellow |
| MICA | blue |
| MICAL1 | turquoise |
| MICAL2 | purple |
| MICAL3 | yellow |
| MICALL1 | brown |
| MICALL2 | salmon |
| MICB | turquoise |
| MID1 | yellow |
| MID1IP1 | blue |
| MID2 | blue |
| MIDN | yellow |
| MIER1 | turquoise |
| MIER2 | yellow |
| MIER3 | turquoise |
| MIF | turquoise |
| MIF4GD | grey60 |
| MIIP | blue |
| MINA | blue |
| MINK1 | yellow |
| MINPP1 | turquoise |
| MIOS | brown |
| MIPEP | turquoise |
| MIPOL1 | brown |
| MIR1302-11 | yellow |
| MIR17HG | red |
| MIR181A2HG | brown |
| MITF | blue |
| MKI67 | green |
| MKI67IP | greenyellow |
| MKKS | midnightblue |
| MKL1 | blue |
| MKL2 | blue |
| MKNK1 | brown |
| MKNK2 | tan |
| MKRN1 | blue |
| MKRN3 | blue |
| MKS1 | yellow |
| MLANA | turquoise |
| MLEC | yellow |
| MLF1 | turquoise |
| MLF1IP | green |
| MLF2 | cyan |
| MLH1 | blue |
| MLKL | turquoise |
| MLL | yellow |
| MLL2 | yellow |
| MLL3 | brown |
| MLLT1 | yellow |
| MLLT10 | green |
| MLLT11 | yellow |
| MLLT3 | turquoise |
| MLLT4 | pink |
| MLLT6 | yellow |
| MLPH | tan |
| MLST8 | blue |
| MLXIP | yellow |
| MMAA | turquoise |
| MMAB | turquoise |
| MMACHC | green |
| MMADHC | yellow |
| MMD | brown |
| MME | black |
| MMEL1 | blue |
| MMGT1 | red |
| MMP1 | purple |
| MMP11 | lightcyan |
| MMP12 | blue |
| MMP13 | lightcyan |
| MMP14 | purple |
| MMP15 | turquoise |
| MMP16 | blue |
| MMP17 | blue |
| MMP19 | black |
| MMP2 | purple |
| MMP23B | black |
| MMP25 | turquoise |
| MMP28 | blue |
| MMP3 | blue |
| MMP7 | cyan |
| MMP9 | brown |
| MMRN1 | black |
| MMRN2 | black |
| MND1 | green |
| MNDA | brown |
| MNT | yellow |
| MNX1 | turquoise |
| MOBKL1A | blue |
| MOBKL2A | turquoise |
| MOBKL2B | turquoise |
| MOBKL2C | turquoise |
| MOBKL3 | turquoise |
| MOCOS | blue |
| MOCS1 | black |
| MOCS2 | blue |
| MOCS3 | blue |
| MOGAT2 | tan |
| MOGS | blue |
| MON1A | darkred |
| MON1B | salmon |
| MORC2 | brown |
| MORC3 | turquoise |
| MORC4 | turquoise |
| MORF4L2 | turquoise |
| MORN1 | brown |
| MORN2 | turquoise |
| MORN4 | brown |
| MOSC1 | turquoise |
| MOSC2 | blue |
| MOSPD1 | turquoise |
| MOSPD2 | blue |
| MOSPD3 | brown |
| MOV10 | turquoise |
| MOV10L1 | purple |
| MOXD1 | turquoise |
| MPDU1 | brown |
| MPDZ | yellow |
| MPEG1 | turquoise |
| MPG | blue |
| MPHOSPH6 | tan |
| MPHOSPH8 | turquoise |
| MPHOSPH9 | turquoise |
| MPI | blue |
| MPND | blue |
| MPP1 | brown |
| MPP3 | turquoise |
| MPP6 | orange |
| MPP7 | blue |
| MPPED2 | blue |
| MPRIP | yellow |
| MPST | blue |
| MPV17L | tan |
| MPV17L2 | blue |
| MPZ | turquoise |
| MPZL1 | brown |
| MPZL2 | turquoise |
| MPZL3 | red |
| MR1 | turquoise |
| MRAP2 | brown |
| MRAS | blue |
| MRC1L1 | black |
| MRC2 | purple |
| MRE11A | pink |
| MREG | blue |
| MRFAP1 | blue |
| MRGPRF | blue |
| MRGPRX3 | turquoise |
| MRI1 | blue |
| MRPL1 | red |
| MRPL10 | turquoise |
| MRPL11 | blue |
| MRPL12 | grey60 |
| MRPL13 | midnightblue |
| MRPL14 | magenta |
| MRPL15 | midnightblue |
| MRPL17 | red |
| MRPL18 | blue |
| MRPL19 | turquoise |
| MRPL2 | magenta |
| MRPL20 | blue |
| MRPL21 | blue |
| MRPL22 | blue |
| MRPL23 | blue |
| MRPL24 | lightgreen |
| MRPL27 | grey60 |
| MRPL28 | blue |
| MRPL30 | turquoise |
| MRPL32 | blue |
| MRPL33 | turquoise |
| MRPL34 | blue |
| MRPL35 | blue |
| MRPL35P3 | blue |
| MRPL36 | turquoise |
| MRPL37 | green |
| MRPL38 | grey60 |
| MRPL39 | blue |
| MRPL4 | blue |
| MRPL40 | yellow |
| MRPL41 | blue |
| MRPL42 | midnightblue |
| MRPL45 | yellow |
| MRPL47 | blue |
| MRPL48 | turquoise |
| MRPL49 | brown |
| MRPL50 | red |
| MRPL51 | cyan |
| MRPL52 | blue |
| MRPL53 | blue |
| MRPL54 | yellow |
| MRPL55 | blue |
| MRPL9 | lightgreen |
| MRPS10 | magenta |
| MRPS11 | blue |
| MRPS12 | blue |
| MRPS15 | green |
| MRPS16 | blue |
| MRPS17 | brown |
| MRPS18A | magenta |
| MRPS18B | magenta |
| MRPS18C | brown |
| MRPS2 | blue |
| MRPS21 | lightgreen |
| MRPS23 | grey60 |
| MRPS24 | blue |
| MRPS25 | blue |
| MRPS26 | blue |
| MRPS27 | blue |
| MRPS28 | midnightblue |
| MRPS30 | brown |
| MRPS31 | turquoise |
| MRPS33 | blue |
| MRPS34 | blue |
| MRPS35 | cyan |
| MRPS36 | yellow |
| MRPS6 | turquoise |
| MRPS7 | grey60 |
| MRPS9 | blue |
| MRRF | red |
| MRS2 | green |
| MRTO4 | green |
| MRVI1 | blue |
| MS4A4A | brown |
| MS4A4E | turquoise |
| MS4A6A | turquoise |
| MS4A7 | brown |
| MSC | brown |
| MSH2 | green |
| MSH5 | magenta |
| MSH6 | green |
| MSI2 | grey60 |
| MSL1 | yellow |
| MSL3 | turquoise |
| MSL3L2 | turquoise |
| MSLN | brown |
| MSMP | yellow |
| MSN | brown |
| MSR1 | brown |
| MSRA | blue |
| MSRB2 | turquoise |
| MSRB3 | blue |
| MST1 | black |
| MST1R | blue |
| MST4 | green |
| MSTO1 | lightgreen |
| MSX1 | blue |
| MSX2 | tan |
| MT-ATP6 | greenyellow |
| MT-CO1 | greenyellow |
| MT-CO2 | greenyellow |
| MT-CO3 | greenyellow |
| MT-CYB | greenyellow |
| MT-ND1 | greenyellow |
| MT-ND2 | greenyellow |
| MT-ND3 | greenyellow |
| MT-ND4 | greenyellow |
| MT-ND4L | greenyellow |
| MT-ND5 | greenyellow |
| MT-ND6 | greenyellow |
| MT1E | blue |
| MT1F | blue |
| MT1G | blue |
| MT1M | blue |
| MT1X | blue |
| MT2A | blue |
| MTA1 | yellow |
| MTA3 | red |
| MTAP | brown |
| MTBP | midnightblue |
| MTCH1 | magenta |
| MTCH2 | red |
| MTCP1 | red |
| MTCP1NB | turquoise |
| MTDH | midnightblue |
| MTERF | yellow |
| MTERFD1 | midnightblue |
| MTERFD2 | turquoise |
| MTF1 | yellow |
| MTF2 | pink |
| MTFP1 | blue |
| MTFR1 | midnightblue |
| MTG1 | yellow |
| MTHFD1 | green |
| MTHFD1L | red |
| MTHFD2 | green |
| MTHFD2L | red |
| MTHFR | turquoise |
| MTHFS | turquoise |
| MTHFSD | yellow |
| MTIF2 | green |
| MTL5 | brown |
| MTM1 | turquoise |
| MTMR1 | pink |
| MTMR10 | blue |
| MTMR11 | blue |
| MTMR12 | brown |
| MTMR2 | turquoise |
| MTMR3 | blue |
| MTMR4 | grey60 |
| MTMR7 | blue |
| MTMR8 | turquoise |
| MTMR9 | blue |
| MTMR9L | black |
| MTO1 | turquoise |
| MTPAP | green |
| MTPN | green |
| MTRF1L | pink |
| MTRNR2L9 | red |
| MTRR | lightgreen |
| MTSS1 | blue |
| MTSS1L | magenta |
| MTUS1 | blue |
| MTX1 | lightgreen |
| MTX2 | brown |
| MTX3 | blue |
| MUC1 | turquoise |
| MUC15 | brown |
| MUC16 | lightgreen |
| MUC20 | yellow |
| MUC5B | brown |
| MUCL1 | tan |
| MUM1 | lightyellow |
| MUSTN1 | turquoise |
| MUT | magenta |
| MUTYH | yellow |
| MVD | turquoise |
| MVK | tan |
| MVP | brown |
| MX1 | turquoise |
| MX2 | turquoise |
| MXD1 | brown |
| MXD3 | blue |
| MXD4 | turquoise |
| MXI1 | turquoise |
| MXRA5 | purple |
| MXRA7 | blue |
| MXRA8 | blue |
| MYADM | lightyellow |
| MYBBP1A | yellow |
| MYBL1 | midnightblue |
| MYBL2 | green |
| MYBPC1 | brown |
| MYC | blue |
| MYCBP | blue |
| MYCBP2 | turquoise |
| MYCL1 | turquoise |
| MYCT1 | black |
| MYD88 | brown |
| MYEF2 | brown |
| MYEOV2 | yellow |
| MYH10 | blue |
| MYH11 | black |
| MYH14 | brown |
| MYH7B | yellow |
| MYH9 | blue |
| MYL12A | blue |
| MYL12B | turquoise |
| MYL5 | brown |
| MYL6 | lightcyan |
| MYL6B | blue |
| MYL9 | blue |
| MYLIP | turquoise |
| MYLK | black |
| MYO10 | turquoise |
| MYO15B | black |
| MYO18A | turquoise |
| MYO19 | green |
| MYO1B | lightcyan |
| MYO1C | yellow |
| MYO1D | lightcyan |
| MYO1E | lightcyan |
| MYO1F | turquoise |
| MYO1G | turquoise |
| MYO3B | brown |
| MYO5A | brown |
| MYO5B | brown |
| MYO5C | turquoise |
| MYO6 | brown |
| MYO7A | turquoise |
| MYO9A | blue |
| MYO9B | brown |
| MYOF | blue |
| MYOM2 | darkgreen |
| MYOZ1 | brown |
| MYPOP | yellow |
| MYST2 | grey60 |
| MYST3 | turquoise |
| MYST4 | yellow |
| MZF1 | turquoise |
| N4BP2 | turquoise |
| N4BP2L1 | turquoise |
| N4BP2L2 | turquoise |
| N4BP2L2IT1 | turquoise |
| N6AMT1 | turquoise |
| N6AMT2 | turquoise |
| NAA10 | blue |
| NAA15 | red |
| NAA16 | pink |
| NAA20 | blue |
| NAA38 | green |
| NAA40 | yellow |
| NAA50 | blue |
| NAAA | brown |
| NAALADL2 | brown |
| NAB1 | blue |
| NAB2 | blue |
| NACA | salmon |
| NACC1 | green |
| NACC2 | blue |
| NADK | turquoise |
| NAE1 | turquoise |
| NAGA | brown |
| NAGK | brown |
| NAGLU | brown |
| NAGS | blue |
| NAIP | black |
| NALCN | turquoise |
| NAMPTL | tan |
| NANOS1 | turquoise |
| NANS | blue |
| NAP1L1 | red |
| NAP1L5 | blue |
| NAPA | yellow |
| NAPEPLD | brown |
| NAPG | turquoise |
| NAPRT1 | darkred |
| NAPSB | turquoise |
| NARF | grey60 |
| NARFL | blue |
| NARS | red |
| NARS2 | brown |
| NASP | green |
| NAT1 | blue |
| NAT10 | red |
| NAT14 | turquoise |
| NAT6 | yellow |
| NAT8L | turquoise |
| NAT9 | grey60 |
| NAV1 | blue |
| NAV2 | yellow |
| NBEA | blue |
| NBEAL1 | pink |
| NBEAL2 | yellow |
| NBL1 | purple |
| NBN | midnightblue |
| NBPF1 | blue |
| NBPF12 | yellow |
| NBPF14 | brown |
| NBPF15 | blue |
| NBPF3 | yellow |
| NBPF8 | yellow |
| NBR2 | blue |
| NCALD | blue |
| NCAPD2 | cyan |
| NCAPD3 | green |
| NCAPG | green |
| NCAPG2 | green |
| NCAPH | green |
| NCAPH2 | blue |
| NCBP2 | green |
| NCCRP1 | brown |
| NCDN | yellow |
| NCF1 | turquoise |
| NCF2 | brown |
| NCF4 | turquoise |
| NCK1 | blue |
| NCK2 | magenta |
| NCKAP1 | turquoise |
| NCKAP1L | turquoise |
| NCKAP5 | brown |
| NCL | greenyellow |
| NCLN | blue |
| NCOA1 | turquoise |
| NCOA2 | midnightblue |
| NCOA3 | turquoise |
| NCOA6 | yellow |
| NCOA7 | red |
| NCOR1 | turquoise |
| NCOR2 | lightyellow |
| NCRNA00081 | black |
| NCRNA00085 | yellow |
| NCRNA00086 | orange |
| NCRNA00087 | brown |
| NCRNA00094 | yellow |
| NCRNA00103 | blue |
| NCRNA00105 | turquoise |
| NCRNA00106 | yellow |
| NCRNA00107 | royalblue |
| NCRNA00115 | turquoise |
| NCRNA00116 | yellow |
| NCRNA00117 | turquoise |
| NCRNA00152 | lightcyan |
| NCRNA00164 | yellow |
| NCRNA00171 | magenta |
| NCRNA00174 | yellow |
| NCRNA00182 | blue |
| NCRNA00183 | turquoise |
| NCRNA00188 | salmon |
| NCRNA00201 | red |
| NCRNA00202 | red |
| NCRNA00205 | turquoise |
| NCRNA00219 | salmon |
| NCRNA00265 | yellow |
| NCS1 | green |
| NCSTN | lightgreen |
| NCUBE1 | turquoise |
| NDC80 | green |
| NDE1 | yellow |
| NDFIP2 | turquoise |
| NDN | black |
| NDOR1 | yellow |
| NDRG1 | turquoise |
| NDRG2 | brown |
| NDRG3 | yellow |
| NDRG4 | turquoise |
| NDST1 | yellow |
| NDST2 | turquoise |
| NDUFA1 | yellow |
| NDUFA10 | turquoise |
| NDUFA11 | blue |
| NDUFA12 | yellow |
| NDUFA13 | blue |
| NDUFA2 | greenyellow |
| NDUFA3 | greenyellow |
| NDUFA4 | blue |
| NDUFA4L2 | blue |
| NDUFA5 | brown |
| NDUFA6 | blue |
| NDUFA7 | blue |
| NDUFA8 | blue |
| NDUFA9 | cyan |
| NDUFAB1 | blue |
| NDUFAF2 | salmon |
| NDUFAF3 | blue |
| NDUFAF4 | green |
| NDUFB1 | greenyellow |
| NDUFB10 | blue |
| NDUFB11 | blue |
| NDUFB2 | blue |
| NDUFB3 | blue |
| NDUFB4 | yellow |
| NDUFB5 | blue |
| NDUFB6 | turquoise |
| NDUFB7 | blue |
| NDUFB9 | midnightblue |
| NDUFC1 | yellow |
| NDUFC2 | turquoise |
| NDUFS2 | lightgreen |
| NDUFS4 | greenyellow |
| NDUFS5 | blue |
| NDUFS6 | turquoise |
| NDUFS7 | yellow |
| NDUFS8 | blue |
| NDUFV1 | blue |
| NDUFV2 | blue |
| NDUFV3 | green |
| NEB | turquoise |
| NEBL | turquoise |
| NECAB3 | brown |
| NECAP1 | cyan |
| NEDD1 | red |
| NEDD4 | yellow |
| NEDD4L | brown |
| NEDD8 | yellow |
| NEDD9 | turquoise |
| NEGR1 | black |
| NEIL1 | blue |
| NEIL2 | blue |
| NEIL3 | green |
| NEK10 | blue |
| NEK11 | blue |
| NEK2 | green |
| NEK3 | pink |
| NEK6 | brown |
| NEK7 | red |
| NEK9 | turquoise |
| NELF | magenta |
| NELL2 | turquoise |
| NENF | blue |
| NEO1 | yellow |
| NES | blue |
| NET1 | brown |
| NETO2 | green |
| NEU1 | magenta |
| NEU3 | turquoise |
| NEURL1B | blue |
| NEURL2 | blue |
| NEURL3 | blue |
| NEXN | black |
| NF1 | blue |
| NF2 | yellow |
| NFAM1 | brown |
| NFASC | yellow |
| NFAT5 | yellow |
| NFATC1 | yellow |
| NFATC2 | turquoise |
| NFATC3 | turquoise |
| NFATC4 | blue |
| NFE2L1 | blue |
| NFE2L2 | blue |
| NFE2L3 | turquoise |
| NFIA | blue |
| NFIB | brown |
| NFIC | yellow |
| NFIL3 | brown |
| NFIX | yellow |
| NFKB1 | turquoise |
| NFKB2 | turquoise |
| NFKBIA | turquoise |
| NFKBIB | blue |
| NFKBID | turquoise |
| NFKBIE | turquoise |
| NFKBIL1 | magenta |
| NFKBIL2 | darkred |
| NFKBIZ | blue |
| NFRKB | red |
| NFS1 | yellow |
| NFU1 | brown |
| NFXL1 | red |
| NFYA | magenta |
| NFYC | yellow |
| NGDN | blue |
| NGEF | brown |
| NGFR | black |
| NGFRAP1 | turquoise |
| NGLY1 | turquoise |
| NGRN | turquoise |
| NHEDC2 | turquoise |
| NHEJ1 | turquoise |
| NHLRC1 | turquoise |
| NHLRC3 | turquoise |
| NHP2 | turquoise |
| NHP2L1 | blue |
| NHS | yellow |
| NHSL1 | greenyellow |
| NHSL2 | black |
| NICN1 | blue |
| NID1 | blue |
| NID2 | purple |
| NIN | turquoise |
| NINJ1 | brown |
| NINJ2 | cyan |
| NINL | magenta |
| NIP7 | green |
| NIPA1 | green |
| NIPA2 | midnightblue |
| NIPAL1 | brown |
| NIPAL2 | midnightblue |
| NIPAL3 | brown |
| NIPBL | red |
| NIPSNAP1 | brown |
| NIPSNAP3A | red |
| NIPSNAP3B | blue |
| NISCH | lightyellow |
| NIT1 | lightgreen |
| NIT2 | blue |
| NKD2 | blue |
| NKG7 | turquoise |
| NKIRAS2 | yellow |
| NKTR | blue |
| NKX3-1 | brown |
| NLE1 | salmon |
| NLGN2 | lightyellow |
| NLN | green |
| NLRC3 | turquoise |
| NLRC5 | turquoise |
| NLRP1 | turquoise |
| NLRP2 | blue |
| NLRP3 | brown |
| NLRX1 | red |
| NMB | turquoise |
| NMD3 | turquoise |
| NME1 | grey60 |
| NME2 | grey60 |
| NME3 | yellow |
| NME4 | turquoise |
| NME7 | brown |
| NMI | turquoise |
| NMNAT1 | blue |
| NMRAL1 | brown |
| NMT2 | yellow |
| NNAT | blue |
| NNMT | blue |
| NNT | turquoise |
| NOB1 | blue |
| NOC2L | blue |
| NOC4L | blue |
| NOD1 | turquoise |
| NOD2 | brown |
| NOL10 | green |
| NOL11 | grey60 |
| NOL12 | blue |
| NOL3 | turquoise |
| NOL6 | green |
| NOL7 | green |
| NOL9 | red |
| NOLC1 | blue |
| NOM1 | green |
| NOMO1 | turquoise |
| NOMO2 | turquoise |
| NOMO3 | yellow |
| NONO | magenta |
| NOP10 | yellow |
| NOP14 | yellow |
| NOP16 | blue |
| NOP2 | cyan |
| NOP56 | blue |
| NOP58 | green |
| NOS1AP | yellow |
| NOS3 | brown |
| NOSTRIN | blue |
| NOTCH1 | yellow |
| NOTCH3 | yellow |
| NOTCH4 | black |
| NOV | blue |
| NOX4 | purple |
| NOXA1 | brown |
| NOXO1 | blue |
| NPAS2 | yellow |
| NPAS3 | blue |
| NPC1 | yellow |
| NPC2 | brown |
| NPDC1 | blue |
| NPEPL1 | yellow |
| NPEPPS | yellow |
| NPFF | lightyellow |
| NPHP1 | turquoise |
| NPHP3 | blue |
| NPHP4 | yellow |
| NPIP | yellow |
| NPIPL2 | yellow |
| NPIPL3 | yellow |
| NPIPP1 | yellow |
| NPL | brown |
| NPLOC4 | grey60 |
| NPM1 | blue |
| NPM2 | brown |
| NPM3 | blue |
| NPNT | turquoise |
| NPR1 | black |
| NPR2 | blue |
| NPR3 | turquoise |
| NPTN | blue |
| NPTXR | salmon |
| NPY1R | brown |
| NQO1 | turquoise |
| NQO2 | blue |
| NR1D2 | pink |
| NR1H3 | brown |
| NR1I3 | lightgreen |
| NR2C1 | blue |
| NR2C2 | yellow |
| NR2C2AP | blue |
| NR2F1 | black |
| NR2F2 | yellow |
| NR2F6 | turquoise |
| NR3C1 | turquoise |
| NR3C2 | blue |
| NR4A1 | blue |
| NR4A2 | yellow |
| NR4A3 | turquoise |
| NR6A1 | brown |
| NRARP | turquoise |
| NRAS | red |
| NRBP2 | darkred |
| NRG1 | blue |
| NRG2 | purple |
| NRIP1 | blue |
| NRIP2 | turquoise |
| NRIP3 | purple |
| NRM | magenta |
| NRN1 | blue |
| NRP1 | blue |
| NRP2 | brown |
| NRSN1 | royalblue |
| NRSN2 | yellow |
| NRTN | brown |
| NRXN2 | blue |
| NRXN3 | blue |
| NSD1 | yellow |
| NSDHL | turquoise |
| NSF | turquoise |
| NSFL1C | greenyellow |
| NSG1 | greenyellow |
| NSMAF | turquoise |
| NSMCE1 | yellow |
| NSMCE2 | midnightblue |
| NSMCE4A | turquoise |
| NSUN2 | turquoise |
| NSUN3 | turquoise |
| NSUN4 | yellow |
| NSUN5 | blue |
| NSUN5P1 | yellow |
| NSUN5P2 | brown |
| NSUN6 | yellow |
| NSUN7 | brown |
| NT5C | grey60 |
| NT5C1B | yellow |
| NT5C2 | yellow |
| NT5C3 | turquoise |
| NT5C3L | brown |
| NT5DC1 | pink |
| NT5DC2 | turquoise |
| NT5DC3 | turquoise |
| NT5E | blue |
| NT5M | brown |
| NTAN1 | turquoise |
| NTHL1 | blue |
| NTM | purple |
| NTN1 | magenta |
| NTN3 | brown |
| NTN4 | darkturquoise |
| NTRK2 | blue |
| NTRK3 | blue |
| NUAK1 | purple |
| NUB1 | turquoise |
| NUBP2 | blue |
| NUBPL | red |
| NUCB1 | blue |
| NUCB2 | tan |
| NUCKS1 | green |
| NUDC | yellow |
| NUDCD1 | midnightblue |
| NUDCD3 | yellow |
| NUDT1 | green |
| NUDT10 | purple |
| NUDT11 | blue |
| NUDT12 | red |
| NUDT13 | turquoise |
| NUDT14 | turquoise |
| NUDT15 | red |
| NUDT16 | turquoise |
| NUDT16L1 | blue |
| NUDT16P | brown |
| NUDT17 | lightgreen |
| NUDT18 | brown |
| NUDT19 | brown |
| NUDT2 | yellow |
| NUDT21 | turquoise |
| NUDT22 | yellow |
| NUDT3 | magenta |
| NUDT4 | blue |
| NUDT5 | blue |
| NUDT6 | blue |
| NUDT7 | blue |
| NUDT8 | tan |
| NUDT9 | turquoise |
| NUF2 | green |
| NUMA1 | yellow |
| NUMBL | yellow |
| NUP107 | green |
| NUP153 | green |
| NUP155 | green |
| NUP188 | yellow |
| NUP205 | blue |
| NUP210 | turquoise |
| NUP214 | yellow |
| NUP35 | blue |
| NUP37 | red |
| NUP50 | yellow |
| NUP54 | red |
| NUP62 | yellow |
| NUP62CL | blue |
| NUP85 | grey60 |
| NUP88 | green |
| NUP93 | green |
| NUPL2 | turquoise |
| NUPR1 | turquoise |
| NUS1 | red |
| NUSAP1 | green |
| NUTF2 | turquoise |
| NVL | brown |
| NXF1 | yellow |
| NXN | purple |
| NXPH3 | blue |
| NXPH4 | turquoise |
| NXT1 | blue |
| NXT2 | blue |
| NYNRIN | yellow |
| OAF | black |
| OAS1 | turquoise |
| OAS2 | turquoise |
| OAS3 | turquoise |
| OASL | turquoise |
| OAT | turquoise |
| OAZ1 | brown |
| OAZ3 | blue |
| OBFC1 | blue |
| OBFC2A | turquoise |
| OBSCN | yellow |
| OBSL1 | brown |
| OCEL1 | blue |
| OCIAD1 | red |
| OCIAD2 | blue |
| OCLN | turquoise |
| OCRL | turquoise |
| ODC1 | green |
| ODF2 | yellow |
| ODF2L | pink |
| ODF3B | turquoise |
| ODZ3 | blue |
| ODZ4 | purple |
| OFD1 | red |
| OGDH | yellow |
| OGFOD2 | greenyellow |
| OGFR | turquoise |
| OGFRL1 | purple |
| OGG1 | yellow |
| OGN | black |
| OGT | turquoise |
| OIP5 | green |
| OLA1 | turquoise |
| OLFM1 | turquoise |
| OLFM2 | magenta |
| OLFM4 | blue |
| OLFML1 | blue |
| OLFML2A | blue |
| OLFML2B | purple |
| OLFML3 | blue |
| OLR1 | brown |
| OMA1 | turquoise |
| OMD | blue |
| OPA1 | blue |
| OPHN1 | yellow |
| OPLAH | darkred |
| OPN3 | turquoise |
| OPTN | turquoise |
| OR10AD1 | turquoise |
| OR2A1 | turquoise |
| OR2A7 | brown |
| OR2B2 | turquoise |
| OR2I1P | turquoise |
| OR7E12P | black |
| ORAI1 | blue |
| ORAI2 | turquoise |
| ORAI3 | turquoise |
| ORAOV1 | yellow |
| ORC1L | green |
| ORC2L | pink |
| ORC3L | red |
| ORC4L | red |
| ORC6L | green |
| ORMDL2 | turquoise |
| ORMDL3 | turquoise |
| OSBP2 | yellow |
| OSBPL10 | blue |
| OSBPL1A | brown |
| OSBPL2 | brown |
| OSBPL3 | turquoise |
| OSBPL5 | tan |
| OSBPL6 | purple |
| OSBPL7 | turquoise |
| OSBPL9 | blue |
| OSCAR | brown |
| OSCP1 | brown |
| OSGEPL1 | turquoise |
| OSGIN1 | turquoise |
| OSGIN2 | blue |
| OSM | brown |
| OSMR | blue |
| OSR1 | blue |
| OSR2 | blue |
| OST4 | yellow |
| OSTC | yellow |
| OSTF1 | brown |
| OSTM1 | brown |
| OTUD1 | turquoise |
| OTUD3 | red |
| OTUD6B | midnightblue |
| OTUD7B | lightgreen |
| OTX1 | magenta |
| OVCA2 | turquoise |
| OVGP1 | blue |
| OVOL1 | turquoise |
| OVOL2 | brown |
| OXA1L | red |
| OXCT1 | brown |
| OXER1 | darkgreen |
| OXNAD1 | turquoise |
| OXR1 | midnightblue |
| OXSM | blue |
| OXSR1 | turquoise |
| OXTR | blue |
| P2RX4 | brown |
| P2RX5 | turquoise |
| P2RX7 | brown |
| P2RY1 | brown |
| P2RY10 | turquoise |
| P2RY11 | turquoise |
| P2RY13 | turquoise |
| P2RY2 | turquoise |
| P2RY6 | brown |
| P4HA1 | lightcyan |
| P4HA2 | lightcyan |
| P4HA3 | purple |
| P4HB | grey60 |
| P4HTM | blue |
| PA2G4 | green |
| PAAF1 | yellow |
| PABPC1 | greenyellow |
| PABPC1L | yellow |
| PABPC1P1 | turquoise |
| PABPC1P3 | turquoise |
| PABPC4 | green |
| PABPC4L | yellow |
| PACRGL | pink |
| PACS1 | yellow |
| PACS2 | yellow |
| PACSIN2 | yellow |
| PACSIN3 | brown |
| PADI2 | brown |
| PAF1 | greenyellow |
| PAFAH1B2 | red |
| PAFAH1B3 | brown |
| PAFAH2 | blue |
| PAG1 | turquoise |
| PAICS | green |
| PAIP1 | brown |
| PAIP2B | brown |
| PAK1 | yellow |
| PAK1IP1 | blue |
| PAK2 | yellow |
| PAK3 | turquoise |
| PAK4 | brown |
| PALLD | blue |
| PALM | blue |
| PALM2 | turquoise |
| PALM2-AKAP2 | blue |
| PALMD | blue |
| PAM | blue |
| PAM16 | blue |
| PAN2 | turquoise |
| PAN3 | turquoise |
| PANK1 | blue |
| PANK2 | turquoise |
| PANK3 | red |
| PANK4 | turquoise |
| PANX1 | red |
| PANX2 | turquoise |
| PAOX | turquoise |
| PAPD4 | turquoise |
| PAPD7 | yellow |
| PAPLN | turquoise |
| PAPSS1 | brown |
| PAPSS2 | blue |
| PAQR3 | brown |
| PAQR4 | blue |
| PAQR5 | turquoise |
| PAQR6 | brown |
| PAQR7 | blue |
| PAQR8 | turquoise |
| PARD3 | turquoise |
| PARD3B | blue |
| PARD6A | blue |
| PARD6B | brown |
| PARD6G | magenta |
| PARG | pink |
| PARK2 | blue |
| PARK7 | midnightblue |
| PARL | green |
| PARM1 | turquoise |
| PARP1 | green |
| PARP10 | turquoise |
| PARP11 | turquoise |
| PARP12 | turquoise |
| PARP14 | turquoise |
| PARP15 | turquoise |
| PARP2 | green |
| PARP3 | turquoise |
| PARP4 | turquoise |
| PARP6 | yellow |
| PARP8 | turquoise |
| PARP9 | turquoise |
| PART1 | brown |
| PARVA | blue |
| PARVB | brown |
| PARVG | turquoise |
| PASK | turquoise |
| PATL1 | yellow |
| PATZ1 | yellow |
| PAWR | turquoise |
| PAX6 | blue |
| PAX8 | blue |
| PAXIP1 | brown |
| PBEF1 | tan |
| PBK | green |
| PBLD | blue |
| PBX1 | brown |
| PBX2 | magenta |
| PBX3 | turquoise |
| PBXIP1 | turquoise |
| PC | blue |
| PCBD1 | blue |
| PCBP2 | yellow |
| PCBP3 | brown |
| PCBP4 | brown |
| PCCA | blue |
| PCCB | blue |
| PCDH1 | turquoise |
| PCDH12 | black |
| PCDH17 | lightcyan |
| PCDH18 | black |
| PCDH7 | lightcyan |
| PCDHA10 | orange |
| PCDHA11 | orange |
| PCDHA12 | orange |
| PCDHA13 | orange |
| PCDHA2 | orange |
| PCDHA4 | orange |
| PCDHA6 | orange |
| PCDHA8 | orange |
| PCDHAC1 | orange |
| PCDHAC2 | orange |
| PCDHB10 | orange |
| PCDHB11 | orange |
| PCDHB12 | orange |
| PCDHB13 | orange |
| PCDHB14 | orange |
| PCDHB15 | orange |
| PCDHB16 | orange |
| PCDHB18 | orange |
| PCDHB2 | orange |
| PCDHB3 | orange |
| PCDHB4 | orange |
| PCDHB5 | orange |
| PCDHB8 | orange |
| PCDHB9 | orange |
| PCDHGA1 | orange |
| PCDHGA10 | orange |
| PCDHGA11 | orange |
| PCDHGA12 | orange |
| PCDHGA2 | orange |
| PCDHGA3 | orange |
| PCDHGA5 | orange |
| PCDHGA6 | orange |
| PCDHGA7 | orange |
| PCDHGA8 | orange |
| PCDHGB1 | orange |
| PCDHGB2 | orange |
| PCDHGB4 | orange |
| PCDHGB6 | orange |
| PCDHGB7 | orange |
| PCDHGB8P | orange |
| PCDHGC3 | blue |
| PCGF1 | blue |
| PCGF2 | yellow |
| PCGF3 | yellow |
| PCGF5 | turquoise |
| PCID2 | blue |
| PCIF1 | yellow |
| PCK2 | blue |
| PCLO | orange |
| PCM1 | turquoise |
| PCMT1 | green |
| PCMTD1 | midnightblue |
| PCMTD2 | black |
| PCNA | green |
| PCNT | yellow |
| PCNX | turquoise |
| PCNXL2 | turquoise |
| PCNXL3 | yellow |
| PCOLCE | purple |
| PCOLCE2 | brown |
| PCP2 | brown |
| PCSK1N | brown |
| PCSK5 | blue |
| PCSK6 | brown |
| PCSK7 | turquoise |
| PCTK3 | magenta |
| PCTP | grey60 |
| PCYOX1 | turquoise |
| PCYOX1L | turquoise |
| PCYT1A | blue |
| PCYT2 | grey60 |
| PDAP1 | blue |
| PDCD1 | turquoise |
| PDCD10 | green |
| PDCD11 | lightgreen |
| PDCD1LG2 | turquoise |
| PDCD2 | midnightblue |
| PDCD2L | blue |
| PDCD4 | black |
| PDCD5 | blue |
| PDCD6 | turquoise |
| PDCL | turquoise |
| PDCL3 | green |
| PDDC1 | brown |
| PDE1A | black |
| PDE1B | brown |
| PDE1C | blue |
| PDE3A | blue |
| PDE3B | turquoise |
| PDE4A | brown |
| PDE4B | turquoise |
| PDE4D | blue |
| PDE4DIP | brown |
| PDE5A | blue |
| PDE6B | turquoise |
| PDE7A | midnightblue |
| PDE8A | blue |
| PDE8B | blue |
| PDE9A | magenta |
| PDGFA | blue |
| PDGFB | blue |
| PDGFC | blue |
| PDGFD | black |
| PDGFRA | blue |
| PDGFRB | purple |
| PDGFRL | blue |
| PDHA1 | blue |
| PDHX | red |
| PDIA3 | turquoise |
| PDIA4 | blue |
| PDIA5 | turquoise |
| PDIA6 | blue |
| PDIK1L | blue |
| PDK1 | turquoise |
| PDK2 | turquoise |
| PDK3 | brown |
| PDK4 | black |
| PDLIM1 | blue |
| PDLIM2 | blue |
| PDLIM3 | blue |
| PDLIM4 | blue |
| PDLIM5 | blue |
| PDLIM7 | purple |
| PDP1 | tan |
| PDPN | purple |
| PDPR | yellow |
| PDRG1 | blue |
| PDS5B | blue |
| PDSS1 | green |
| PDSS2 | red |
| PDXDC1 | turquoise |
| PDXDC2P | yellow |
| PDXK | green |
| PDXP | blue |
| PDZD2 | turquoise |
| PDZD8 | red |
| PDZK1 | blue |
| PDZK1IP1 | turquoise |
| PDZRN3 | blue |
| PEA15 | blue |
| PEBP1 | turquoise |
| PECI | blue |
| PECR | turquoise |
| PEG10 | brown |
| PELI1 | turquoise |
| PELI2 | black |
| PELI3 | brown |
| PELO | brown |
| PELP1 | yellow |
| PEMT | salmon |
| PEPD | brown |
| PER1 | blue |
| PER2 | blue |
| PER3 | blue |
| PERP | turquoise |
| PES1 | blue |
| PEX1 | yellow |
| PEX10 | turquoise |
| PEX11A | turquoise |
| PEX11B | lightgreen |
| PEX11G | brown |
| PEX13 | red |
| PEX14 | blue |
| PEX16 | blue |
| PEX19 | lightgreen |
| PEX2 | midnightblue |
| PEX26 | yellow |
| PEX3 | pink |
| PEX5 | cyan |
| PEX6 | magenta |
| PEX7 | brown |
| PFAS | yellow |
| PFDN2 | lightgreen |
| PFDN4 | green |
| PFDN5 | brown |
| PFDN6 | magenta |
| PFKFB1 | darkgreen |
| PFKFB2 | blue |
| PFKFB3 | blue |
| PFKFB4 | turquoise |
| PFKL | blue |
| PFKM | blue |
| PFKP | blue |
| PFN1 | brown |
| PFN2 | turquoise |
| PGAM1 | turquoise |
| PGAM2 | yellow |
| PGAP1 | blue |
| PGAP2 | brown |
| PGAP3 | blue |
| PGBD1 | blue |
| PGBD2 | red |
| PGBD5 | blue |
| PGCP | blue |
| PGD | blue |
| PGF | black |
| PGK1 | turquoise |
| PGLS | turquoise |
| PGM1 | turquoise |
| PGM2 | turquoise |
| PGM2L1 | purple |
| PGM3 | yellow |
| PGM5 | black |
| PGM5P1 | blue |
| PGPEP1 | turquoise |
| PGRMC1 | turquoise |
| PGRMC2 | yellow |
| PGS1 | turquoise |
| PHACTR1 | turquoise |
| PHACTR2 | blue |
| PHACTR4 | yellow |
| PHB | grey60 |
| PHB2 | cyan |
| PHC1 | cyan |
| PHC2 | blue |
| PHC3 | turquoise |
| PHF1 | magenta |
| PHF10 | pink |
| PHF11 | turquoise |
| PHF12 | yellow |
| PHF14 | green |
| PHF15 | turquoise |
| PHF16 | brown |
| PHF17 | blue |
| PHF19 | turquoise |
| PHF20 | blue |
| PHF20L1 | midnightblue |
| PHF5A | blue |
| PHF6 | red |
| PHF7 | turquoise |
| PHF8 | yellow |
| PHGDH | blue |
| PHKA1 | brown |
| PHKA2 | yellow |
| PHKB | yellow |
| PHKG1 | brown |
| PHKG2 | blue |
| PHLDA1 | blue |
| PHLDA2 | turquoise |
| PHLDA3 | blue |
| PHLDB1 | blue |
| PHLDB2 | blue |
| PHLDB3 | yellow |
| PHLPP1 | brown |
| PHLPP2 | yellow |
| PHOSPHO1 | turquoise |
| PHOSPHO2 | red |
| PHPT1 | brown |
| PHRF1 | yellow |
| PHTF1 | turquoise |
| PHYH | turquoise |
| PHYHD1 | blue |
| PHYHIP | black |
| PI15 | blue |
| PI16 | black |
| PI3 | brown |
| PI4K2B | red |
| PI4KA | yellow |
| PI4KAP1 | yellow |
| PI4KAP2 | yellow |
| PIAS1 | turquoise |
| PIAS2 | red |
| PIAS3 | yellow |
| PIAS4 | yellow |
| PIBF1 | blue |
| PICK1 | brown |
| PID1 | blue |
| PIF1 | green |
| PIGA | turquoise |
| PIGC | green |
| PIGG | turquoise |
| PIGK | red |
| PIGL | blue |
| PIGM | lightgreen |
| PIGN | red |
| PIGO | brown |
| PIGP | blue |
| PIGQ | yellow |
| PIGR | black |
| PIGS | yellow |
| PIGT | yellow |
| PIGU | brown |
| PIGV | blue |
| PIGW | green |
| PIGX | green |
| PIGY | blue |
| PIGZ | yellow |
| PIK3AP1 | turquoise |
| PIK3C2A | red |
| PIK3C2B | yellow |
| PIK3C2G | blue |
| PIK3CA | blue |
| PIK3CB | yellow |
| PIK3CD | turquoise |
| PIK3CG | turquoise |
| PIK3IP1 | turquoise |
| PIK3R1 | blue |
| PIK3R2 | greenyellow |
| PIK3R3 | brown |
| PIK3R5 | turquoise |
| PIK3R6 | brown |
| PILRA | brown |
| PILRB | yellow |
| PIM1 | turquoise |
| PIM2 | turquoise |
| PIM3 | blue |
| PIN1 | blue |
| PIN4 | greenyellow |
| PINK1 | blue |
| PINX1 | greenyellow |
| PION | turquoise |
| PIP | tan |
| PIP4K2C | brown |
| PIP5K1C | yellow |
| PIP5K2A | turquoise |
| PIP5K2B | turquoise |
| PIP5KL1 | brown |
| PIR | blue |
| PISD | blue |
| PITPNA | yellow |
| PITPNB | yellow |
| PITPNC1 | turquoise |
| PITPNM1 | turquoise |
| PITPNM2 | magenta |
| PITRM1 | lightgreen |
| PITX1 | brown |
| PIWIL4 | turquoise |
| PJA1 | blue |
| PK4P | brown |
| PKD1 | yellow |
| PKD1P6 | yellow |
| PKD2 | black |
| PKD2L1 | brown |
| PKDCC | black |
| PKIB | turquoise |
| PKIG | blue |
| PKM2 | turquoise |
| PKMYT1 | green |
| PKN1 | turquoise |
| PKN2 | pink |
| PKN3 | blue |
| PKP1 | brown |
| PKP2 | cyan |
| PKP3 | black |
| PLA2G12A | brown |
| PLA2G15 | brown |
| PLA2G16 | blue |
| PLA2G2A | black |
| PLA2G4A | blue |
| PLA2G4B | turquoise |
| PLA2G4C | turquoise |
| PLA2G4F | turquoise |
| PLA2G5 | blue |
| PLA2G6 | yellow |
| PLA2G7 | turquoise |
| PLA2R1 | blue |
| PLAA | green |
| PLAC2 | turquoise |
| PLAC8 | turquoise |
| PLAC9 | black |
| PLAG1 | blue |
| PLAGL1 | blue |
| PLAGL2 | yellow |
| PLAT | blue |
| PLAU | lightcyan |
| PLAUR | brown |
| PLB1 | blue |
| PLBD1 | brown |
| PLBD2 | lightyellow |
| PLCB1 | blue |
| PLCB2 | turquoise |
| PLCB3 | turquoise |
| PLCB4 | brown |
| PLCD1 | blue |
| PLCD3 | blue |
| PLCE1 | yellow |
| PLCG1 | yellow |
| PLCG2 | turquoise |
| PLCH1 | green |
| PLCH2 | turquoise |
| PLCL2 | turquoise |
| PLCXD1 | blue |
| PLD1 | blue |
| PLD2 | yellow |
| PLD3 | brown |
| PLD4 | turquoise |
| PLD6 | turquoise |
| PLEC | lightcyan |
| PLEK | turquoise |
| PLEK2 | turquoise |
| PLEKHA1 | brown |
| PLEKHA2 | turquoise |
| PLEKHA4 | blue |
| PLEKHA5 | cyan |
| PLEKHA6 | brown |
| PLEKHA7 | yellow |
| PLEKHA8 | turquoise |
| PLEKHB1 | brown |
| PLEKHC1 | blue |
| PLEKHF1 | turquoise |
| PLEKHF2 | midnightblue |
| PLEKHG1 | blue |
| PLEKHG2 | lightyellow |
| PLEKHG3 | brown |
| PLEKHG4 | brown |
| PLEKHG4B | magenta |
| PLEKHG5 | blue |
| PLEKHG6 | cyan |
| PLEKHH1 | brown |
| PLEKHH2 | blue |
| PLEKHH3 | brown |
| PLEKHJ1 | blue |
| PLEKHM1P | grey60 |
| PLEKHM3 | turquoise |
| PLEKHN1 | brown |
| PLEKHO1 | turquoise |
| PLEKHO2 | brown |
| PLGLB1 | turquoise |
| PLIN1 | black |
| PLIN2 | brown |
| PLIN3 | tan |
| PLIN4 | black |
| PLIN5 | blue |
| PLK1 | green |
| PLK1S1 | turquoise |
| PLK2 | blue |
| PLK3 | brown |
| PLK4 | green |
| PLLP | brown |
| PLN | blue |
| PLOD1 | turquoise |
| PLOD2 | lightcyan |
| PLOD3 | yellow |
| PLP1 | blue |
| PLP2 | green |
| PLS1 | turquoise |
| PLS3 | lightcyan |
| PLSCR1 | turquoise |
| PLSCR3 | yellow |
| PLSCR4 | black |
| PLTP | brown |
| PLVAP | black |
| PLXDC1 | black |
| PLXDC2 | blue |
| PLXNA1 | yellow |
| PLXNA2 | blue |
| PLXNA3 | yellow |
| PLXNA4 | black |
| PLXNB1 | magenta |
| PLXNB2 | yellow |
| PLXNB3 | brown |
| PLXNC1 | brown |
| PLXND1 | lightyellow |
| PM20D2 | red |
| PMAIP1 | blue |
| PMEPA1 | turquoise |
| PMF1 | lightgreen |
| PML | turquoise |
| PMM1 | turquoise |
| PMM2 | brown |
| PMP22 | blue |
| PMPCA | green |
| PMS1 | pink |
| PMS2CL | yellow |
| PMS2L1 | yellow |
| PMS2L11 | yellow |
| PMS2L17 | yellow |
| PMS2L2 | yellow |
| PMS2L3 | yellow |
| PMS2L4 | brown |
| PMS2L5 | brown |
| PMVK | blue |
| PNCK | turquoise |
| PNKD | blue |
| PNKP | yellow |
| PNMA1 | blue |
| PNMA3 | turquoise |
| PNMAL1 | brown |
| PNN | grey60 |
| PNO1 | green |
| PNP | blue |
| PNPLA2 | blue |
| PNPLA3 | turquoise |
| PNPLA4 | blue |
| PNPLA6 | brown |
| PNPLA7 | blue |
| PNPO | blue |
| PNPT1 | green |
| PNRC1 | turquoise |
| POC1A | green |
| PODN | blue |
| PODNL1 | purple |
| PODXL | cyan |
| PODXL2 | blue |
| POFUT2 | yellow |
| POGK | lightgreen |
| POGZ | yellow |
| POLA1 | green |
| POLA2 | green |
| POLB | brown |
| POLD1 | green |
| POLD2 | green |
| POLD3 | green |
| POLD4 | brown |
| POLDIP2 | green |
| POLDIP3 | yellow |
| POLE | magenta |
| POLE2 | green |
| POLE3 | green |
| POLE4 | blue |
| POLG2 | grey60 |
| POLH | magenta |
| POLI | red |
| POLL | turquoise |
| POLM | blue |
| POLN | blue |
| POLQ | green |
| POLR1A | magenta |
| POLR1B | blue |
| POLR1C | magenta |
| POLR1E | blue |
| POLR2A | yellow |
| POLR2C | turquoise |
| POLR2F | blue |
| POLR2G | blue |
| POLR2H | blue |
| POLR2I | blue |
| POLR2J | blue |
| POLR2J2 | yellow |
| POLR2J3 | yellow |
| POLR2K | midnightblue |
| POLR2L | yellow |
| POLR3A | yellow |
| POLR3C | lightgreen |
| POLR3D | blue |
| POLR3F | brown |
| POLR3G | blue |
| POLR3GL | turquoise |
| POLR3K | green |
| POLRMT | blue |
| POM121 | yellow |
| POM121C | yellow |
| POM121L9P | turquoise |
| POMC | turquoise |
| POMGNT1 | yellow |
| POMP | brown |
| POMT1 | yellow |
| POMZP3 | yellow |
| PON2 | turquoise |
| PON3 | darkgreen |
| POP1 | green |
| POP4 | blue |
| POP5 | blue |
| POP7 | blue |
| POPDC2 | black |
| POR | blue |
| PORCN | blue |
| POSTN | purple |
| POT1 | brown |
| POTEI | yellow |
| POU2F2 | turquoise |
| POU2F3 | brown |
| POU5F1 | magenta |
| POU5F2 | turquoise |
| POU6F1 | black |
| PPA1 | blue |
| PPA2 | turquoise |
| PPAN | blue |
| PPAP2A | black |
| PPAP2B | black |
| PPAP2C | brown |
| PPAPDC1A | lightcyan |
| PPAPDC1B | turquoise |
| PPAPDC2 | turquoise |
| PPAPDC3 | black |
| PPARA | yellow |
| PPARD | yellow |
| PPARG | black |
| PPARGC1B | turquoise |
| PPAT | green |
| PPCDC | yellow |
| PPCS | blue |
| PPDPF | turquoise |
| PPEF1 | turquoise |
| PPEF2 | royalblue |
| PPFIA1 | yellow |
| PPFIA3 | turquoise |
| PPFIA4 | turquoise |
| PPFIBP1 | lightcyan |
| PPFIBP2 | blue |
| PPIA | turquoise |
| PPIAL3 | turquoise |
| PPIAP11 | turquoise |
| PPIAP19 | turquoise |
| PPIAP2 | turquoise |
| PPIB | brown |
| PPIC | lightcyan |
| PPID | red |
| PPIE | green |
| PPIF | blue |
| PPIH | green |
| PPIL1 | green |
| PPIL2 | yellow |
| PPIL5 | darkturquoise |
| PPIL6 | red |
| PPIP5K1 | yellow |
| PPL | brown |
| PPM1D | grey60 |
| PPM1F | blue |
| PPM1G | green |
| PPM1H | turquoise |
| PPM1J | blue |
| PPM1K | turquoise |
| PPM1L | brown |
| PPM1M | turquoise |
| PPM1N | brown |
| PPME1 | turquoise |
| PPOX | lightgreen |
| PPP1CB | turquoise |
| PPP1R10 | yellow |
| PPP1R11 | magenta |
| PPP1R12B | pink |
| PPP1R12C | turquoise |
| PPP1R13B | yellow |
| PPP1R13L | turquoise |
| PPP1R14A | blue |
| PPP1R14B | turquoise |
| PPP1R14BP3 | yellow |
| PPP1R14C | blue |
| PPP1R15A | blue |
| PPP1R16A | darkred |
| PPP1R16B | turquoise |
| PPP1R1B | brown |
| PPP1R2 | turquoise |
| PPP1R3B | turquoise |
| PPP1R3C | blue |
| PPP1R3D | blue |
| PPP1R3F | yellow |
| PPP1R9A | brown |
| PPP1R9B | turquoise |
| PPP2CB | turquoise |
| PPP2R1B | red |
| PPP2R2A | yellow |
| PPP2R3A | brown |
| PPP2R3B | magenta |
| PPP2R4 | turquoise |
| PPP2R5A | midnightblue |
| PPP2R5C | turquoise |
| PPP2R5D | magenta |
| PPP3CA | red |
| PPP3CB | turquoise |
| PPP3CC | turquoise |
| PPP3R1 | red |
| PPP4C | blue |
| PPP4R1 | turquoise |
| PPP4R1L | turquoise |
| PPP5C | yellow |
| PPP6C | red |
| PPP6R1 | turquoise |
| PPPDE1 | green |
| PPPDE2 | yellow |
| PPRC1 | yellow |
| PPT1 | brown |
| PPT2 | magenta |
| PPWD1 | turquoise |
| PQBP1 | blue |
| PQLC1 | blue |
| PQLC3 | brown |
| PRAF2 | lightyellow |
| PRAGMIN | yellow |
| PRAME | yellow |
| PRC1 | green |
| PRCC | lightgreen |
| PRCP | turquoise |
| PRDM1 | turquoise |
| PRDM2 | turquoise |
| PRDM5 | blue |
| PRDX1 | green |
| PRDX2 | blue |
| PRDX3 | greenyellow |
| PRDX4 | blue |
| PRDX5 | turquoise |
| PRDX6 | blue |
| PRELID1 | blue |
| PRELID2 | blue |
| PRELP | blue |
| PREP | red |
| PREPL | pink |
| PREX1 | turquoise |
| PREX2 | black |
| PRF1 | turquoise |
| PRH1 | cyan |
| PRH2 | cyan |
| PRICKLE1 | blue |
| PRICKLE2 | blue |
| PRICKLE3 | blue |
| PRICKLE4 | blue |
| PRIM1 | green |
| PRIM2 | green |
| PRINS | turquoise |
| PRKAA1 | red |
| PRKAA2 | turquoise |
| PRKAB2 | turquoise |
| PRKACA | yellow |
| PRKACB | turquoise |
| PRKAG2 | yellow |
| PRKAR1A | grey60 |
| PRKAR1B | blue |
| PRKAR2A | turquoise |
| PRKAR2B | blue |
| PRKCA | blue |
| PRKCB | turquoise |
| PRKCD | brown |
| PRKCDBP | blue |
| PRKCE | blue |
| PRKCH | turquoise |
| PRKCI | brown |
| PRKCSH | yellow |
| PRKCZ | brown |
| PRKD1 | blue |
| PRKD2 | turquoise |
| PRKD3 | turquoise |
| PRKDC | blue |
| PRKG1 | blue |
| PRKRA | brown |
| PRKRIP1 | yellow |
| PRKRIR | brown |
| PRKX | red |
| PRLR | brown |
| PRMT1 | blue |
| PRMT2 | pink |
| PRMT3 | red |
| PRMT5 | turquoise |
| PRMT6 | blue |
| PRNP | brown |
| PROC | turquoise |
| PROCA1 | turquoise |
| PROCR | brown |
| PRODH | yellow |
| PROM1 | brown |
| PROM2 | brown |
| PROS1 | black |
| PROSC | turquoise |
| PRPF18 | brown |
| PRPF19 | green |
| PRPF3 | lightgreen |
| PRPF38A | green |
| PRPF40B | yellow |
| PRPF4B | pink |
| PRPF8 | yellow |
| PRPS1 | brown |
| PRPS2 | green |
| PRPSAP1 | grey60 |
| PRR11 | green |
| PRR12 | yellow |
| PRR13 | brown |
| PRR14 | yellow |
| PRR15L | brown |
| PRR16 | blue |
| PRR19 | brown |
| PRR22 | blue |
| PRR24 | blue |
| PRR3 | magenta |
| PRR4 | brown |
| PRR5 | yellow |
| PRR5L | turquoise |
| PRR7 | turquoise |
| PRRC1 | red |
| PRRG1 | brown |
| PRRG2 | brown |
| PRRG4 | turquoise |
| PRRT2 | turquoise |
| PRRT3 | yellow |
| PRRX1 | blue |
| PRRX2 | lightcyan |
| PRSS12 | turquoise |
| PRSS16 | brown |
| PRSS22 | turquoise |
| PRSS27 | turquoise |
| PRSS50 | brown |
| PRSS53 | magenta |
| PRSS8 | brown |
| PRTFDC1 | turquoise |
| PRUNE | lightgreen |
| PRUNE2 | blue |
| PRX | blue |
| PSAP | brown |
| PSAT1 | green |
| PSCA | turquoise |
| PSD | blue |
| PSD3 | purple |
| PSD4 | turquoise |
| PSEN2 | lightgreen |
| PSENEN | turquoise |
| PSIP1 | turquoise |
| PSKH1 | yellow |
| PSMA1 | red |
| PSMA2 | blue |
| PSMA3 | blue |
| PSMA4 | green |
| PSMA5 | blue |
| PSMA6 | blue |
| PSMA7 | blue |
| PSMB1 | blue |
| PSMB10 | turquoise |
| PSMB2 | green |
| PSMB3 | blue |
| PSMB4 | lightgreen |
| PSMB5 | turquoise |
| PSMB6 | blue |
| PSMB7 | blue |
| PSMB8 | turquoise |
| PSMB9 | turquoise |
| PSMC1 | blue |
| PSMC3 | blue |
| PSMC3IP | green |
| PSMC4 | blue |
| PSMC5 | grey60 |
| PSMD10 | blue |
| PSMD10P | yellow |
| PSMD11 | green |
| PSMD12 | grey60 |
| PSMD13 | blue |
| PSMD14 | green |
| PSMD2 | blue |
| PSMD3 | green |
| PSMD4 | lightgreen |
| PSMD5 | red |
| PSMD8 | turquoise |
| PSMD9 | blue |
| PSME1 | turquoise |
| PSME2 | turquoise |
| PSME3 | yellow |
| PSME4 | brown |
| PSMF1 | yellow |
| PSMG1 | green |
| PSMG2 | blue |
| PSMG3 | blue |
| PSMG4 | magenta |
| PSORS1C1 | turquoise |
| PSPH | brown |
| PSRC1 | green |
| PSTPIP1 | turquoise |
| PSTPIP2 | turquoise |
| PTAFR | turquoise |
| PTAR1 | pink |
| PTBP2 | pink |
| PTCD1 | blue |
| PTCH1 | yellow |
| PTCH2 | blue |
| PTDSS1 | midnightblue |
| PTDSS2 | blue |
| PTEN | turquoise |
| PTER | red |
| PTGDS | turquoise |
| PTGER2 | brown |
| PTGER3 | black |
| PTGER4 | turquoise |
| PTGES | blue |
| PTGES2 | blue |
| PTGFR | black |
| PTGFRN | purple |
| PTGIR | brown |
| PTGIS | blue |
| PTGR1 | blue |
| PTGR2 | yellow |
| PTGS1 | brown |
| PTGS2 | blue |
| PTH1R | blue |
| PTH2R | blue |
| PTK2 | darkred |
| PTK2B | turquoise |
| PTK6 | tan |
| PTK7 | magenta |
| PTMA | green |
| PTMS | cyan |
| PTN | blue |
| PTOV1 | yellow |
| PTP4A1 | turquoise |
| PTP4A2 | brown |
| PTP4A3 | turquoise |
| PTPDC1 | blue |
| PTPLA | brown |
| PTPLAD1 | turquoise |
| PTPLAD2 | turquoise |
| PTPLB | turquoise |
| PTPMT1 | blue |
| PTPN1 | turquoise |
| PTPN12 | red |
| PTPN13 | blue |
| PTPN14 | turquoise |
| PTPN18 | yellow |
| PTPN2 | turquoise |
| PTPN21 | blue |
| PTPN22 | turquoise |
| PTPN23 | lightyellow |
| PTPN3 | brown |
| PTPN4 | yellow |
| PTPN6 | turquoise |
| PTPN7 | turquoise |
| PTPRA | yellow |
| PTPRB | black |
| PTPRC | turquoise |
| PTPRCAP | turquoise |
| PTPRD | purple |
| PTPRE | brown |
| PTPRF | brown |
| PTPRG | blue |
| PTPRJ | brown |
| PTPRK | red |
| PTPRM | blue |
| PTPRN2 | turquoise |
| PTPRO | brown |
| PTPRS | blue |
| PTPRT | blue |
| PTPRU | turquoise |
| PTPRZ1 | brown |
| PTRF | blue |
| PTRH1 | turquoise |
| PTRH2 | grey60 |
| PTS | turquoise |
| PTTG1 | green |
| PTTG1IP | turquoise |
| PTX3 | blue |
| PUF60 | darkred |
| PURA | blue |
| PURB | turquoise |
| PUS1 | blue |
| PUS10 | red |
| PUS7 | blue |
| PUS7L | red |
| PUSL1 | blue |
| PVR | turquoise |
| PVRIG | turquoise |
| PVRL1 | yellow |
| PVRL2 | yellow |
| PVRL3 | black |
| PVRL4 | brown |
| PVT1 | blue |
| PWP2 | blue |
| PWWP2B | blue |
| PXDN | lightcyan |
| PXK | turquoise |
| PXMP2 | blue |
| PXMP4 | tan |
| PXN | lightyellow |
| PYCARD | turquoise |
| PYCR1 | blue |
| PYCR2 | blue |
| PYCRL | darkred |
| PYGB | turquoise |
| PYGL | blue |
| PYGO2 | lightgreen |
| PYROXD1 | cyan |
| PYROXD2 | blue |
| QDPR | yellow |
| QKI | blue |
| QPCT | turquoise |
| QPCTL | blue |
| QPRT | tan |
| QRICH2 | grey60 |
| QRSL1 | red |
| QSER1 | red |
| QSOX1 | turquoise |
| QTRT1 | blue |
| R3HCC1 | turquoise |
| R3HDM1 | yellow |
| R3HDM2 | yellow |
| RAB10 | blue |
| RAB11A | turquoise |
| RAB11B | yellow |
| RAB11FIP1 | yellow |
| RAB11FIP2 | blue |
| RAB11FIP3 | yellow |
| RAB11FIP4 | blue |
| RAB11FIP5 | turquoise |
| RAB12 | green |
| RAB13 | brown |
| RAB14 | red |
| RAB15 | yellow |
| RAB17 | brown |
| RAB18 | turquoise |
| RAB19 | turquoise |
| RAB1A | red |
| RAB1B | greenyellow |
| RAB20 | brown |
| RAB23 | turquoise |
| RAB24 | brown |
| RAB25 | brown |
| RAB26 | brown |
| RAB27A | turquoise |
| RAB27B | brown |
| RAB28 | turquoise |
| RAB2A | midnightblue |
| RAB2B | turquoise |
| RAB30 | turquoise |
| RAB31 | lightcyan |
| RAB32 | brown |
| RAB33B | red |
| RAB34 | blue |
| RAB36 | yellow |
| RAB38 | brown |
| RAB39 | brown |
| RAB3B | darkgreen |
| RAB3D | brown |
| RAB3IL1 | lightyellow |
| RAB3IP | brown |
| RAB40B | grey60 |
| RAB40C | brown |
| RAB42 | brown |
| RAB43 | blue |
| RAB4A | brown |
| RAB4B | turquoise |
| RAB5A | yellow |
| RAB5B | yellow |
| RAB6A | red |
| RAB6B | turquoise |
| RAB7A | yellow |
| RAB7L1 | turquoise |
| RAB8A | turquoise |
| RAB8B | turquoise |
| RAB9A | red |
| RABAC1 | greenyellow |
| RABEP1 | blue |
| RABEP2 | tan |
| RABEPK | blue |
| RABGAP1 | red |
| RABGAP1L | turquoise |
| RABGEF1 | red |
| RABGGTB | salmon |
| RABL2A | yellow |
| RABL2B | yellow |
| RABL3 | blue |
| RABL4 | blue |
| RABL5 | brown |
| RAC1 | greenyellow |
| RAC2 | turquoise |
| RAC3 | blue |
| RACGAP1 | green |
| RAD1 | turquoise |
| RAD18 | turquoise |
| RAD21 | midnightblue |
| RAD23A | greenyellow |
| RAD23B | turquoise |
| RAD51 | green |
| RAD51AP1 | cyan |
| RAD51C | grey60 |
| RAD51L1 | turquoise |
| RAD51L3 | turquoise |
| RAD52 | cyan |
| RAD54B | midnightblue |
| RAD54L | green |
| RAD54L2 | yellow |
| RAD9A | yellow |
| RAE1 | green |
| RAF1 | yellow |
| RAG1AP1 | blue |
| RAGE | brown |
| RAI1 | yellow |
| RAI14 | purple |
| RAI2 | black |
| RALA | purple |
| RALB | lightcyan |
| RALBP1 | brown |
| RALGAPA1 | red |
| RALGAPA2 | blue |
| RALGDS | turquoise |
| RALGPS1 | yellow |
| RALGPS2 | turquoise |
| RAMP1 | turquoise |
| RAMP2 | black |
| RAMP3 | turquoise |
| RAN | green |
| RANBP1 | blue |
| RANBP10 | yellow |
| RANBP17 | brown |
| RANBP3L | darkgreen |
| RANBP6 | greenyellow |
| RANBP9 | magenta |
| RANGAP1 | blue |
| RANGRF | turquoise |
| RAP1B | brown |
| RAP1GAP | brown |
| RAP1GAP2 | magenta |
| RAP2A | green |
| RAP2B | brown |
| RAP2C | brown |
| RAPGEF1 | turquoise |
| RAPGEF3 | black |
| RAPGEF4 | blue |
| RAPGEF5 | blue |
| RAPGEF6 | turquoise |
| RAPGEFL1 | yellow |
| RAPH1 | turquoise |
| RARA | lightyellow |
| RARB | turquoise |
| RARG | brown |
| RARRES1 | brown |
| RARRES2 | purple |
| RARRES3 | turquoise |
| RASA2 | turquoise |
| RASA3 | turquoise |
| RASA4 | turquoise |
| RASA4B | turquoise |
| RASAL1 | brown |
| RASAL2 | yellow |
| RASAL3 | turquoise |
| RASD1 | blue |
| RASD2 | brown |
| RASGEF1A | cyan |
| RASGEF1B | turquoise |
| RASGRF1 | blue |
| RASGRF2 | blue |
| RASGRP1 | turquoise |
| RASGRP2 | turquoise |
| RASGRP3 | turquoise |
| RASIP1 | blue |
| RASL10A | blue |
| RASL11A | black |
| RASL11B | blue |
| RASL12 | blue |
| RASSF1 | turquoise |
| RASSF10 | purple |
| RASSF2 | turquoise |
| RASSF3 | blue |
| RASSF4 | brown |
| RASSF5 | turquoise |
| RASSF6 | turquoise |
| RASSF7 | brown |
| RASSF8 | blue |
| RASSF9 | blue |
| RAVER1 | yellow |
| RAVER2 | brown |
| RB1 | brown |
| RB1CC1 | midnightblue |
| RBBP4 | yellow |
| RBBP7 | green |
| RBBP8 | red |
| RBBP9 | brown |
| RBCK1 | turquoise |
| RBL1 | red |
| RBL2 | turquoise |
| RBM12 | greenyellow |
| RBM12B | midnightblue |
| RBM16 | pink |
| RBM17 | green |
| RBM18 | red |
| RBM19 | yellow |
| RBM26 | turquoise |
| RBM28 | green |
| RBM3 | greenyellow |
| RBM34 | green |
| RBM38 | blue |
| RBM42 | blue |
| RBM43 | turquoise |
| RBM47 | brown |
| RBM4B | pink |
| RBM5 | turquoise |
| RBM6 | turquoise |
| RBM8A | lightgreen |
| RBM9 | turquoise |
| RBMS1 | blue |
| RBMS1P1 | brown |
| RBMS2 | blue |
| RBMS3 | black |
| RBMX2 | green |
| RBP1 | brown |
| RBP5 | turquoise |
| RBP7 | blue |
| RBPJ | turquoise |
| RBPMS | blue |
| RBX1 | yellow |
| RCAN1 | blue |
| RCAN2 | black |
| RCAN3 | yellow |
| RCBTB1 | turquoise |
| RCBTB2 | brown |
| RCC1 | green |
| RCC2 | green |
| RCCD1 | blue |
| RCE1 | turquoise |
| RCL1 | blue |
| RCN1 | red |
| RCN2 | turquoise |
| RCN3 | blue |
| RCOR1 | yellow |
| RCOR2 | brown |
| RCOR3 | yellow |
| RCSD1 | turquoise |
| RDBP | magenta |
| RDH10 | blue |
| RDH11 | midnightblue |
| RDH13 | brown |
| RDH5 | black |
| RDX | red |
| REC8 | turquoise |
| RECK | black |
| RECQL | cyan |
| RECQL4 | darkred |
| RECQL5 | yellow |
| REEP1 | blue |
| REEP3 | purple |
| REEP4 | blue |
| REEP5 | tan |
| REEP6 | tan |
| REL | turquoise |
| RELA | yellow |
| RELB | turquoise |
| RELL1 | blue |
| RELL2 | turquoise |
| RELT | turquoise |
| RENBP | brown |
| REPIN1 | blue |
| REPS1 | pink |
| REPS2 | yellow |
| RER1 | tan |
| RERE | yellow |
| RERG | blue |
| RETSAT | tan |
| REXO1 | yellow |
| REXO2 | turquoise |
| RFC2 | green |
| RFC3 | green |
| RFC4 | green |
| RFC5 | green |
| RFFL | turquoise |
| RFK | red |
| RFNG | grey60 |
| RFTN1 | turquoise |
| RFTN2 | black |
| RFWD3 | green |
| RFX3 | turquoise |
| RFX5 | turquoise |
| RFX8 | blue |
| RFXANK | blue |
| RG9MTD1 | blue |
| RG9MTD2 | brown |
| RG9MTD3 | yellow |
| RGAG4 | black |
| RGL1 | turquoise |
| RGL2 | magenta |
| RGL3 | blue |
| RGL4 | turquoise |
| RGMA | yellow |
| RGMB | blue |
| RGN | brown |
| RGPD5 | blue |
| RGS1 | brown |
| RGS10 | brown |
| RGS11 | blue |
| RGS12 | yellow |
| RGS14 | turquoise |
| RGS16 | lightcyan |
| RGS18 | turquoise |
| RGS19 | turquoise |
| RGS2 | blue |
| RGS3 | lightcyan |
| RGS4 | blue |
| RGS5 | black |
| RHBDD1 | turquoise |
| RHBDD2 | turquoise |
| RHBDD3 | blue |
| RHBDF1 | brown |
| RHBDF2 | turquoise |
| RHBDL1 | turquoise |
| RHBDL2 | turquoise |
| RHCG | turquoise |
| RHEB | turquoise |
| RHEBL1 | turquoise |
| RHOA | lightcyan |
| RHOB | blue |
| RHOBTB1 | blue |
| RHOBTB2 | turquoise |
| RHOBTB3 | blue |
| RHOC | turquoise |
| RHOD | turquoise |
| RHOF | turquoise |
| RHOG | brown |
| RHOH | turquoise |
| RHOJ | black |
| RHOQ | blue |
| RHOU | black |
| RHOV | brown |
| RHPN1 | darkred |
| RHPN2 | brown |
| RIC3 | turquoise |
| RICTOR | turquoise |
| RILP | brown |
| RILPL1 | yellow |
| RILPL2 | turquoise |
| RIMKLB | cyan |
| RIMS2 | turquoise |
| RIMS3 | magenta |
| RIN1 | brown |
| RIN2 | lightcyan |
| RIN3 | brown |
| RING1 | magenta |
| RINL | turquoise |
| RIOK1 | green |
| RIOK3 | red |
| RIPK1 | blue |
| RIPK2 | blue |
| RIPK3 | turquoise |
| RIPK4 | brown |
| RIT1 | lightgreen |
| RLTPR | turquoise |
| RMI1 | green |
| RMND1 | pink |
| RMND5A | red |
| RMND5B | yellow |
| RN18S1 | red |
| RN28S1 | red |
| RNase_MRP | greenyellow |
| RNASE1 | black |
| RNASE10 | yellow |
| RNASE2 | brown |
| RNASE4 | blue |
| RNASE6 | turquoise |
| RNASEH2A | green |
| RNASEH2B | turquoise |
| RNASEK | brown |
| RNASEL | turquoise |
| RNASEN | greenyellow |
| RNaseP_nuc | yellow |
| RNASET2 | turquoise |
| RND1 | tan |
| RND3 | blue |
| RNF103 | red |
| RNF11 | turquoise |
| RNF114 | blue |
| RNF115 | lightgreen |
| RNF121 | magenta |
| RNF122 | turquoise |
| RNF125 | turquoise |
| RNF126 | blue |
| RNF128 | turquoise |
| RNF13 | brown |
| RNF130 | brown |
| RNF133 | blue |
| RNF135 | brown |
| RNF138 | red |
| RNF139 | midnightblue |
| RNF141 | blue |
| RNF144A | lightcyan |
| RNF144B | blue |
| RNF145 | brown |
| RNF146 | blue |
| RNF148 | blue |
| RNF150 | cyan |
| RNF152 | brown |
| RNF166 | turquoise |
| RNF167 | yellow |
| RNF168 | green |
| RNF169 | yellow |
| RNF170 | turquoise |
| RNF180 | blue |
| RNF187 | green |
| RNF19A | turquoise |
| RNF19B | brown |
| RNF2 | red |
| RNF207 | blue |
| RNF213 | turquoise |
| RNF215 | brown |
| RNF216L | yellow |
| RNF217 | blue |
| RNF220 | green |
| RNF24 | turquoise |
| RNF25 | turquoise |
| RNF26 | yellow |
| RNF32 | yellow |
| RNF38 | yellow |
| RNF39 | magenta |
| RNF43 | yellow |
| RNF44 | yellow |
| RNF5 | magenta |
| RNF7 | turquoise |
| RNF8 | green |
| RNFT1 | turquoise |
| RNFT2 | magenta |
| RNLS | blue |
| RNMT | green |
| RNPC3 | turquoise |
| RNPEP | yellow |
| RNPEPL1 | turquoise |
| RNPS1 | green |
| ROBLD3 | blue |
| ROBO1 | turquoise |
| ROBO3 | blue |
| ROBO4 | black |
| ROCK2 | pink |
| ROD1 | red |
| ROGDI | brown |
| ROM1 | blue |
| ROMO1 | yellow |
| ROPN1 | blue |
| ROPN1B | brown |
| ROR1 | blue |
| ROR2 | blue |
| RORA | turquoise |
| RORC | brown |
| RP1-102G20.3 | yellow |
| RP1-102G20.4 | turquoise |
| RP1-117B12.4 | grey60 |
| RP1-122O8.7 | red |
| RP1-122P22.2 | blue |
| RP1-128O3.6 | midnightblue |
| RP1-12G14.3 | pink |
| RP1-12G14.7 | greenyellow |
| RP1-136O14.1 | turquoise |
| RP1-151F17.1 | turquoise |
| RP1-152L7.5 | magenta |
| RP1-164F3.8 | blue |
| RP1-178F15.3 | magenta |
| RP1-179N16.3 | turquoise |
| RP1-184J9.2 | yellow |
| RP1-187N21.2 | blue |
| RP1-191J18.2 | red |
| RP1-199H16.5 | brown |
| RP1-199J3.3 | blue |
| RP1-20B11.2 | turquoise |
| RP1-20N2.6 | brown |
| RP1-21O18.1 | turquoise |
| RP1-222E13.10 | tan |
| RP1-224A6.4 | blue |
| RP1-224A6.6 | turquoise |
| RP1-228P16.1 | turquoise |
| RP1-234P15.4 | turquoise |
| RP1-238O23.4 | blue |
| RP1-238O23.5 | turquoise |
| RP1-245G19.2 | yellow |
| RP1-273P12.3 | brown |
| RP1-283E3.3 | turquoise |
| RP1-283E3.4 | turquoise |
| RP1-286D6.2 | brown |
| RP1-290I10.6 | magenta |
| RP1-29C18.10 | turquoise |
| RP1-29C18.8 | turquoise |
| RP1-308E4.1 | yellow |
| RP1-309F20.2 | yellow |
| RP1-315G1.1 | turquoise |
| RP1-315G1.4 | turquoise |
| RP1-317E23.3 | darkgrey |
| RP1-39G22.4 | brown |
| RP1-3J17.3 | pink |
| RP1-44A20.4 | blue |
| RP1-56L9.3 | yellow |
| RP1-67M12.1 | brown |
| RP1-69D17.4 | brown |
| RP1-71L16.1 | cyan |
| RP1-71L16.2 | brown |
| RP1-80B9.2 | turquoise |
| RP1-85F18.5 | turquoise |
| RP1-86D1.2 | red |
| RP1-86D1.3 | red |
| RP1-86D1.4 | red |
| RP1-86D1.5 | red |
| RP1-90K10.3 | blue |
| RP1-91J24.3 | blue |
| RP1-99E18.2 | red |
| RP1-9E21.5 | blue |
| RP11-1007J8.1 | yellow |
| RP11-101K10.8 | lightyellow |
| RP11-1023L17.1 | blue |
| RP11-1023L17.2 | yellow |
| RP11-102K13.3 | blue |
| RP11-102M11.1 | turquoise |
| RP11-103G8.2 | turquoise |
| RP11-103G8.4 | turquoise |
| RP11-103H7.5 | brown |
| RP11-106E7.1 | turquoise |
| RP11-1079K10.4 | blue |
| RP11-108K14.4 | turquoise |
| RP11-108K14.7 | turquoise |
| RP11-108M9.4 | blue |
| RP11-109P14.9 | blue |
| RP11-10A14.3 | turquoise |
| RP11-10A14.4 | brown |
| RP11-10J21.4 | yellow |
| RP11-10K16.1 | blue |
| RP11-10L12.2 | brown |
| RP11-10L12.6 | brown |
| RP11-10N23.2 | midnightblue |
| RP11-10N23.4 | midnightblue |
| RP11-110G21.1 | midnightblue |
| RP11-110K8.1 | turquoise |
| RP11-1114A5.5 | yellow |
| RP11-112J3.15 | brown |
| RP11-113A11.1 | blue |
| RP11-1148L6.5 | yellow |
| RP11-1149O23.2 | turquoise |
| RP11-1149O23.3 | turquoise |
| RP11-114M11.2 | pink |
| RP11-115C21.2 | blue |
| RP11-115C21.4 | turquoise |
| RP11-115P21.1 | turquoise |
| RP11-117F22.1 | blue |
| RP11-119F19.2 | yellow |
| RP11-119F19.4 | blue |
| RP11-119F7.4 | brown |
| RP11-11M20.2 | yellow |
| RP11-120J20.2 | greenyellow |
| RP11-1220K2.2 | purple |
| RP11-122C9.1 | salmon |
| RP11-122F14.2 | turquoise |
| RP11-122F14.3 | turquoise |
| RP11-122G18.5 | brown |
| RP11-122K13.12 | turquoise |
| RP11-123J14.2 | blue |
| RP11-124A7.1 | turquoise |
| RP11-124N14.3 | darkgrey |
| RP11-124N14.4 | magenta |
| RP11-125B21.2 | brown |
| RP11-126K1.8 | turquoise |
| RP11-1277A3.1 | yellow |
| RP11-1277A3.2 | turquoise |
| RP11-127L20.3 | lightyellow |
| RP11-1280I22.1 | turquoise |
| RP11-1280N14.4 | turquoise |
| RP11-129B22.6 | black |
| RP11-12A16.3 | turquoise |
| RP11-12A20.5 | blue |
| RP11-131L23.1 | blue |
| RP11-132A1.4 | blue |
| RP11-132G19.3 | turquoise |
| RP11-1334A24.4 | turquoise |
| RP11-133M8.1 | turquoise |
| RP11-135A24.4 | salmon |
| RP11-135J2.2 | red |
| RP11-135J2.3 | red |
| RP11-136K14.1 | turquoise |
| RP11-136K14.3 | turquoise |
| RP11-1379J22.3 | blue |
| RP11-137L10.6 | lightyellow |
| RP11-1396O13.18 | brown |
| RP11-139J15.2 | turquoise |
| RP11-139K1.2 | yellow |
| RP11-139O18.1 | salmon |
| RP11-13J17.1 | turquoise |
| RP11-1407O15.2 | yellow |
| RP11-140B16.2 | green |
| RP11-141M1.4 | blue |
| RP11-142M10.2 | brown |
| RP11-145J3.3 | turquoise |
| RP11-146N23.4 | salmon |
| RP11-147I3.1 | yellow |
| RP11-148B6.1 | blue |
| RP11-148K1.10 | yellow |
| RP11-148K1.12 | blue |
| RP11-149B7.3 | red |
| RP11-14C22.6 | blue |
| RP11-14D22.5 | black |
| RP11-14N7.2 | blue |
| RP11-153G4.2 | turquoise |
| RP11-154D6.1 | purple |
| RP11-154H23.1 | blue |
| RP11-154P18.1 | brown |
| RP11-155G14.5 | turquoise |
| RP11-156F12.1 | turquoise |
| RP11-156G14.5 | blue |
| RP11-156K13.1 | brown |
| RP11-157P1.4 | brown |
| RP11-158K1.3 | midnightblue |
| RP11-159F24.1 | yellow |
| RP11-159F24.4 | brown |
| RP11-159F24.6 | darkgrey |
| RP11-159G9.5 | midnightblue |
| RP11-15K19.2 | brown |
| RP11-160H22.1 | yellow |
| RP11-162A12.1 | red |
| RP11-162G10.5 | turquoise |
| RP11-163E9.2 | yellow |
| RP11-163O17.1 | yellow |
| RP11-164H5.1 | brown |
| RP11-166O4.1 | yellow |
| RP11-166O4.5 | yellow |
| RP11-168J18.4 | turquoise |
| RP11-169L17.2 | yellow |
| RP11-170J3.2 | turquoise |
| RP11-172F4.5 | blue |
| RP11-175B9.2 | brown |
| RP11-175F9.1 | yellow |
| RP11-175F9.2 | yellow |
| RP11-175F9.3 | yellow |
| RP11-175I17.4 | blue |
| RP11-176F3.7 | blue |
| RP11-176F3.8 | green |
| RP11-176F3.9 | turquoise |
| RP11-177A2.4 | blue |
| RP11-177H13.2 | lightcyan |
| RP11-178A10.1 | cyan |
| RP11-178C10.2 | turquoise |
| RP11-179B15.2 | turquoise |
| RP11-179G5.4 | yellow |
| RP11-179H18.2 | blue |
| RP11-181D10.2 | turquoise |
| RP11-182B22.2 | red |
| RP11-182I10.1 | turquoise |
| RP11-182I10.2 | turquoise |
| RP11-182I10.3 | black |
| RP11-182I10.4 | blue |
| RP11-182L21.2 | blue |
| RP11-183E9.3 | yellow |
| RP11-184I16.2 | yellow |
| RP11-187C18.2 | black |
| RP11-188C12.2 | turquoise |
| RP11-188P20.2 | blue |
| RP11-18B3.3 | blue |
| RP11-18H7.1 | brown |
| RP11-18I14.7 | brown |
| RP11-190J1.7 | turquoise |
| RP11-192K2.2 | blue |
| RP11-192P3.1 | turquoise |
| RP11-193I22.1 | turquoise |
| RP11-195E11.3 | blue |
| RP11-195F19.5 | brown |
| RP11-195F19.9 | turquoise |
| RP11-196I2.1 | blue |
| RP11-19G24.1 | tan |
| RP11-1A15.2 | turquoise |
| RP11-1E1.1 | turquoise |
| RP11-201O14.1 | pink |
| RP11-202D20.1 | yellow |
| RP11-204J18.3 | blue |
| RP11-206L10.11 | yellow |
| RP11-206L10.2 | yellow |
| RP11-206L10.3 | yellow |
| RP11-206L10.4 | yellow |
| RP11-206L10.5 | yellow |
| RP11-206L10.6 | salmon |
| RP11-206L10.7 | yellow |
| RP11-206L10.8 | turquoise |
| RP11-206L10.9 | turquoise |
| RP11-207C16.4 | turquoise |
| RP11-20L24.1 | turquoise |
| RP11-211A18.1 | turquoise |
| RP11-211G3.2 | blue |
| RP11-211G3.3 | blue |
| RP11-212F11.1 | turquoise |
| RP11-212P7.1 | yellow |
| RP11-213G2.3 | pink |
| RP11-213P13.1 | blue |
| RP11-214J9.1 | yellow |
| RP11-214O14.1 | blue |
| RP11-215B13.4 | turquoise |
| RP11-216L13.8 | yellow |
| RP11-216M21.1 | brown |
| RP11-216N14.9 | yellow |
| RP11-217B7.2 | brown |
| RP11-217E22.2 | brown |
| RP11-218L14.4 | turquoise |
| RP11-219B4.3 | blue |
| RP11-21N7.2 | blue |
| RP11-21N7.6 | yellow |
| RP11-220I1.1 | pink |
| RP11-220M1.1 | yellow |
| RP11-220M1.2 | turquoise |
| RP11-222A11.1 | brown |
| RP11-223D18.1 | turquoise |
| RP11-225L12.2 | royalblue |
| RP11-228B15.4 | turquoise |
| RP11-229P13.23 | blue |
| RP11-22B10.3 | turquoise |
| RP11-22C11.1 | midnightblue |
| RP11-230B22.1 | blue |
| RP11-230F18.2 | green |
| RP11-231D20.1 | yellow |
| RP11-233C13.1 | blue |
| RP11-234A1.1 | black |
| RP11-234K24.3 | brown |
| RP11-236F9.4 | brown |
| RP11-239C17.1 | brown |
| RP11-239L20.4 | brown |
| RP11-239L20.5 | magenta |
| RP11-23P13.4 | lightyellow |
| RP11-241J12.3 | turquoise |
| RP11-242C19.2 | brown |
| RP11-242O24.5 | blue |
| RP11-243J16.7 | turquoise |
| RP11-244H3.1 | turquoise |
| RP11-244H3.2 | brown |
| RP11-245J9.2 | turquoise |
| RP11-245J9.4 | blue |
| RP11-247A12.2 | turquoise |
| RP11-247I13.11 | yellow |
| RP11-247I13.3 | yellow |
| RP11-248C1.2 | turquoise |
| RP11-250B2.1 | turquoise |
| RP11-250B2.3 | turquoise |
| RP11-252I14.2 | blue |
| RP11-255C15.1 | yellow |
| RP11-255H23.2 | brown |
| RP11-255N4.2 | turquoise |
| RP11-255N4.3 | turquoise |
| RP11-256P1.1 | blue |
| RP11-258A12.3 | yellow |
| RP11-258C19.5 | yellow |
| RP11-259F16.3 | turquoise |
| RP11-259F4.1 | greenyellow |
| RP11-259O2.3 | black |
| RP11-25K21.1 | turquoise |
| RP11-261C10.3 | yellow |
| RP11-261C10.5 | brown |
| RP11-262H14.1 | turquoise |
| RP11-262H14.10 | yellow |
| RP11-262H14.4 | blue |
| RP11-262H14.5 | blue |
| RP11-262H14.6 | brown |
| RP11-263K19.4 | yellow |
| RP11-263K19.6 | turquoise |
| RP11-264F23.1 | brown |
| RP11-264I13.2 | turquoise |
| RP11-264J4.6 | blue |
| RP11-264J9.1 | blue |
| RP11-265B8.4 | brown |
| RP11-266J6.1 | blue |
| RP11-267J23.1 | cyan |
| RP11-267N12.3 | purple |
| RP11-271C24.3 | greenyellow |
| RP11-271K21.7 | brown |
| RP11-273G15.2 | blue |
| RP11-274B21.1 | yellow |
| RP11-274B21.2 | yellow |
| RP11-274B21.3 | yellow |
| RP11-274B21.4 | yellow |
| RP11-274H2.3 | lightcyan |
| RP11-274J7.1 | blue |
| RP11-275I14.4 | brown |
| RP11-277I20.2 | turquoise |
| RP11-277I20.3 | turquoise |
| RP11-277L2.2 | turquoise |
| RP11-277L2.3 | turquoise |
| RP11-278J6.1 | turquoise |
| RP11-278L15.1 | turquoise |
| RP11-278L15.2 | turquoise |
| RP11-27I1.2 | turquoise |
| RP11-27I1.4 | midnightblue |
| RP11-27I1.6 | blue |
| RP11-280O24.3 | yellow |
| RP11-281O15.3 | blue |
| RP11-281O15.7 | blue |
| RP11-284M14.1 | turquoise |
| RP11-285F7.2 | yellow |
| RP11-285G1.8 | pink |
| RP11-288G11.3 | turquoise |
| RP11-288H12.3 | turquoise |
| RP11-290D2.3 | blue |
| RP11-290F20.1 | blue |
| RP11-290F20.2 | blue |
| RP11-290F20.3 | brown |
| RP11-291L22.3 | yellow |
| RP11-292B8.1 | blue |
| RP11-293A21.2 | turquoise |
| RP11-294H11.4 | red |
| RP11-295G20.2 | blue |
| RP11-295K3.1 | purple |
| RP11-295P9.3 | yellow |
| RP11-296A18.5 | yellow |
| RP11-296A18.7 | yellow |
| RP11-296O14.3 | turquoise |
| RP11-297K8.2 | blue |
| RP11-298A18.1 | blue |
| RP11-298E9.3 | green |
| RP11-298J20.1 | turquoise |
| RP11-298J20.3 | blue |
| RP11-298J20.4 | yellow |
| RP11-298P3.4 | blue |
| RP11-29B11.4 | turquoise |
| RP11-29H23.1 | lightgreen |
| RP11-29H23.6 | yellow |
| RP11-2B6.1 | blue |
| RP11-2J18.1 | turquoise |
| RP11-2P5.4 | turquoise |
| RP11-301H24.4 | yellow |
| RP11-301L8.2 | turquoise |
| RP11-301M17.1 | blue |
| RP11-303G3.1 | turquoise |
| RP11-305E17.6 | blue |
| RP11-305I9.3 | turquoise |
| RP11-305M3.3 | blue |
| RP11-307B6.3 | black |
| RP11-307C12.11 | turquoise |
| RP11-307E17.8 | blue |
| RP11-308D16.4 | yellow |
| RP11-30L15.3 | midnightblue |
| RP11-30L4.5 | turquoise |
| RP11-311D14.1 | tan |
| RP11-312B8.2 | blue |
| RP11-312O7.2 | brown |
| RP11-313D6.4 | turquoise |
| RP11-313J2.1 | blue |
| RP11-315I20.1 | brown |
| RP11-316K19.3 | blue |
| RP11-316M21.5 | turquoise |
| RP11-316M24.1 | turquoise |
| RP11-317B17.2 | yellow |
| RP11-317B3.2 | turquoise |
| RP11-317F4.1 | turquoise |
| RP11-318M2.2 | turquoise |
| RP11-319G6.1 | brown |
| RP11-319J24.3 | yellow |
| RP11-31E23.1 | brown |
| RP11-31F15.1 | turquoise |
| RP11-31F19.1 | brown |
| RP11-321E8.2 | brown |
| RP11-321E8.5 | orange |
| RP11-321L2.2 | darkgrey |
| RP11-321N4.3 | turquoise |
| RP11-322M19.1 | blue |
| RP11-322M19.2 | yellow |
| RP11-323F5.2 | pink |
| RP11-324H6.6 | turquoise |
| RP11-325F22.3 | turquoise |
| RP11-325F22.4 | purple |
| RP11-325P15.3 | turquoise |
| RP11-326F20.5 | turquoise |
| RP11-326L2.1 | turquoise |
| RP11-328M4.2 | brown |
| RP11-32D17.4 | green |
| RP11-330C7.2 | blue |
| RP11-330L19.2 | blue |
| RP11-330M2.6 | blue |
| RP11-330M2.7 | yellow |
| RP11-331F4.4 | blue |
| RP11-332O19.2 | yellow |
| RP11-332O19.3 | turquoise |
| RP11-334A14.2 | darkgreen |
| RP11-334A14.3 | darkgreen |
| RP11-334A14.5 | blue |
| RP11-336A10.2 | blue |
| RP11-337A23.5 | yellow |
| RP11-337C18.5 | yellow |
| RP11-337C18.8 | lightgreen |
| RP11-338L22.1 | turquoise |
| RP11-339B21.9 | green |
| RP11-33B1.1 | black |
| RP11-33B1.3 | purple |
| RP11-342C23.4 | brown |
| RP11-342M1.3 | blue |
| RP11-342M1.4 | cyan |
| RP11-342M21.1 | blue |
| RP11-342M3.5 | black |
| RP11-343H5.4 | turquoise |
| RP11-343J24.1 | turquoise |
| RP11-344B5.2 | brown |
| RP11-344N10.3 | blue |
| RP11-345I18.4 | turquoise |
| RP11-345P4.5 | blue |
| RP11-346C16.1 | red |
| RP11-346C16.2 | red |
| RP11-346I3.4 | midnightblue |
| RP11-347C12.1 | yellow |
| RP11-347C18.3 | turquoise |
| RP11-348H3.6 | turquoise |
| RP11-34E5.2 | turquoise |
| RP11-34E5.4 | turquoise |
| RP11-34F20.4 | turquoise |
| RP11-34F20.5 | turquoise |
| RP11-34P13.12 | yellow |
| RP11-34P13.13 | blue |
| RP11-351K16.4 | turquoise |
| RP11-351K16.5 | turquoise |
| RP11-351N5.1 | turquoise |
| RP11-351O1.2 | turquoise |
| RP11-353N4.1 | turquoise |
| RP11-353N4.3 | turquoise |
| RP11-354E23.2 | blue |
| RP11-354M20.3 | turquoise |
| RP11-355O1.7 | salmon |
| RP11-356I2.4 | turquoise |
| RP11-357C3.3 | yellow |
| RP11-357H14.12 | blue |
| RP11-357L2.1 | turquoise |
| RP11-358L16.2 | turquoise |
| RP11-359B20.1 | yellow |
| RP11-359C24.1 | turquoise |
| RP11-35G22.1 | darkred |
| RP11-360L9.7 | green |
| RP11-360O19.5 | magenta |
| RP11-361F15.2 | blue |
| RP11-361K17.2 | red |
| RP11-362A9.3 | turquoise |
| RP11-363P13.1 | blue |
| RP11-364F11.1 | blue |
| RP11-364L4.1 | turquoise |
| RP11-364P22.1 | brown |
| RP11-364P22.2 | yellow |
| RP11-365D23.4 | yellow |
| RP11-365H22.1 | brown |
| RP11-366O20.4 | turquoise |
| RP11-367H5.5 | blue |
| RP11-367H5.7 | greenyellow |
| RP11-367J11.2 | black |
| RP11-369J21.6 | brown |
| RP11-36N20.1 | blue |
| RP11-36N20.2 | blue |
| RP11-370K11.2 | turquoise |
| RP11-373A9.1 | blue |
| RP11-374F3.4 | turquoise |
| RP11-378G13.2 | blue |
| RP11-378J18.6 | turquoise |
| RP11-379B18.5 | blue |
| RP11-379F4.4 | brown |
| RP11-379J5.2 | yellow |
| RP11-379K17.7 | salmon |
| RP11-37B2.1 | midnightblue |
| RP11-380G5.3 | black |
| RP11-380P13.2 | darkgrey |
| RP11-381E24.1 | red |
| RP11-381K20.4 | red |
| RP11-381O7.3 | blue |
| RP11-382A18.1 | brown |
| RP11-382D8.4 | yellow |
| RP11-382J12.1 | darkgrey |
| RP11-382J24.2 | turquoise |
| RP11-383F6.1 | turquoise |
| RP11-383H13.1 | blue |
| RP11-383J24.2 | turquoise |
| RP11-383J24.5 | midnightblue |
| RP11-384C4.2 | turquoise |
| RP11-384C4.3 | turquoise |
| RP11-384C4.6 | turquoise |
| RP11-384K6.2 | yellow |
| RP11-385F5.4 | yellow |
| RP11-388P9.2 | red |
| RP11-38L15.3 | yellow |
| RP11-390B4.3 | turquoise |
| RP11-390F4.3 | turquoise |
| RP11-390F4.8 | turquoise |
| RP11-390G14.1 | yellow |
| RP11-390P2.2 | brown |
| RP11-390P2.3 | yellow |
| RP11-390P2.4 | turquoise |
| RP11-391H12.5 | turquoise |
| RP11-391M1.3 | blue |
| RP11-392A22.1 | blue |
| RP11-392B6.1 | blue |
| RP11-392O18.1 | brown |
| RP11-393B14.1 | turquoise |
| RP11-393I2.2 | red |
| RP11-394O4.2 | black |
| RP11-395I14.2 | turquoise |
| RP11-395P17.3 | yellow |
| RP11-397D12.6 | purple |
| RP11-397D12.7 | yellow |
| RP11-397F23.2 | turquoise |
| RP11-397P13.6 | pink |
| RP11-397P13.7 | blue |
| RP11-398C13.4 | brown |
| RP11-398F12.1 | yellow |
| RP11-398K22.12 | blue |
| RP11-399O19.5 | black |
| RP11-39E3.4 | turquoise |
| RP11-39E3.5 | yellow |
| RP11-39K24.5 | turquoise |
| RP11-39K24.9 | turquoise |
| RP11-39N8.2 | blue |
| RP11-3A3.1 | darkgrey |
| RP11-3B7.6 | turquoise |
| RP11-3J10.4 | brown |
| RP11-3J10.7 | blue |
| RP11-3N2.13 | brown |
| RP11-3P17.3 | salmon |
| RP11-400K9.4 | midnightblue |
| RP11-401A10.2 | turquoise |
| RP11-401L13.2 | yellow |
| RP11-402L5.1 | turquoise |
| RP11-403I13.4 | blue |
| RP11-403I13.7 | turquoise |
| RP11-403I13.8 | turquoise |
| RP11-404E16.1 | blue |
| RP11-405L18.1 | blue |
| RP11-405L18.4 | blue |
| RP11-408A13.1 | turquoise |
| RP11-409K20.8 | turquoise |
| RP11-40F6.2 | turquoise |
| RP11-410L14.1 | turquoise |
| RP11-410L14.2 | blue |
| RP11-411G7.1 | yellow |
| RP11-411K7.1 | turquoise |
| RP11-413E1.2 | turquoise |
| RP11-413E6.8 | brown |
| RP11-413G22.2 | yellow |
| RP11-413M3.4 | turquoise |
| RP11-414H17.5 | blue |
| RP11-414K1.3 | brown |
| RP11-414P19.1 | turquoise |
| RP11-415J8.3 | blue |
| RP11-417B4.2 | turquoise |
| RP11-417E7.1 | lightcyan |
| RP11-417F21.1 | blue |
| RP11-417O11.5 | blue |
| RP11-417O11.6 | blue |
| RP11-418J17.1 | turquoise |
| RP11-420A21.1 | blue |
| RP11-420A23.1 | turquoise |
| RP11-421E17.3 | brown |
| RP11-421L21.3 | red |
| RP11-422J8.1 | salmon |
| RP11-423E7.1 | brown |
| RP11-423H2.1 | blue |
| RP11-423O2.1 | yellow |
| RP11-426A6.7 | yellow |
| RP11-428C6.1 | brown |
| RP11-429A24.4 | brown |
| RP11-429G19.2 | turquoise |
| RP11-430C17.1 | blue |
| RP11-430C7.2 | turquoise |
| RP11-430K21.2 | blue |
| RP11-432A8.2 | brown |
| RP11-432J9.5 | brown |
| RP11-433J22.2 | blue |
| RP11-435O5.2 | brown |
| RP11-436H11.2 | blue |
| RP11-436H11.3 | blue |
| RP11-436H11.5 | blue |
| RP11-438E5.1 | darkgreen |
| RP11-439A17.2 | brown |
| RP11-439E19.1 | turquoise |
| RP11-43D2.4 | blue |
| RP11-43F13.1 | blue |
| RP11-440L14.1 | turquoise |
| RP11-443B7.1 | turquoise |
| RP11-444I9.2 | blue |
| RP11-444I9.3 | blue |
| RP11-444M10.3 | blue |
| RP11-445O16.3 | darkgrey |
| RP11-445O3.1 | darkgrey |
| RP11-446H18.3 | blue |
| RP11-448G4.1 | turquoise |
| RP11-448G4.2 | turquoise |
| RP11-449H3.2 | salmon |
| RP11-449K6.4 | green |
| RP11-449M18.2 | turquoise |
| RP11-44I10.1 | turquoise |
| RP11-44N11.1 | turquoise |
| RP11-451H23.1 | yellow |
| RP11-452F19.3 | blue |
| RP11-452K12.7 | blue |
| RP11-453E17.1 | red |
| RP11-453E17.2 | turquoise |
| RP11-453F18__B.1 | blue |
| RP11-454L1.2 | magenta |
| RP11-456N14.2 | turquoise |
| RP11-458B24.2 | darkgrey |
| RP11-460I13.6 | turquoise |
| RP11-462D18.2 | brown |
| RP11-463H24.4 | turquoise |
| RP11-464F9.1 | turquoise |
| RP11-465B22.3 | brown |
| RP11-465N4.4 | lightgreen |
| RP11-466F5.3 | yellow |
| RP11-466F5.4 | pink |
| RP11-466H18.1 | salmon |
| RP11-467H10.1 | turquoise |
| RP11-46A10.4 | yellow |
| RP11-46H11.1 | yellow |
| RP11-470H9.1 | turquoise |
| RP11-471M10.1 | black |
| RP11-471M10.2 | black |
| RP11-472B18.2 | blue |
| RP11-472I20.1 | red |
| RP11-473O4.1 | brown |
| RP11-473O4.4 | greenyellow |
| RP11-474D14.2 | blue |
| RP11-474D14.3 | turquoise |
| RP11-474L11.5 | turquoise |
| RP11-475C16.1 | royalblue |
| RP11-475O6.1 | brown |
| RP11-477E3.2 | turquoise |
| RP11-477J21.5 | turquoise |
| RP11-477J21.6 | lightyellow |
| RP11-479G22.3 | blue |
| RP11-479G22.5 | blue |
| RP11-47G11.2 | turquoise |
| RP11-480D4.3 | turquoise |
| RP11-480I12.1 | magenta |
| RP11-480I12.2 | brown |
| RP11-480I12.3 | magenta |
| RP11-480I12.5 | brown |
| RP11-481K16.2 | turquoise |
| RP11-483F11.5 | blue |
| RP11-484D18.2 | blue |
| RP11-484O2.1 | darkgrey |
| RP11-486B10.4 | yellow |
| RP11-486G15.1 | brown |
| RP11-487E1.1 | blue |
| RP11-488L18.4 | turquoise |
| RP11-488L18.8 | royalblue |
| RP11-48O20.4 | turquoise |
| RP11-490F3.1 | turquoise |
| RP11-491H9.3 | blue |
| RP11-492E3.1 | blue |
| RP11-492I21.1 | blue |
| RP11-493K19.3 | turquoise |
| RP11-496H1.1 | brown |
| RP11-499P20.2 | pink |
| RP11-49O14.3 | blue |
| RP11-4K16.2 | brown |
| RP11-4K3__A.2 | yellow |
| RP11-4K3__A.5 | turquoise |
| RP11-501E14.1 | darkgrey |
| RP11-503E24.2 | yellow |
| RP11-503N18.5 | turquoise |
| RP11-504P24.2 | brown |
| RP11-504P24.4 | yellow |
| RP11-506B15.4 | yellow |
| RP11-506B4.3 | turquoise |
| RP11-506B4.4 | turquoise |
| RP11-506B4.5 | turquoise |
| RP11-506M12.1 | yellow |
| RP11-506M13.3 | blue |
| RP11-506N2.1 | darkgrey |
| RP11-508N12.3 | brown |
| RP11-508N12.4 | magenta |
| RP11-509J21.1 | turquoise |
| RP11-510N19.5 | turquoise |
| RP11-512F24.1 | magenta |
| RP11-513G11.1 | salmon |
| RP11-513I15.6 | magenta |
| RP11-513M16.2 | blue |
| RP11-517I3.1 | blue |
| RP11-518D3.3 | blue |
| RP11-518L10.3 | turquoise |
| RP11-521B24.2 | yellow |
| RP11-523H24.3 | turquoise |
| RP11-524O24.2 | yellow |
| RP11-527B17.2 | blue |
| RP11-528L24.2 | blue |
| RP11-52I18.1 | greenyellow |
| RP11-52J3.2 | lightyellow |
| RP11-533E19.3 | turquoise |
| RP11-534G20.3 | yellow |
| RP11-535M15.1 | brown |
| RP11-535M15.2 | yellow |
| RP11-536K7.3 | yellow |
| RP11-536O18.1 | blue |
| RP11-537E18.1 | turquoise |
| RP11-537H15.3 | green |
| RP11-537I16.2 | yellow |
| RP11-539G18.1 | blue |
| RP11-539I5.1 | brown |
| RP11-539L10.3 | turquoise |
| RP11-53O19.1 | blue |
| RP11-540D14.6 | yellow |
| RP11-544M22.8 | brown |
| RP11-545E17.3 | yellow |
| RP11-545G3.1 | brown |
| RP11-546N22.5 | purple |
| RP11-546N22.6 | black |
| RP11-549K20.1 | royalblue |
| RP11-54A4.2 | blue |
| RP11-54K16.2 | brown |
| RP11-550E22.4 | turquoise |
| RP11-550I24.2 | blue |
| RP11-551L14.1 | cyan |
| RP11-552M11.2 | turquoise |
| RP11-553K8.3 | turquoise |
| RP11-553K8.5 | turquoise |
| RP11-556O5.4 | blue |
| RP11-557C18.3 | turquoise |
| RP11-560J1.1 | brown |
| RP11-561N12.4 | pink |
| RP11-561O23.8 | brown |
| RP11-562A8.1 | turquoise |
| RP11-563E2.2 | turquoise |
| RP11-564P3.1 | darkgrey |
| RP11-567M21.1 | blue |
| RP11-568K15.1 | blue |
| RP11-570D4.2 | turquoise |
| RP11-571F15.2 | blue |
| RP11-571F15.3 | blue |
| RP11-574F21.2 | royalblue |
| RP11-574H13.2 | yellow |
| RP11-574K11.16 | lightyellow |
| RP11-574K11.18 | brown |
| RP11-574K11.20 | yellow |
| RP11-574K11.5 | blue |
| RP11-575A19.2 | blue |
| RP11-575L7.8 | turquoise |
| RP11-576I22.2 | purple |
| RP11-578O24.2 | turquoise |
| RP11-57H12.5 | yellow |
| RP11-589F5.3 | blue |
| RP11-58A11.2 | turquoise |
| RP11-58B17.1 | brown |
| RP11-58H15.1 | turquoise |
| RP11-58H15.4 | yellow |
| RP11-592N21.1 | blue |
| RP11-597D13.9 | blue |
| RP11-598P20.3 | brown |
| RP11-5K16.2 | blue |
| RP11-5N23.2 | turquoise |
| RP11-600F24.2 | blue |
| RP11-611D20.2 | brown |
| RP11-612J15.2 | blue |
| RP11-613C6.4 | turquoise |
| RP11-613M10.6 | yellow |
| RP11-617D20.1 | blue |
| RP11-61L23.2 | turquoise |
| RP11-622K12.1 | brown |
| RP11-622P13.2 | turquoise |
| RP11-626E13.1 | brown |
| RP11-627C21.1 | blue |
| RP11-62I21.1 | lightcyan |
| RP11-62J1.3 | yellow |
| RP11-631M6.1 | black |
| RP11-631M6.3 | turquoise |
| RP11-632C17__A.1 | brown |
| RP11-632F7.3 | blue |
| RP11-632K20.1 | black |
| RP11-63P12.6 | brown |
| RP11-642D21.1 | blue |
| RP11-642D21.2 | blue |
| RP11-645C24.2 | turquoise |
| RP11-64I24.2 | turquoise |
| RP11-656D10.3 | yellow |
| RP11-656D10.5 | blue |
| RP11-656D10.6 | green |
| RP11-65F13.1 | turquoise |
| RP11-65F13.2 | black |
| RP11-65J3.1 | blue |
| RP11-65N13.4 | turquoise |
| RP11-661D19.1 | blue |
| RP11-662B19.1 | blue |
| RP11-662B19.2 | yellow |
| RP11-665C16.1 | turquoise |
| RP11-666A1.1 | greenyellow |
| RP11-666A8.1 | grey60 |
| RP11-67L3.5 | tan |
| RP11-681L8.1 | darkgrey |
| RP11-68I3.1 | turquoise |
| RP11-692C10.1 | blue |
| RP11-696N14.1 | blue |
| RP11-697E2.1 | yellow |
| RP11-697G4.3 | blue |
| RP11-697K23.1 | turquoise |
| RP11-697M17.1 | turquoise |
| RP11-697M17.2 | darkgrey |
| RP11-697N18.1 | yellow |
| RP11-697N18.3 | yellow |
| RP11-69E11.4 | turquoise |
| RP11-69I8.3 | blue |
| RP11-6D1.5 | midnightblue |
| RP11-6F2.7 | brown |
| RP11-701P16.1 | brown |
| RP11-702A23.1 | greenyellow |
| RP11-703G6.1 | yellow |
| RP11-704P17.1 | turquoise |
| RP11-706O15.1 | magenta |
| RP11-70J12.1 | lightcyan |
| RP11-70L8.1 | brown |
| RP11-714G12.1 | brown |
| RP11-723D22.3 | lightyellow |
| RP11-723O4.6 | brown |
| RP11-726G1.1 | cyan |
| RP11-727A23.1 | yellow |
| RP11-72M14.1 | turquoise |
| RP11-730A19.6 | lightgreen |
| RP11-731F5.2 | turquoise |
| RP11-731I19.1 | blue |
| RP11-732M18.3 | pink |
| RP11-738E22.1 | black |
| RP11-738E22.2 | black |
| RP11-745A24.1 | turquoise |
| RP11-745J15.1 | brown |
| RP11-746L20.1 | greenyellow |
| RP11-74C1.2 | brown |
| RP11-74C1.4 | brown |
| RP11-752L20.4 | purple |
| RP11-759F5.1 | yellow |
| RP11-75G10.2 | yellow |
| RP11-760H22.2 | blue |
| RP11-761E20.1 | brown |
| RP11-764K9.2 | darkgreen |
| RP11-767L7.2 | turquoise |
| RP11-767N6.2 | blue |
| RP11-767N6.7 | brown |
| RP11-773D16.1 | yellow |
| RP11-774O3.3 | blue |
| RP11-775J23.1 | blue |
| RP11-775J23.2 | yellow |
| RP11-77O7.1 | turquoise |
| RP11-77P16.4 | blue |
| RP11-786O7.1 | brown |
| RP11-791I24.4 | salmon |
| RP11-792D21.2 | brown |
| RP11-797H7.1 | yellow |
| RP11-797H7.5 | brown |
| RP11-798M19.3 | blue |
| RP11-79D8.2 | turquoise |
| RP11-79I23.2 | blue |
| RP11-79L9.2 | turquoise |
| RP11-79N23.1 | brown |
| RP11-806O11.1 | brown |
| RP11-809C18.3 | yellow |
| RP11-809O17.1 | brown |
| RP11-812I20.2 | yellow |
| RP11-814E24.3 | blue |
| RP11-814P23.1 | yellow |
| RP11-81B10.2 | black |
| RP11-81F11.3 | blue |
| RP11-81N13.1 | blue |
| RP11-823P9.1 | brown |
| RP11-823P9.3 | yellow |
| RP11-82L18.2 | turquoise |
| RP11-834C11.4 | blue |
| RP11-83A16.1 | blue |
| RP11-83A24.1 | red |
| RP11-83J16.1 | turquoise |
| RP11-83M16.5 | darkgrey |
| RP11-841C19.1 | cyan |
| RP11-848G14.5 | blue |
| RP11-85F14.5 | turquoise |
| RP11-861M13.1 | blue |
| RP11-86H7.1 | darkgreen |
| RP11-875O11.1 | turquoise |
| RP11-87H9.3 | blue |
| RP11-884K10.5 | greenyellow |
| RP11-885N19.5 | turquoise |
| RP11-889L3.3 | turquoise |
| RP11-88B8.2 | blue |
| RP11-88I18.2 | turquoise |
| RP11-89C21.2 | brown |
| RP11-89C21.3 | yellow |
| RP11-89K10.1 | blue |
| RP11-8L18.2 | turquoise |
| RP11-8L18.3 | brown |
| RP11-904M10.1 | cyan |
| RP11-90B22.2 | black |
| RP11-90L20.3 | yellow |
| RP11-90O23.1 | turquoise |
| RP11-91A18.4 | greenyellow |
| RP11-93B14.5 | brown |
| RP11-93K22.11 | yellow |
| RP11-946L20.4 | darkgrey |
| RP11-96C23.7 | greenyellow |
| RP11-96C23.8 | yellow |
| RP11-96C23.9 | blue |
| RP11-96L14.7 | blue |
| RP11-974F13.6 | blue |
| RP11-981G7.2 | blue |
| RP11-981G7.4 | purple |
| RP11-98D18.9 | lightgreen |
| RP11-98I9.4 | turquoise |
| RP11-9E13.2 | red |
| RP13-1056D16.2 | blue |
| RP13-128O4.3 | blue |
| RP13-131K19.1 | turquoise |
| RP13-131K19.2 | turquoise |
| RP13-15M17.1 | tan |
| RP13-188A5.1 | blue |
| RP13-204A15.3 | blue |
| RP13-216E22.4 | turquoise |
| RP13-33H18.1 | blue |
| RP13-36G14.3 | blue |
| RP13-467E5.1 | red |
| RP13-507I23.1 | pink |
| RP13-582O9.5 | darkred |
| RP13-685P2.5 | yellow |
| RP13-766D20.2 | grey60 |
| RP2 | brown |
| RP3-323N1.2 | turquoise |
| RP3-324O17.4 | red |
| RP3-324O17.5 | yellow |
| RP3-329E20.2 | lightyellow |
| RP3-337H4.6 | magenta |
| RP3-354N19.3 | turquoise |
| RP3-355C18.7 | darkgrey |
| RP3-355L5.2 | yellow |
| RP3-355L5.3 | red |
| RP3-368A4.3 | blue |
| RP3-370M22.6 | green |
| RP3-377D14.1 | yellow |
| RP3-393P12.3 | turquoise |
| RP3-395M20.8 | turquoise |
| RP3-398G3.3 | blue |
| RP3-399L15.3 | yellow |
| RP3-402G11.5 | yellow |
| RP3-403L10.3 | turquoise |
| RP3-406A7.5 | brown |
| RP3-410C9.1 | greenyellow |
| RP3-412A9.10 | blue |
| RP3-412A9.11 | brown |
| RP3-415N12.1 | blue |
| RP3-430N8.8 | yellow |
| RP3-449O17.1 | yellow |
| RP3-468O1.2 | turquoise |
| RP3-470L14.1 | blue |
| RP3-476K8.4 | turquoise |
| RP3-477O4.14 | turquoise |
| RP3-481F12.4 | yellow |
| RP3-483K16.2 | blue |
| RP3-486I3.4 | brown |
| RP3-486I3.5 | yellow |
| RP3-486L4.3 | red |
| RP3-486L4.4 | turquoise |
| RP3-487J7.2 | turquoise |
| RP3-508I15.16 | turquoise |
| RP3-508I15.19 | turquoise |
| RP3-508I15.9 | yellow |
| RP3-510D11.1 | lightcyan |
| RP3-511B24.4 | blue |
| RP3-521E19.1 | blue |
| RP3-522J7.5 | blue |
| RP3-525L6.2 | turquoise |
| RP4-533D7.3 | turquoise |
| RP4-533D7.4 | brown |
| RP4-537K17.2 | brown |
| RP4-543J19.8 | turquoise |
| RP4-545C24.1 | blue |
| RP4-549L20.2 | yellow |
| RP4-555L14.5 | turquoise |
| RP4-564F22.2 | green |
| RP4-565E6.1 | blue |
| RP4-569M23.2 | tan |
| RP4-583P15.10 | yellow |
| RP4-591B8.2 | yellow |
| RP4-591N18.2 | blue |
| RP4-594I10.2 | brown |
| RP4-597J3.1 | blue |
| RP4-604K5.1 | red |
| RP4-614O4.5 | blue |
| RP4-617A9.4 | yellow |
| RP4-622L5.7 | yellow |
| RP4-633I8.2 | turquoise |
| RP4-633I8.3 | blue |
| RP4-633I8.4 | yellow |
| RP4-635E18.6 | blue |
| RP4-635E8.1 | turquoise |
| RP4-639F20.1 | lightcyan |
| RP4-655C5.1 | turquoise |
| RP4-655J12.3 | brown |
| RP4-655L22.2 | blue |
| RP4-657D16.3 | turquoise |
| RP4-669L17.10 | yellow |
| RP4-669P10.18 | turquoise |
| RP4-669P10.19 | yellow |
| RP4-671G15.2 | blue |
| RP4-671G15.3 | blue |
| RP4-682C21.2 | turquoise |
| RP4-694B14.4 | brown |
| RP4-694B14.5 | brown |
| RP4-695O20__B.10 | yellow |
| RP4-697K14.7 | turquoise |
| RP4-717I23.3 | turquoise |
| RP4-725G10.1 | brown |
| RP4-728D4.2 | turquoise |
| RP4-730K3.3 | blue |
| RP4-742C19.8 | turquoise |
| RP4-742J24.2 | brown |
| RP4-756H11.3 | yellow |
| RP4-758J18.10 | turquoise |
| RP4-758J18.2 | turquoise |
| RP4-758J18.5 | turquoise |
| RP4-758J18.6 | blue |
| RP4-761I2.2 | green |
| RP4-765A10.1 | pink |
| RP4-765A10.2 | yellow |
| RP4-765C7.2 | turquoise |
| RP4-773A18.2 | turquoise |
| RP4-778K6.2 | green |
| RP4-778K6.3 | blue |
| RP4-781K5.2 | greenyellow |
| RP4-798C17.5 | brown |
| RP4-798C17.6 | blue |
| RP4-800G7.2 | yellow |
| RP4-803J11.2 | turquoise |
| RP4-811H24.6 | yellow |
| RP4-814D15.1 | yellow |
| RP4-814D15.2 | greenyellow |
| RP5-1007M22.2 | turquoise |
| RP5-1022P6.3 | turquoise |
| RP5-1022P6.6 | blue |
| RP5-1024G6.2 | blue |
| RP5-1033H22.2 | blue |
| RP5-1043F6.2 | turquoise |
| RP5-1049G16.4 | turquoise |
| RP5-1056L3.3 | greenyellow |
| RP5-1061H20.4 | blue |
| RP5-1086K13.3 | turquoise |
| RP5-1087E8.3 | yellow |
| RP5-1091E12.1 | brown |
| RP5-1092L12.2 | darkgreen |
| RP5-1103G7.4 | yellow |
| RP5-1112D6.3 | blue |
| RP5-1112D6.4 | turquoise |
| RP5-1113E3.3 | salmon |
| RP5-1116C7.1 | blue |
| RP5-1120P11.1 | darkgreen |
| RP5-1125A11.1 | blue |
| RP5-1141O19.2 | turquoise |
| RP5-1142A6.2 | yellow |
| RP5-1158E12.3 | yellow |
| RP5-1160K1.3 | blue |
| RP5-1160K1.6 | yellow |
| RP5-1166A24.1 | blue |
| RP5-1166H10.2 | turquoise |
| RP5-1168M19.1 | brown |
| RP5-1174J21.2 | turquoise |
| RP5-1177E19.2 | turquoise |
| RP5-1180C10.2 | yellow |
| RP5-1182A14.3 | turquoise |
| RP5-1187M17.10 | magenta |
| RP5-1198E17.1 | pink |
| RP5-821D11.7 | yellow |
| RP5-827C21.4 | brown |
| RP5-835G14.1 | turquoise |
| RP5-836N10.1 | turquoise |
| RP5-837D10.2 | turquoise |
| RP5-837J1.1 | turquoise |
| RP5-848E13.3 | brown |
| RP5-857K21.10 | greenyellow |
| RP5-857K21.6 | red |
| RP5-857K21.8 | greenyellow |
| RP5-862P8.2 | green |
| RP5-867C24.1 | turquoise |
| RP5-874C20.3 | blue |
| RP5-890O3.9 | yellow |
| RP5-891H21.4 | yellow |
| RP5-892G19.1 | turquoise |
| RP5-894A10.2 | brown |
| RP5-894A10.5 | brown |
| RP5-901O8.3 | turquoise |
| RP5-902P8.10 | blue |
| RP5-907D15.2 | brown |
| RP5-916O11.1 | red |
| RP5-930J4.4 | blue |
| RP5-931E15.2 | darkgrey |
| RP5-956O18.3 | brown |
| RP5-977B1.7 | yellow |
| RP5-979D14.1 | blue |
| RP5-981O7.2 | blue |
| RP5-981O7.3 | turquoise |
| RP5-994D16.9 | yellow |
| RP5-995J12.2 | brown |
| RP5-997D16.2 | yellow |
| RP5-998N21.5 | brown |
| RP6-109B7.2 | yellow |
| RP6-109B7.3 | blue |
| RP6-109B7.4 | yellow |
| RP9 | magenta |
| RP9P | turquoise |
| RPA1 | green |
| RPA3 | yellow |
| RPAIN | magenta |
| RPAP2 | pink |
| RPF2 | red |
| RPGR | blue |
| RPIA | green |
| RPL10 | salmon |
| RPL10A | salmon |
| RPL10P3 | greenyellow |
| RPL11 | salmon |
| RPL12 | salmon |
| RPL12P11 | blue |
| RPL13 | salmon |
| RPL13A | salmon |
| RPL13AP5 | salmon |
| RPL13AP6 | turquoise |
| RPL13P5 | cyan |
| RPL14 | salmon |
| RPL15 | salmon |
| RPL15P3 | salmon |
| RPL18 | salmon |
| RPL18A | salmon |
| RPL18AP3 | salmon |
| RPL19 | salmon |
| RPL19P14 | turquoise |
| RPL21 | salmon |
| RPL21P44 | turquoise |
| RPL22 | salmon |
| RPL22L1 | salmon |
| RPL23 | salmon |
| RPL23A | salmon |
| RPL23AP1 | turquoise |
| RPL23AP32 | blue |
| RPL23AP53 | blue |
| RPL23AP64 | turquoise |
| RPL23AP7 | turquoise |
| RPL24 | salmon |
| RPL26 | salmon |
| RPL26L1 | turquoise |
| RPL27 | salmon |
| RPL27A | salmon |
| RPL28 | turquoise |
| RPL29 | salmon |
| RPL3 | salmon |
| RPL30 | salmon |
| RPL31 | salmon |
| RPL32 | salmon |
| RPL32P3 | yellow |
| RPL34 | salmon |
| RPL35 | salmon |
| RPL35A | salmon |
| RPL36 | salmon |
| RPL36A | salmon |
| RPL36AL | brown |
| RPL37 | salmon |
| RPL37A | salmon |
| RPL39 | salmon |
| RPL39L | blue |
| RPL4 | salmon |
| RPL41 | royalblue |
| RPL5 | salmon |
| RPL7 | midnightblue |
| RPL7A | salmon |
| RPL7AP6 | salmon |
| RPL7L1 | magenta |
| RPL8 | darkred |
| RPL9 | salmon |
| RPL9P7 | salmon |
| RPLP0 | salmon |
| RPLP0P2 | lightcyan |
| RPLP1 | salmon |
| RPLP2 | salmon |
| RPN1 | blue |
| RPN2 | blue |
| RPP21 | magenta |
| RPP25 | blue |
| RPP38 | lightgreen |
| RPP40 | blue |
| RPRD1A | red |
| RPRD1B | yellow |
| RPRD2 | yellow |
| RPS10 | salmon |
| RPS10L | yellow |
| RPS10P7 | yellow |
| RPS12 | salmon |
| RPS13 | salmon |
| RPS14 | salmon |
| RPS15 | salmon |
| RPS15A | salmon |
| RPS15AP10 | turquoise |
| RPS15AP16 | brown |
| RPS16 | salmon |
| RPS17 | darkgrey |
| RPS18 | salmon |
| RPS19 | salmon |
| RPS19BP1 | blue |
| RPS2 | salmon |
| RPS20 | salmon |
| RPS21 | salmon |
| RPS23 | salmon |
| RPS24 | salmon |
| RPS25 | salmon |
| RPS26 | yellow |
| RPS27 | salmon |
| RPS27A | salmon |
| RPS27L | brown |
| RPS28 | salmon |
| RPS29 | salmon |
| RPS2P32 | turquoise |
| RPS3 | salmon |
| RPS3A | salmon |
| RPS3AP6 | salmon |
| RPS3P2 | blue |
| RPS4X | salmon |
| RPS5 | salmon |
| RPS5P2 | greenyellow |
| RPS6 | brown |
| RPS6KA1 | brown |
| RPS6KA2 | blue |
| RPS6KA3 | turquoise |
| RPS6KA4 | brown |
| RPS6KA5 | turquoise |
| RPS6KA6 | brown |
| RPS6KB1 | grey60 |
| RPS6KB2 | blue |
| RPS6KL1 | brown |
| RPS7 | salmon |
| RPS8 | salmon |
| RPS9P1 | tan |
| RPSA | salmon |
| RPSAP58 | brown |
| RPTOR | yellow |
| RPUSD1 | blue |
| RPUSD3 | blue |
| RRAD | blue |
| RRAGA | blue |
| RRAGB | pink |
| RRAGC | yellow |
| RRAGD | turquoise |
| RRAS | blue |
| RRAS2 | red |
| RRBP1 | salmon |
| RREB1 | yellow |
| RRM1 | green |
| RRM2 | green |
| RRM2B | midnightblue |
| RRN3 | red |
| RRN3P2 | turquoise |
| RRP1 | blue |
| RRP12 | blue |
| RRP15 | green |
| RRP1B | green |
| RRP7A | blue |
| RRP9 | green |
| RRS1 | darkred |
| RSAD1 | turquoise |
| RSAD2 | turquoise |
| RSC1A1 | red |
| RSF1 | yellow |
| RSL1D1 | red |
| RSL24D1 | red |
| RSPH3 | pink |
| RSPO3 | turquoise |
| RSRC1 | green |
| RSU1 | greenyellow |
| RTEL1 | yellow |
| RTKN | lightgreen |
| RTKN2 | green |
| RTN1 | turquoise |
| RTN2 | blue |
| RTN3 | turquoise |
| RTN4 | blue |
| RTN4IP1 | red |
| RTN4R | brown |
| RTP4 | turquoise |
| RTTN | red |
| RUFY1 | yellow |
| RUFY2 | blue |
| RUFY3 | blue |
| RUNDC2A | black |
| RUNDC2B | turquoise |
| RUNX1 | lightyellow |
| RUNX1T1 | black |
| RUNX2 | purple |
| RUNX3 | turquoise |
| RUSC1 | lightgreen |
| RUSC2 | blue |
| RUVBL1 | green |
| RUVBL2 | green |
| RWDD2B | red |
| RWDD3 | pink |
| RWDD4A | red |
| RXRA | yellow |
| RXRB | magenta |
| RYBP | red |
| RYK | brown |
| RYR2 | blue |
| S100A1 | brown |
| S100A10 | purple |
| S100A11 | lightgreen |
| S100A13 | yellow |
| S100A14 | blue |
| S100A16 | turquoise |
| S100A2 | turquoise |
| S100A3 | brown |
| S100A4 | brown |
| S100A6 | yellow |
| S100A7 | tan |
| S100A8 | brown |
| S100A9 | brown |
| S100B | blue |
| S100P | turquoise |
| S1PR1 | turquoise |
| S1PR2 | turquoise |
| S1PR3 | blue |
| S1PR4 | turquoise |
| S1PR5 | blue |
| SAA1 | turquoise |
| SAA2 | turquoise |
| SAAL1 | red |
| SAC3D1 | green |
| SACS | turquoise |
| SAE1 | green |
| SALL4 | lightcyan |
| SAMD10 | blue |
| SAMD11 | blue |
| SAMD12 | midnightblue |
| SAMD13 | brown |
| SAMD14 | turquoise |
| SAMD4A | blue |
| SAMD4B | greenyellow |
| SAMD8 | blue |
| SAMD9 | turquoise |
| SAMD9L | turquoise |
| SAMHD1 | turquoise |
| SAMM50 | blue |
| SAMSN1 | turquoise |
| SAP130 | yellow |
| SAP30 | blue |
| SAP30BP | grey60 |
| SAPS2 | yellow |
| SAR1B | tan |
| SARS2 | brown |
| SART1 | greenyellow |
| SASH1 | blue |
| SASH3 | turquoise |
| SASS6 | green |
| SAT1 | brown |
| SAT2 | blue |
| SATB1 | turquoise |
| SAV1 | brown |
| SBDS | turquoise |
| SBDSP | blue |
| SBF1 | yellow |
| SBF2 | blue |
| SBNO2 | brown |
| SBSN | turquoise |
| SC4MOL | tan |
| SC5DL | darkgreen |
| SCAF1 | yellow |
| SCAMP3 | lightgreen |
| SCAMP4 | yellow |
| SCAMP5 | turquoise |
| SCAND1 | blue |
| SCAND2 | turquoise |
| SCAP | yellow |
| SCARA3 | turquoise |
| SCARA5 | black |
| SCARB1 | blue |
| SCARB2 | blue |
| SCARF1 | brown |
| SCARF2 | blue |
| SCARNA1 | royalblue |
| SCARNA13 | salmon |
| SCARNA16 | lightyellow |
| SCARNA17 | royalblue |
| SCARNA21 | royalblue |
| SCARNA22 | royalblue |
| SCARNA4 | royalblue |
| SCARNA6 | greenyellow |
| SCCPDH | brown |
| SCD | tan |
| SCD5 | blue |
| SCFD1 | red |
| SCG2 | black |
| SCG5 | blue |
| SCGB2A2 | blue |
| SCGB3A2 | turquoise |
| SCHIP1 | blue |
| SCIN | purple |
| SCLY | yellow |
| SCMH1 | brown |
| SCML1 | turquoise |
| SCML2 | blue |
| SCN1B | black |
| SCN3A | blue |
| SCN7A | black |
| SCNM1 | lightgreen |
| SCNN1A | blue |
| SCNN1B | turquoise |
| SCNN1D | blue |
| SCO2 | brown |
| SCOC | turquoise |
| SCP2 | darkgreen |
| SCPEP1 | brown |
| SCRG1 | turquoise |
| SCRIB | darkred |
| SCRN1 | purple |
| SCRN2 | turquoise |
| SCRN3 | blue |
| SCUBE2 | blue |
| SCUBE3 | magenta |
| SCYL1 | blue |
| SDC1 | turquoise |
| SDC2 | lightcyan |
| SDC3 | lightyellow |
| SDC4 | brown |
| SDCBP | brown |
| SDCBP2 | turquoise |
| SDCCAG3 | green |
| SDCCAG8 | turquoise |
| SDF2L1 | yellow |
| SDF4 | brown |
| SDHA | green |
| SDHAF1 | blue |
| SDHAP1 | yellow |
| SDHAP2 | yellow |
| SDHAP3 | blue |
| SDHC | lightgreen |
| SDHD | greenyellow |
| SDK1 | turquoise |
| SDK2 | yellow |
| SDPR | black |
| SDR16C5 | tan |
| SDR42E1 | brown |
| SDS | brown |
| SDSL | brown |
| SEC11A | turquoise |
| SEC11C | turquoise |
| SEC13 | turquoise |
| SEC14L1 | turquoise |
| SEC14L2 | darkgreen |
| SEC16A | yellow |
| SEC16B | blue |
| SEC22B | blue |
| SEC22C | turquoise |
| SEC23A | purple |
| SEC23B | blue |
| SEC23IP | greenyellow |
| SEC24A | turquoise |
| SEC24B | yellow |
| SEC24D | brown |
| SEC31B | turquoise |
| SEC61A2 | green |
| SEC61B | yellow |
| SEC61G | yellow |
| SEC62 | blue |
| SEC63 | red |
| SECISBP2 | turquoise |
| SECISBP2L | blue |
| SECTM1 | turquoise |
| SEH1L | blue |
| SEL1L | turquoise |
| SEL1L3 | turquoise |
| SELENBP1 | brown |
| SELL | turquoise |
| SELPLG | turquoise |
| SEMA3A | turquoise |
| SEMA3B | blue |
| SEMA3C | blue |
| SEMA3D | blue |
| SEMA3E | tan |
| SEMA3F | yellow |
| SEMA3G | black |
| SEMA4A | turquoise |
| SEMA4B | turquoise |
| SEMA4C | magenta |
| SEMA4D | turquoise |
| SEMA4F | yellow |
| SEMA4G | yellow |
| SEMA5A | blue |
| SEMA6A | turquoise |
| SEMA6B | black |
| SEMA6C | yellow |
| SEMA6D | blue |
| SEMA7A | turquoise |
| SENP2 | greenyellow |
| SENP5 | yellow |
| SENP7 | turquoise |
| SEPHS1 | brown |
| SEPHS2 | tan |
| SEPN1 | yellow |
| SEPP1 | black |
| SEPSECS | turquoise |
| 1-Sep | turquoise |
| 10-Sep | yellow |
| 11-Sep | blue |
| 13-Sep | blue |
| 3-Sep | blue |
| 4-Sep | blue |
| 5-Sep | magenta |
| 6-Sep | turquoise |
| SEPT7L | purple |
| 8-Sep | lightcyan |
| 9-Sep | yellow |
| SEPW1 | turquoise |
| SEPX1 | tan |
| SERAC1 | red |
| SERBP1 | green |
| SERF2 | yellow |
| SERGEF | red |
| SERHL | tan |
| SERHL2 | tan |
| SERINC1 | blue |
| SERINC2 | turquoise |
| SERINC4 | blue |
| SERINC5 | blue |
| SERP1 | blue |
| SERPINA1 | brown |
| SERPINA3 | turquoise |
| SERPINB1 | brown |
| SERPINB5 | brown |
| SERPINB6 | blue |
| SERPINB8 | brown |
| SERPINB9 | turquoise |
| SERPINE1 | blue |
| SERPINE2 | blue |
| SERPINF1 | blue |
| SERPINF2 | turquoise |
| SERPING1 | turquoise |
| SERPINH1 | lightcyan |
| SERPINI1 | turquoise |
| SERTAD1 | yellow |
| SERTAD2 | yellow |
| SERTAD3 | yellow |
| SERTAD4 | brown |
| SESN1 | black |
| SESN2 | brown |
| SESN3 | blue |
| SESTD1 | turquoise |
| SET | green |
| SETBP1 | black |
| SETD1A | yellow |
| SETD1B | yellow |
| SETD2 | blue |
| SETD4 | pink |
| SETD5 | yellow |
| SETD6 | brown |
| SETD7 | blue |
| SETD8 | greenyellow |
| SETDB1 | lightgreen |
| SETDB2 | turquoise |
| SETMAR | blue |
| SETX | turquoise |
| SEZ6L2 | turquoise |
| SF1 | yellow |
| SF3A1 | yellow |
| SF3A2 | yellow |
| SF3A3 | green |
| SF3B1 | turquoise |
| SF3B3 | green |
| SF3B4 | lightgreen |
| SF3B5 | blue |
| SFI1 | turquoise |
| SFMBT1 | turquoise |
| SFMBT2 | turquoise |
| SFN | turquoise |
| SFRP1 | brown |
| SFRP2 | blue |
| SFRP4 | blue |
| SFRS11 | pink |
| SFRS12 | blue |
| SFRS13B | red |
| SFRS15 | yellow |
| SFRS16 | yellow |
| SFRS17A | turquoise |
| SFRS18 | red |
| SFRS4 | turquoise |
| SFT2D1 | midnightblue |
| SFT2D2 | blue |
| SFXN1 | brown |
| SFXN2 | yellow |
| SFXN3 | blue |
| SFXN4 | blue |
| SFXN5 | blue |
| SGCB | blue |
| SGCD | blue |
| SGCE | blue |
| SGIP1 | purple |
| SGK1 | brown |
| SGK196 | turquoise |
| SGK3 | midnightblue |
| SGMS1 | brown |
| SGMS2 | lightcyan |
| SGOL1 | green |
| SGOL2 | green |
| SGPL1 | brown |
| SGPP1 | turquoise |
| SGPP2 | darkgreen |
| SGSH | blue |
| SGSM2 | yellow |
| SGSM3 | brown |
| SGTA | greenyellow |
| SGTB | brown |
| SH2B1 | yellow |
| SH2B2 | blue |
| SH2B3 | brown |
| SH2D1A | turquoise |
| SH2D2A | turquoise |
| SH2D3A | brown |
| SH2D3C | turquoise |
| SH2D4A | turquoise |
| SH3BGR | brown |
| SH3BGRL | blue |
| SH3BGRL2 | blue |
| SH3BGRL3 | brown |
| SH3BP1 | turquoise |
| SH3BP2 | turquoise |
| SH3BP4 | yellow |
| SH3BP5 | turquoise |
| SH3BP5L | magenta |
| SH3D19 | black |
| SH3D20 | turquoise |
| SH3GL1 | yellow |
| SH3GLB2 | yellow |
| SH3KBP1 | turquoise |
| SH3PXD2A | lightyellow |
| SH3PXD2B | lightyellow |
| SH3RF1 | magenta |
| SH3RF2 | brown |
| SH3RF3 | blue |
| SH3TC1 | turquoise |
| SH3TC2 | turquoise |
| SH3YL1 | brown |
| SHANK2 | magenta |
| SHARPIN | darkred |
| SHB | turquoise |
| SHC1 | lightcyan |
| SHC2 | green |
| SHC4 | brown |
| SHCBP1 | green |
| SHE | black |
| SHF | blue |
| SHFM1 | blue |
| SHFM3P1 | yellow |
| SHISA4 | blue |
| SHISA5 | turquoise |
| SHKBP1 | turquoise |
| SHMT1 | yellow |
| SHMT1P1 | yellow |
| SHMT2 | blue |
| SHOX2 | blue |
| SHPRH | pink |
| SHQ1 | blue |
| SHROOM1 | blue |
| SHROOM3 | brown |
| SHROOM4 | black |
| SIAE | blue |
| SIAH1 | yellow |
| SIAH2 | green |
| SIDT1 | turquoise |
| SIDT2 | black |
| SIGIRR | turquoise |
| SIGLEC1 | brown |
| SIGLEC12 | brown |
| SIGLEC15 | tan |
| SIGLEC5 | brown |
| SIGLEC7 | brown |
| SIGLEC8 | turquoise |
| SIGLEC9 | brown |
| SIGMAR1 | blue |
| SIK1 | turquoise |
| SIK2 | blue |
| SIK3 | turquoise |
| SIKE1 | turquoise |
| SIL1 | brown |
| SIM1 | blue |
| SIM2 | brown |
| SIN3B | yellow |
| SIP1 | blue |
| SIPA1 | turquoise |
| SIPA1L1 | lightyellow |
| SIPA1L2 | turquoise |
| SIPA1L3 | yellow |
| SIRPA | brown |
| SIRPB1 | brown |
| SIRPB2 | brown |
| SIRT2 | yellow |
| SIRT5 | magenta |
| SIRT7 | grey60 |
| SIT1 | turquoise |
| SIVA1 | blue |
| SIX1 | brown |
| SIX2 | turquoise |
| SIX4 | brown |
| SIX5 | yellow |
| SKA1 | green |
| SKA2 | grey60 |
| SKA3 | green |
| SKAP1 | turquoise |
| SKAP2 | turquoise |
| SKI | yellow |
| SKIL | purple |
| SKIV2L | magenta |
| SKP1P1 | blue |
| SKP2 | green |
| SLA | turquoise |
| SLA2 | turquoise |
| SLAIN1 | turquoise |
| SLAIN2 | yellow |
| SLAMF1 | turquoise |
| SLAMF7 | turquoise |
| SLAMF8 | turquoise |
| SLBP | green |
| SLC10A3 | blue |
| SLC10A7 | turquoise |
| SLC11A1 | brown |
| SLC11A2 | red |
| SLC12A2 | brown |
| SLC12A4 | lightcyan |
| SLC12A6 | turquoise |
| SLC12A7 | yellow |
| SLC12A8 | blue |
| SLC12A9 | yellow |
| SLC13A3 | turquoise |
| SLC13A4 | yellow |
| SLC15A1 | brown |
| SLC15A2 | turquoise |
| SLC15A3 | brown |
| SLC16A1 | blue |
| SLC16A10 | turquoise |
| SLC16A13 | yellow |
| SLC16A2 | blue |
| SLC16A3 | brown |
| SLC16A4 | blue |
| SLC16A5 | blue |
| SLC16A7 | black |
| SLC16A8 | magenta |
| SLC17A5 | turquoise |
| SLC17A9 | turquoise |
| SLC18A2 | blue |
| SLC19A1 | blue |
| SLC19A2 | turquoise |
| SLC19A3 | black |
| SLC1A1 | blue |
| SLC1A2 | blue |
| SLC1A3 | brown |
| SLC1A4 | turquoise |
| SLC1A5 | blue |
| SLC20A1 | turquoise |
| SLC20A2 | brown |
| SLC22A1 | blue |
| SLC22A15 | blue |
| SLC22A17 | blue |
| SLC22A18 | blue |
| SLC22A23 | blue |
| SLC22A5 | yellow |
| SLC23A1 | turquoise |
| SLC23A2 | blue |
| SLC23A3 | yellow |
| SLC24A2 | lightcyan |
| SLC24A3 | brown |
| SLC24A6 | brown |
| SLC25A1 | turquoise |
| SLC25A10 | grey60 |
| SLC25A12 | blue |
| SLC25A13 | green |
| SLC25A14 | red |
| SLC25A15 | brown |
| SLC25A16 | red |
| SLC25A17 | turquoise |
| SLC25A19 | grey60 |
| SLC25A20 | brown |
| SLC25A22 | cyan |
| SLC25A23 | brown |
| SLC25A24 | red |
| SLC25A25 | blue |
| SLC25A27 | blue |
| SLC25A28 | turquoise |
| SLC25A29 | magenta |
| SLC25A3 | turquoise |
| SLC25A30 | blue |
| SLC25A32 | midnightblue |
| SLC25A33 | turquoise |
| SLC25A34 | turquoise |
| SLC25A35 | yellow |
| SLC25A36 | brown |
| SLC25A37 | turquoise |
| SLC25A38 | turquoise |
| SLC25A39 | green |
| SLC25A4 | brown |
| SLC25A40 | green |
| SLC25A42 | turquoise |
| SLC25A43 | green |
| SLC25A45 | turquoise |
| SLC25A46 | brown |
| SLC25A5 | blue |
| SLC25A5P1 | yellow |
| SLC25A6 | salmon |
| SLC26A1 | turquoise |
| SLC26A10 | lightyellow |
| SLC26A11 | turquoise |
| SLC26A2 | turquoise |
| SLC26A4 | turquoise |
| SLC26A6 | darkgreen |
| SLC27A1 | blue |
| SLC27A3 | blue |
| SLC27A4 | green |
| SLC27A5 | blue |
| SLC28A3 | brown |
| SLC29A1 | magenta |
| SLC29A2 | magenta |
| SLC29A3 | turquoise |
| SLC2A1 | turquoise |
| SLC2A10 | blue |
| SLC2A11 | brown |
| SLC2A12 | brown |
| SLC2A13 | brown |
| SLC2A14 | turquoise |
| SLC2A3 | brown |
| SLC2A4RG | brown |
| SLC2A5 | brown |
| SLC2A6 | turquoise |
| SLC2A8 | tan |
| SLC2A9 | brown |
| SLC30A1 | brown |
| SLC30A4 | brown |
| SLC30A7 | red |
| SLC30A9 | red |
| SLC31A1 | tan |
| SLC31A2 | brown |
| SLC33A1 | blue |
| SLC34A2 | yellow |
| SLC35A1 | pink |
| SLC35A2 | blue |
| SLC35A3 | red |
| SLC35A4 | yellow |
| SLC35B1 | grey60 |
| SLC35B2 | magenta |
| SLC35B3 | blue |
| SLC35B4 | brown |
| SLC35C1 | brown |
| SLC35C2 | yellow |
| SLC35D2 | turquoise |
| SLC35E1 | turquoise |
| SLC35E2 | turquoise |
| SLC35E2B | turquoise |
| SLC35E3 | midnightblue |
| SLC35E4 | yellow |
| SLC35F2 | green |
| SLC35F3 | brown |
| SLC35F5 | red |
| SLC36A1 | black |
| SLC36A4 | red |
| SLC37A1 | blue |
| SLC37A2 | brown |
| SLC37A3 | brown |
| SLC37A4 | red |
| SLC38A1 | tan |
| SLC38A10 | turquoise |
| SLC38A2 | blue |
| SLC38A5 | brown |
| SLC38A6 | brown |
| SLC39A1 | lightgreen |
| SLC39A10 | turquoise |
| SLC39A11 | tan |
| SLC39A14 | purple |
| SLC39A3 | blue |
| SLC39A6 | red |
| SLC39A7 | magenta |
| SLC39A8 | brown |
| SLC3A2 | turquoise |
| SLC40A1 | black |
| SLC41A1 | yellow |
| SLC41A2 | brown |
| SLC41A3 | brown |
| SLC43A1 | blue |
| SLC43A2 | brown |
| SLC43A3 | green |
| SLC44A1 | red |
| SLC44A2 | turquoise |
| SLC44A3 | turquoise |
| SLC45A3 | brown |
| SLC45A4 | darkred |
| SLC46A1 | black |
| SLC46A3 | black |
| SLC47A1 | brown |
| SLC48A1 | blue |
| SLC4A11 | yellow |
| SLC4A2 | blue |
| SLC4A3 | magenta |
| SLC4A4 | blue |
| SLC4A5 | yellow |
| SLC4A7 | blue |
| SLC5A1 | brown |
| SLC5A3 | turquoise |
| SLC5A6 | blue |
| SLC6A11 | turquoise |
| SLC6A12 | brown |
| SLC6A14 | brown |
| SLC6A17 | blue |
| SLC6A6 | brown |
| SLC6A8 | turquoise |
| SLC6A9 | green |
| SLC7A1 | blue |
| SLC7A11 | green |
| SLC7A2 | blue |
| SLC7A5 | green |
| SLC7A5P2 | blue |
| SLC7A6 | turquoise |
| SLC7A7 | turquoise |
| SLC7A8 | blue |
| SLC8A1 | turquoise |
| SLC9A1 | yellow |
| SLC9A2 | turquoise |
| SLC9A3 | turquoise |
| SLC9A3R1 | brown |
| SLC9A3R2 | tan |
| SLC9A6 | brown |
| SLC9A7 | cyan |
| SLC9A8 | turquoise |
| SLC9A9 | turquoise |
| SLCO1A2 | cyan |
| SLCO2A1 | black |
| SLCO2B1 | brown |
| SLCO3A1 | turquoise |
| SLCO4A1 | brown |
| SLCO5A1 | turquoise |
| SLFN11 | turquoise |
| SLFN12 | turquoise |
| SLFN12L | turquoise |
| SLFN13 | turquoise |
| SLFN5 | turquoise |
| SLFNL1 | yellow |
| SLIT2 | black |
| SLIT3 | blue |
| SLITRK6 | brown |
| SLMO1 | green |
| SLMO2 | blue |
| SLPI | turquoise |
| SMAD1 | blue |
| SMAD2 | red |
| SMAD3 | black |
| SMAD4 | red |
| SMAD6 | turquoise |
| SMAD7 | purple |
| SMAGP | brown |
| SMAP1 | blue |
| SMAP2 | turquoise |
| SMARCA1 | blue |
| SMARCA2 | turquoise |
| SMARCA4 | greenyellow |
| SMARCB1 | green |
| SMARCC1 | greenyellow |
| SMARCC2 | yellow |
| SMARCD1 | yellow |
| SMARCD2 | grey60 |
| SMARCD3 | blue |
| SMARCE1 | turquoise |
| SMC1A | magenta |
| SMC2 | green |
| SMC4 | green |
| SMC5 | pink |
| SMC6 | red |
| SMCHD1 | darkturquoise |
| SMCR7 | brown |
| SMCR8 | turquoise |
| SMG5 | lightgreen |
| SMG6 | yellow |
| SMG7 | yellow |
| SMN1 | blue |
| SMN2 | turquoise |
| SMO | brown |
| SMOC1 | brown |
| SMOC2 | blue |
| SMOX | turquoise |
| SMPD1 | blue |
| SMPD2 | red |
| SMPD4 | yellow |
| SMPDL3A | brown |
| SMPDL3B | blue |
| SMS | green |
| SMTN | yellow |
| SMURF1 | yellow |
| SMURF2 | grey60 |
| SMYD2 | green |
| SMYD3 | blue |
| SMYD5 | red |
| SNAI1 | purple |
| SNAI2 | black |
| SNAP29 | yellow |
| SNAP47 | green |
| SNAPC2 | turquoise |
| SNAPC3 | brown |
| SNAPC4 | yellow |
| SNCA | black |
| SNCAIP | blue |
| SNCG | black |
| SNED1 | black |
| SNF8 | grey60 |
| SNHG10 | blue |
| SNHG11 | yellow |
| SNHG12 | yellow |
| SNHG3 | salmon |
| SNHG5 | salmon |
| SNHG6 | salmon |
| SNHG7 | royalblue |
| SNHG8 | salmon |
| SNN | turquoise |
| SNORA11 | royalblue |
| SNORA12 | royalblue |
| SNORA14 | royalblue |
| SNORA18 | royalblue |
| SNORA2 | royalblue |
| SNORA20 | royalblue |
| SNORA21 | royalblue |
| SNORA22 | royalblue |
| SNORA23 | greenyellow |
| SNORA24 | royalblue |
| SNORA26 | royalblue |
| SNORA3 | royalblue |
| SNORA30 | royalblue |
| SNORA31 | royalblue |
| SNORA33 | royalblue |
| SNORA38 | royalblue |
| SNORA40 | royalblue |
| SNORA42 | royalblue |
| SNORA47 | royalblue |
| SNORA48 | royalblue |
| SNORA49 | royalblue |
| SNORA5 | royalblue |
| SNORA50 | royalblue |
| SNORA52 | royalblue |
| SNORA53 | greenyellow |
| SNORA54 | royalblue |
| SNORA57 | royalblue |
| SNORA63 | greenyellow |
| SNORA64 | royalblue |
| SNORA65 | royalblue |
| SNORA67 | royalblue |
| SNORA68 | royalblue |
| SNORA7 | royalblue |
| SNORA70 | red |
| SNORA71 | royalblue |
| SNORA73 | greenyellow |
| SNORA74 | greenyellow |
| SNORA75 | darkgrey |
| SNORA79 | royalblue |
| SNORA8 | royalblue |
| SNORA81 | royalblue |
| SNORA84 | royalblue |
| SNORD116 | royalblue |
| SNORD14 | royalblue |
| SNORD15 | royalblue |
| SNORD17 | greenyellow |
| SNORD22 | royalblue |
| SNORD89 | royalblue |
| SNORD94 | royalblue |
| snoU13 | royalblue |
| snoU2-25 | salmon |
| snoU6-53 | royalblue |
| snoU6-77 | royalblue |
| snoU85 | salmon |
| snoU89 | salmon |
| snoU90 | blue |
| snoU97 | royalblue |
| SNRK | black |
| SNRNP25 | blue |
| SNRNP40 | green |
| SNRNP48 | brown |
| SNRNP70 | yellow |
| SNRPA | blue |
| SNRPA1 | green |
| SNRPB | blue |
| SNRPB2 | blue |
| SNRPC | magenta |
| SNRPD1 | green |
| SNRPD2 | blue |
| SNRPD3 | blue |
| SNRPE | green |
| SNRPF | green |
| SNRPG | darkturquoise |
| SNRPN | turquoise |
| SNTA1 | blue |
| SNTB1 | midnightblue |
| SNTB2 | blue |
| SNTG2 | blue |
| SNURF | royalblue |
| SNX1 | blue |
| SNX10 | brown |
| SNX11 | turquoise |
| SNX14 | blue |
| SNX16 | midnightblue |
| SNX18 | brown |
| SNX2 | brown |
| SNX20 | turquoise |
| SNX21 | blue |
| SNX22 | turquoise |
| SNX24 | brown |
| SNX25 | blue |
| SNX29 | black |
| SNX30 | blue |
| SNX33 | yellow |
| SNX5 | green |
| SNX6 | red |
| SNX7 | blue |
| SNX8 | blue |
| SNX9 | blue |
| SOAT1 | brown |
| SOBP | blue |
| SOCS1 | turquoise |
| SOCS2 | black |
| SOCS3 | brown |
| SOCS5 | blue |
| SOCS6 | red |
| SOCS7 | magenta |
| SOD1 | blue |
| SOD2 | brown |
| SOD3 | blue |
| SOLH | yellow |
| SON | turquoise |
| SORBS1 | blue |
| SORBS2 | brown |
| SORBS3 | lightyellow |
| SORCS2 | blue |
| SORD | turquoise |
| SORL1 | turquoise |
| SORT1 | turquoise |
| SOS1 | turquoise |
| SOS2 | red |
| SOX10 | tan |
| SOX12 | yellow |
| SOX13 | yellow |
| SOX18 | brown |
| SOX4 | magenta |
| SOX5 | blue |
| SOX6 | turquoise |
| SOX8 | brown |
| SOX9 | magenta |
| SP100 | turquoise |
| SP110 | turquoise |
| SP140 | turquoise |
| SP140L | turquoise |
| SP2 | yellow |
| SP4 | turquoise |
| SPA17 | turquoise |
| SPAG1 | greenyellow |
| SPAG16 | blue |
| SPAG4 | turquoise |
| SPAG5 | green |
| SPAG7 | brown |
| SPAG9 | grey60 |
| SPARC | purple |
| SPARCL1 | black |
| SPATA1 | turquoise |
| SPATA13 | turquoise |
| SPATA18 | blue |
| SPATA2 | yellow |
| SPATA20 | blue |
| SPATA24 | blue |
| SPATA2L | blue |
| SPATA5 | red |
| SPATA6 | blue |
| SPATA7 | blue |
| SPATC1 | darkred |
| SPATS2 | yellow |
| SPATS2L | blue |
| SPC24 | green |
| SPC25 | green |
| SPCS1 | turquoise |
| SPCS2 | turquoise |
| SPCS3 | turquoise |
| SPDEF | tan |
| SPDYE1 | yellow |
| SPDYE3 | yellow |
| SPEF2 | blue |
| SPEG | brown |
| SPESP1 | turquoise |
| SPG20 | blue |
| SPG7 | yellow |
| SPHK1 | grey60 |
| SPHK2 | yellow |
| SPI1 | brown |
| SPIB | turquoise |
| SPIN1 | brown |
| SPIN3 | brown |
| SPIN4 | brown |
| SPINK9 | darkgrey |
| SPINT1 | turquoise |
| SPIRE1 | cyan |
| SPIRE2 | brown |
| SPN | turquoise |
| SPNS1 | yellow |
| SPNS2 | blue |
| SPOCD1 | purple |
| SPOCK1 | purple |
| SPOCK2 | turquoise |
| SPON1 | blue |
| SPON2 | blue |
| SPOP | turquoise |
| SPOPL | blue |
| SPP1 | turquoise |
| SPP2 | darkgrey |
| SPR | blue |
| SPRED1 | blue |
| SPRED2 | blue |
| SPRY1 | black |
| SPRY2 | blue |
| SPRY3 | yellow |
| SPRY4 | lightyellow |
| SPSB1 | turquoise |
| SPSB2 | cyan |
| SPTB | brown |
| SPTBN1 | blue |
| SPTBN2 | brown |
| SPTBN5 | yellow |
| SPTLC2 | blue |
| SPTLC3 | brown |
| SQLE | midnightblue |
| SQRDL | brown |
| SQSTM1 | brown |
| SRC | yellow |
| SRCAP | yellow |
| SRCRB4D | turquoise |
| SRD5A1 | turquoise |
| SRD5A3 | tan |
| SREBF1 | brown |
| SREBF2 | yellow |
| SRF | magenta |
| SRGAP1 | lightyellow |
| SRGAP2 | blue |
| SRGAP2P1 | blue |
| SRGAP2P2 | brown |
| SRGAP3 | turquoise |
| SRGN | brown |
| SRI | red |
| SRM | yellow |
| SRP19 | blue |
| SRP54 | red |
| SRP68 | grey60 |
| SRP9 | green |
| SRP9L1 | blue |
| SRPK1 | green |
| SRPK2 | brown |
| SRPK3 | brown |
| SRPR | yellow |
| SRPRB | blue |
| SRPX | black |
| SRPX2 | purple |
| SRRD | yellow |
| SRRM1 | yellow |
| SRRM2 | yellow |
| SRRT | greenyellow |
| SRSF5 | black |
| SRSF8 | pink |
| SRXN1 | blue |
| SS18L1 | brown |
| SS18L2 | yellow |
| SSB | pink |
| SSBP1 | blue |
| SSBP3 | yellow |
| SSBP4 | brown |
| SSFA2 | pink |
| SSH1 | lightyellow |
| SSH2 | turquoise |
| SSH3 | brown |
| SSNA1 | blue |
| SSPN | cyan |
| SSPO | yellow |
| SSR1 | green |
| SSR2 | lightgreen |
| SSR3 | yellow |
| SSR4 | turquoise |
| SSRP1 | green |
| SSSCA1 | blue |
| SSU72 | greenyellow |
| SSX2IP | green |
| ST13 | turquoise |
| ST14 | turquoise |
| ST18 | brown |
| ST20 | blue |
| ST3GAL1 | turquoise |
| ST3GAL2 | turquoise |
| ST3GAL3 | yellow |
| ST3GAL4 | turquoise |
| ST3GAL5 | turquoise |
| ST3GAL6 | turquoise |
| ST5 | blue |
| ST6GAL1 | turquoise |
| ST6GAL2 | lightcyan |
| ST6GALNAC1 | darkgreen |
| ST6GALNAC2 | grey60 |
| ST6GALNAC4 | turquoise |
| ST6GALNAC5 | blue |
| ST6GALNAC6 | turquoise |
| ST7L | pink |
| ST8SIA1 | cyan |
| ST8SIA4 | turquoise |
| STAB1 | brown |
| STAC | brown |
| STAC2 | brown |
| STAC3 | brown |
| STAG1 | turquoise |
| STAG3 | turquoise |
| STAG3L1 | yellow |
| STAG3L2 | yellow |
| STAG3L3 | yellow |
| STAG3L4 | blue |
| STAM | yellow |
| STAMBP | red |
| STAMBPL1 | turquoise |
| STAP2 | blue |
| STARD10 | brown |
| STARD13 | blue |
| STARD3 | turquoise |
| STARD3NL | brown |
| STARD4 | turquoise |
| STARD5 | turquoise |
| STARD8 | black |
| STARD9 | black |
| STAT1 | turquoise |
| STAT2 | turquoise |
| STAT3 | turquoise |
| STAT4 | turquoise |
| STAT5A | turquoise |
| STAT5B | turquoise |
| STAT6 | turquoise |
| STAU1 | greenyellow |
| STAU2 | midnightblue |
| STBD1 | blue |
| STC2 | turquoise |
| STEAP1 | blue |
| STEAP2 | blue |
| STEAP3 | blue |
| STEAP4 | blue |
| STIL | green |
| STIM1 | blue |
| STIM2 | turquoise |
| STIP1 | blue |
| STK10 | turquoise |
| STK11 | blue |
| STK11IP | yellow |
| STK17A | turquoise |
| STK17B | turquoise |
| STK19 | magenta |
| STK24 | yellow |
| STK25 | yellow |
| STK3 | midnightblue |
| STK32A | blue |
| STK32C | blue |
| STK33 | cyan |
| STK35 | yellow |
| STK36 | yellow |
| STK38 | magenta |
| STK38L | cyan |
| STK39 | turquoise |
| STK4 | turquoise |
| STK40 | yellow |
| STMN1 | green |
| STMN2 | blue |
| STMN3 | turquoise |
| STOM | brown |
| STOML1 | blue |
| STOML2 | blue |
| STON1 | blue |
| STON2 | blue |
| STOX2 | yellow |
| STRA13 | grey60 |
| STRA6 | turquoise |
| STRADA | turquoise |
| STRADB | pink |
| STRAP | cyan |
| STRBP | brown |
| STRN | turquoise |
| STRN3 | red |
| STRN4 | yellow |
| STS | blue |
| STT3A | red |
| STT3B | turquoise |
| STUB1 | turquoise |
| STX10 | blue |
| STX11 | turquoise |
| STX12 | blue |
| STX16 | turquoise |
| STX17 | turquoise |
| STX19 | brown |
| STX1A | brown |
| STX2 | blue |
| STX3 | red |
| STX4 | brown |
| STX6 | green |
| STX7 | turquoise |
| STXBP2 | yellow |
| STXBP3 | pink |
| STXBP4 | blue |
| STXBP5 | blue |
| STYK1 | blue |
| STYX | red |
| STYXL1 | turquoise |
| SUB1 | green |
| SUCLA2 | blue |
| SUCLG2 | blue |
| SUCNR1 | brown |
| SUFU | lightyellow |
| SUGP2 | yellow |
| SUGT1 | red |
| SUGT1L1 | blue |
| SULF1 | lightcyan |
| SULF2 | blue |
| SULT1A1 | blue |
| SULT1A3 | yellow |
| SULT1C2 | brown |
| SULT2B1 | tan |
| SUMF1 | turquoise |
| SUMF2 | brown |
| SUMO1 | turquoise |
| SUMO2 | grey60 |
| SUMO3 | green |
| SUMO4 | turquoise |
| SUN1 | turquoise |
| SUN2 | yellow |
| SUOX | darkgreen |
| SUPT16H | green |
| SUPT3H | magenta |
| SUPT5H | yellow |
| SUPT6H | turquoise |
| SUPV3L1 | yellow |
| SURF1 | turquoise |
| SURF2 | blue |
| SURF6 | blue |
| SUSD1 | brown |
| SUSD2 | blue |
| SUSD3 | turquoise |
| SUSD4 | brown |
| SUSD5 | blue |
| SUV39H1 | green |
| SUV39H2 | green |
| SUV420H1 | yellow |
| SUV420H2 | yellow |
| SV2B | turquoise |
| SVEP1 | black |
| SVIL | yellow |
| SVIP | red |
| SVOPL | brown |
| SWAP70 | blue |
| SYAP1 | turquoise |
| SYBU | darkturquoise |
| SYCE1L | brown |
| SYCP2 | brown |
| SYCP2L | yellow |
| SYCP3 | black |
| SYDE1 | lightcyan |
| SYDE2 | brown |
| SYK | turquoise |
| SYMPK | yellow |
| SYNCRIP | red |
| SYNE1 | turquoise |
| SYNE2 | turquoise |
| SYNGAP1 | lightyellow |
| SYNGR1 | brown |
| SYNGR2 | grey60 |
| SYNGR3 | turquoise |
| SYNJ1 | turquoise |
| SYNJ2 | yellow |
| SYNJ2BP | blue |
| SYNM | blue |
| SYNPO | lightyellow |
| SYNPO2 | black |
| SYNRG | turquoise |
| SYP | turquoise |
| SYPL1 | turquoise |
| SYS1 | turquoise |
| SYT1 | blue |
| SYT11 | brown |
| SYT15 | turquoise |
| SYT7 | magenta |
| SYT8 | brown |
| SYTL1 | turquoise |
| SYTL2 | lightcyan |
| SYTL3 | turquoise |
| SYTL4 | blue |
| SYVN1 | yellow |
| TAB1 | yellow |
| TAB2 | pink |
| TAB3 | yellow |
| TAC4 | turquoise |
| TACC1 | black |
| TACC2 | magenta |
| TACC3 | green |
| TACO1 | grey60 |
| TACR1 | black |
| TACSTD2 | turquoise |
| TADA1 | green |
| TADA3 | yellow |
| TAF10 | turquoise |
| TAF11 | magenta |
| TAF12 | brown |
| TAF13 | turquoise |
| TAF15 | turquoise |
| TAF1A | red |
| TAF1B | yellow |
| TAF1C | yellow |
| TAF1D | pink |
| TAF2 | midnightblue |
| TAF4 | greenyellow |
| TAF4B | purple |
| TAF5L | green |
| TAF6 | yellow |
| TAF8 | magenta |
| TAF9B | red |
| TAF9BP1 | turquoise |
| TAF9P3 | pink |
| TAGAP | turquoise |
| TAGLN | blue |
| TAGLN2 | lightgreen |
| TAGLN2P1 | midnightblue |
| TALDO1 | blue |
| TANC1 | yellow |
| TANC2 | lightcyan |
| TANK | turquoise |
| TAP1 | turquoise |
| TAP2 | turquoise |
| TAPBP | turquoise |
| TAPBPL | turquoise |
| TAPT1 | turquoise |
| TARBP1 | red |
| TARS | turquoise |
| TARS2 | lightgreen |
| TARSL2 | turquoise |
| TAS1R3 | turquoise |
| TAS2R13 | cyan |
| TAS2R14 | cyan |
| TAS2R19 | cyan |
| TAS2R20 | cyan |
| TAS2R3 | pink |
| TAS2R31 | cyan |
| TAS2R4 | yellow |
| TAS2R5 | yellow |
| TAS2R50 | cyan |
| TASP1 | yellow |
| TATDN1 | midnightblue |
| TATDN2 | yellow |
| TAX1BP1 | tan |
| TAX1BP3 | turquoise |
| TAZ | turquoise |
| TBC1D1 | blue |
| TBC1D10A | blue |
| TBC1D10C | turquoise |
| TBC1D14 | turquoise |
| TBC1D16 | brown |
| TBC1D17 | yellow |
| TBC1D19 | blue |
| TBC1D2 | purple |
| TBC1D22B | magenta |
| TBC1D23 | red |
| TBC1D24 | tan |
| TBC1D25 | yellow |
| TBC1D2B | turquoise |
| TBC1D3 | yellow |
| TBC1D30 | turquoise |
| TBC1D4 | turquoise |
| TBC1D5 | turquoise |
| TBC1D7 | brown |
| TBC1D8 | brown |
| TBC1D8B | yellow |
| TBC1D9 | black |
| TBC1D9B | yellow |
| TBCA | blue |
| TBCB | blue |
| TBCC | magenta |
| TBCD | grey60 |
| TBCE | green |
| TBCEL | turquoise |
| TBCK | blue |
| TBKBP1 | blue |
| TBL1X | magenta |
| TBL1XR1 | green |
| TBL2 | blue |
| TBP | pink |
| TBPL1 | pink |
| TBRG1 | turquoise |
| TBRG4 | green |
| TBX15 | black |
| TBX18 | blue |
| TBX19 | yellow |
| TBX2 | blue |
| TBX3 | yellow |
| TBXAS1 | brown |
| TC2N | brown |
| TCEA1 | midnightblue |
| TCEA1P | yellow |
| TCEA2 | yellow |
| TCEA3 | brown |
| TCEAL1 | blue |
| TCEAL3 | blue |
| TCEAL4 | blue |
| TCEAL8 | blue |
| TCEANC | turquoise |
| TCEB1 | midnightblue |
| TCEB1P19 | pink |
| TCEB2 | blue |
| TCEB3CL | blue |
| TCF19 | green |
| TCF20 | greenyellow |
| TCF25 | yellow |
| TCF3 | yellow |
| TCF4 | blue |
| TCF7 | turquoise |
| TCF7L1 | magenta |
| TCF7L2 | yellow |
| TCFL5 | turquoise |
| TCIRG1 | turquoise |
| TCL6 | blue |
| TCN2 | turquoise |
| TCOF1 | yellow |
| TCP1 | green |
| TCP11L1 | blue |
| TCP11L2 | black |
| TCTA | blue |
| TCTEX1D2 | blue |
| TCTEX1D4 | blue |
| TCTN1 | blue |
| TCTN2 | yellow |
| TDG | green |
| TDP1 | green |
| TDRD1 | tan |
| TDRD3 | blue |
| TDRD5 | brown |
| TDRD7 | turquoise |
| TDRD9 | blue |
| TDRKH | lightgreen |
| TEAD1 | blue |
| TEAD2 | magenta |
| TEAD3 | magenta |
| TEAD4 | cyan |
| TEC | brown |
| TECPR1 | turquoise |
| TECR | blue |
| TEF | blue |
| TEK | black |
| TELO2 | blue |
| Telomerase-vert | blue |
| TENC1 | black |
| TEP1 | turquoise |
| TERF1 | midnightblue |
| TERT | blue |
| TES | yellow |
| TESC | turquoise |
| TESK1 | yellow |
| TESK2 | yellow |
| TET1 | magenta |
| TET2 | blue |
| TET3 | yellow |
| TETRAN | blue |
| TEX10 | red |
| TEX2 | grey60 |
| TEX261 | red |
| TEX264 | yellow |
| TEX9 | blue |
| TF | turquoise |
| TFAM | blue |
| TFAP2A | magenta |
| TFAP2B | tan |
| TFAP2C | brown |
| TFAP2E | yellow |
| TFAP4 | blue |
| TFB1M | pink |
| TFB2M | green |
| TFCP2 | yellow |
| TFCP2L1 | brown |
| TFDP1 | blue |
| TFDP2 | brown |
| TFEB | turquoise |
| TFEC | turquoise |
| TFF3 | green |
| TFG | turquoise |
| TFPI | black |
| TFPI2 | turquoise |
| TFPT | blue |
| TFRC | green |
| TGDS | blue |
| TGFA | cyan |
| TGFB1 | brown |
| TGFB1I1 | purple |
| TGFB2 | turquoise |
| TGFB3 | blue |
| TGFBI | brown |
| TGFBR1 | lightcyan |
| TGFBR2 | black |
| TGFBR3 | blue |
| TGIF1 | turquoise |
| TGIF2 | magenta |
| TGM2 | brown |
| TGM5 | turquoise |
| TGOLN2 | yellow |
| TGS1 | midnightblue |
| TH1L | brown |
| THADA | pink |
| THAP4 | blue |
| THAP6 | turquoise |
| THAP7 | blue |
| THAP8 | brown |
| THAP9 | red |
| THBS1 | purple |
| THBS2 | purple |
| THBS3 | yellow |
| THBS4 | black |
| THEM4 | yellow |
| THNSL1 | brown |
| THNSL2 | brown |
| THOC1 | green |
| THOC2 | red |
| THOC3 | green |
| THOC4 | grey60 |
| THOC5 | turquoise |
| THOC6 | blue |
| THOP1 | blue |
| THRA | blue |
| THRB | blue |
| THRSP | tan |
| THSD1 | black |
| THSD4 | blue |
| THSD7A | black |
| THSD7B | blue |
| THTPA | turquoise |
| THUMPD3 | turquoise |
| THY1 | purple |
| THYN1 | turquoise |
| TIA1 | pink |
| TIAF1 | turquoise |
| TIAL1 | yellow |
| TIAM1 | brown |
| TIAM2 | red |
| TICAM1 | yellow |
| TIE1 | black |
| TIFA | blue |
| TIGD2 | red |
| TIGD5 | darkred |
| TIGD7 | red |
| TIGIT | turquoise |
| TIMELESS | green |
| TIMM10 | blue |
| TIMM13 | blue |
| TIMM17A | green |
| TIMM17B | blue |
| TIMM23 | green |
| TIMM44 | blue |
| TIMM50 | green |
| TIMM8A | blue |
| TIMM8B | yellow |
| TIMP1 | black |
| TIMP2 | purple |
| TIMP3 | blue |
| TIMP4 | black |
| TINAGL1 | blue |
| TIPARP | turquoise |
| TIPIN | green |
| TIPRL | green |
| TIRAP | turquoise |
| TJAP1 | magenta |
| TJP1 | blue |
| TJP2 | brown |
| TJP3 | brown |
| TK1 | green |
| TK2 | blue |
| TKT | blue |
| TLCD1 | blue |
| TLCD2 | blue |
| TLE1 | brown |
| TLE2 | magenta |
| TLE3 | yellow |
| TLE4 | turquoise |
| TLE6 | turquoise |
| TLK1 | yellow |
| TLL1 | black |
| TLN1 | lightyellow |
| TLN2 | lightyellow |
| TLR1 | brown |
| TLR2 | brown |
| TLR3 | turquoise |
| TLR4 | brown |
| TLR5 | red |
| TLR6 | turquoise |
| TLR7 | turquoise |
| TLR8 | turquoise |
| TM2D2 | brown |
| TM2D3 | blue |
| TM4SF1 | turquoise |
| TM4SF18 | tan |
| TM6SF1 | brown |
| TM7SF2 | brown |
| TM7SF3 | cyan |
| TM9SF1 | tan |
| TM9SF2 | turquoise |
| TMBIM1 | blue |
| TMBIM4 | brown |
| TMBIM6 | tan |
| TMC1 | turquoise |
| TMC4 | brown |
| TMC5 | tan |
| TMC6 | turquoise |
| TMC7 | turquoise |
| TMC8 | turquoise |
| TMCC2 | blue |
| TMCC3 | brown |
| TMCO1 | turquoise |
| TMCO3 | turquoise |
| TMCO4 | yellow |
| TMCO6 | turquoise |
| TMED1 | blue |
| TMED2 | turquoise |
| TMED3 | turquoise |
| TMED5 | red |
| TMED7 | brown |
| TMED7-TICAM2 | brown |
| TMED9 | turquoise |
| TMEFF1 | turquoise |
| TMEM101 | salmon |
| TMEM102 | blue |
| TMEM104 | yellow |
| TMEM105 | turquoise |
| TMEM106A | black |
| TMEM106B | turquoise |
| TMEM106C | turquoise |
| TMEM107 | greenyellow |
| TMEM108 | yellow |
| TMEM109 | blue |
| TMEM110 | turquoise |
| TMEM111 | yellow |
| TMEM116 | brown |
| TMEM119 | brown |
| TMEM120A | brown |
| TMEM120B | yellow |
| TMEM121 | brown |
| TMEM123 | brown |
| TMEM125 | brown |
| TMEM126A | yellow |
| TMEM126B | greenyellow |
| TMEM128 | blue |
| TMEM129 | turquoise |
| TMEM130 | blue |
| TMEM132A | turquoise |
| TMEM133 | blue |
| TMEM134 | brown |
| TMEM135 | blue |
| TMEM136 | blue |
| TMEM139 | brown |
| TMEM140 | turquoise |
| TMEM141 | turquoise |
| TMEM144 | blue |
| TMEM147 | blue |
| TMEM149 | turquoise |
| TMEM14A | turquoise |
| TMEM14B | blue |
| TMEM14C | yellow |
| TMEM14E | turquoise |
| TMEM150A | blue |
| TMEM150B | brown |
| TMEM150C | blue |
| TMEM151B | magenta |
| TMEM154 | turquoise |
| TMEM156 | turquoise |
| TMEM158 | blue |
| TMEM159 | turquoise |
| TMEM160 | blue |
| TMEM161A | blue |
| TMEM163 | turquoise |
| TMEM164 | blue |
| TMEM167A | greenyellow |
| TMEM17 | turquoise |
| TMEM170A | red |
| TMEM173 | turquoise |
| TMEM175 | turquoise |
| TMEM176A | turquoise |
| TMEM176B | turquoise |
| TMEM177 | blue |
| TMEM18 | red |
| TMEM180 | brown |
| TMEM181 | pink |
| TMEM183A | green |
| TMEM184A | turquoise |
| TMEM184B | yellow |
| TMEM185A | yellow |
| TMEM185B | red |
| TMEM186 | brown |
| TMEM187 | blue |
| TMEM189 | turquoise |
| TMEM19 | turquoise |
| TMEM191A | brown |
| TMEM192 | blue |
| TMEM194A | green |
| TMEM194B | turquoise |
| TMEM195 | blue |
| TMEM198 | brown |
| TMEM2 | blue |
| TMEM20 | blue |
| TMEM200A | blue |
| TMEM200B | blue |
| TMEM200C | cyan |
| TMEM201 | yellow |
| TMEM203 | blue |
| TMEM204 | black |
| TMEM205 | yellow |
| TMEM206 | green |
| TMEM208 | blue |
| TMEM209 | green |
| TMEM212 | darkgrey |
| TMEM214 | yellow |
| TMEM216 | salmon |
| TMEM217 | blue |
| TMEM218 | turquoise |
| TMEM22 | blue |
| TMEM220 | black |
| TMEM223 | blue |
| TMEM229B | turquoise |
| TMEM231 | turquoise |
| TMEM25 | brown |
| TMEM26 | blue |
| TMEM30A | brown |
| TMEM30B | turquoise |
| TMEM33 | red |
| TMEM37 | brown |
| TMEM38A | blue |
| TMEM38B | blue |
| TMEM40 | turquoise |
| TMEM41A | brown |
| TMEM41B | greenyellow |
| TMEM42 | yellow |
| TMEM43 | blue |
| TMEM44 | blue |
| TMEM45A | red |
| TMEM45B | tan |
| TMEM46 | blue |
| TMEM47 | blue |
| TMEM48 | green |
| TMEM49 | lightcyan |
| TMEM50B | blue |
| TMEM51 | brown |
| TMEM52 | blue |
| TMEM53 | yellow |
| TMEM54 | turquoise |
| TMEM55A | blue |
| TMEM56 | brown |
| TMEM59 | yellow |
| TMEM60 | yellow |
| TMEM62 | tan |
| TMEM63A | yellow |
| TMEM63B | magenta |
| TMEM63C | tan |
| TMEM64 | midnightblue |
| TMEM65 | midnightblue |
| TMEM66 | turquoise |
| TMEM67 | red |
| TMEM68 | midnightblue |
| TMEM69 | green |
| TMEM70 | midnightblue |
| TMEM71 | turquoise |
| TMEM79 | lightgreen |
| TMEM80 | yellow |
| TMEM86A | tan |
| TMEM86B | turquoise |
| TMEM87A | red |
| TMEM87B | blue |
| TMEM8A | salmon |
| TMEM8B | brown |
| TMEM9 | brown |
| TMEM90B | purple |
| TMEM91 | blue |
| TMEM92 | salmon |
| TMEM93 | blue |
| TMEM97 | green |
| TMEM98 | blue |
| TMEM99 | turquoise |
| TMEM9B | brown |
| TMLHE | pink |
| TMOD2 | blue |
| TMOD3 | blue |
| TMPO | green |
| TMPRSS13 | brown |
| TMPRSS2 | brown |
| TMPRSS3 | blue |
| TMSB10 | blue |
| TMSB15A | brown |
| TMSB15B | brown |
| TMSB4X | brown |
| TMSL3 | turquoise |
| TMTC1 | black |
| TMTC2 | blue |
| TMTC3 | red |
| TMTC4 | brown |
| TMUB1 | blue |
| TMX1 | red |
| TMX2 | turquoise |
| TMX3 | blue |
| TMX4 | blue |
| TNC | blue |
| TNF | turquoise |
| TNFAIP1 | blue |
| TNFAIP2 | turquoise |
| TNFAIP3 | turquoise |
| TNFAIP6 | lightcyan |
| TNFAIP8 | turquoise |
| TNFAIP8L1 | brown |
| TNFAIP8L2 | turquoise |
| TNFAIP8L3 | blue |
| TNFRSF10A | turquoise |
| TNFRSF10B | blue |
| TNFRSF10C | brown |
| TNFRSF10D | blue |
| TNFRSF11A | yellow |
| TNFRSF11B | brown |
| TNFRSF12A | turquoise |
| TNFRSF14 | turquoise |
| TNFRSF17 | turquoise |
| TNFRSF18 | turquoise |
| TNFRSF19 | blue |
| TNFRSF1A | brown |
| TNFRSF1B | turquoise |
| TNFRSF21 | magenta |
| TNFRSF25 | turquoise |
| TNFRSF4 | turquoise |
| TNFSF10 | turquoise |
| TNFSF11 | blue |
| TNFSF12 | turquoise |
| TNFSF13 | brown |
| TNFSF13B | turquoise |
| TNFSF14 | turquoise |
| TNFSF15 | turquoise |
| TNFSF4 | turquoise |
| TNFSF8 | turquoise |
| TNFSF9 | brown |
| TNIK | turquoise |
| TNIP1 | turquoise |
| TNIP2 | turquoise |
| TNK1 | brown |
| TNK2 | yellow |
| TNKS | blue |
| TNKS1BP1 | turquoise |
| TNNI2 | brown |
| TNNI3K | red |
| TNNT2 | cyan |
| TNPO2 | yellow |
| TNPO3 | blue |
| TNRC18 | yellow |
| TNRC6B | turquoise |
| TNS1 | blue |
| TNS3 | brown |
| TNS4 | blue |
| TNXB | black |
| TOB1 | greenyellow |
| TOB2 | yellow |
| TOLLIP | blue |
| TOM1 | yellow |
| TOM1L1 | brown |
| TOM1L2 | yellow |
| TOMM20 | green |
| TOMM22 | blue |
| TOMM34 | green |
| TOMM40 | green |
| TOMM40L | lightgreen |
| TOMM5 | blue |
| TOMM6 | magenta |
| TOMM7 | salmon |
| TOMM70A | turquoise |
| TOP1MT | darkred |
| TOP1P1 | darkgreen |
| TOP2A | green |
| TOP2B | yellow |
| TOP3A | yellow |
| TOP3B | yellow |
| TOPBP1 | green |
| TOR2A | brown |
| TOR3A | turquoise |
| TOX4 | greenyellow |
| TP53 | turquoise |
| TP53BP2 | blue |
| TP53I11 | yellow |
| TP53I13 | turquoise |
| TP53I3 | blue |
| TP53INP1 | turquoise |
| TP53INP2 | yellow |
| TP53RK | midnightblue |
| TP53TG1 | blue |
| TP63 | blue |
| TPBG | blue |
| TPCN1 | lightyellow |
| TPCN2 | yellow |
| TPD52 | darkred |
| TPD52L1 | brown |
| TPD52L2 | turquoise |
| TPI1 | cyan |
| TPI1P3 | brown |
| TPK1 | turquoise |
| TPM1 | lightcyan |
| TPM2 | blue |
| TPM3 | lightgreen |
| TPM4 | purple |
| TPMT | green |
| TPP1 | brown |
| TPP2 | turquoise |
| TPPP | yellow |
| TPPP3 | blue |
| TPRG1 | black |
| TPRG1L | turquoise |
| TPRKB | blue |
| TPSAB1 | black |
| TPST1 | blue |
| TPST2 | turquoise |
| TPT1 | blue |
| TPX2 | green |
| TRA2A | lightyellow |
| TRABD | turquoise |
| TRAC | turquoise |
| TRADD | turquoise |
| TRAF1 | turquoise |
| TRAF2 | blue |
| TRAF3 | turquoise |
| TRAF3IP1 | yellow |
| TRAF3IP2 | red |
| TRAF3IP3 | turquoise |
| TRAF4 | brown |
| TRAF5 | turquoise |
| TRAF7 | turquoise |
| TRAFD1 | turquoise |
| TRAIP | green |
| TRAK1 | blue |
| TRAK2 | blue |
| TRAM1 | midnightblue |
| TRAM1L1 | brown |
| TRAM2 | black |
| TRANK1 | turquoise |
| TRAP1 | blue |
| TRAPPC1 | yellow |
| TRAPPC2 | red |
| TRAPPC2L | blue |
| TRAPPC4 | brown |
| TRAPPC5 | yellow |
| TRAPPC6A | blue |
| TRAPPC9 | darkred |
| TRBC2 | turquoise |
| TRBV20-1 | turquoise |
| TRBV28 | turquoise |
| TRBV29-1 | turquoise |
| TRBV5-1 | turquoise |
| TRDMT1 | blue |
| TREM1 | brown |
| TREM2 | brown |
| TRERF1 | turquoise |
| TREX1 | turquoise |
| TREX2 | brown |
| TRGV3 | turquoise |
| TRIAP1 | blue |
| TRIB1 | yellow |
| TRIB2 | blue |
| TRIB3 | turquoise |
| TRIM11 | lightgreen |
| TRIM14 | turquoise |
| TRIM16 | blue |
| TRIM16L | blue |
| TRIM17 | yellow |
| TRIM2 | brown |
| TRIM21 | turquoise |
| TRIM22 | turquoise |
| TRIM24 | greenyellow |
| TRIM25 | turquoise |
| TRIM26 | magenta |
| TRIM28 | yellow |
| TRIM29 | brown |
| TRIM3 | yellow |
| TRIM32 | blue |
| TRIM34 | turquoise |
| TRIM35 | turquoise |
| TRIM36 | tan |
| TRIM37 | grey60 |
| TRIM38 | turquoise |
| TRIM39 | magenta |
| TRIM4 | yellow |
| TRIM41 | blue |
| TRIM44 | red |
| TRIM45 | brown |
| TRIM46 | brown |
| TRIM47 | grey60 |
| TRIM5 | turquoise |
| TRIM52 | turquoise |
| TRIM56 | turquoise |
| TRIM59 | green |
| TRIM6 | blue |
| TRIM60P18 | pink |
| TRIM62 | yellow |
| TRIM65 | grey60 |
| TRIM66 | blue |
| TRIM68 | blue |
| TRIM69 | turquoise |
| TRIM73 | turquoise |
| TRIM8 | lightyellow |
| TRIO | red |
| TRIOBP | blue |
| TRIP10 | blue |
| TRIP13 | green |
| TRIP6 | brown |
| TRIT1 | green |
| TRMT1 | blue |
| TRMT11 | red |
| TRMT12 | midnightblue |
| TRMT2A | yellow |
| TRMT6 | green |
| TRMT61A | blue |
| TRMT61B | red |
| TRNAU1AP | brown |
| TRNP1 | turquoise |
| TRNT1 | yellow |
| TRO | blue |
| TROAP | green |
| TRPA1 | black |
| TRPC1 | blue |
| TRPM2 | brown |
| TRPM4 | turquoise |
| TRPM6 | blue |
| TRPM8 | turquoise |
| TRPS1 | brown |
| TRPT1 | turquoise |
| TRPV1 | yellow |
| TRPV2 | turquoise |
| TRPV4 | turquoise |
| TRPV6 | tan |
| TRRAP | yellow |
| TRUB1 | green |
| TRUB2 | blue |
| TSC1 | blue |
| TSC2 | yellow |
| TSC22D1 | blue |
| TSC22D2 | yellow |
| TSC22D3 | turquoise |
| TSC22D4 | turquoise |
| TSEN15 | green |
| TSEN2 | yellow |
| TSEN34 | blue |
| TSEN54 | grey60 |
| TSFM | blue |
| TSGA10 | brown |
| TSGA14 | blue |
| TSHZ1 | blue |
| TSHZ2 | black |
| TSHZ3 | purple |
| TSKU | tan |
| TSN | red |
| TSNARE1 | darkred |
| TSPAN1 | blue |
| TSPAN10 | turquoise |
| TSPAN12 | brown |
| TSPAN13 | turquoise |
| TSPAN14 | turquoise |
| TSPAN15 | purple |
| TSPAN17 | blue |
| TSPAN18 | lightyellow |
| TSPAN2 | blue |
| TSPAN3 | blue |
| TSPAN31 | blue |
| TSPAN32 | turquoise |
| TSPAN33 | blue |
| TSPAN4 | brown |
| TSPAN5 | blue |
| TSPAN6 | turquoise |
| TSPAN7 | black |
| TSPAN9 | purple |
| TSPO | turquoise |
| TSPYL1 | pink |
| TSPYL2 | turquoise |
| TSPYL3 | brown |
| TSPYL4 | pink |
| TSPYL5 | brown |
| TSR1 | blue |
| TSR2 | brown |
| TSSC1 | red |
| TSSC4 | blue |
| TSSK3 | yellow |
| TST | yellow |
| TSTA3 | darkred |
| TSTD1 | brown |
| TSTD2 | blue |
| TTC12 | blue |
| TTC13 | red |
| TTC14 | turquoise |
| TTC17 | red |
| TTC18 | black |
| TTC21A | blue |
| TTC21B | turquoise |
| TTC22 | blue |
| TTC23 | blue |
| TTC26 | turquoise |
| TTC27 | green |
| TTC28 | black |
| TTC28AS | yellow |
| TTC3 | pink |
| TTC30A | red |
| TTC31 | turquoise |
| TTC32 | pink |
| TTC33 | red |
| TTC35 | midnightblue |
| TTC36 | tan |
| TTC38 | turquoise |
| TTC39A | brown |
| TTC39B | brown |
| TTC39C | turquoise |
| TTC3L | blue |
| TTC4 | green |
| TTC5 | black |
| TTC7A | turquoise |
| TTC7B | blue |
| TTC8 | blue |
| TTC9 | brown |
| TTF2 | green |
| TTK | green |
| TTLL1 | brown |
| TTLL11 | yellow |
| TTLL12 | blue |
| TTLL3 | lightyellow |
| TTLL4 | magenta |
| TTLL5 | yellow |
| TTLL7 | turquoise |
| TTN | turquoise |
| TTPAL | yellow |
| TTYH1 | brown |
| TTYH2 | turquoise |
| TTYH3 | yellow |
| TUB | blue |
| TUBA1A | blue |
| TUBA1B | darkturquoise |
| TUBA1C | green |
| TUBA4A | turquoise |
| TUBB | green |
| TUBB2A | blue |
| TUBB2B | blue |
| TUBB2C | blue |
| TUBB3 | blue |
| TUBB6 | blue |
| TUBBP5 | brown |
| TUBD1 | grey60 |
| TUBE1 | pink |
| TUBG1 | green |
| TUBG2 | blue |
| TUBGCP3 | yellow |
| TUBGCP6 | turquoise |
| TUFM | blue |
| TUFT1 | brown |
| TUG1 | yellow |
| TULP3 | cyan |
| TULP4 | pink |
| TUSC1 | turquoise |
| TUSC3 | turquoise |
| TUT1 | salmon |
| TWF1 | red |
| TWIST1 | blue |
| TWISTNB | turquoise |
| TWSG1 | lightcyan |
| TXLNB | turquoise |
| TXN | blue |
| TXN2 | blue |
| TXNDC11 | turquoise |
| TXNDC15 | turquoise |
| TXNDC16 | darkgreen |
| TXNDC17 | blue |
| TXNDC3 | turquoise |
| TXNDC5 | turquoise |
| TXNIP | turquoise |
| TXNL4A | blue |
| TXNRD1 | midnightblue |
| TXNRD2 | yellow |
| TXNRD3 | turquoise |
| TYK2 | turquoise |
| TYMP | brown |
| TYMS | green |
| TYRO3 | turquoise |
| TYROBP | brown |
| TYSND1 | blue |
| TYW1B | yellow |
| TYW3 | pink |
| U1 | royalblue |
| U11 | royalblue |
| U12 | royalblue |
| U2 | greenyellow |
| U2AF1 | green |
| U2AF1L4 | turquoise |
| U3 | brown |
| U4 | royalblue |
| U47924.1 | cyan |
| U47924.7 | cyan |
| U4atac | royalblue |
| U5 | royalblue |
| U6 | red |
| U6atac | royalblue |
| UACA | blue |
| UAP1 | turquoise |
| UAP1L1 | brown |
| UBA1 | yellow |
| UBA2 | blue |
| UBA52 | salmon |
| UBA6 | blue |
| UBA7 | turquoise |
| UBAC2 | blue |
| UBAP2 | brown |
| UBAP2L | lightgreen |
| UBASH3A | turquoise |
| UBASH3B | brown |
| UBB | yellow |
| UBD | turquoise |
| UBE2B | blue |
| UBE2C | green |
| UBE2CBP | red |
| UBE2D1 | red |
| UBE2D4 | yellow |
| UBE2E1 | yellow |
| UBE2E2 | blue |
| UBE2E3 | magenta |
| UBE2F | brown |
| UBE2H | turquoise |
| UBE2I | greenyellow |
| UBE2J2 | yellow |
| UBE2L6 | turquoise |
| UBE2M | blue |
| UBE2O | grey60 |
| UBE2Q1 | lightgreen |
| UBE2Q2 | blue |
| UBE2Q2P1 | blue |
| UBE2S | green |
| UBE2T | green |
| UBE2V1 | yellow |
| UBE2V1P1 | lightyellow |
| UBE2V2 | midnightblue |
| UBE2W | midnightblue |
| UBE2Z | grey60 |
| UBE3A | turquoise |
| UBE3C | green |
| UBE4A | red |
| UBE4B | red |
| UBFD1 | red |
| UBIAD1 | turquoise |
| UBL3 | blue |
| UBL4A | blue |
| UBL5 | yellow |
| UBLCP1 | blue |
| UBN1 | yellow |
| UBN2 | yellow |
| UBOX5 | yellow |
| UBP1 | yellow |
| UBQLN2 | yellow |
| UBQLN4 | lightgreen |
| UBR1 | blue |
| UBR5 | midnightblue |
| UBR7 | green |
| UBTD1 | lightcyan |
| UBTD2 | blue |
| UBXN11 | brown |
| UBXN2A | red |
| UBXN2B | midnightblue |
| UBXN7 | yellow |
| UBXN8 | blue |
| UCHL1 | blue |
| UCHL3 | turquoise |
| UCHL5 | green |
| UCK2 | lightgreen |
| UCKL1 | yellow |
| UCP2 | turquoise |
| UFC1 | lightgreen |
| UFM1 | yellow |
| UFSP1 | blue |
| UGCG | turquoise |
| UGDH | purple |
| UGGT2 | turquoise |
| UGP2 | red |
| UGT8 | brown |
| UHMK1 | blue |
| UHRF1 | green |
| UHRF1BP1 | magenta |
| UHRF2 | turquoise |
| UIMC1 | turquoise |
| ULK1 | yellow |
| ULK2 | blue |
| ULK3 | yellow |
| ULK4 | blue |
| UMPS | green |
| UNC119 | turquoise |
| UNC119B | pink |
| UNC13B | brown |
| UNC13D | turquoise |
| UNC45A | turquoise |
| UNC5B | lightcyan |
| UNC5C | blue |
| UNC5CL | magenta |
| UNC93B1 | brown |
| UNG | green |
| UNK | yellow |
| UPF2 | green |
| UPF3A | brown |
| UPK3B | yellow |
| UPK3BL | yellow |
| UPP1 | brown |
| UQCC | yellow |
| UQCR10 | blue |
| UQCR11 | yellow |
| UQCRB | midnightblue |
| UQCRBP1 | blue |
| UQCRC1 | blue |
| UQCRC2 | red |
| UQCRFS1 | blue |
| UQCRH | blue |
| UQCRQ | yellow |
| URB1 | green |
| URB2 | green |
| URGCP | brown |
| USE1 | brown |
| USF1 | turquoise |
| USMG5 | yellow |
| USP1 | green |
| USP10 | greenyellow |
| USP11 | lightyellow |
| USP13 | green |
| USP14 | green |
| USP18 | turquoise |
| USP20 | turquoise |
| USP21 | lightgreen |
| USP22 | yellow |
| USP25 | pink |
| USP28 | red |
| USP3 | turquoise |
| USP30 | turquoise |
| USP31 | yellow |
| USP32 | grey60 |
| USP35 | yellow |
| USP36 | yellow |
| USP37 | red |
| USP4 | turquoise |
| USP40 | blue |
| USP43 | turquoise |
| USP45 | red |
| USP46 | red |
| USP49 | magenta |
| USP5 | cyan |
| USP53 | blue |
| USP54 | turquoise |
| USP6NL | lightgreen |
| USP7 | red |
| UST | blue |
| UTP14A | green |
| UTP18 | grey60 |
| UTP20 | green |
| UTP23 | midnightblue |
| UTP6 | yellow |
| UTRN | turquoise |
| UTS2 | turquoise |
| UXT | yellow |
| VAC14 | yellow |
| VAMP1 | turquoise |
| VAMP2 | yellow |
| VAMP3 | blue |
| VAMP5 | turquoise |
| VAMP7 | green |
| VAMP8 | blue |
| VANGL1 | turquoise |
| VANGL2 | magenta |
| VAPA | turquoise |
| VAPB | greenyellow |
| VARS | magenta |
| VARSL | magenta |
| VASH1 | brown |
| VASH2 | magenta |
| VASN | salmon |
| VASP | lightcyan |
| VAT1 | blue |
| VAV1 | turquoise |
| VAV2 | yellow |
| VAV3 | blue |
| VAX2 | brown |
| VBP1 | blue |
| VCAM1 | turquoise |
| VCAN | purple |
| VCL | lightcyan |
| VCPIP1 | midnightblue |
| VDAC1 | turquoise |
| VDAC2 | green |
| VDAC3 | blue |
| VDR | lightcyan |
| VEGFA | turquoise |
| VEGFB | blue |
| VEGFC | blue |
| VENTX | black |
| VEPH1 | turquoise |
| VEZF1 | grey60 |
| VEZT | red |
| VGF | blue |
| VGLL1 | brown |
| VGLL3 | blue |
| VGLL4 | turquoise |
| VHL | yellow |
| VIL2 | brown |
| VILL | turquoise |
| VIM | blue |
| VIPR1 | blue |
| VKORC1 | turquoise |
| VKORC1L1 | turquoise |
| VLDLR | turquoise |
| VMA21 | blue |
| VMAC | turquoise |
| VMO1 | brown |
| VN1R1 | pink |
| VNN1 | brown |
| VNN2 | turquoise |
| VOPP1 | turquoise |
| VPREB3 | turquoise |
| VPS13A | pink |
| VPS13B | turquoise |
| VPS13C | turquoise |
| VPS13D | red |
| VPS24 | red |
| VPS25 | blue |
| VPS26A | yellow |
| VPS28 | darkred |
| VPS35 | turquoise |
| VPS36 | blue |
| VPS37A | blue |
| VPS37B | yellow |
| VPS37C | greenyellow |
| VPS4A | greenyellow |
| VPS4B | red |
| VPS52 | magenta |
| VPS53 | yellow |
| VPS54 | red |
| VPS72 | lightgreen |
| VRK1 | green |
| VSIG10 | yellow |
| VSIG10L | brown |
| VSIG2 | blue |
| VSIG4 | brown |
| VSNL1 | brown |
| VSTM2L | darkgreen |
| VTA1 | pink |
| VTCN1 | brown |
| VWA1 | tan |
| VWA2 | brown |
| VWA5A | blue |
| VWDE | brown |
| VWF | black |
| WAC | yellow |
| WARS | turquoise |
| WARS2 | red |
| WAS | turquoise |
| WASF1 | yellow |
| WASF2 | yellow |
| WASF3 | blue |
| WASH3P | yellow |
| WASH4P | yellow |
| WASH6P | yellow |
| WASL | turquoise |
| WBP1 | brown |
| WBP11 | cyan |
| WBP2 | yellow |
| WBP2NL | yellow |
| WBP5 | blue |
| WBSCR22 | blue |
| WBSCR27 | brown |
| WDFY1 | turquoise |
| WDFY2 | blue |
| WDFY3 | blue |
| WDFY4 | turquoise |
| WDHD1 | green |
| WDR1 | yellow |
| WDR12 | blue |
| WDR13 | turquoise |
| WDR18 | blue |
| WDR19 | blue |
| WDR20 | turquoise |
| WDR24 | yellow |
| WDR26 | yellow |
| WDR27 | pink |
| WDR3 | green |
| WDR31 | brown |
| WDR34 | green |
| WDR35 | cyan |
| WDR36 | salmon |
| WDR37 | yellow |
| WDR4 | blue |
| WDR43 | green |
| WDR45L | grey60 |
| WDR5 | blue |
| WDR52 | yellow |
| WDR53 | green |
| WDR54 | blue |
| WDR55 | brown |
| WDR6 | turquoise |
| WDR60 | yellow |
| WDR61 | turquoise |
| WDR62 | green |
| WDR67 | midnightblue |
| WDR74 | blue |
| WDR75 | green |
| WDR76 | green |
| WDR77 | blue |
| WDR78 | yellow |
| WDR81 | turquoise |
| WDR85 | yellow |
| WDR86 | blue |
| WDR90 | brown |
| WDR91 | yellow |
| WDSUB1 | brown |
| WDTC1 | yellow |
| WDYHV1 | blue |
| WEE1 | brown |
| WFDC1 | blue |
| WFDC2 | blue |
| WFDC3 | turquoise |
| WFS1 | blue |
| WHSC1 | yellow |
| WHSC1L1 | yellow |
| WHSC2 | yellow |
| WIBG | brown |
| WIPF1 | turquoise |
| WIPF2 | yellow |
| WIPI1 | brown |
| WISP1 | purple |
| WISP2 | blue |
| WIZ | yellow |
| WNK1 | yellow |
| WNK2 | magenta |
| WNT10A | turquoise |
| WNT2 | purple |
| WNT2B | yellow |
| WNT4 | turquoise |
| WNT5A | turquoise |
| WNT5B | blue |
| WNT6 | magenta |
| WNT7B | turquoise |
| WNT9A | turquoise |
| WRAP53 | yellow |
| WRB | brown |
| WRN | yellow |
| WRNIP1 | magenta |
| WSB1 | blue |
| WSB2 | red |
| WTAP | turquoise |
| WTIP | blue |
| WWC1 | turquoise |
| WWC2 | lightcyan |
| WWC3 | yellow |
| WWOX | blue |
| WWP1 | blue |
| WWP2 | yellow |
| WWTR1 | turquoise |
| XAF1 | turquoise |
| XBP1 | turquoise |
| XG | purple |
| XIAP | blue |
| XIST | blue |
| XKR6 | turquoise |
| XKR9 | yellow |
| XPC | turquoise |
| XPNPEP3 | yellow |
| XPO5 | magenta |
| XPO7 | yellow |
| XPOT | turquoise |
| XPR1 | blue |
| XRCC2 | green |
| XRCC3 | yellow |
| XRCC6 | blue |
| XRCC6BP1 | turquoise |
| XRN1 | turquoise |
| XRN2 | blue |
| XRRA1 | yellow |
| XXbac-B135H6.15 | brown |
| XXbac-B461K10.4 | yellow |
| XXbac-B476C20.14 | yellow |
| XXbac-B562F10.11 | yellow |
| XXbac-BPG246D15.8 | turquoise |
| XXbac-BPG246D15.9 | turquoise |
| XXbac-BPG248L24.12 | brown |
| XXbac-BPG27H4.7 | magenta |
| XXbac-BPG296P20.14 | magenta |
| XXbac-BPG299F13.14 | turquoise |
| XXbac-BPG55C20.1 | blue |
| XXyac-R12DG2.2 | yellow |
| XXyac-R12DG2.3 | yellow |
| XXyac-YRM2039.2 | yellow |
| XXyac-YX65C7_A.2 | blue |
| XYLB | turquoise |
| XYLT1 | blue |
| XYLT2 | turquoise |
| Y_RNA | royalblue |
| YAF2 | turquoise |
| YAP1 | turquoise |
| YARS | green |
| YARS2 | cyan |
| YBX1 | green |
| YDJC | blue |
| YEATS2 | green |
| YEATS4 | blue |
| YES1 | turquoise |
| YIF1A | turquoise |
| YIF1B | blue |
| YIPF1 | tan |
| YIPF2 | turquoise |
| YIPF3 | magenta |
| YIPF4 | red |
| YIPF6 | red |
| YJEFN3 | yellow |
| YKT6 | turquoise |
| YLPM1 | yellow |
| YME1L1 | green |
| YOD1 | green |
| YPEL1 | yellow |
| YPEL2 | blue |
| YPEL3 | turquoise |
| YRDC | green |
| YTHDC2 | turquoise |
| YTHDF3 | midnightblue |
| YWHAE | lightgreen |
| YWHAG | blue |
| YWHAH | green |
| YWHAQ | green |
| YWHAZ | midnightblue |
| YY1AP1 | yellow |
| YY2 | yellow |
| Z73979.1 | lightyellow |
| Z73979.2 | blue |
| Z83843.1 | blue |
| Z83844.1 | yellow |
| Z83851.1 | blue |
| Z83851.3 | blue |
| Z83851.4 | brown |
| Z97630.1 | turquoise |
| Z97652.2 | yellow |
| Z98256.1 | blue |
| Z98258.1 | blue |
| Z99572.1 | tan |
| ZADH2 | red |
| ZAP70 | turquoise |
| ZBED1 | purple |
| ZBED3 | yellow |
| ZBED4 | yellow |
| ZBED5 | turquoise |
| ZBP1 | turquoise |
| ZBTB10 | midnightblue |
| ZBTB12 | magenta |
| ZBTB16 | blue |
| ZBTB2 | pink |
| ZBTB20 | blue |
| ZBTB22 | magenta |
| ZBTB24 | red |
| ZBTB25 | turquoise |
| ZBTB32 | turquoise |
| ZBTB33 | red |
| ZBTB37 | turquoise |
| ZBTB38 | turquoise |
| ZBTB4 | lightyellow |
| ZBTB41 | green |
| ZBTB42 | brown |
| ZBTB43 | blue |
| ZBTB44 | red |
| ZBTB45 | yellow |
| ZBTB46 | turquoise |
| ZBTB47 | blue |
| ZBTB5 | brown |
| ZBTB6 | red |
| ZBTB7A | yellow |
| ZBTB7B | brown |
| ZBTB7C | brown |
| ZBTB8A | red |
| ZC3H12A | turquoise |
| ZC3H12C | brown |
| ZC3H12D | turquoise |
| ZC3H13 | blue |
| ZC3H15 | green |
| ZC3H18 | yellow |
| ZC3H3 | darkred |
| ZC3H7B | yellow |
| ZC3H8 | red |
| ZC3HAV1 | turquoise |
| ZC3HAV1L | turquoise |
| ZC4H2 | yellow |
| ZCCHC10 | brown |
| ZCCHC11 | turquoise |
| ZCCHC14 | yellow |
| ZCCHC17 | blue |
| ZCCHC2 | turquoise |
| ZCCHC24 | blue |
| ZCCHC6 | blue |
| ZCCHC7 | turquoise |
| ZCRB1 | red |
| ZCWPW1 | turquoise |
| ZDBF2 | turquoise |
| ZDHHC1 | blue |
| ZDHHC11 | yellow |
| ZDHHC12 | blue |
| ZDHHC13 | red |
| ZDHHC14 | turquoise |
| ZDHHC16 | lightgreen |
| ZDHHC17 | turquoise |
| ZDHHC18 | yellow |
| ZDHHC2 | turquoise |
| ZDHHC20 | red |
| ZDHHC21 | pink |
| ZDHHC23 | brown |
| ZDHHC4 | turquoise |
| ZDHHC5 | greenyellow |
| ZDHHC8 | yellow |
| ZDHHC9 | turquoise |
| ZEB1 | blue |
| ZEB2 | brown |
| ZEB2AS | brown |
| ZER1 | yellow |
| ZFAND1 | midnightblue |
| ZFAND2A | green |
| ZFAND3 | yellow |
| ZFAND5 | blue |
| ZFAND6 | blue |
| ZFAT | turquoise |
| ZFHX4 | blue |
| ZFP3 | yellow |
| ZFP30 | pink |
| ZFP36 | turquoise |
| ZFP36L1 | lightyellow |
| ZFP36L2 | turquoise |
| ZFP41 | darkred |
| ZFP57 | blue |
| ZFP62 | brown |
| ZFP64 | brown |
| ZFP90 | blue |
| ZFPL1 | turquoise |
| ZFPM1 | yellow |
| ZFPM2 | blue |
| ZFR | brown |
| ZFYVE16 | blue |
| ZFYVE19 | turquoise |
| ZFYVE20 | blue |
| ZFYVE21 | turquoise |
| ZFYVE28 | turquoise |
| ZFYVE9 | brown |
| ZG16B | brown |
| ZGLP1 | blue |
| ZHX1 | midnightblue |
| ZHX2 | yellow |
| ZHX3 | lightyellow |
| ZIM2 | blue |
| ZKSCAN1 | greenyellow |
| ZKSCAN3 | magenta |
| ZKSCAN4 | turquoise |
| ZMAT1 | blue |
| ZMAT3 | blue |
| ZMIZ1 | yellow |
| ZMIZ2 | yellow |
| ZMPSTE24 | turquoise |
| ZMYM1 | pink |
| ZMYM4 | pink |
| ZMYM6 | turquoise |
| ZMYND11 | brown |
| ZMYND12 | brown |
| ZMYND15 | brown |
| ZMYND19 | blue |
| ZMYND8 | yellow |
| ZNF10 | pink |
| ZNF100 | pink |
| ZNF101 | turquoise |
| ZNF107 | pink |
| ZNF117 | brown |
| ZNF12 | yellow |
| ZNF121 | red |
| ZNF124 | red |
| ZNF131 | yellow |
| ZNF132 | blue |
| ZNF133 | brown |
| ZNF134 | pink |
| ZNF135 | pink |
| ZNF136 | turquoise |
| ZNF137P | pink |
| ZNF138 | pink |
| ZNF141 | turquoise |
| ZNF142 | yellow |
| ZNF146 | pink |
| ZNF154 | pink |
| ZNF16 | darkred |
| ZNF160 | pink |
| ZNF165 | green |
| ZNF167 | turquoise |
| ZNF169 | turquoise |
| ZNF175 | blue |
| ZNF177 | pink |
| ZNF184 | magenta |
| ZNF185 | turquoise |
| ZNF189 | blue |
| ZNF192 | turquoise |
| ZNF193 | blue |
| ZNF195 | red |
| ZNF197 | blue |
| ZNF20 | red |
| ZNF205 | brown |
| ZNF211 | pink |
| ZNF212 | yellow |
| ZNF213 | greenyellow |
| ZNF217 | greenyellow |
| ZNF219 | brown |
| ZNF22 | pink |
| ZNF223 | pink |
| ZNF224 | turquoise |
| ZNF226 | pink |
| ZNF229 | brown |
| ZNF232 | magenta |
| ZNF238 | yellow |
| ZNF239 | blue |
| ZNF24 | red |
| ZNF248 | yellow |
| ZNF250 | darkred |
| ZNF251 | darkred |
| ZNF252 | darkred |
| ZNF254 | pink |
| ZNF256 | pink |
| ZNF257 | turquoise |
| ZNF259 | red |
| ZNF260 | pink |
| ZNF264 | pink |
| ZNF266 | turquoise |
| ZNF267 | turquoise |
| ZNF273 | brown |
| ZNF274 | pink |
| ZNF275 | turquoise |
| ZNF276 | turquoise |
| ZNF28 | pink |
| ZNF280C | red |
| ZNF280D | blue |
| ZNF282 | brown |
| ZNF283 | pink |
| ZNF286 | magenta |
| ZNF287 | blue |
| ZNF292 | red |
| ZNF295 | yellow |
| ZNF296 | yellow |
| ZNF3 | brown |
| ZNF300 | yellow |
| ZNF302 | pink |
| ZNF304 | pink |
| ZNF316 | yellow |
| ZNF318 | magenta |
| ZNF32 | brown |
| ZNF320 | pink |
| ZNF321 | pink |
| ZNF322A | brown |
| ZNF323 | brown |
| ZNF329 | pink |
| ZNF32OS1 | brown |
| ZNF330 | brown |
| ZNF331 | brown |
| ZNF333 | pink |
| ZNF334 | blue |
| ZNF337 | yellow |
| ZNF33A | yellow |
| ZNF33B | brown |
| ZNF34 | darkred |
| ZNF343 | greenyellow |
| ZNF346 | blue |
| ZNF347 | pink |
| ZNF35 | yellow |
| ZNF354A | yellow |
| ZNF354B | yellow |
| ZNF354C | blue |
| ZNF358 | magenta |
| ZNF362 | yellow |
| ZNF365 | lightcyan |
| ZNF366 | turquoise |
| ZNF367 | green |
| ZNF37A | turquoise |
| ZNF37B | turquoise |
| ZNF382 | pink |
| ZNF384 | cyan |
| ZNF385A | lightyellow |
| ZNF385D | black |
| ZNF391 | yellow |
| ZNF394 | turquoise |
| ZNF395 | brown |
| ZNF397 | red |
| ZNF398 | lightgreen |
| ZNF414 | yellow |
| ZNF415 | pink |
| ZNF417 | pink |
| ZNF418 | pink |
| ZNF419 | pink |
| ZNF425 | brown |
| ZNF426 | pink |
| ZNF428 | brown |
| ZNF429 | red |
| ZNF43 | pink |
| ZNF431 | pink |
| ZNF432 | darkgreen |
| ZNF433 | pink |
| ZNF435 | magenta |
| ZNF436 | blue |
| ZNF439 | pink |
| ZNF44 | blue |
| ZNF440 | red |
| ZNF441 | turquoise |
| ZNF443 | red |
| ZNF444 | magenta |
| ZNF454 | yellow |
| ZNF460 | pink |
| ZNF461 | pink |
| ZNF462 | brown |
| ZNF467 | blue |
| ZNF468 | pink |
| ZNF469 | yellow |
| ZNF470 | pink |
| ZNF471 | pink |
| ZNF48 | magenta |
| ZNF480 | blue |
| ZNF483 | blue |
| ZNF484 | turquoise |
| ZNF485 | blue |
| ZNF487 | blue |
| ZNF491 | blue |
| ZNF493 | pink |
| ZNF496 | magenta |
| ZNF497 | yellow |
| ZNF501 | pink |
| ZNF502 | brown |
| ZNF503 | lightcyan |
| ZNF506 | pink |
| ZNF507 | pink |
| ZNF511 | blue |
| ZNF512 | yellow |
| ZNF512B | brown |
| ZNF513 | turquoise |
| ZNF514 | brown |
| ZNF516 | blue |
| ZNF517 | darkred |
| ZNF518A | red |
| ZNF518B | darkgreen |
| ZNF519 | red |
| ZNF521 | blue |
| ZNF524 | blue |
| ZNF525 | pink |
| ZNF527 | pink |
| ZNF528 | pink |
| ZNF529 | pink |
| ZNF532 | yellow |
| ZNF542 | pink |
| ZNF544 | pink |
| ZNF548 | pink |
| ZNF549 | pink |
| ZNF550 | pink |
| ZNF551 | pink |
| ZNF552 | blue |
| ZNF558 | red |
| ZNF559 | pink |
| ZNF561 | pink |
| ZNF562 | red |
| ZNF564 | turquoise |
| ZNF566 | pink |
| ZNF567 | pink |
| ZNF568 | pink |
| ZNF57 | turquoise |
| ZNF570 | pink |
| ZNF572 | midnightblue |
| ZNF573 | pink |
| ZNF577 | darkgreen |
| ZNF579 | magenta |
| ZNF580 | yellow |
| ZNF581 | salmon |
| ZNF582 | blue |
| ZNF584 | pink |
| ZNF586 | turquoise |
| ZNF587 | pink |
| ZNF589 | turquoise |
| ZNF592 | yellow |
| ZNF593 | blue |
| ZNF594 | yellow |
| ZNF595 | blue |
| ZNF596 | blue |
| ZNF597 | turquoise |
| ZNF598 | blue |
| ZNF599 | pink |
| ZNF605 | pink |
| ZNF606 | pink |
| ZNF607 | pink |
| ZNF608 | blue |
| ZNF609 | yellow |
| ZNF611 | pink |
| ZNF613 | darkgreen |
| ZNF614 | pink |
| ZNF616 | pink |
| ZNF618 | yellow |
| ZNF619 | turquoise |
| ZNF620 | black |
| ZNF621 | yellow |
| ZNF623 | darkred |
| ZNF625 | pink |
| ZNF626 | tan |
| ZNF629 | yellow |
| ZNF630 | yellow |
| ZNF639 | green |
| ZNF642 | yellow |
| ZNF643 | blue |
| ZNF644 | pink |
| ZNF649 | darkgreen |
| ZNF652 | blue |
| ZNF655 | turquoise |
| ZNF66 | pink |
| ZNF660 | blue |
| ZNF662 | blue |
| ZNF664 | magenta |
| ZNF667 | pink |
| ZNF669 | green |
| ZNF670 | green |
| ZNF671 | turquoise |
| ZNF673 | yellow |
| ZNF674 | yellow |
| ZNF675 | pink |
| ZNF678 | red |
| ZNF680 | pink |
| ZNF681 | pink |
| ZNF682 | pink |
| ZNF684 | pink |
| ZNF687 | lightgreen |
| ZNF689 | darkgreen |
| ZNF69 | pink |
| ZNF691 | yellow |
| ZNF692 | magenta |
| ZNF695 | green |
| ZNF696 | darkred |
| ZNF697 | blue |
| ZNF7 | darkred |
| ZNF70 | yellow |
| ZNF701 | pink |
| ZNF702P | red |
| ZNF703 | lightyellow |
| ZNF704 | yellow |
| ZNF706 | midnightblue |
| ZNF707 | darkred |
| ZNF708 | pink |
| ZNF709 | black |
| ZNF71 | pink |
| ZNF711 | red |
| ZNF713 | brown |
| ZNF714 | pink |
| ZNF717 | brown |
| ZNF718 | pink |
| ZNF721 | pink |
| ZNF724P | green |
| ZNF726 | pink |
| ZNF736 | pink |
| ZNF737 | blue |
| ZNF738 | pink |
| ZNF74 | blue |
| ZNF746 | yellow |
| ZNF749 | pink |
| ZNF750 | magenta |
| ZNF75A | red |
| ZNF76 | magenta |
| ZNF761 | pink |
| ZNF763 | turquoise |
| ZNF765 | pink |
| ZNF766 | pink |
| ZNF767 | yellow |
| ZNF768 | turquoise |
| ZNF77 | brown |
| ZNF770 | red |
| ZNF771 | brown |
| ZNF772 | pink |
| ZNF773 | pink |
| ZNF774 | brown |
| ZNF775 | turquoise |
| ZNF776 | pink |
| ZNF777 | lightgreen |
| ZNF780A | turquoise |
| ZNF780B | turquoise |
| ZNF782 | yellow |
| ZNF783 | yellow |
| ZNF785 | yellow |
| ZNF786 | lightgreen |
| ZNF787 | blue |
| ZNF789 | yellow |
| ZNF790 | pink |
| ZNF791 | turquoise |
| ZNF792 | turquoise |
| ZNF793 | pink |
| ZNF8 | pink |
| ZNF808 | pink |
| ZNF81 | turquoise |
| ZNF812 | blue |
| ZNF813 | pink |
| ZNF814 | pink |
| ZNF815 | turquoise |
| ZNF816 | pink |
| ZNF818P | pink |
| ZNF821 | magenta |
| ZNF823 | red |
| ZNF827 | yellow |
| ZNF828 | yellow |
| ZNF829 | pink |
| ZNF83 | pink |
| ZNF833P | green |
| ZNF836 | pink |
| ZNF837 | brown |
| ZNF84 | pink |
| ZNF841 | darkgreen |
| ZNF844 | blue |
| ZNF845 | pink |
| ZNF846 | black |
| ZNF85 | pink |
| ZNF852 | turquoise |
| ZNF860 | blue |
| ZNF862 | yellow |
| ZNF865 | yellow |
| ZNF879 | black |
| ZNF880 | pink |
| ZNF883 | brown |
| ZNF891 | pink |
| ZNF90 | pink |
| ZNF91 | pink |
| ZNF92 | pink |
| ZNF98 | pink |
| ZNFX1 | turquoise |
| ZNHIT1 | turquoise |
| ZNHIT2 | blue |
| ZNHIT6 | red |
| ZNRD1 | darkturquoise |
| ZNRF1 | yellow |
| ZNRF2 | turquoise |
| ZNRF3 | turquoise |
| ZP3 | turquoise |
| ZRANB1 | yellow |
| ZRANB2 | pink |
| ZRANB3 | red |
| ZRSR2 | turquoise |
| ZSCAN12 | turquoise |
| ZSCAN18 | pink |
| ZSCAN2 | magenta |
| ZSCAN21 | brown |
| ZSCAN23 | blue |
| ZSCAN30 | red |
| ZSWIM4 | yellow |
| ZSWIM6 | black |
| ZSWIM7 | greenyellow |
| ZUFSP | red |
| ZW10 | red |
| ZWILCH | green |
| ZWINT | green |
| ZYG11A | blue |
| ZYX | lightyellow |
